# Supplementary material for: Yeast population dynamics in Brazilian bioethanol production
Source: G3 (Bethesda). 2023 Jun 2;13(7):jkad104. doi: 10.1093/g3journal/jkad104 (PMC10320141; doi:10.1093/g3journal/jkad104)

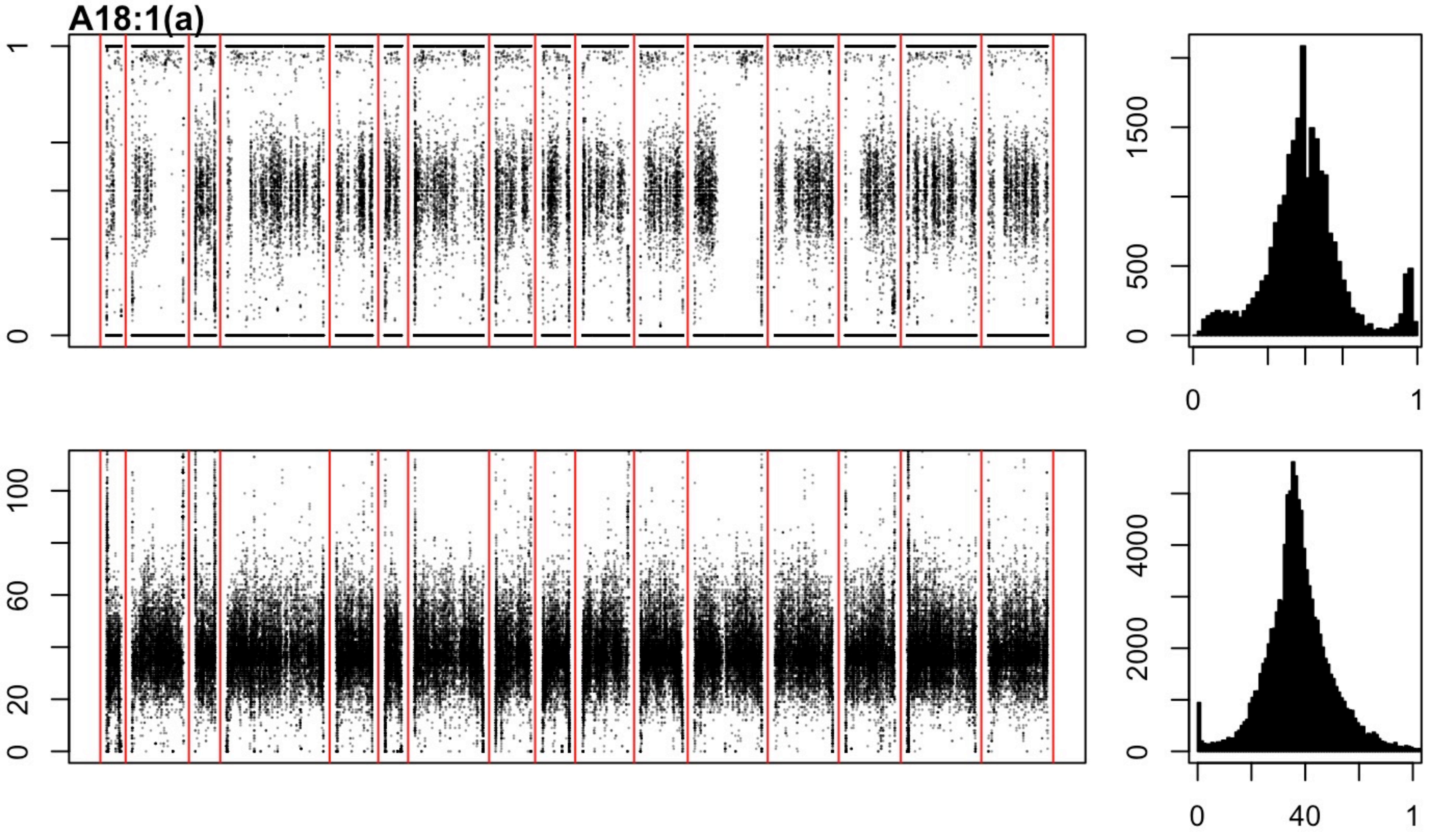
A18:1(a)
1
0
1500
500
0
0
1
100
60
20
0
4000
2000
0
0
40
1

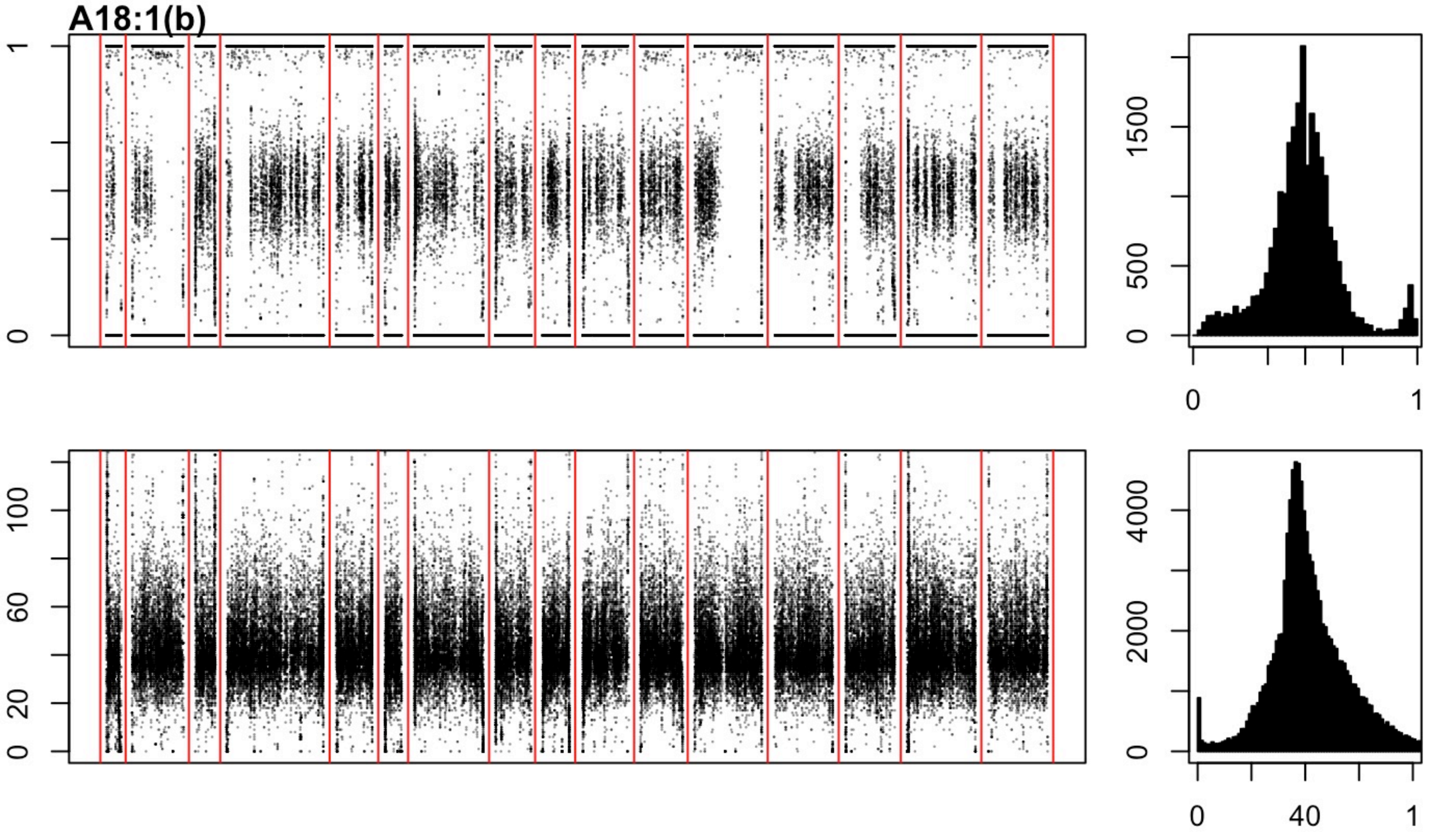

A18:1(b)
1
0
1500
500
0
0
1
100
60
20
0
4000
2000
0
0
40
1

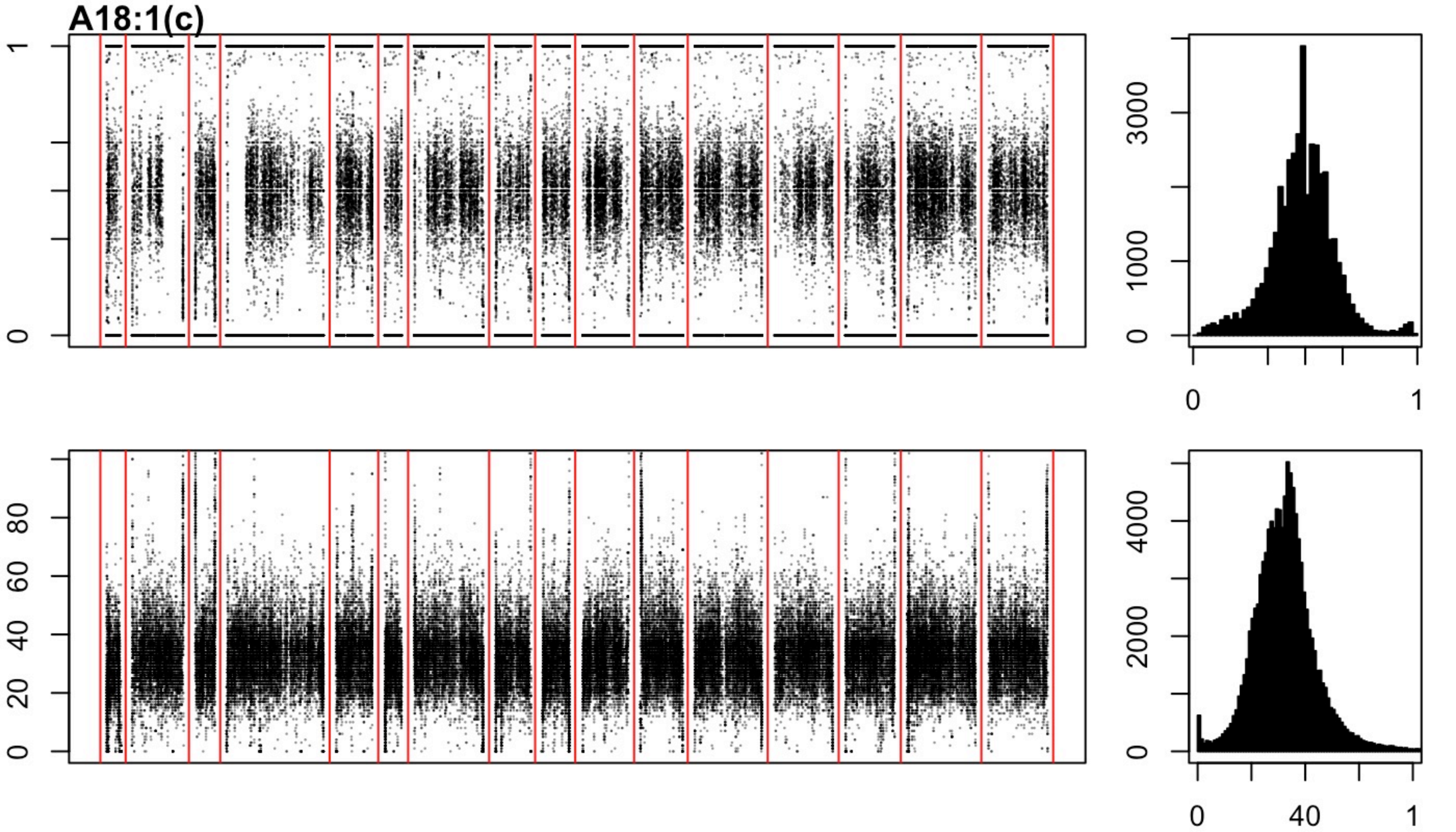
A18:1(c)
1
0
3000
1000
0
0
1
80
60
40
20
0
4000
2000
0
0
40
1

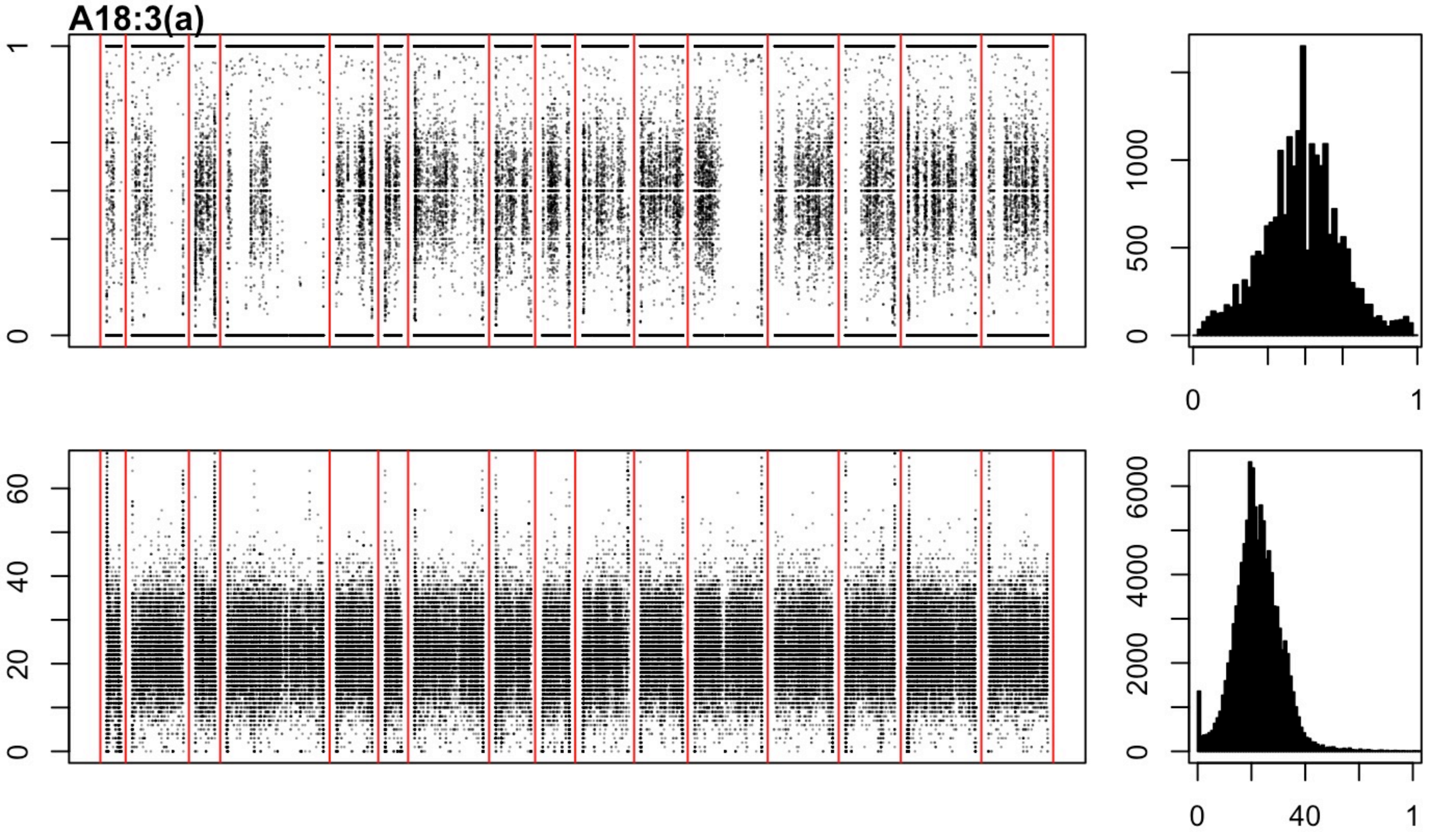
A18:3(a)
1
0
1000
500
0
0
1
60
40
20
0
6000
4000
2000
0
0
40
1

A18:3(b)

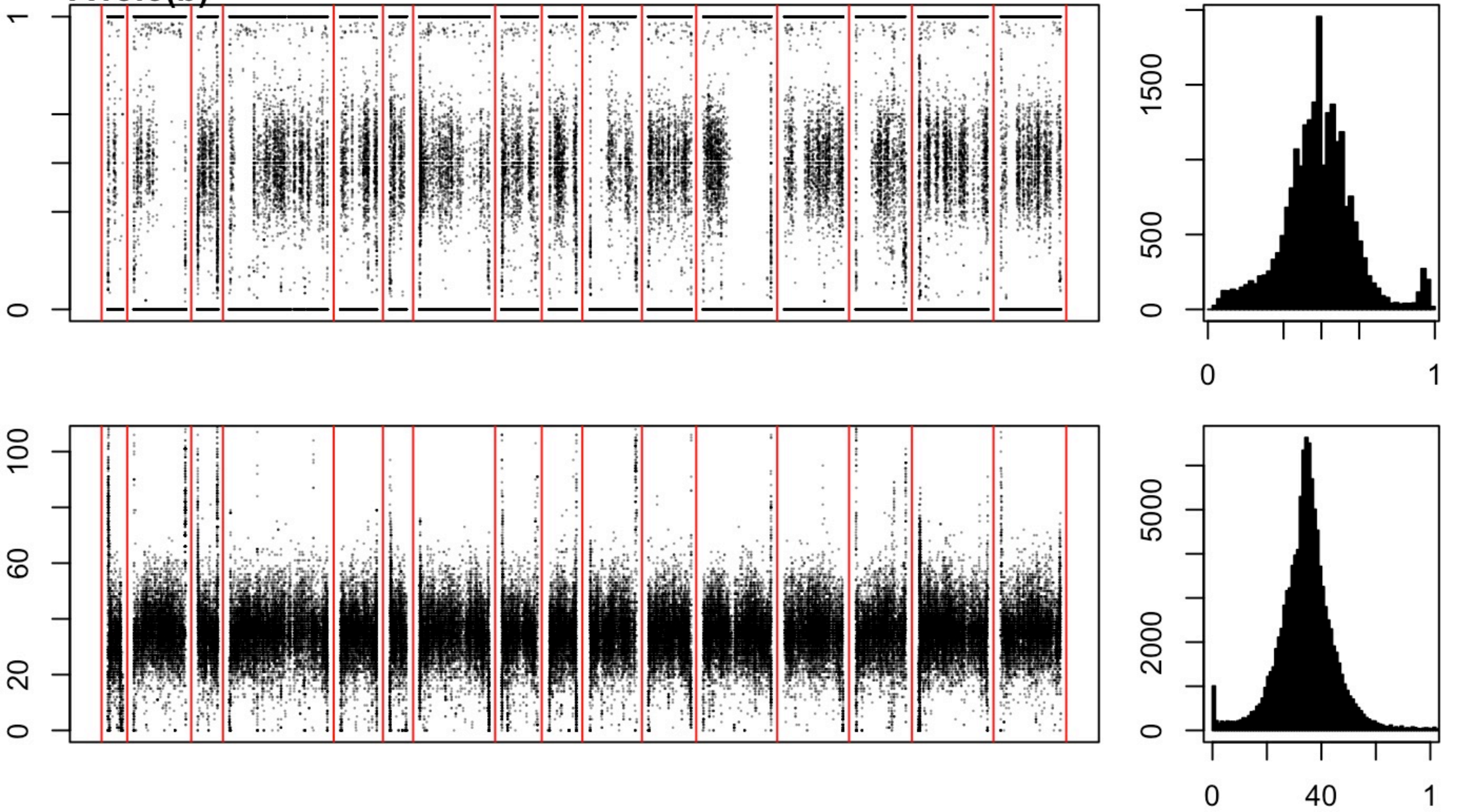

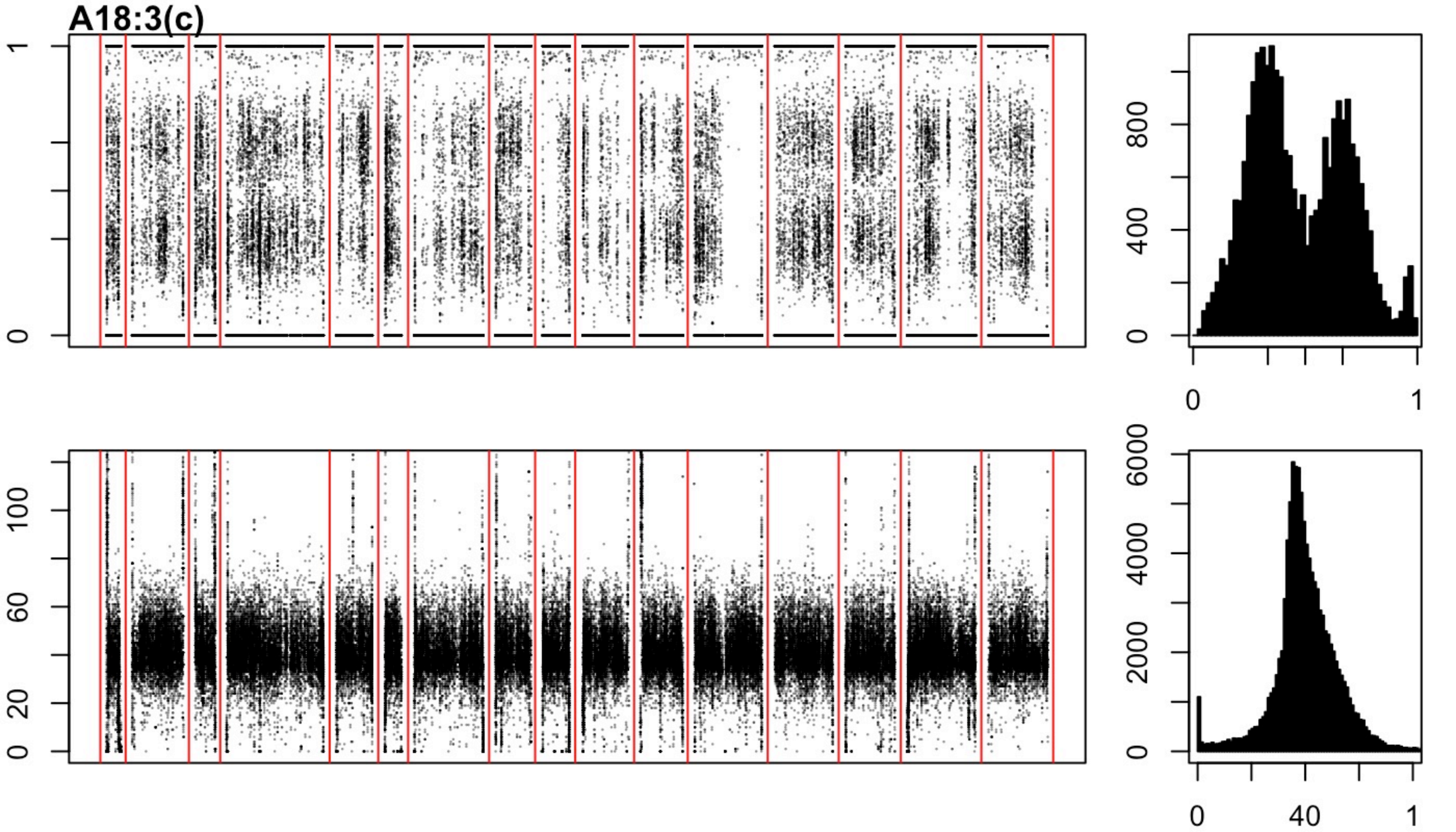

A18:3(c)
1
0
800
400
0
0
1
100
60
20
0
6000
4000
2000
0
0
40
1

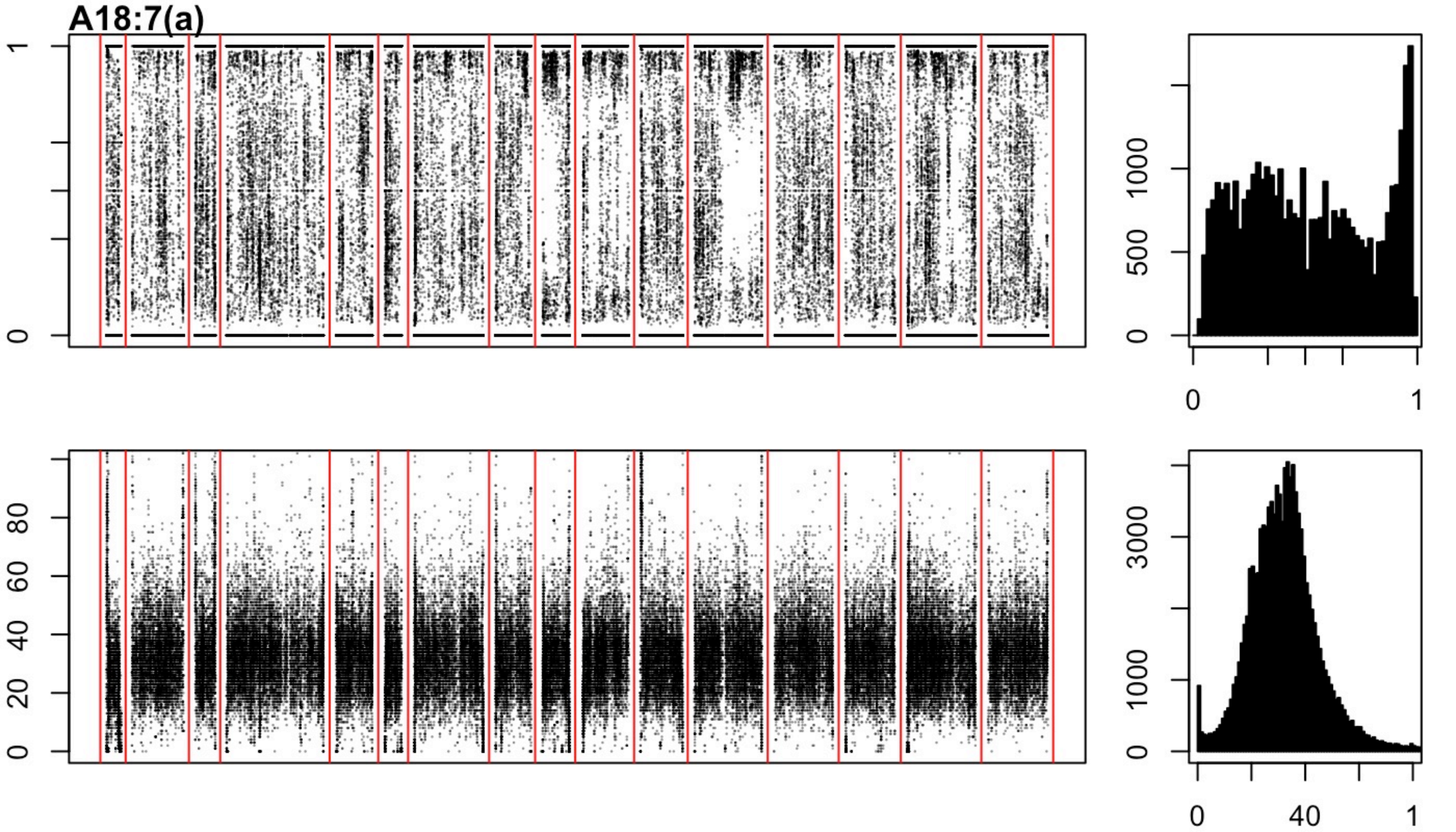
A18:7(a)
1
0
1000
500
0
0
1
80
60
40
20
0
3000
1000
0
0
40
1

A18:7(b)

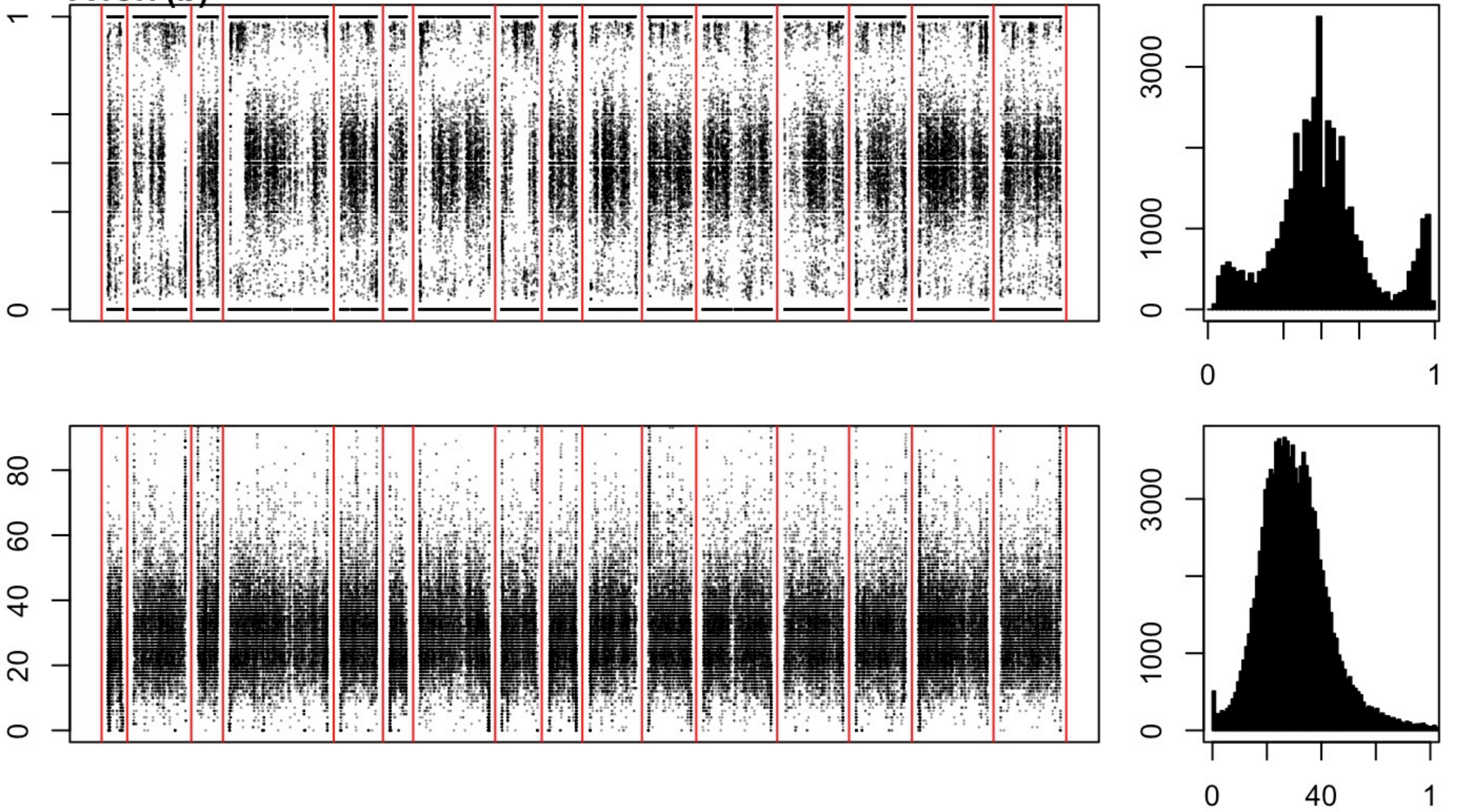

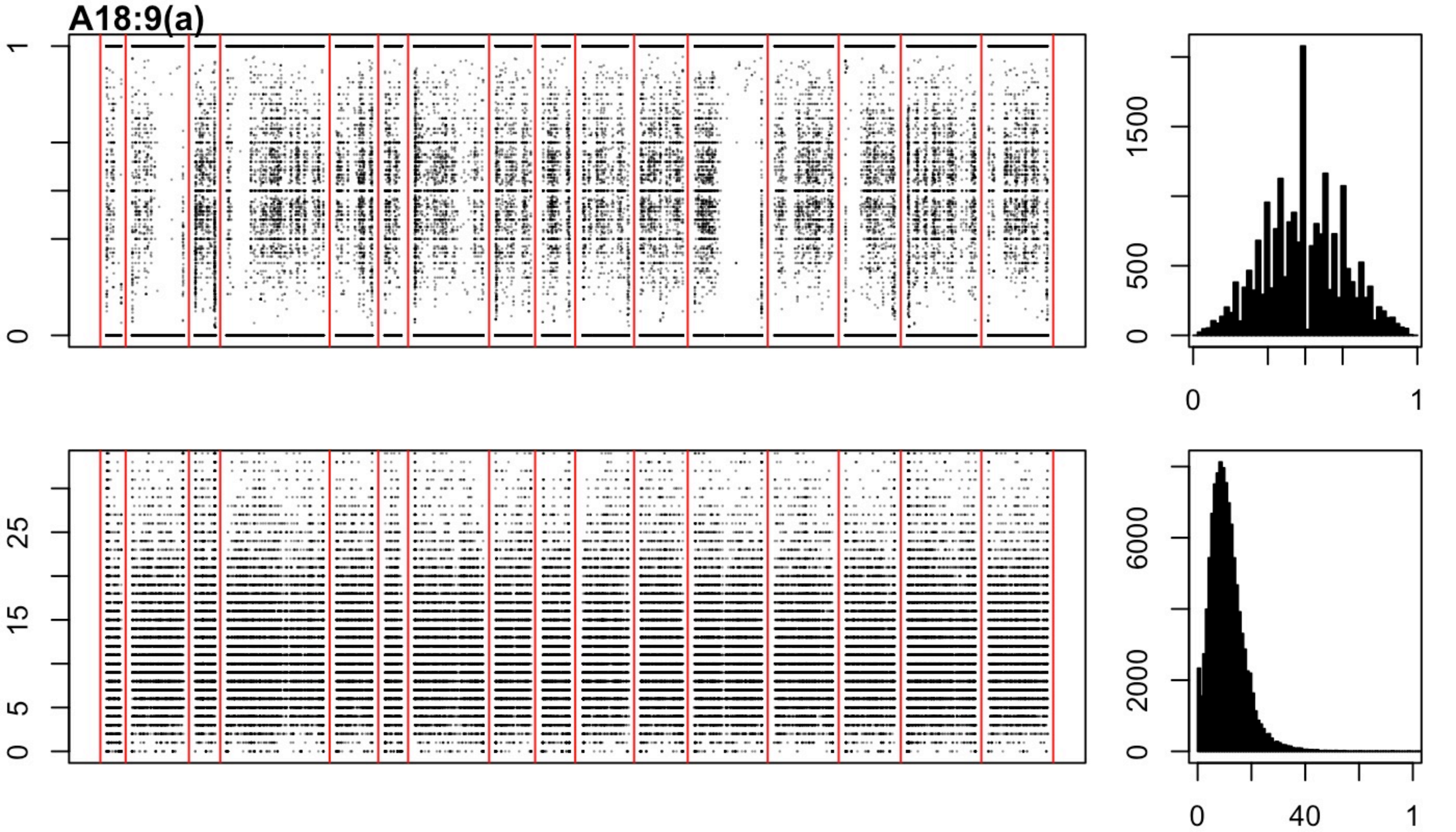

A18:9(a)
0
1
0
500
1500
0
1
0
5
15
25
0
2000
6000
0
40
1

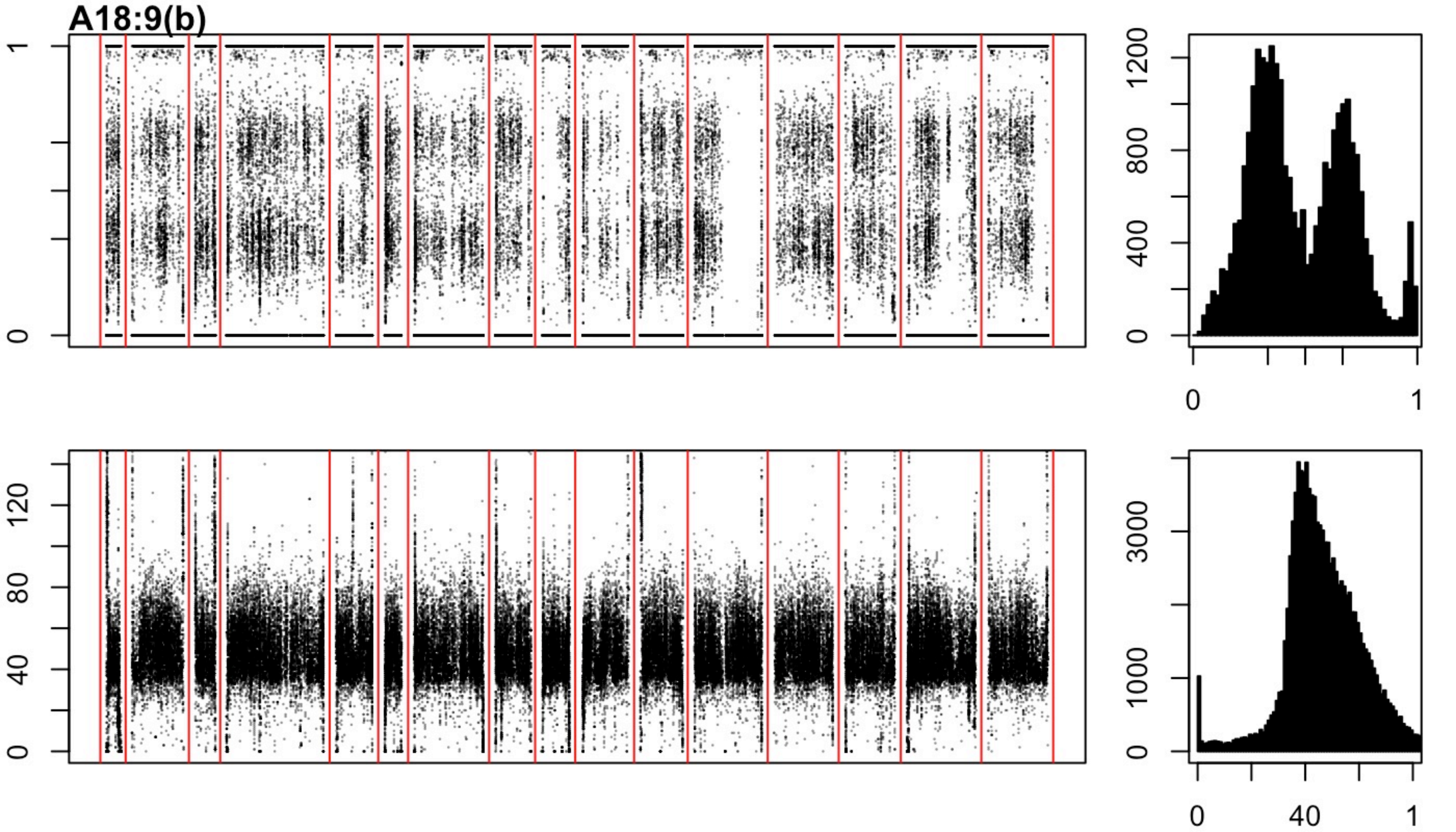
A18:9(b)
1
0
1200
800
400
0
0
1
120
80
40
0
3000
1000
0
0
40
1

A18:9(c)

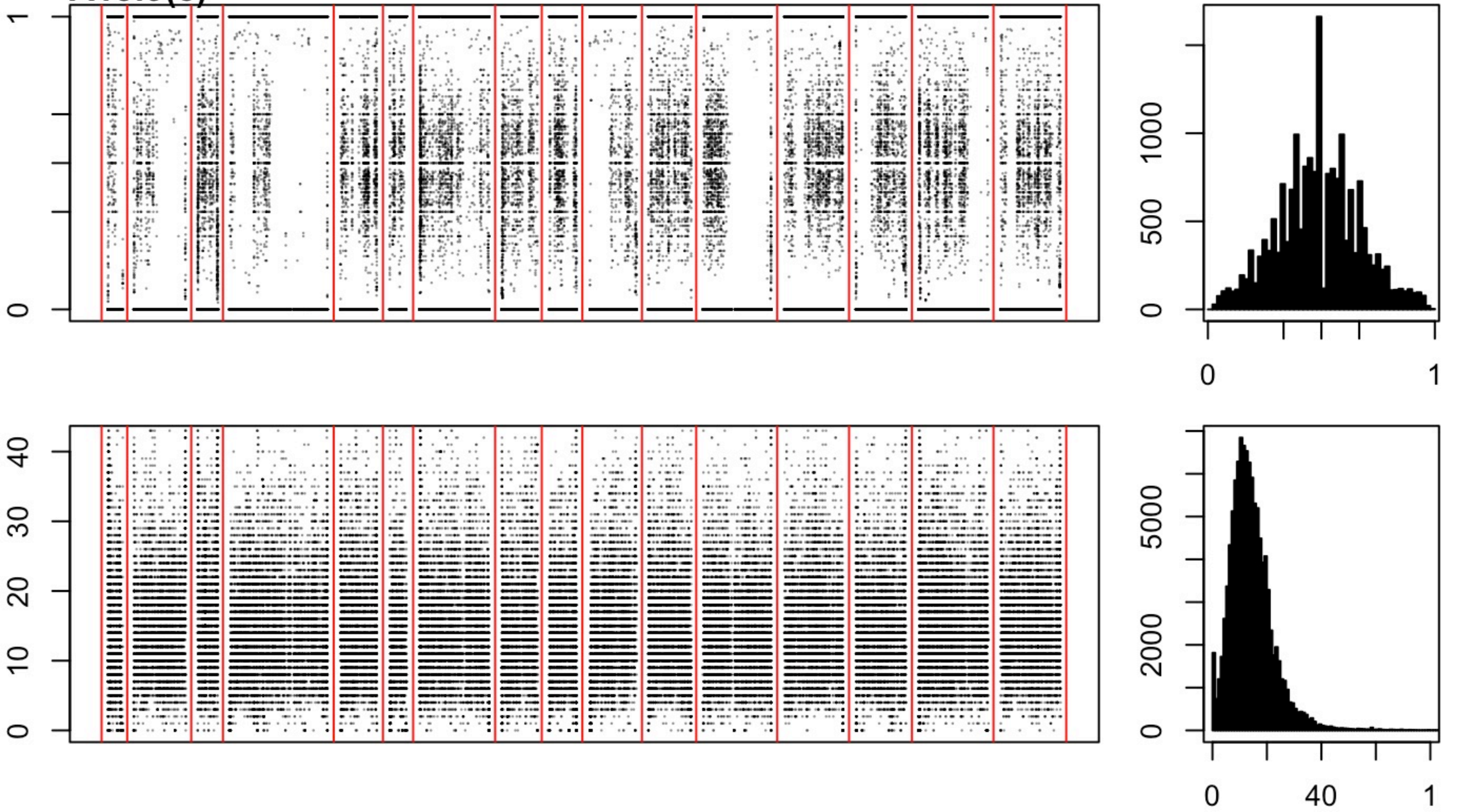

A18:17(a)

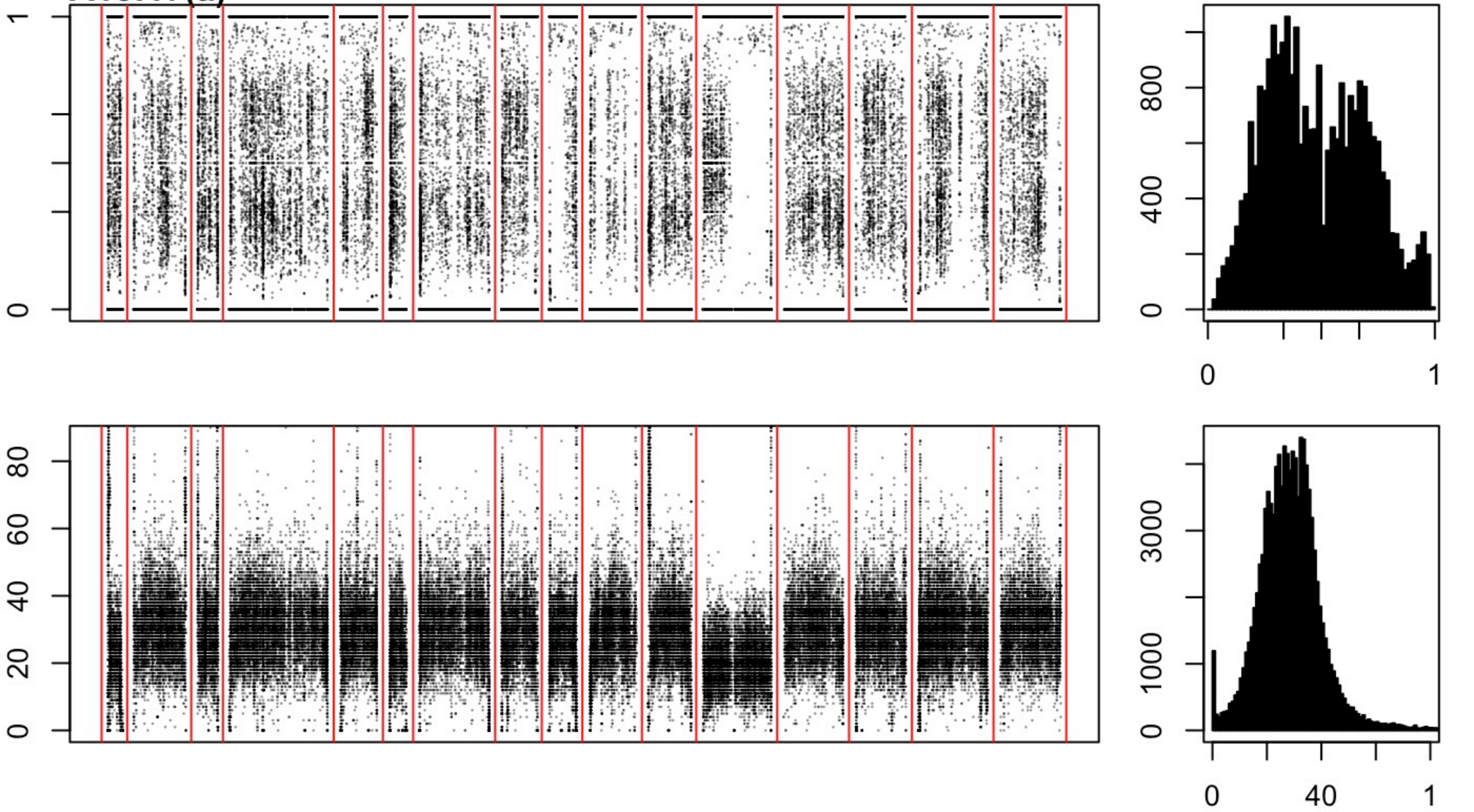

A18:17(b)

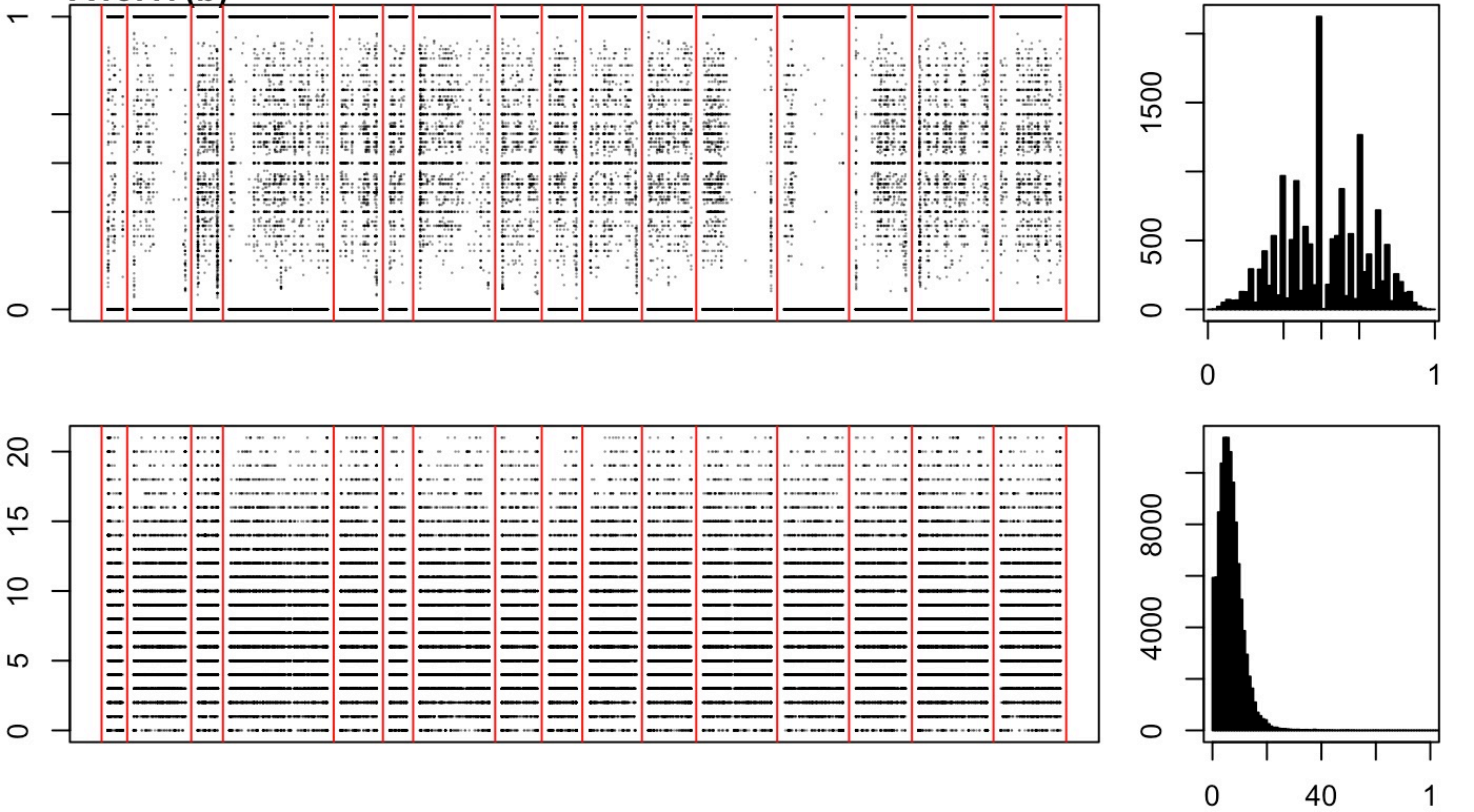

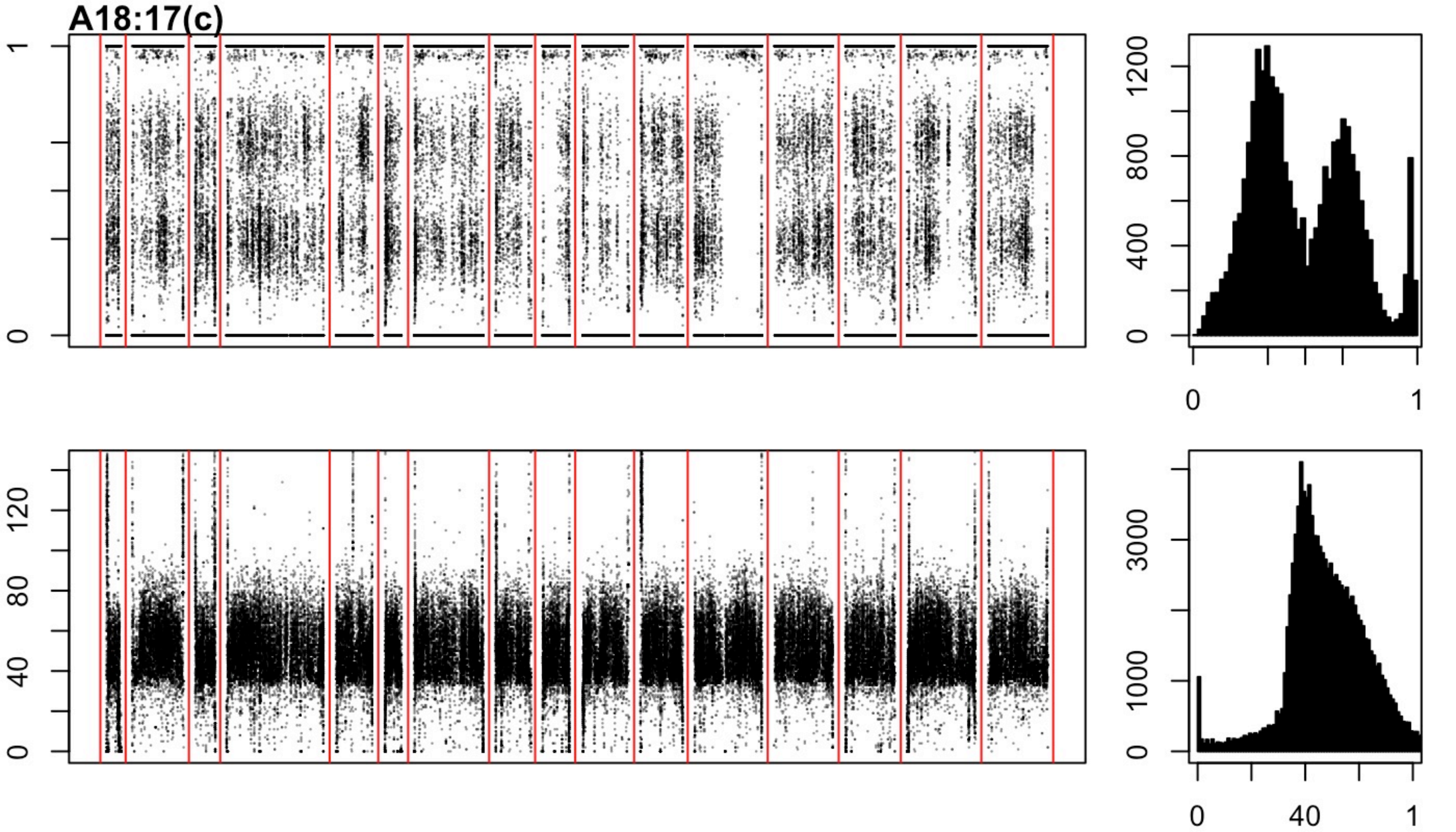

A18:17(c)
1
0
1200
800
400
0
0
1
120
80
40
0
3000
1000
0
40
1

A18:27(a)

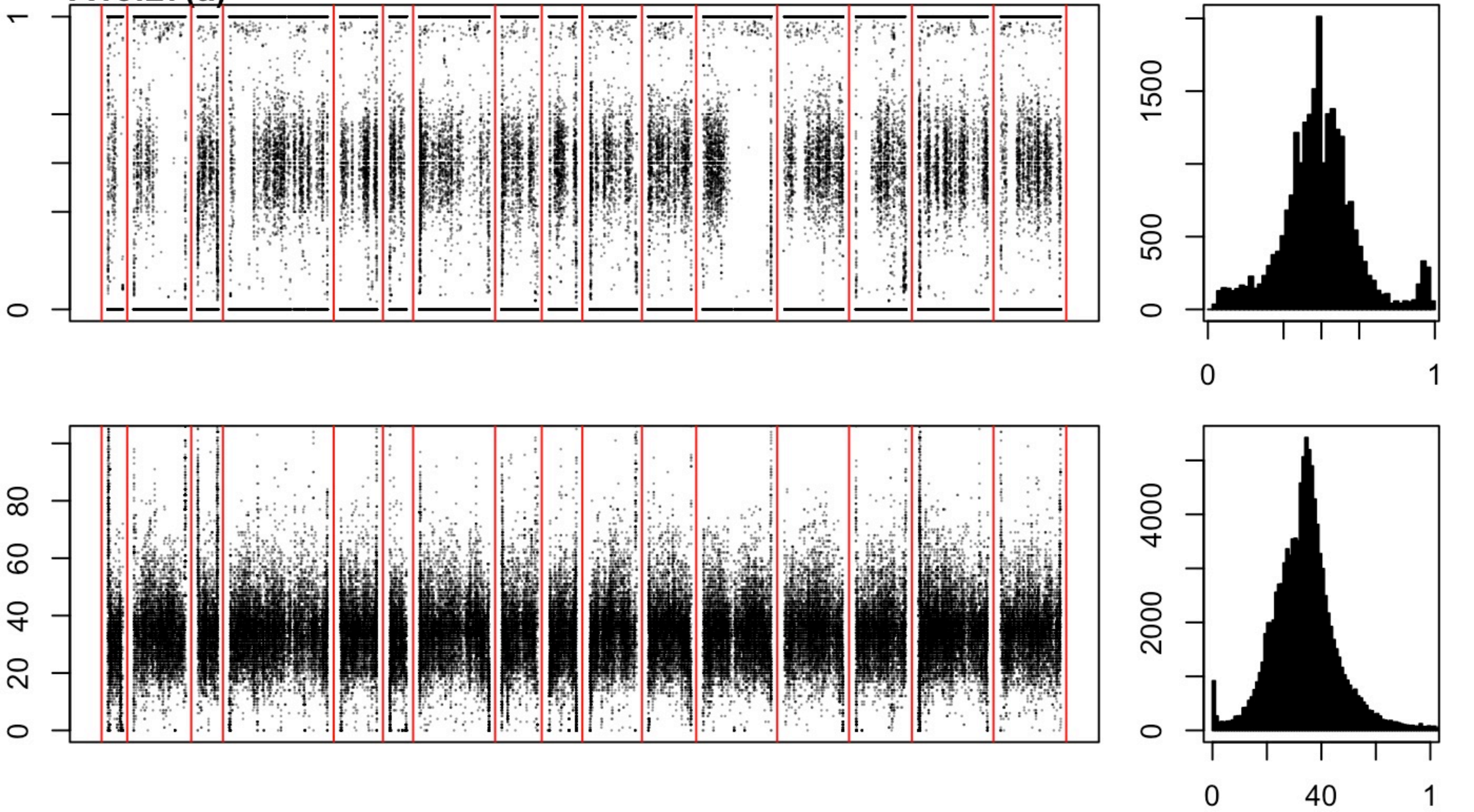

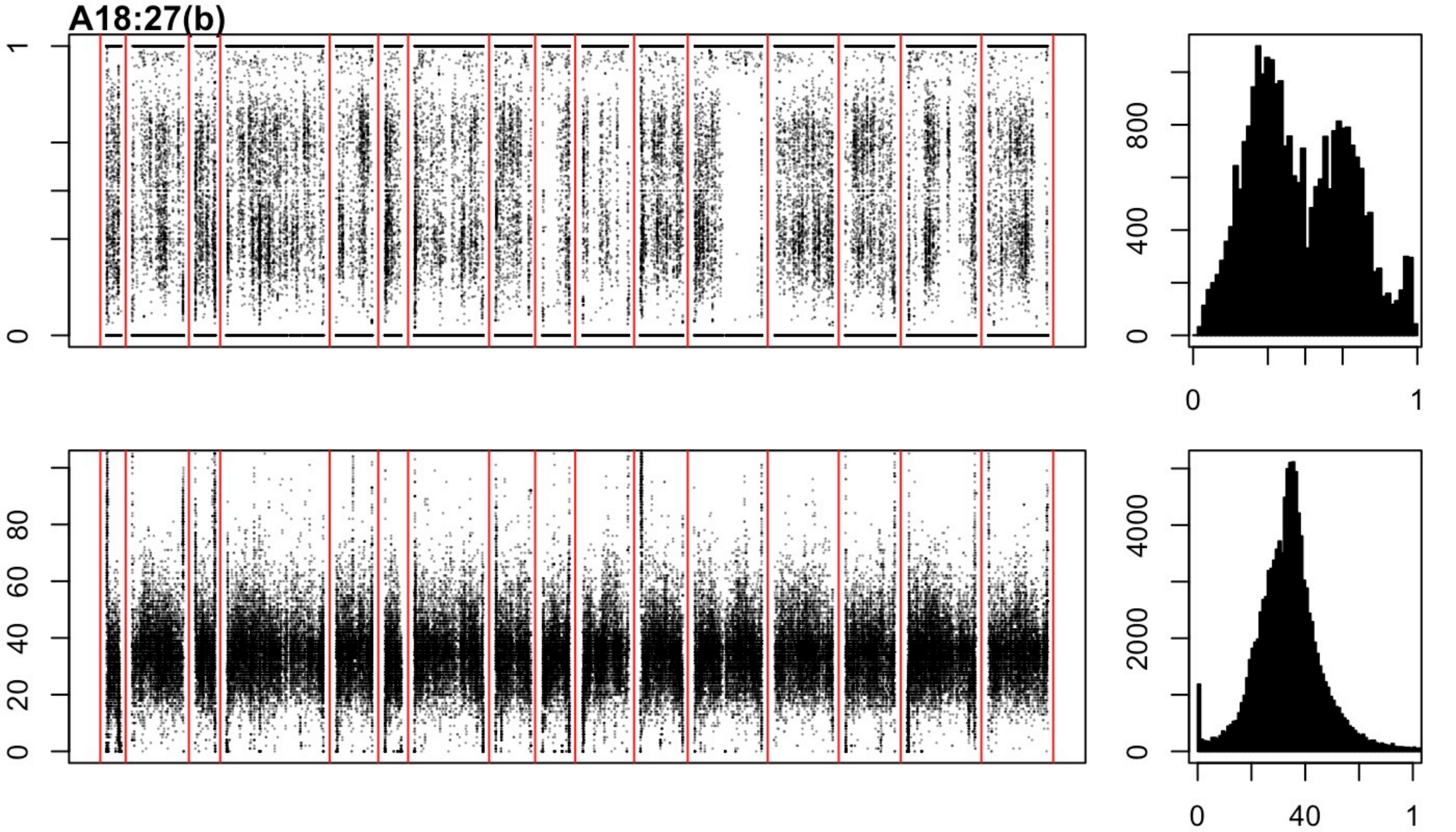
A18:27(b)
1
0
0
400
800
0
1
0
20
40
60
80
0
2000
4000
0
40
1

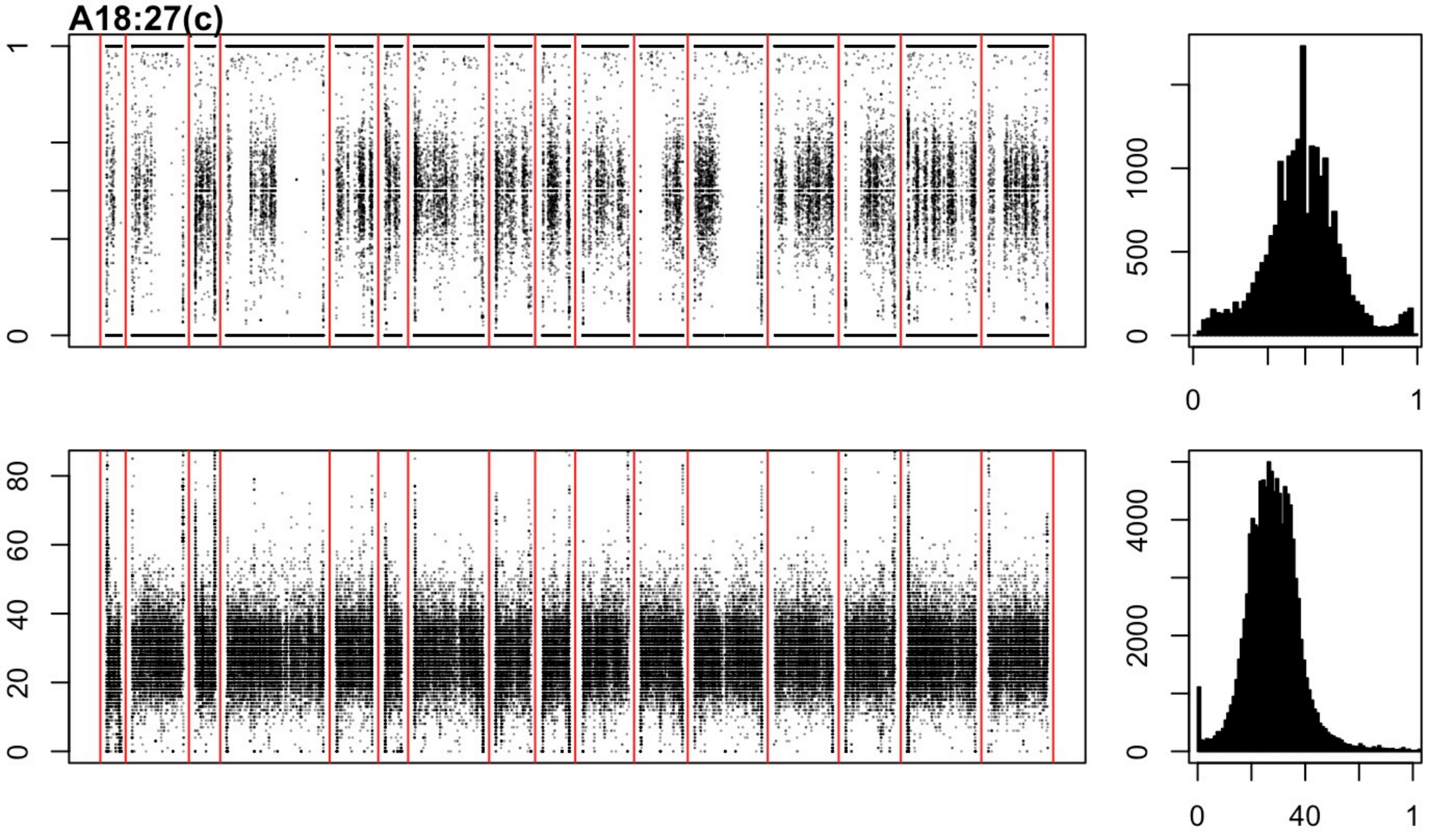
A18:27(c)
1
0
1000
500
0
0
1
80
60
40
20
0
4000
2000
0
0
40
1

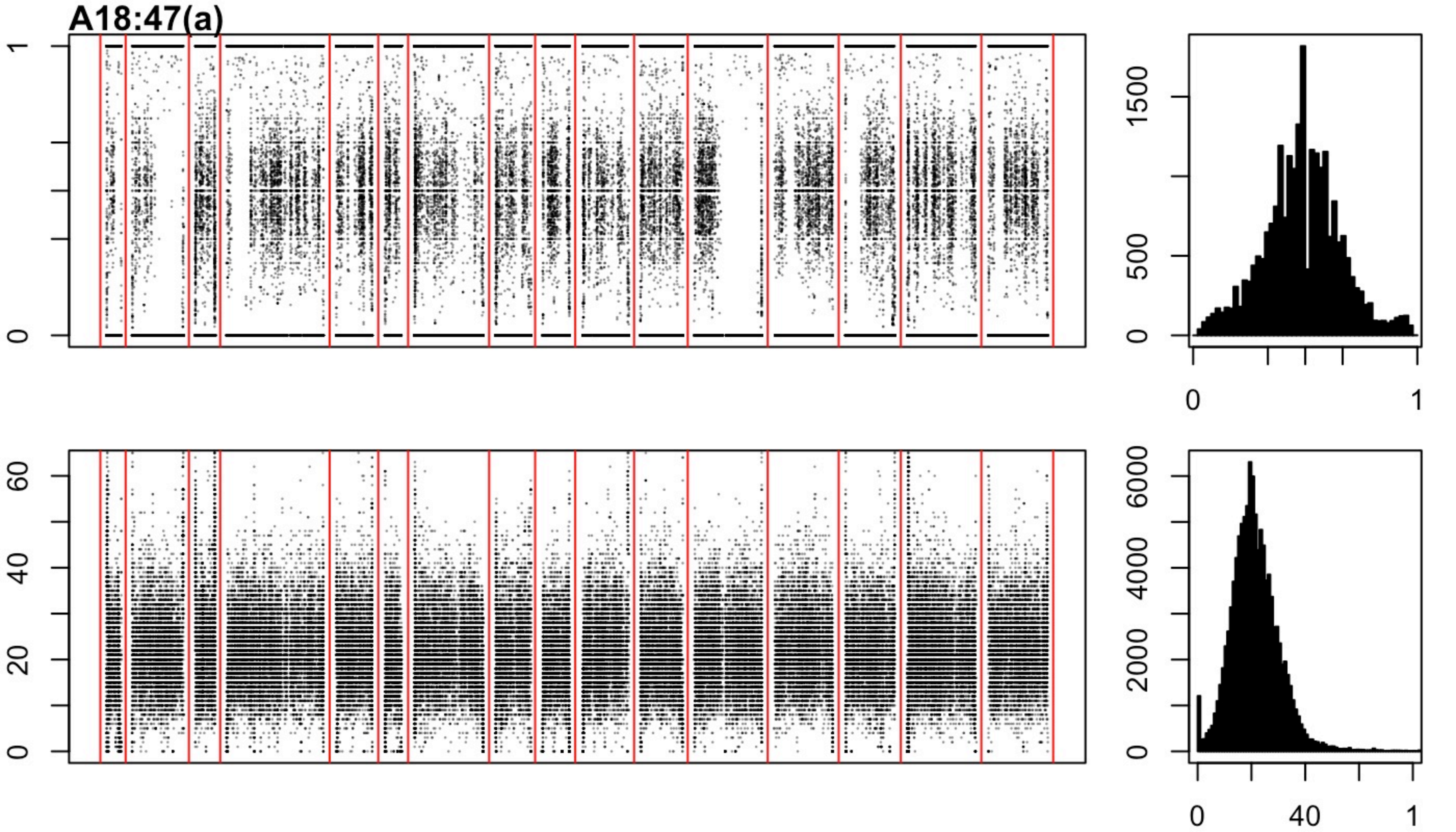
A18:47(a)
1
0
1500
500
0
0
1
60
40
20
0
6000
4000
2000
0
0
40
1

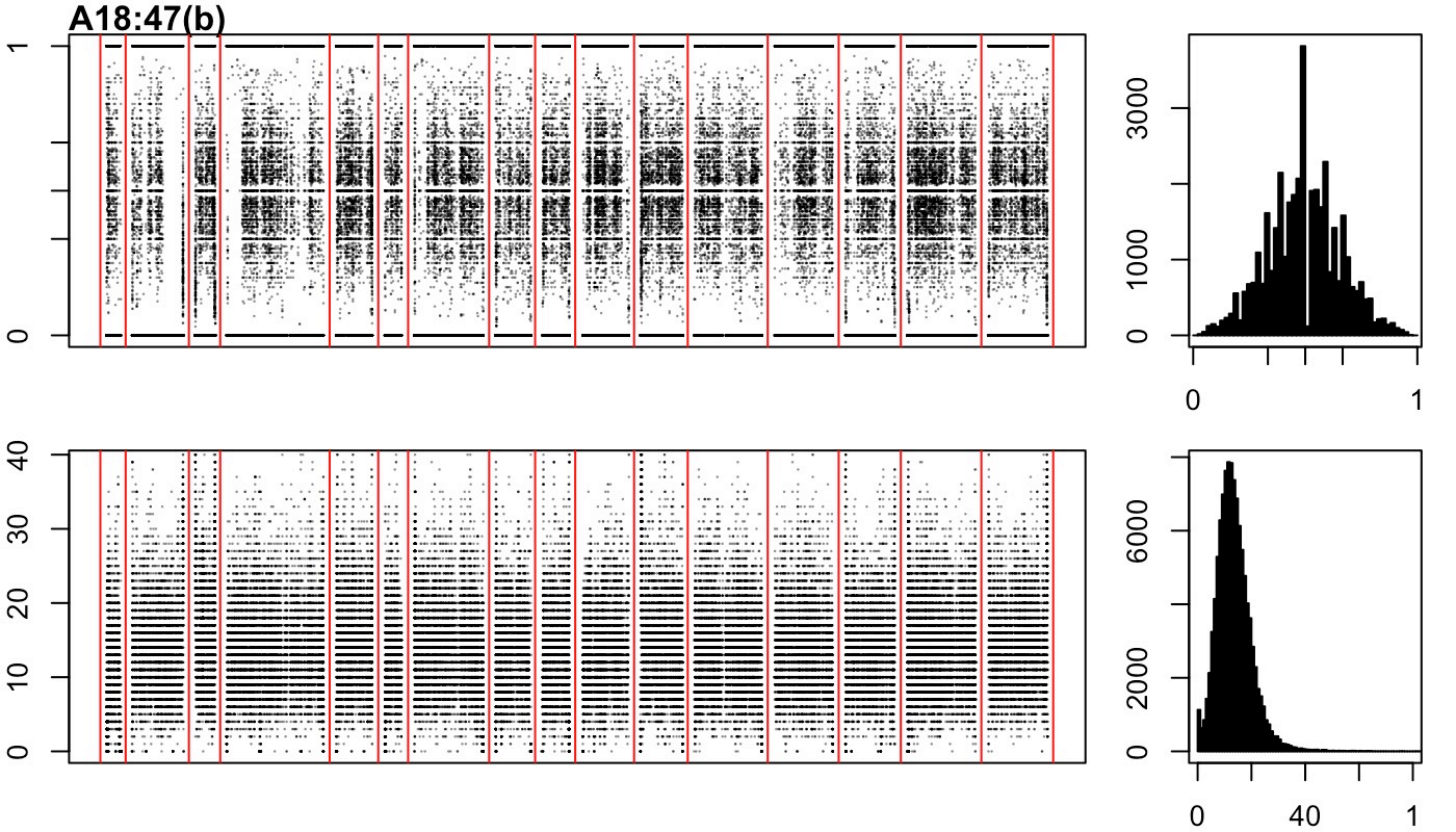
A18:47(b)
1
0
3000
1000
0
0
1
40
30
20
10
0
6000
2000
0
0
40
1

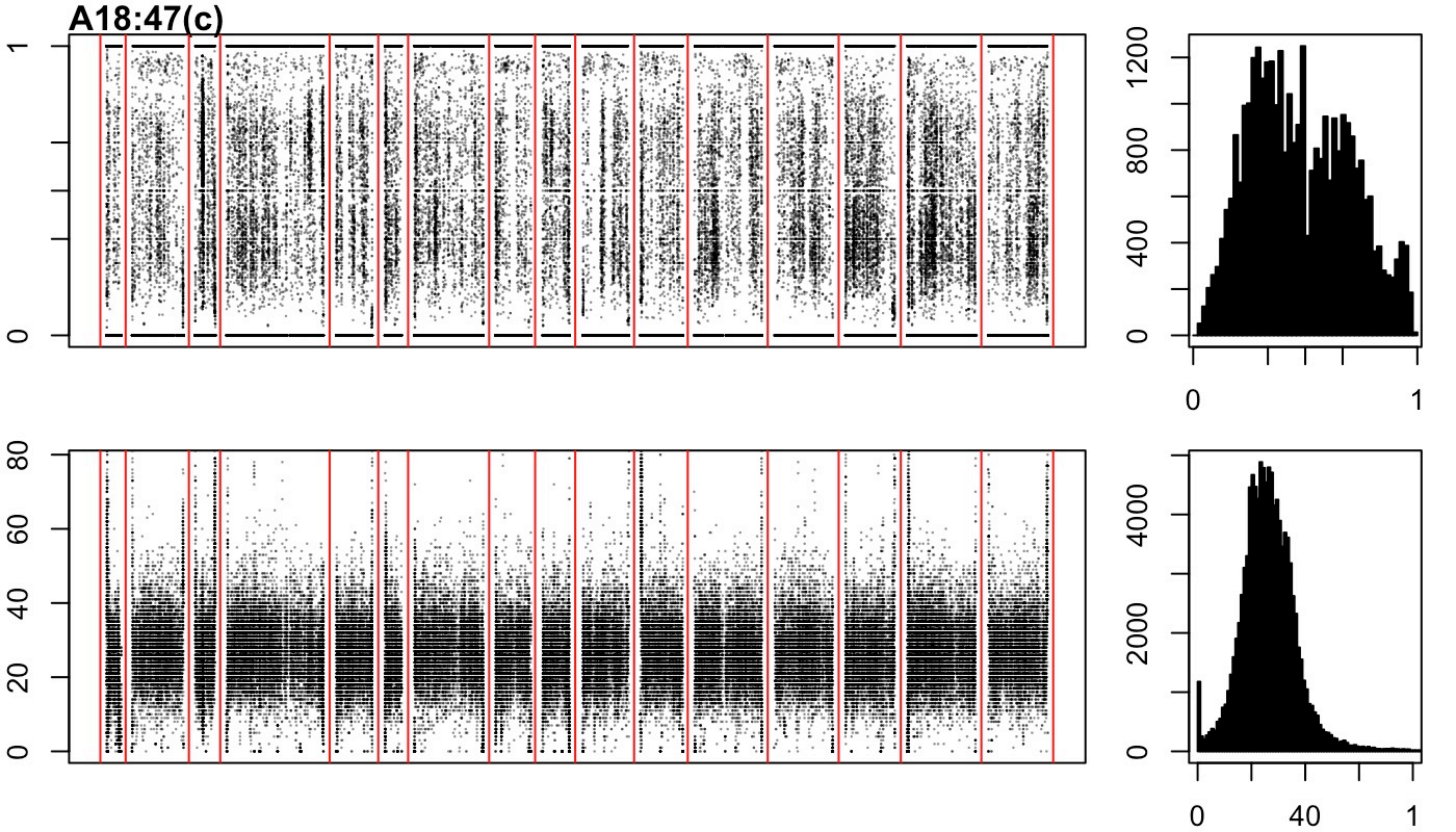

A18:47(c)
1
0
1200
800
400
0
0
1
80
60
40
20
0
4000
2000
0
0
40
1

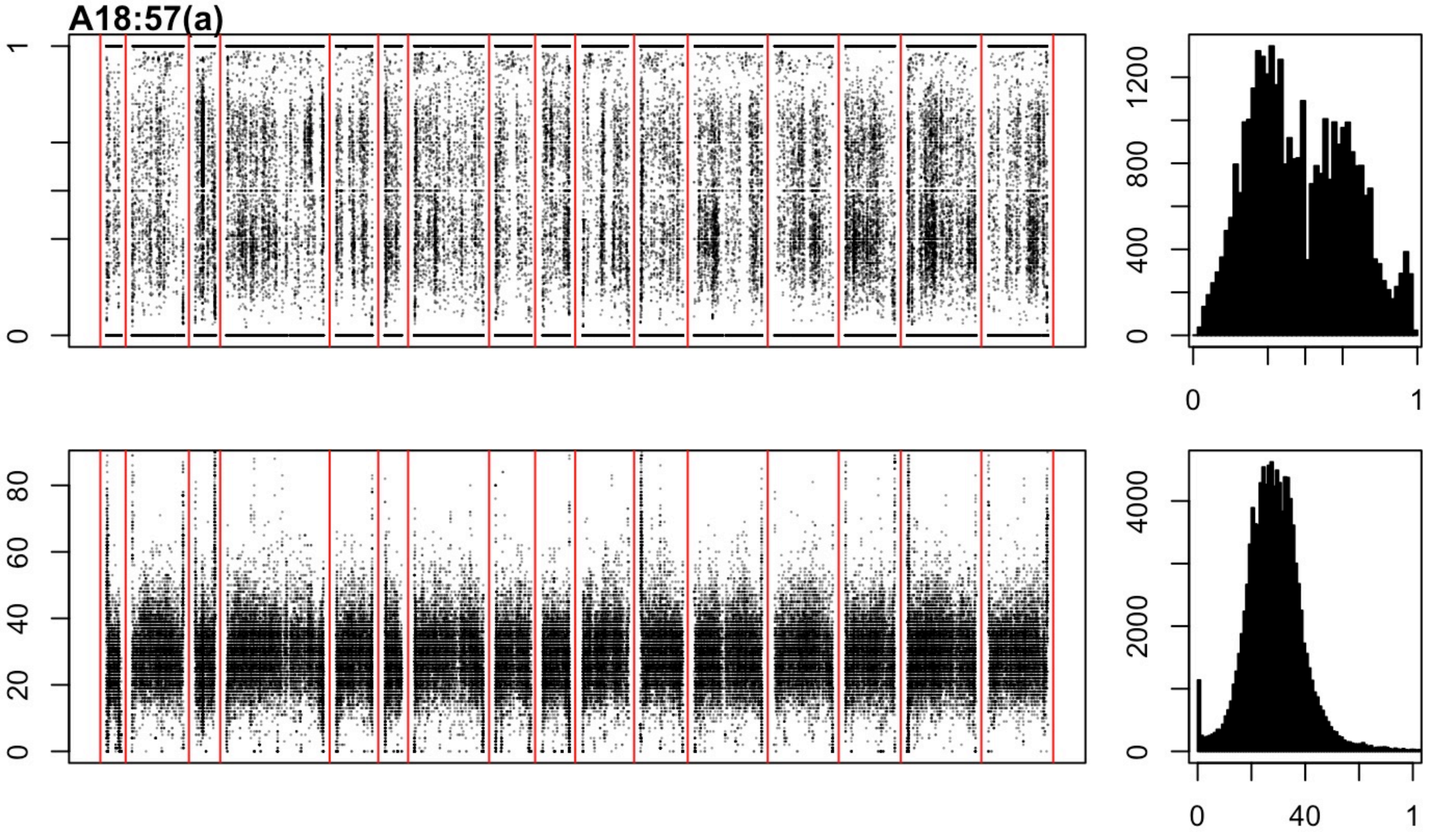

A18:57(a)
1
0
1200
800
400
0
0
1
80
60
40
20
0
4000
2000
0
0
40
1

A18:57(b)

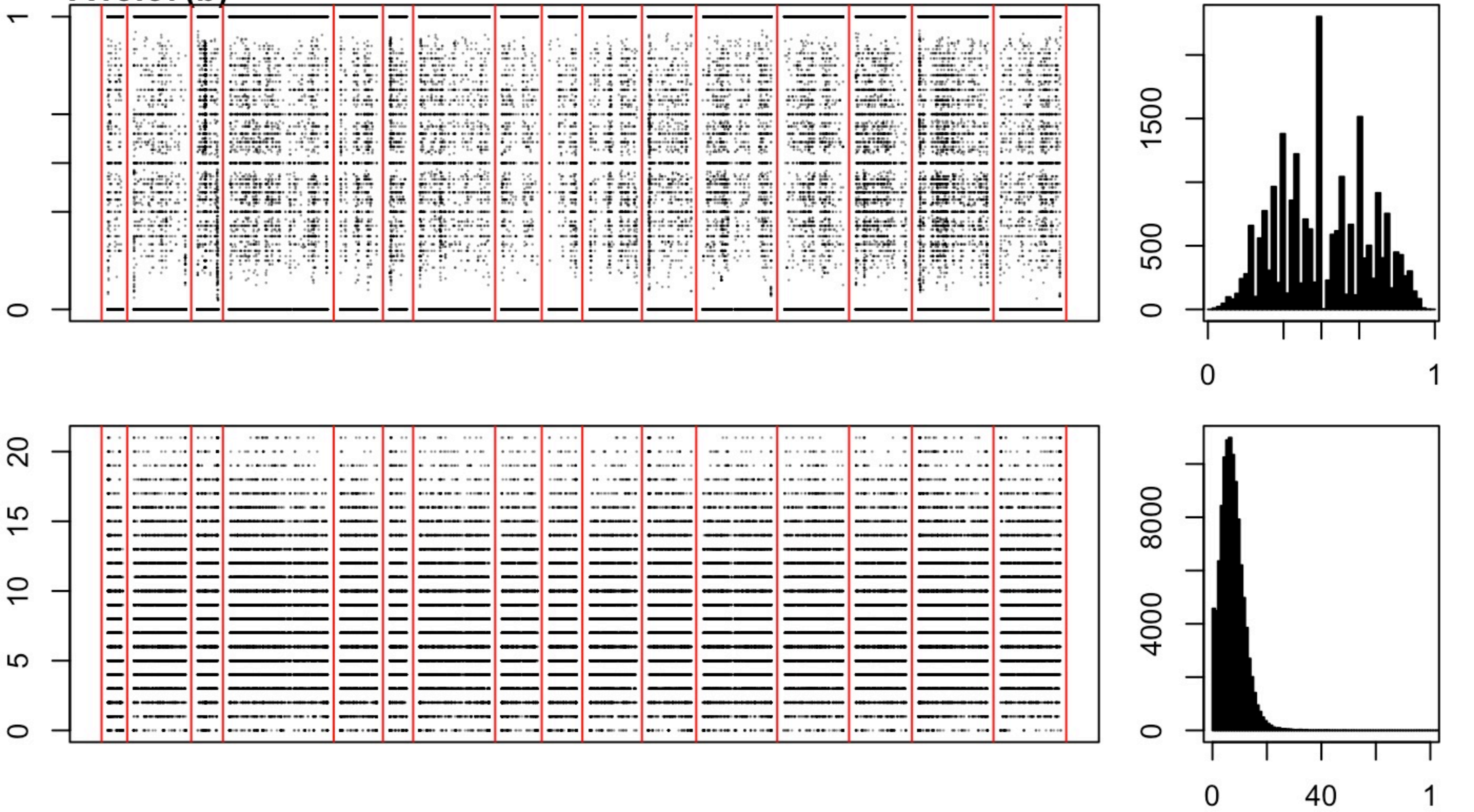

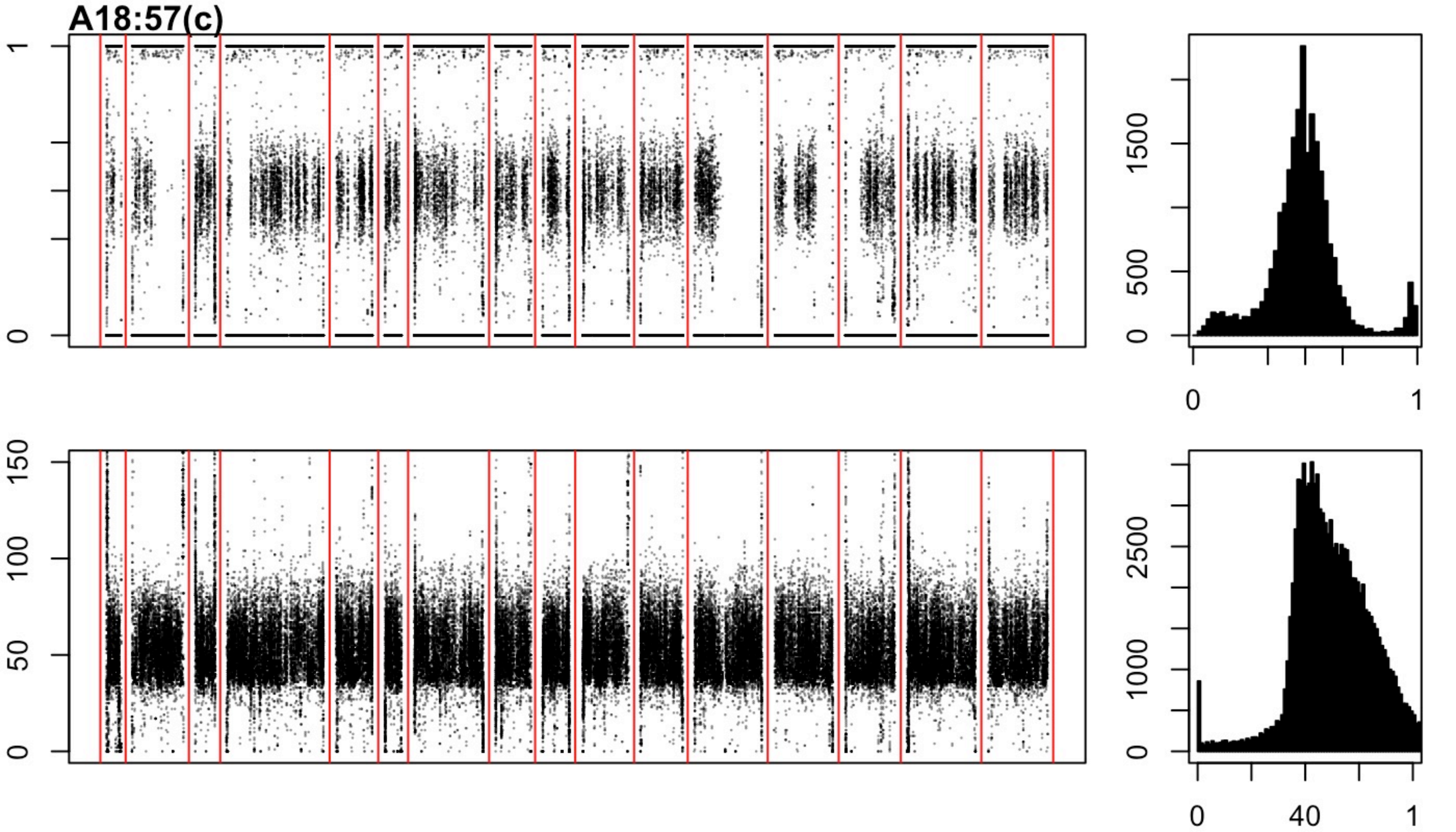

A18:57(c)
1
0
1500
500
0
0
1
150
100
50
0
2500
1000
0
0
40
1

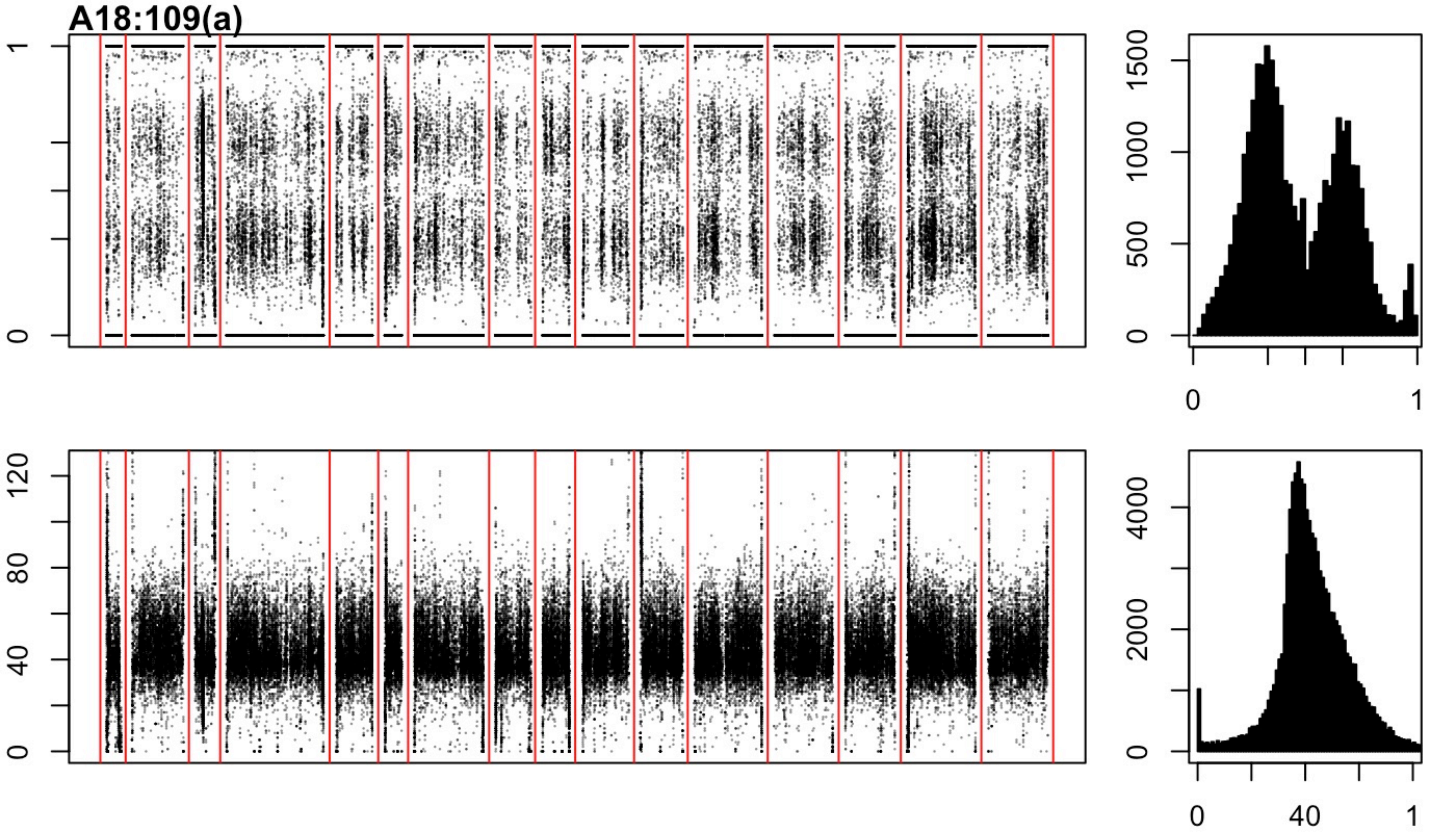
A18:109(a)
1
0
1500
1000
500
0
0
1
120
80
40
0
4000
2000
0
0
40
1

A18:109(b)

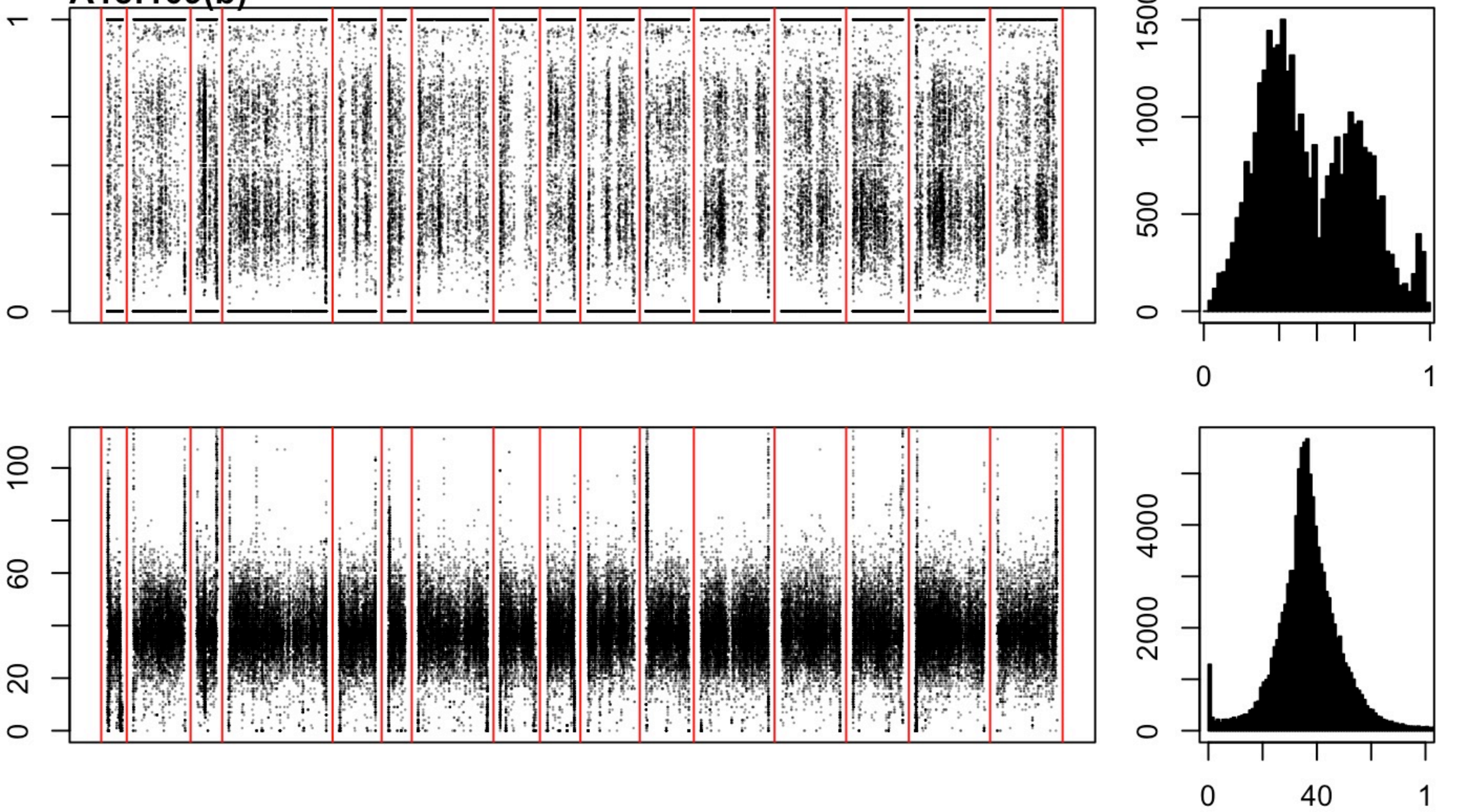

A18:109(c)

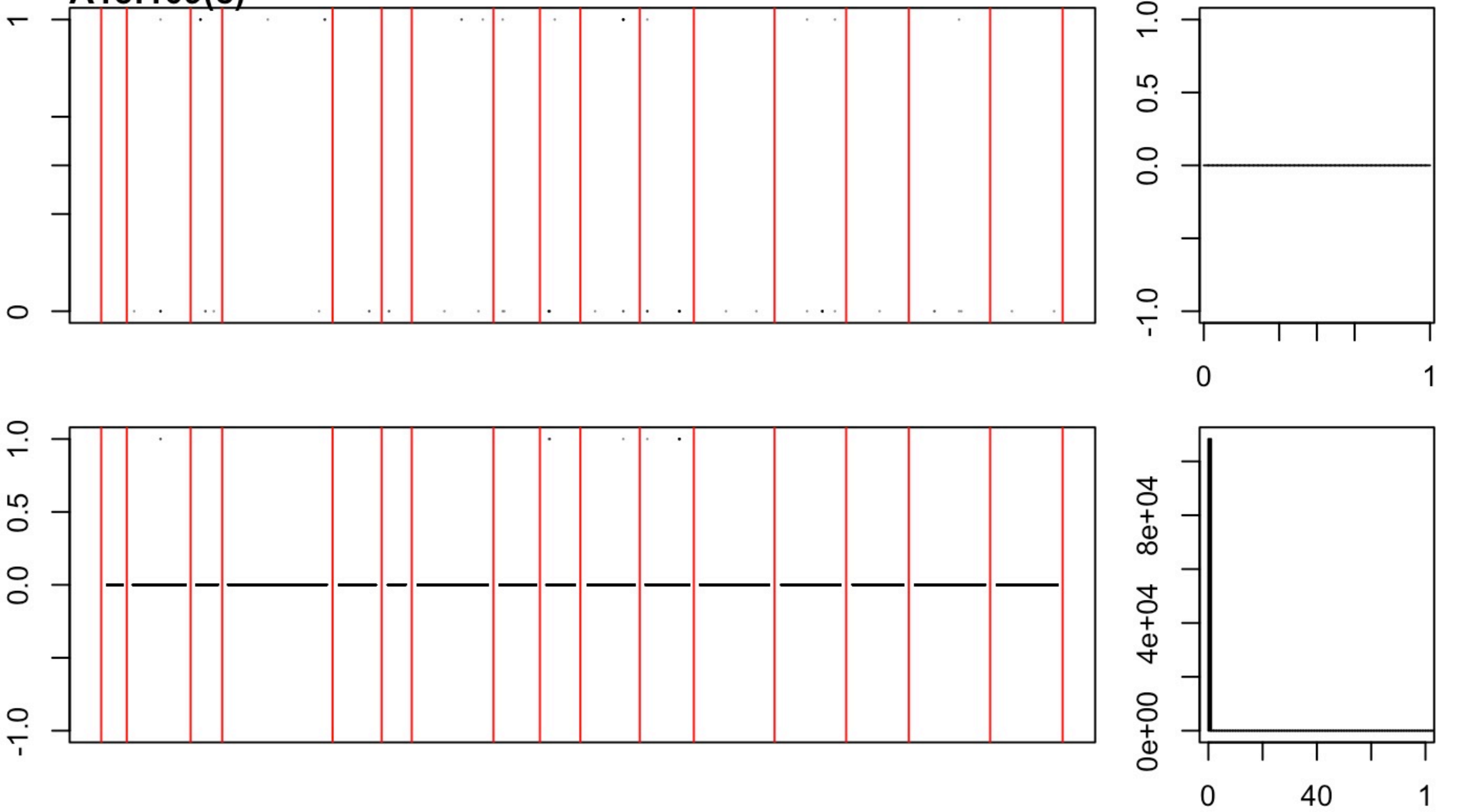

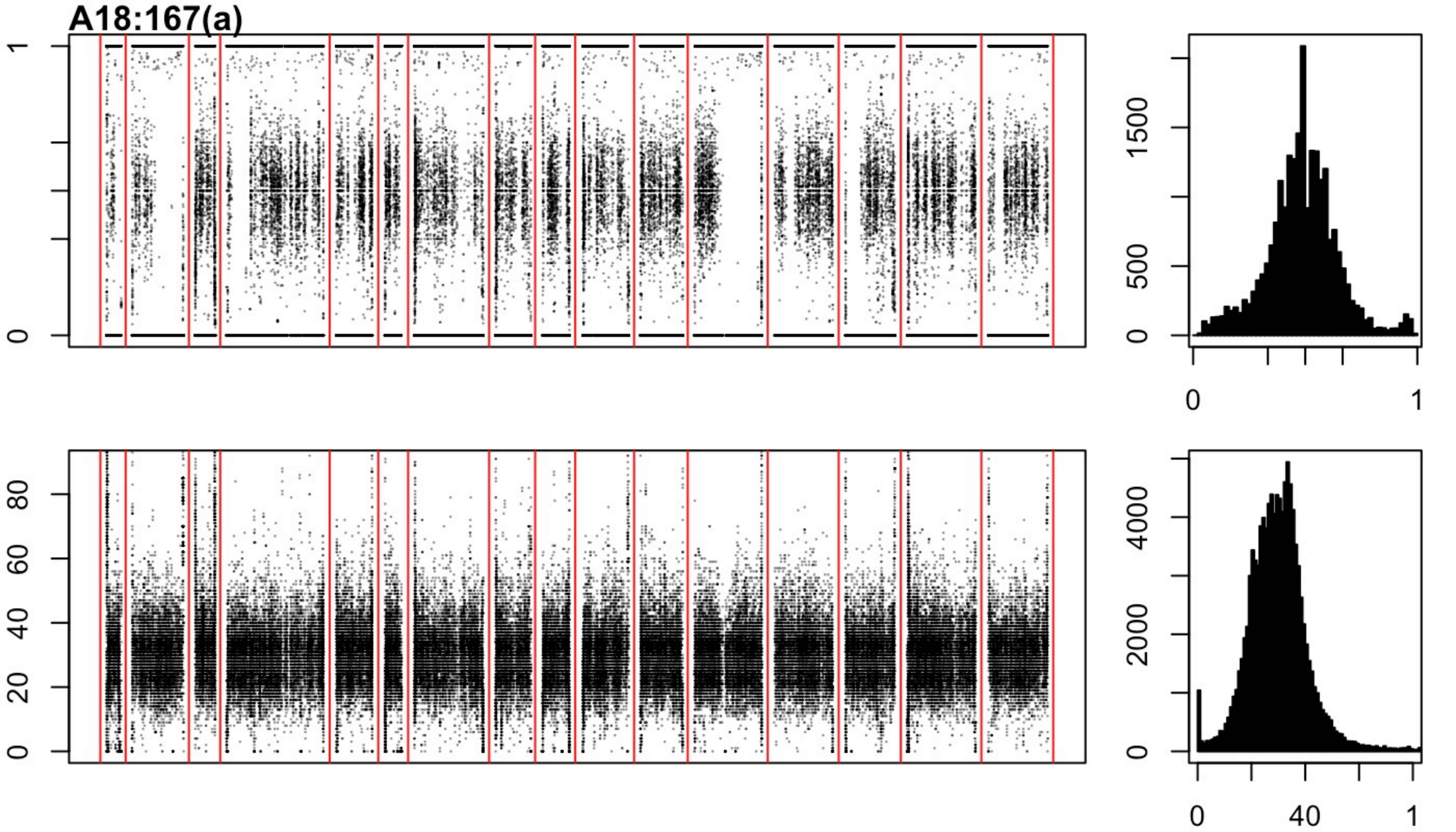
A18:167(a)
1
0
1500
500
0
0
1
80
60
40
20
0
4000
2000
0
0
40
1

A18:167(b)

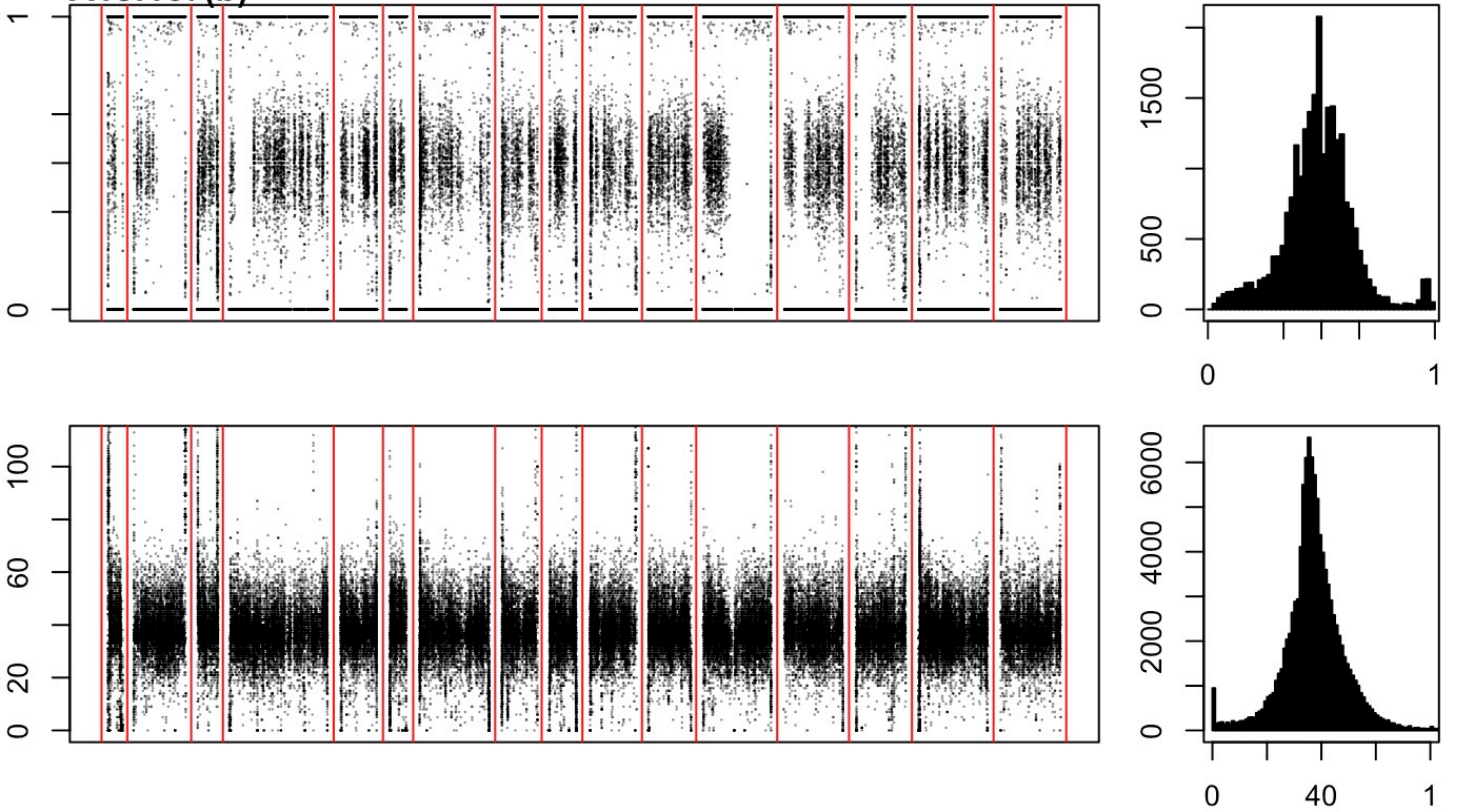

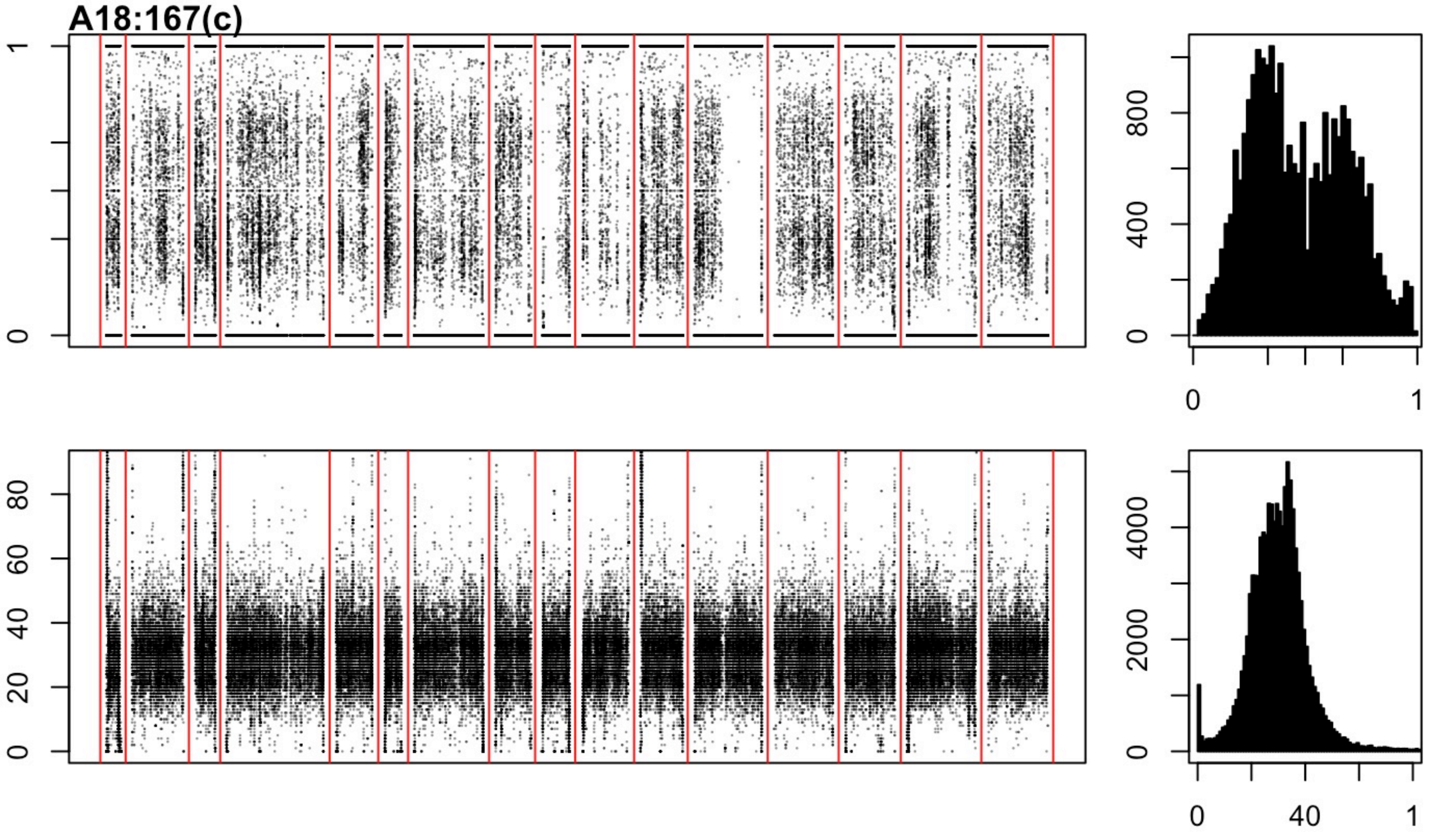
A18:167(c)
0
1
0
400
800
0
1
0
20
40
60
80
0
2000
4000
0
40
1

A18:217(c)

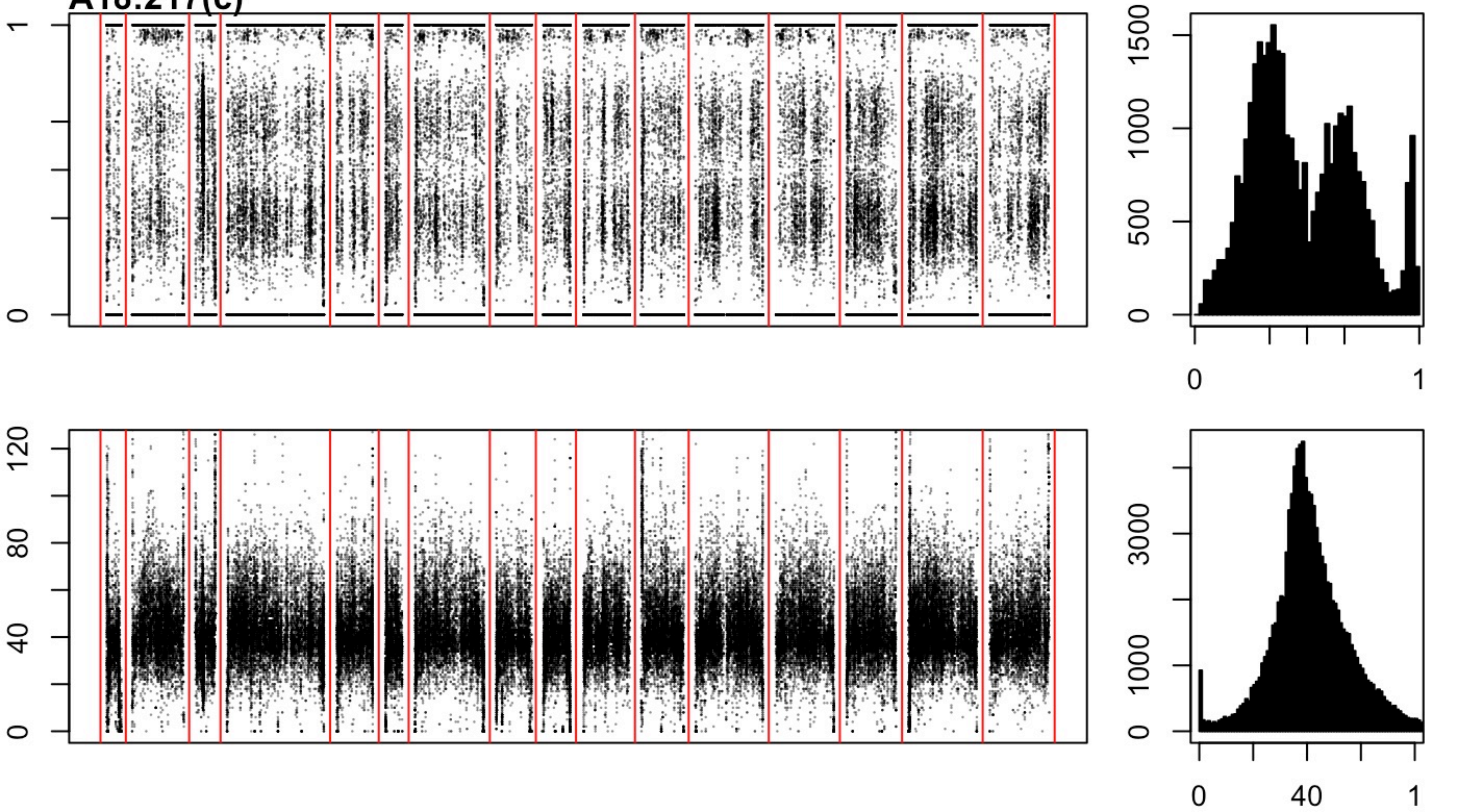

A18:217(d)

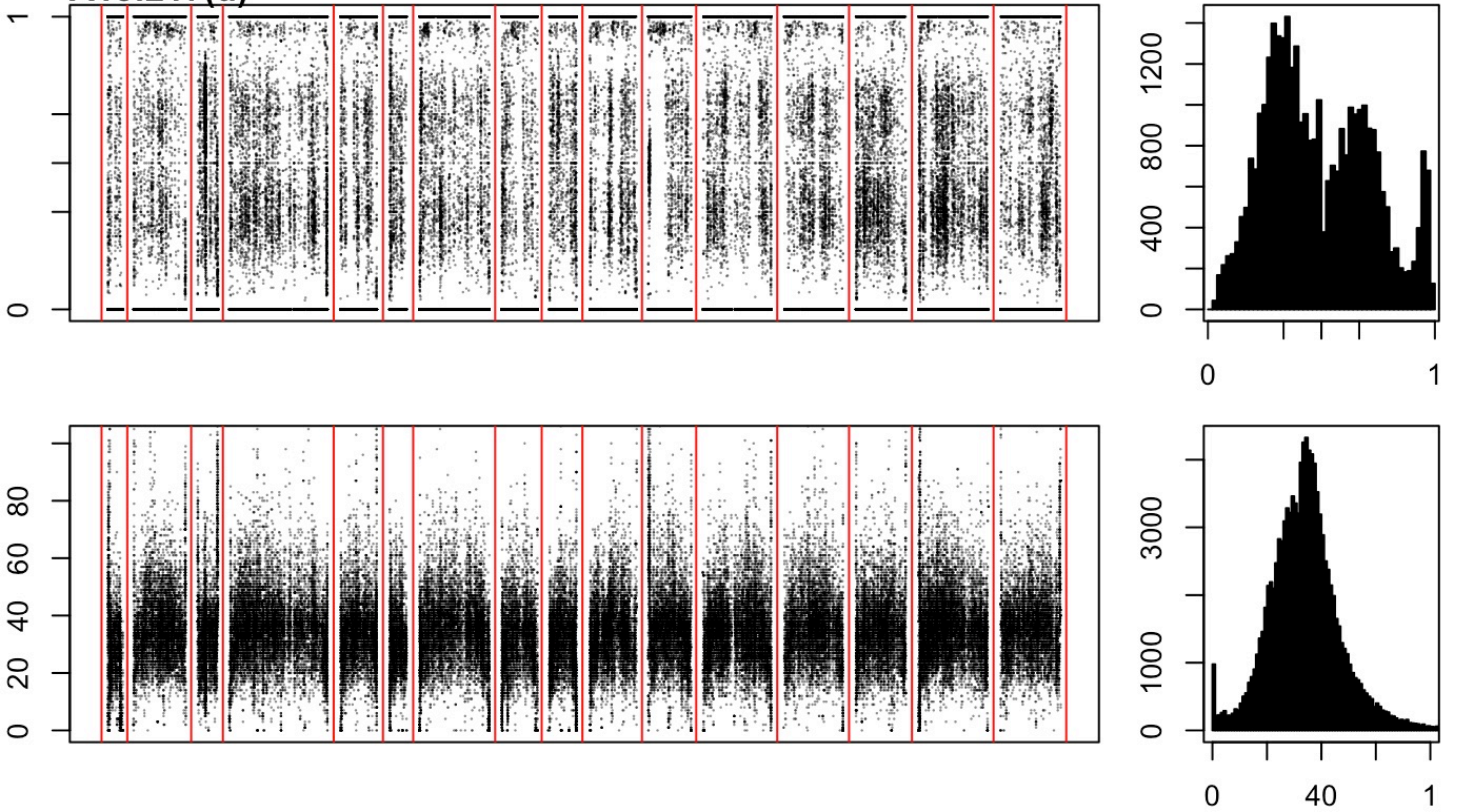

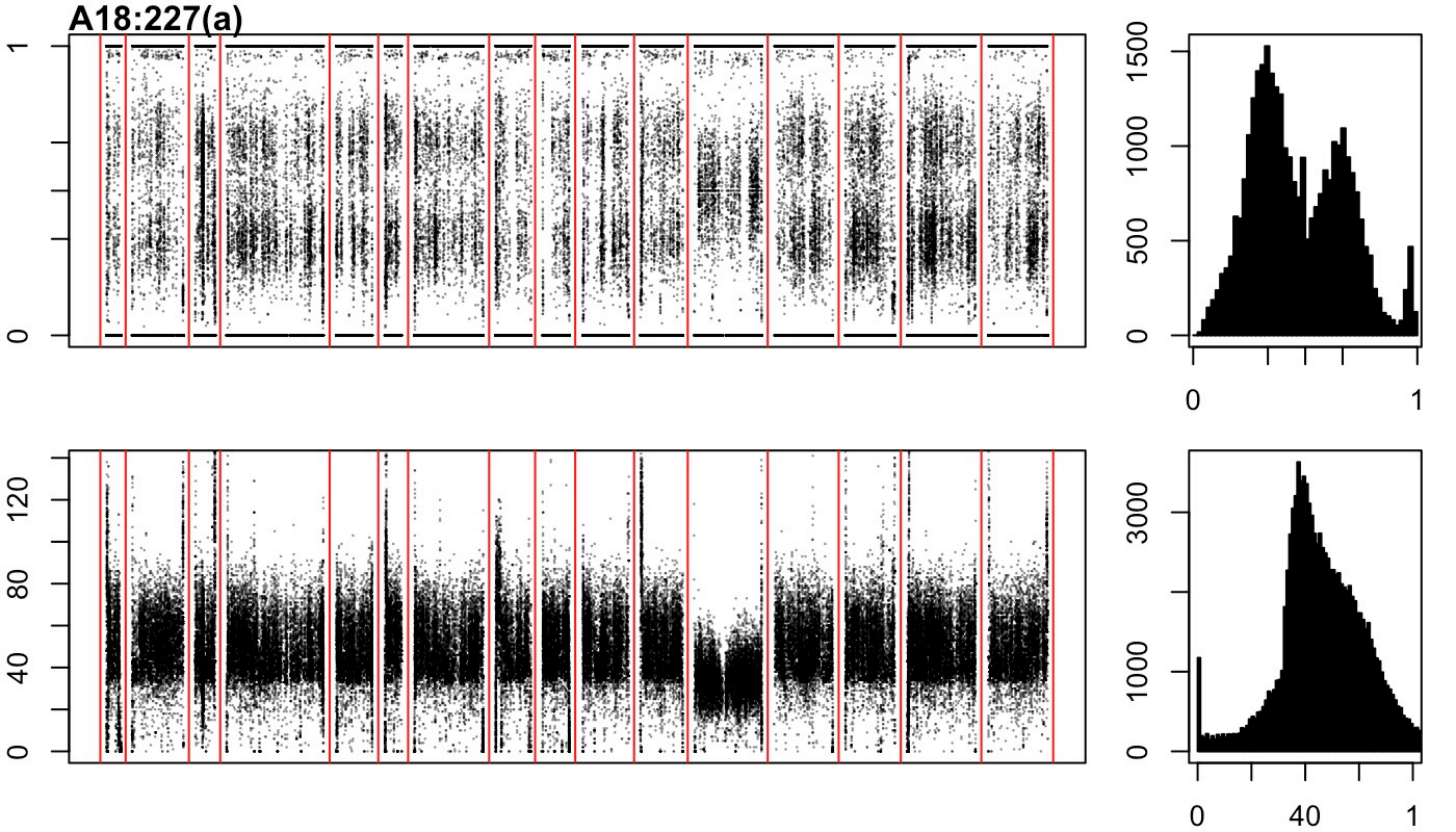

A18:227(a)
1
0
1500
1000
500
0
0
1
120
80
40
0
3000
1000
0
0
40
1

A18:227(b)

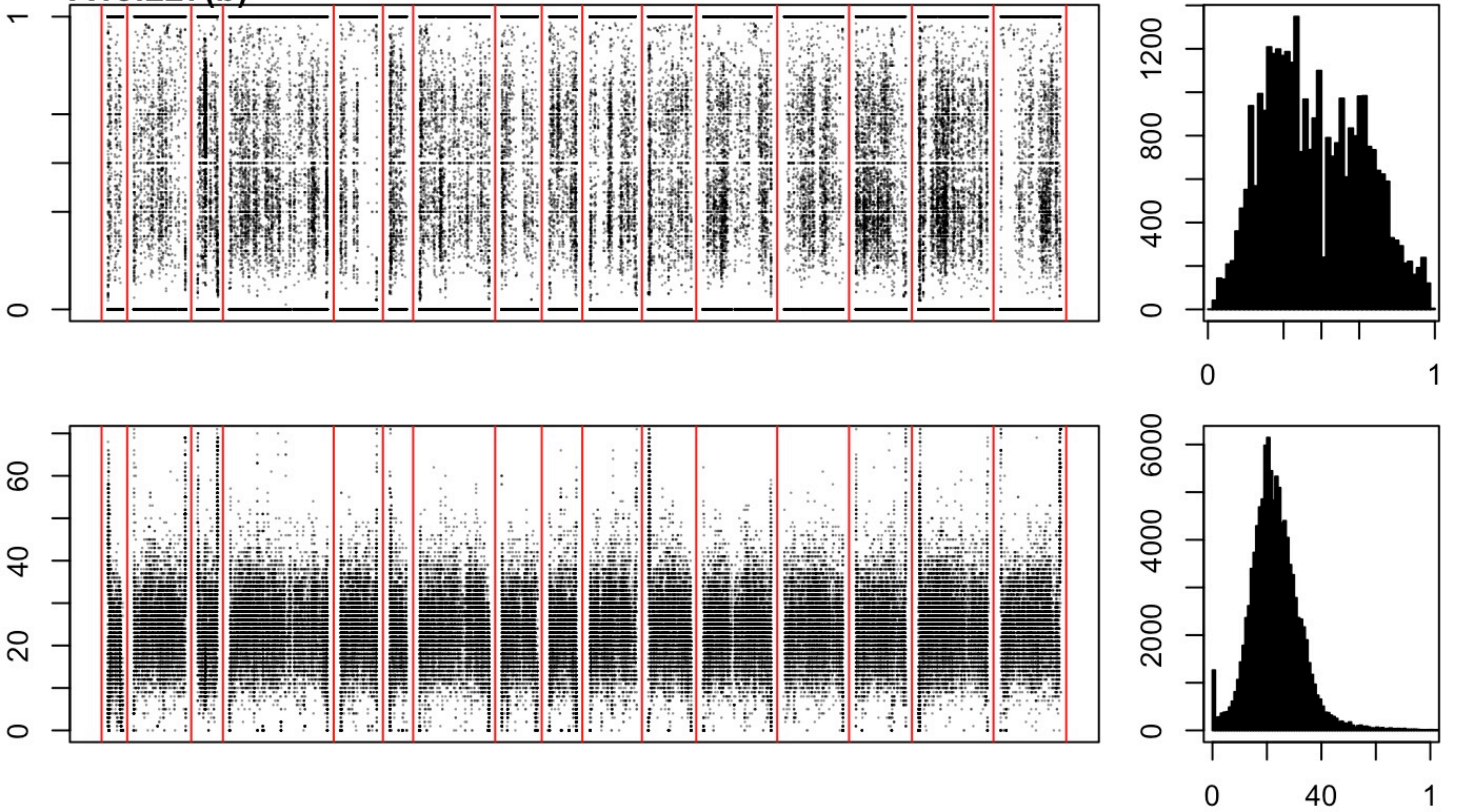

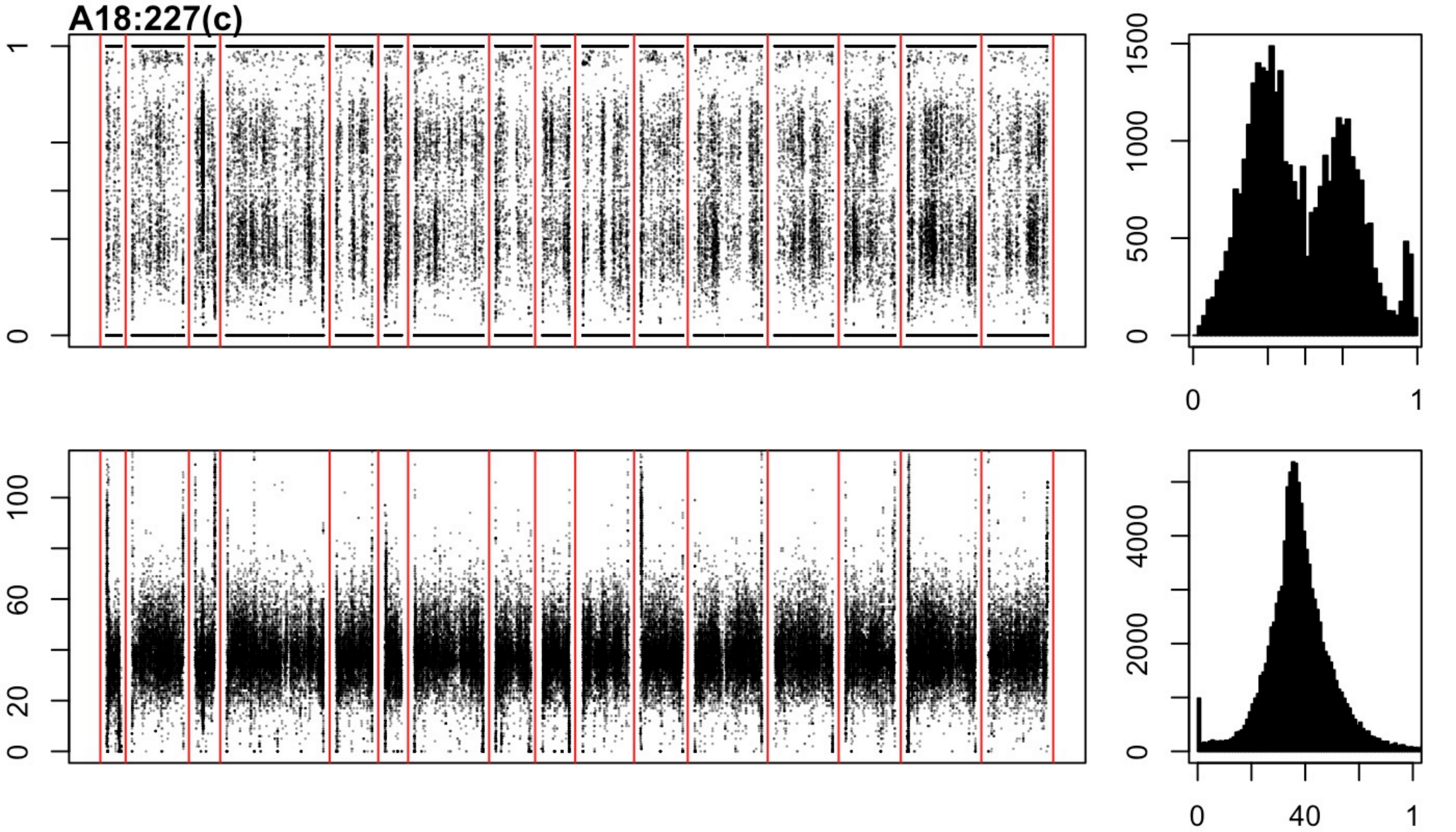
A18:227(c)
1
0
1500
1000
500
0
0
1
100
60
20
0
4000
2000
0
0
40
1

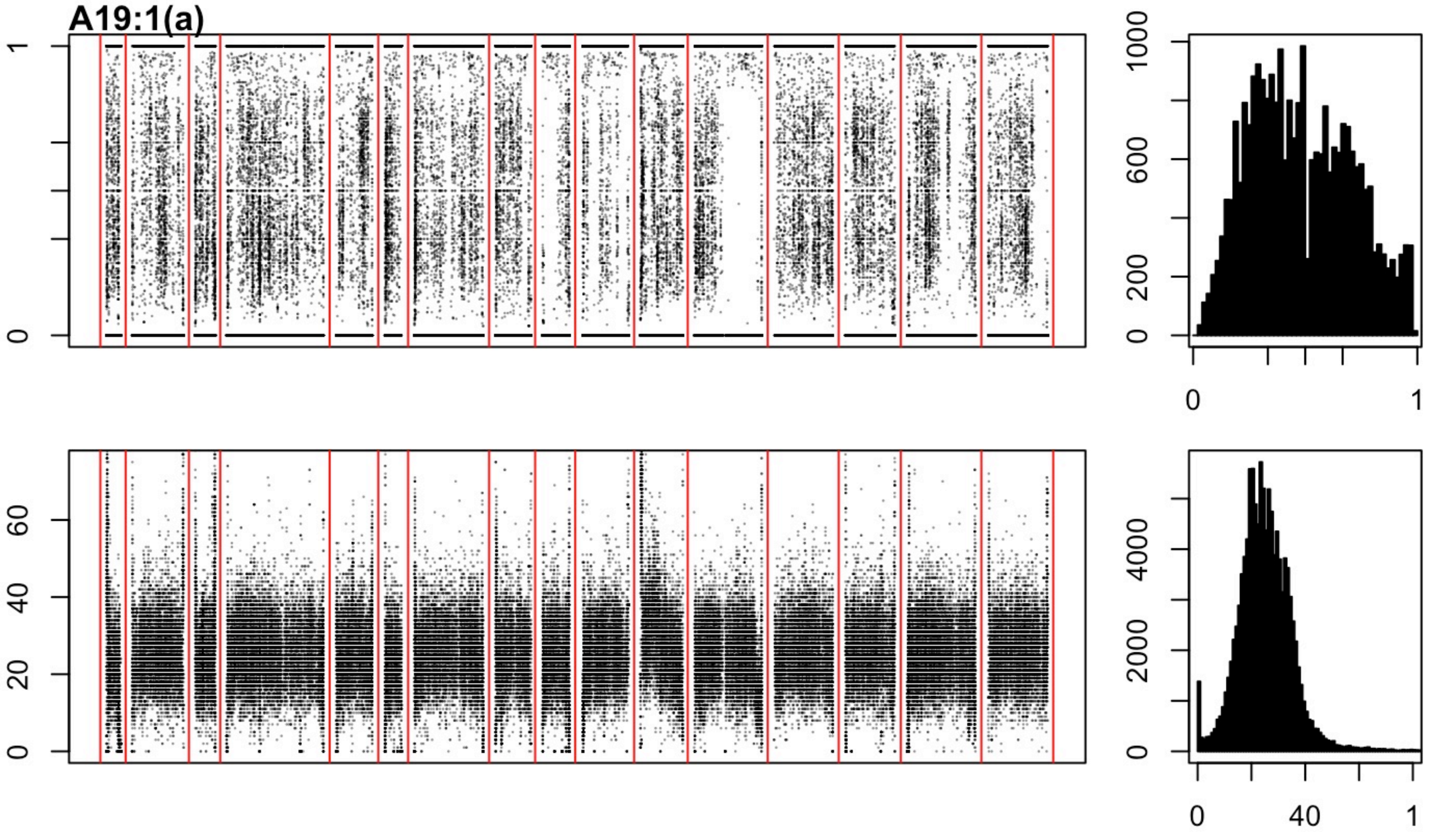

A19:1(a)
1
0
1000
600
200
0
0
1
60
40
20
0
4000
2000
0
0
40
1

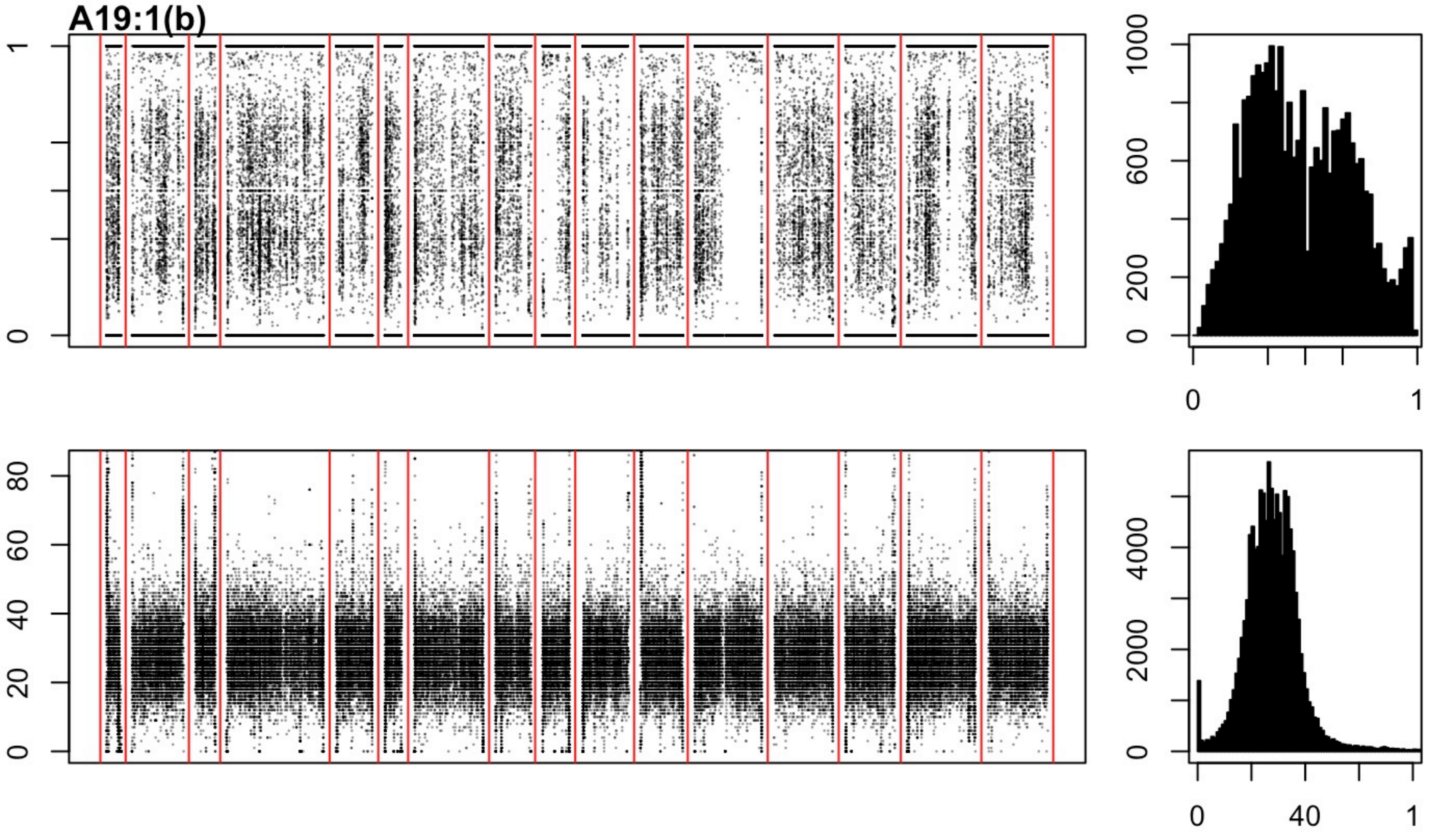
A19:1(b)
1
0
1000
600
200
0
0
1
80
60
40
20
0
4000
2000
0
0
40
1

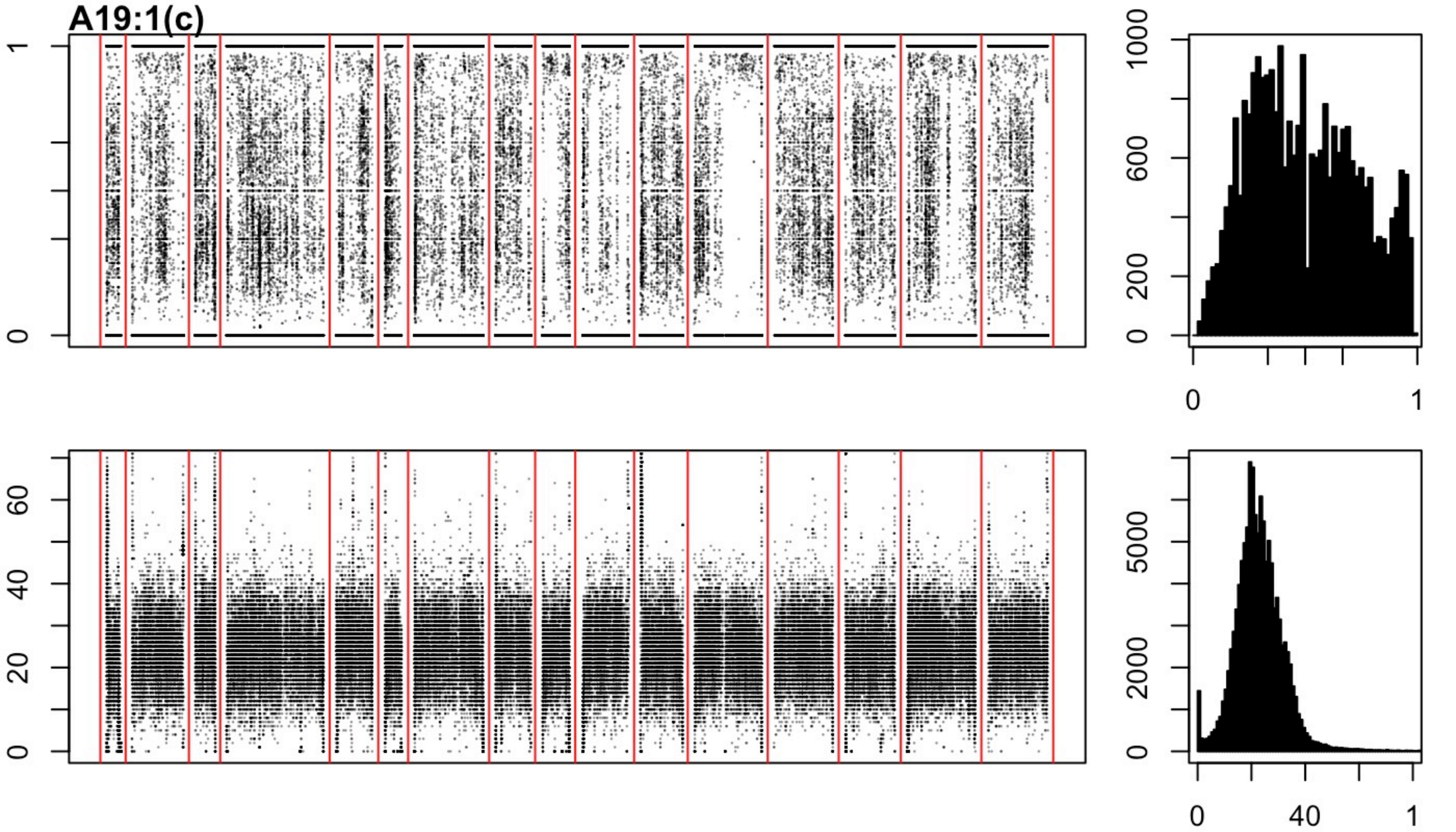
A19:1(c)
1
0
1000
600
200
0
0
1
60
40
20
0
5000
2000
0
0
40
1

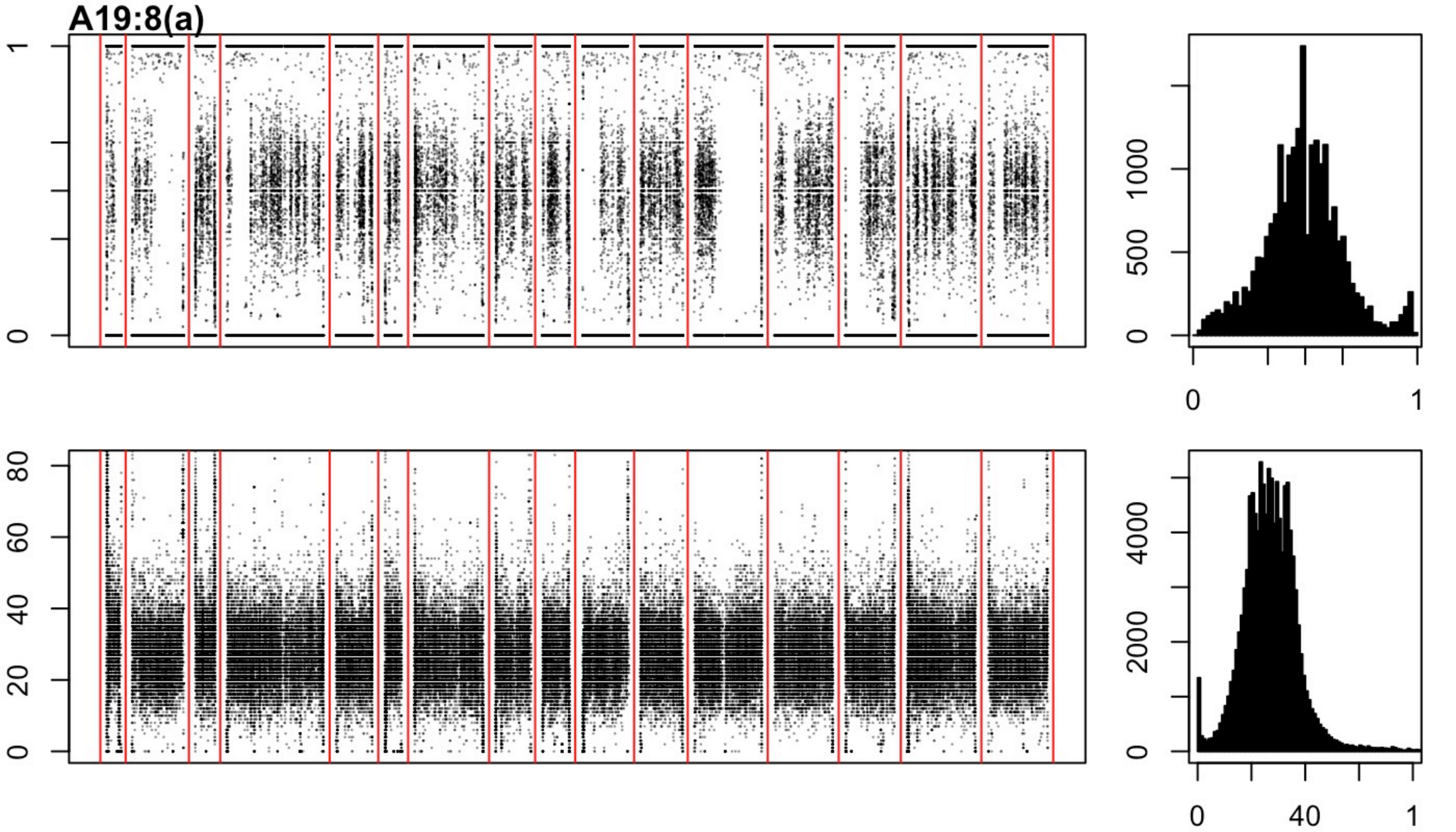

A19:8(a)
1
0
1000
500
0
0
1
80
60
40
20
0
4000
2000
0
0
40
1

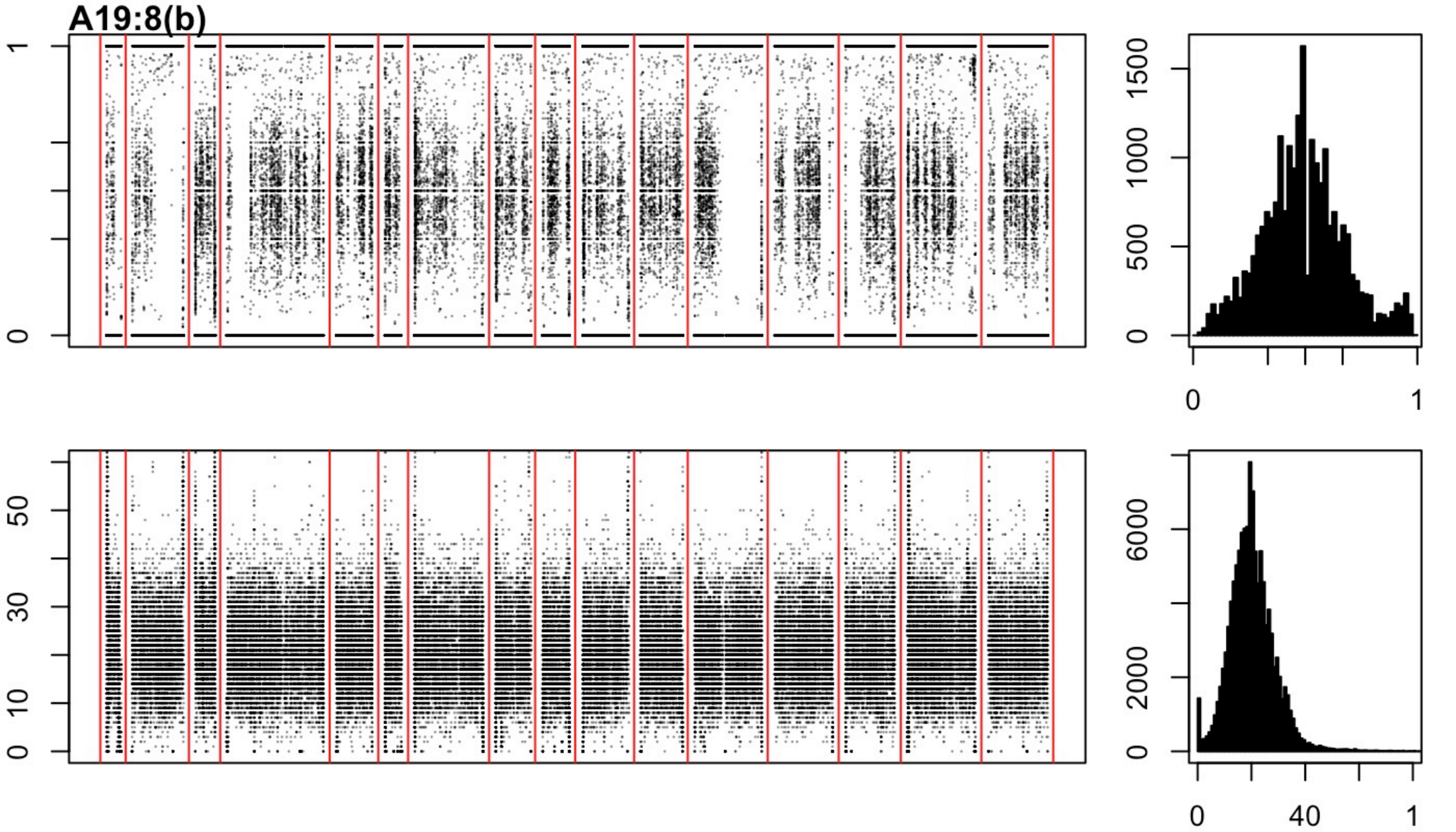

A19:8(b)
0
1
0
500
1000
1500
0
1
0
10
30
50
0
2000
6000
0
40
1

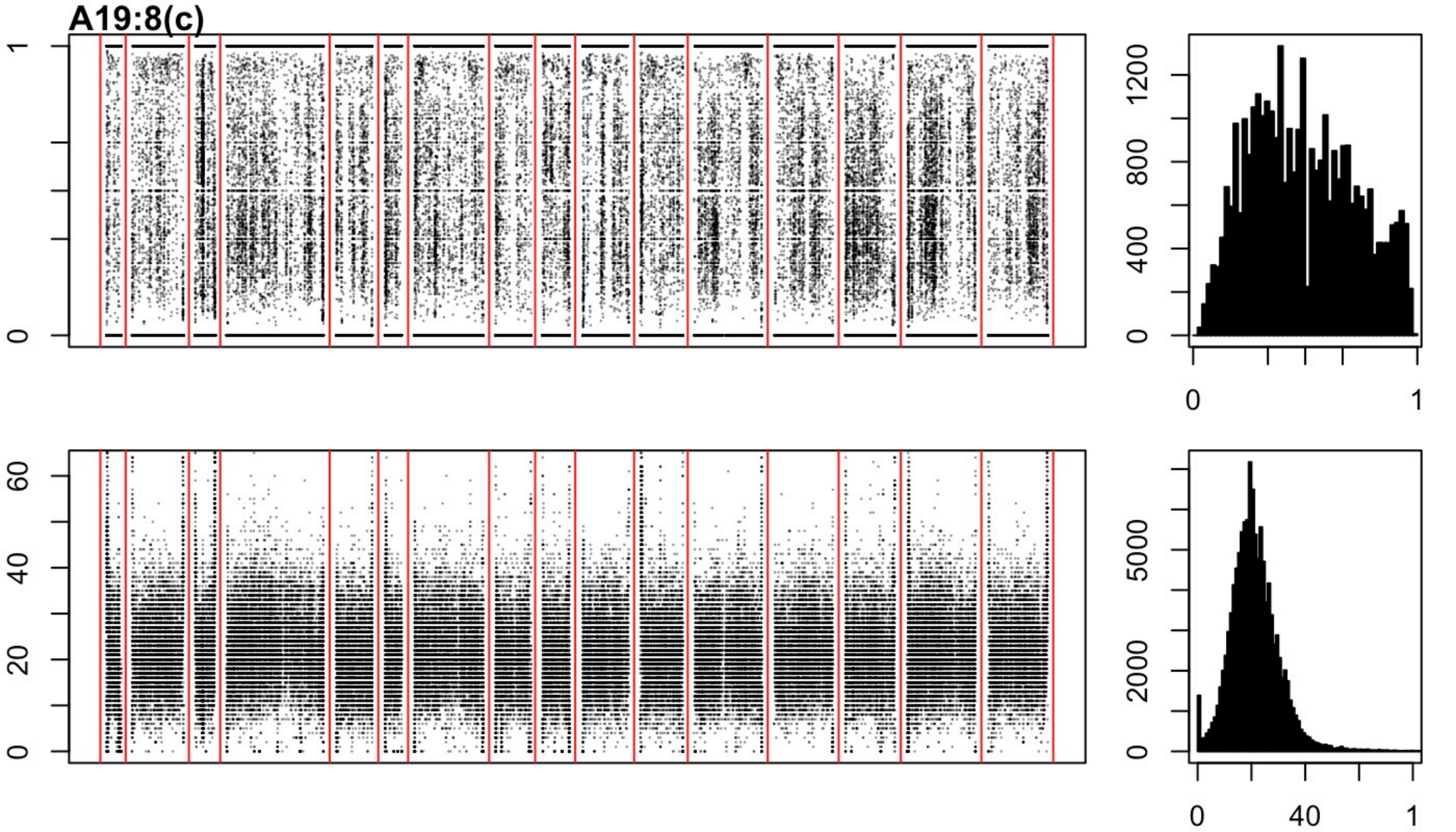
A19:8(c)
0
1
1200
800
400
0
0
1
60
40
20
0
5000
2000
0
0
40
1

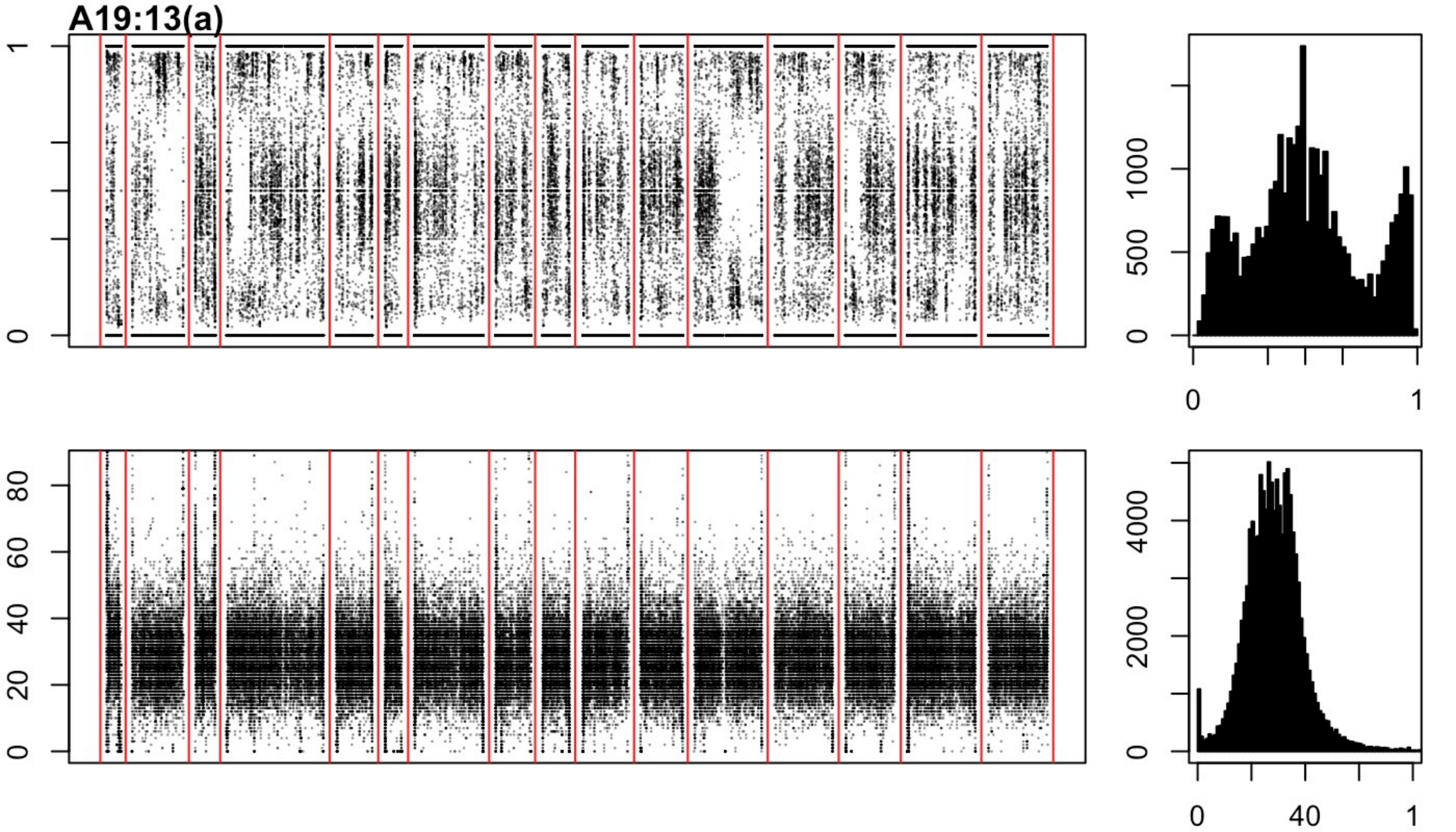

A19:13(a)
1
0
0
500
1000
0
1
80
60
40
20
0
4000
2000
0
0
40
1

A19:13(b)

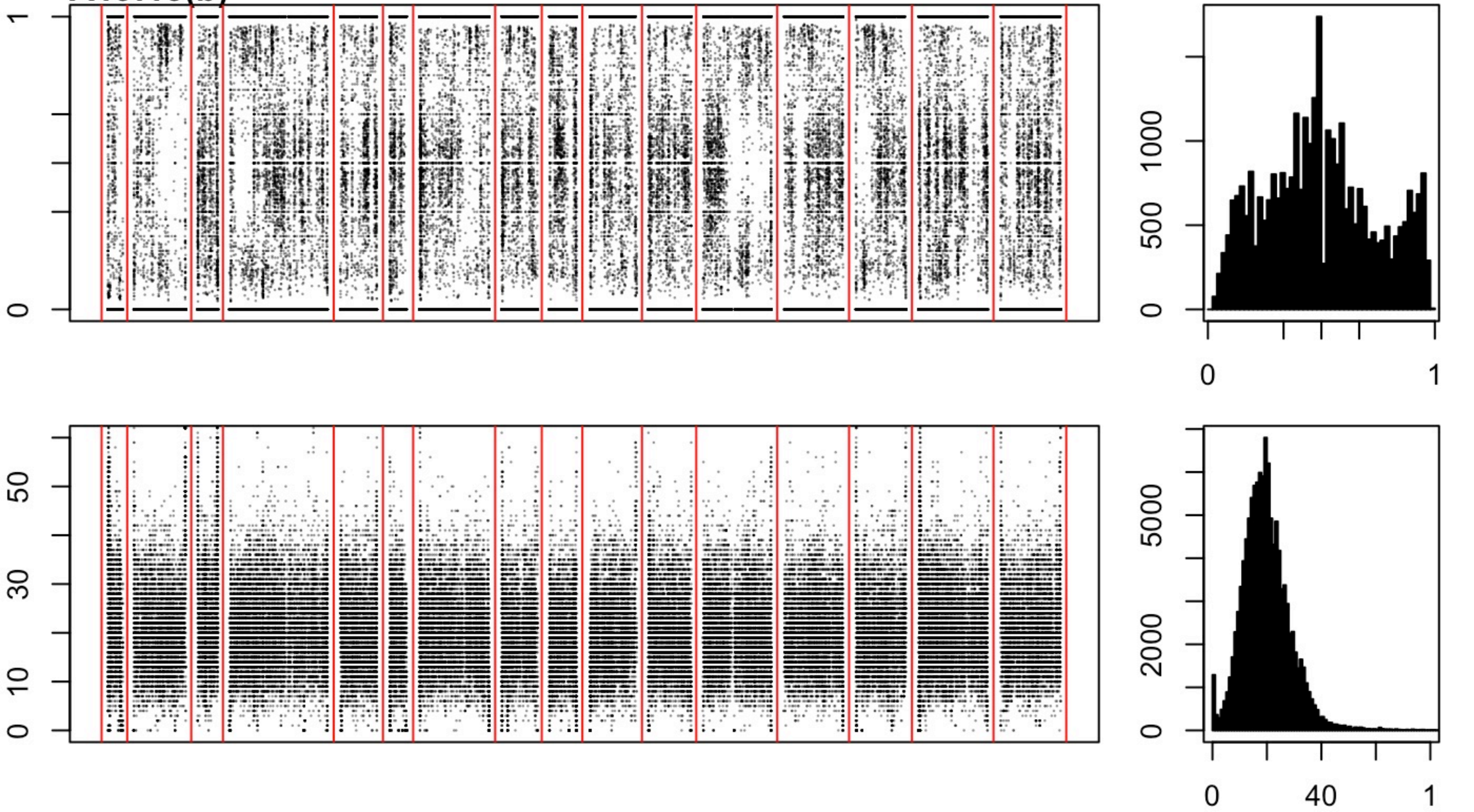

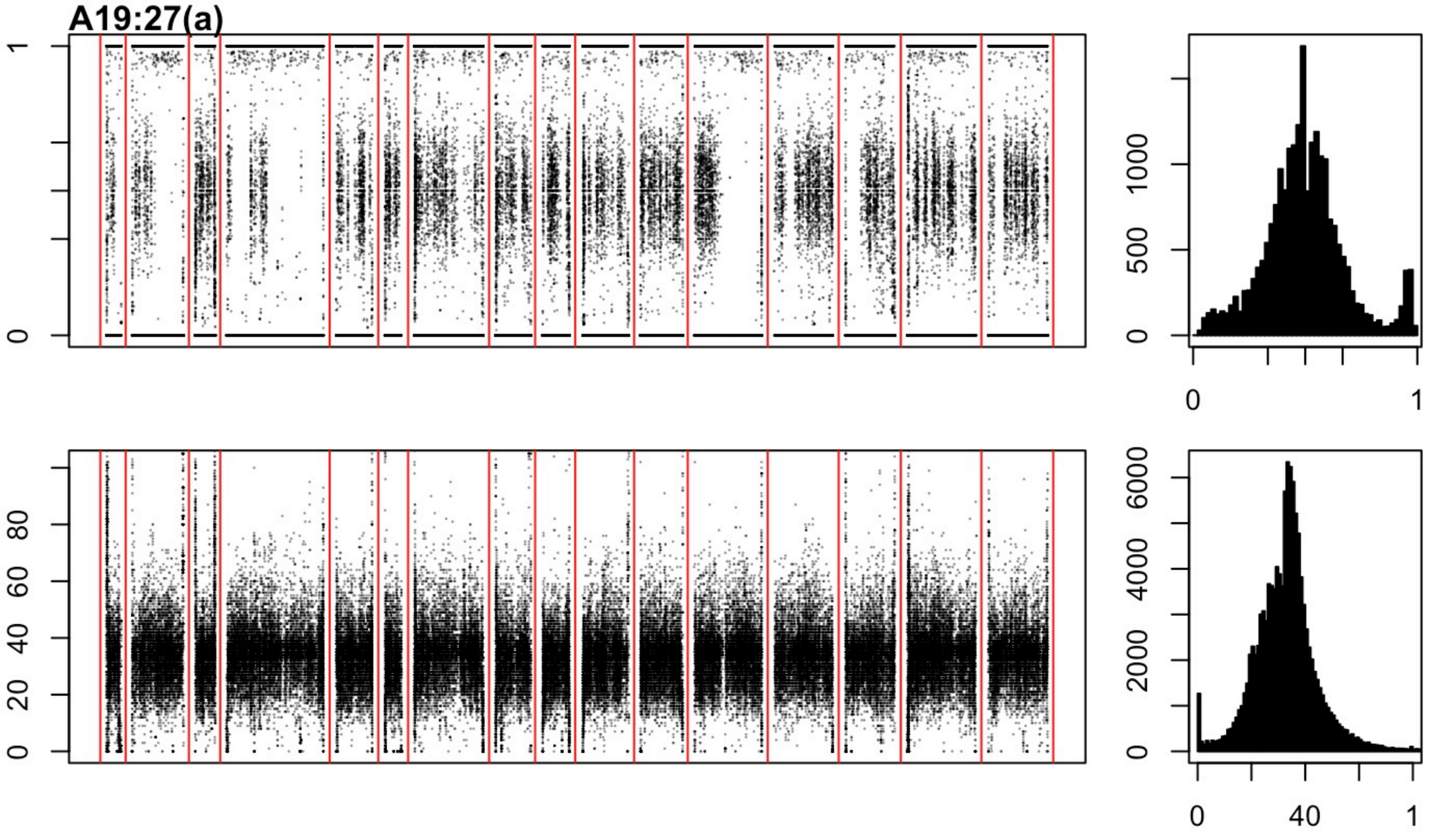
A19:27(a)
1
0
1000
500
0
0
1
80
60
40
20
0
6000
4000
2000
0
0
40
1

A19:27(b)

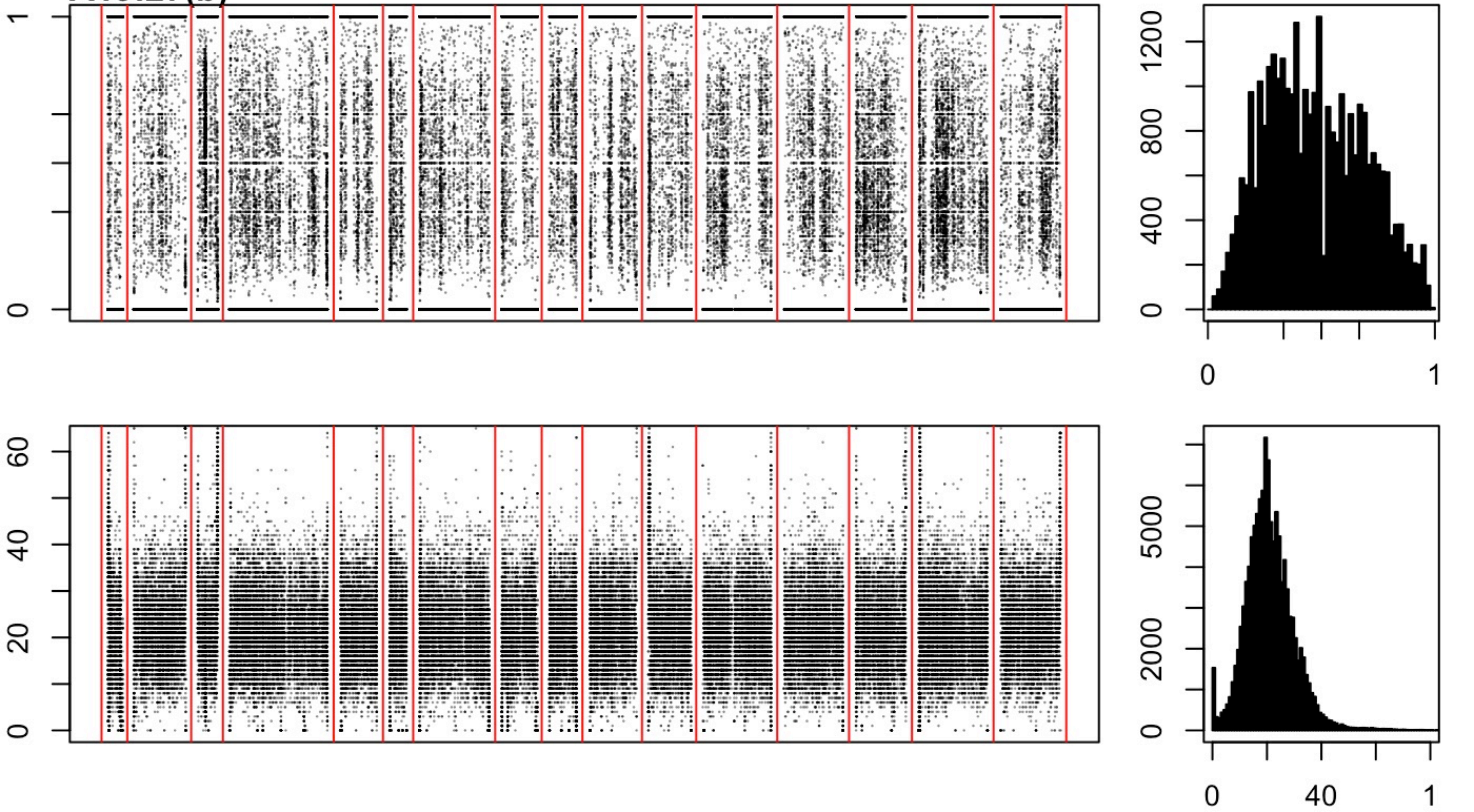

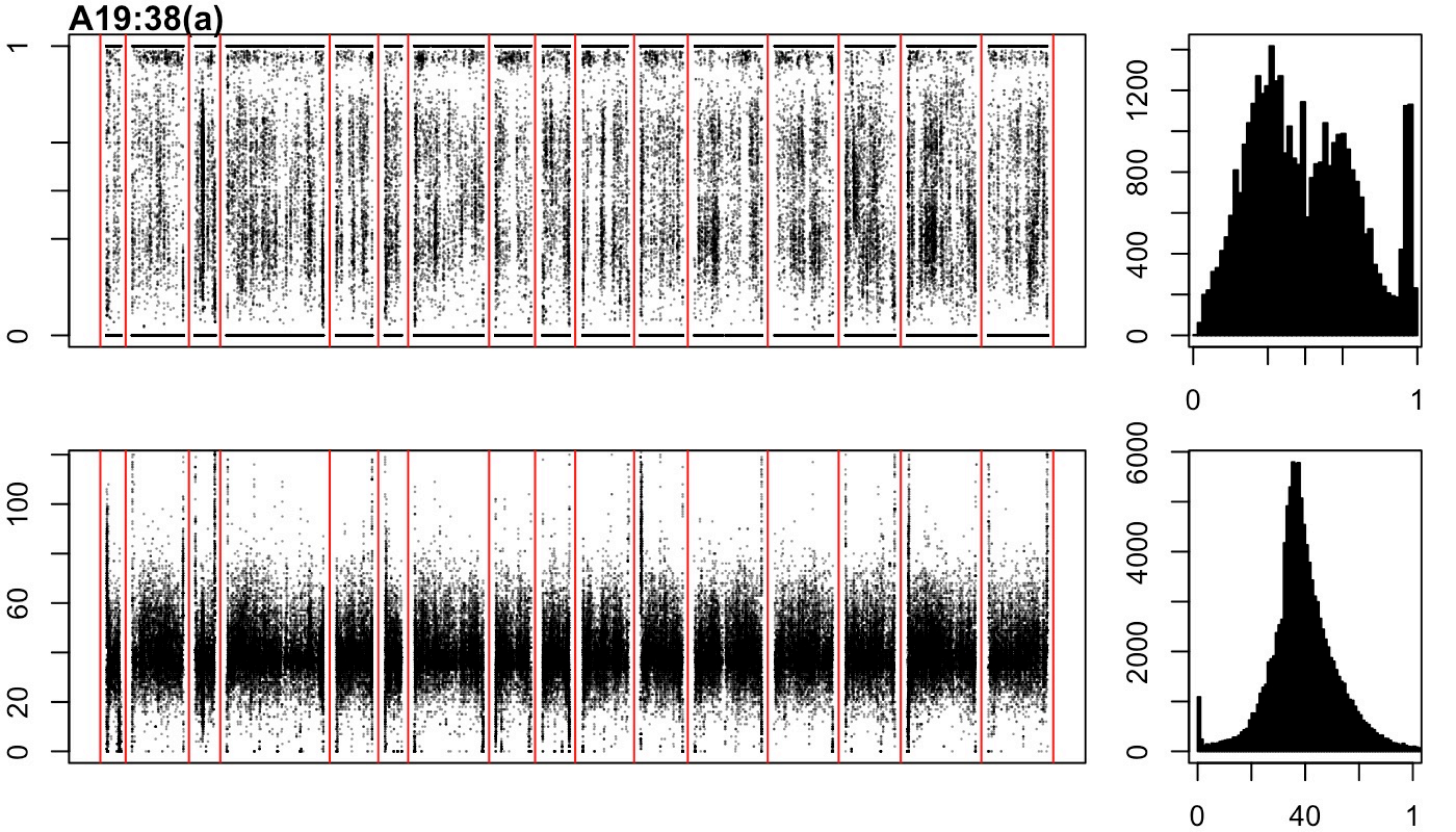
A19:38(a)
1
0
0
400
800
1200
0
1
100
60
20
0
6000
4000
2000
0
0
40
1

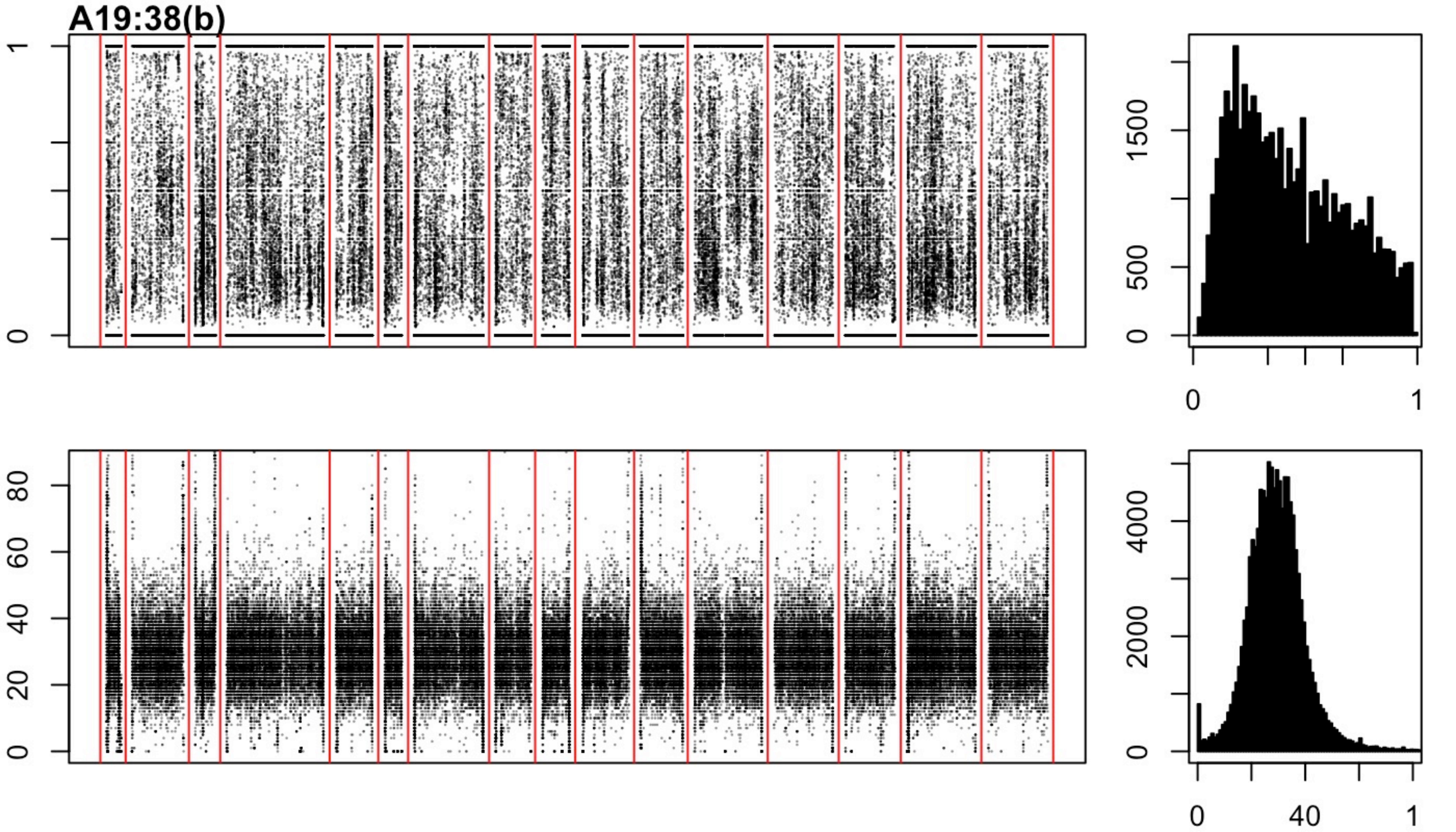

A19:38(b)
1
0
1500
500
0
0
1
80
60
40
20
0
4000
2000
0
0
40
1

A19:51(a)

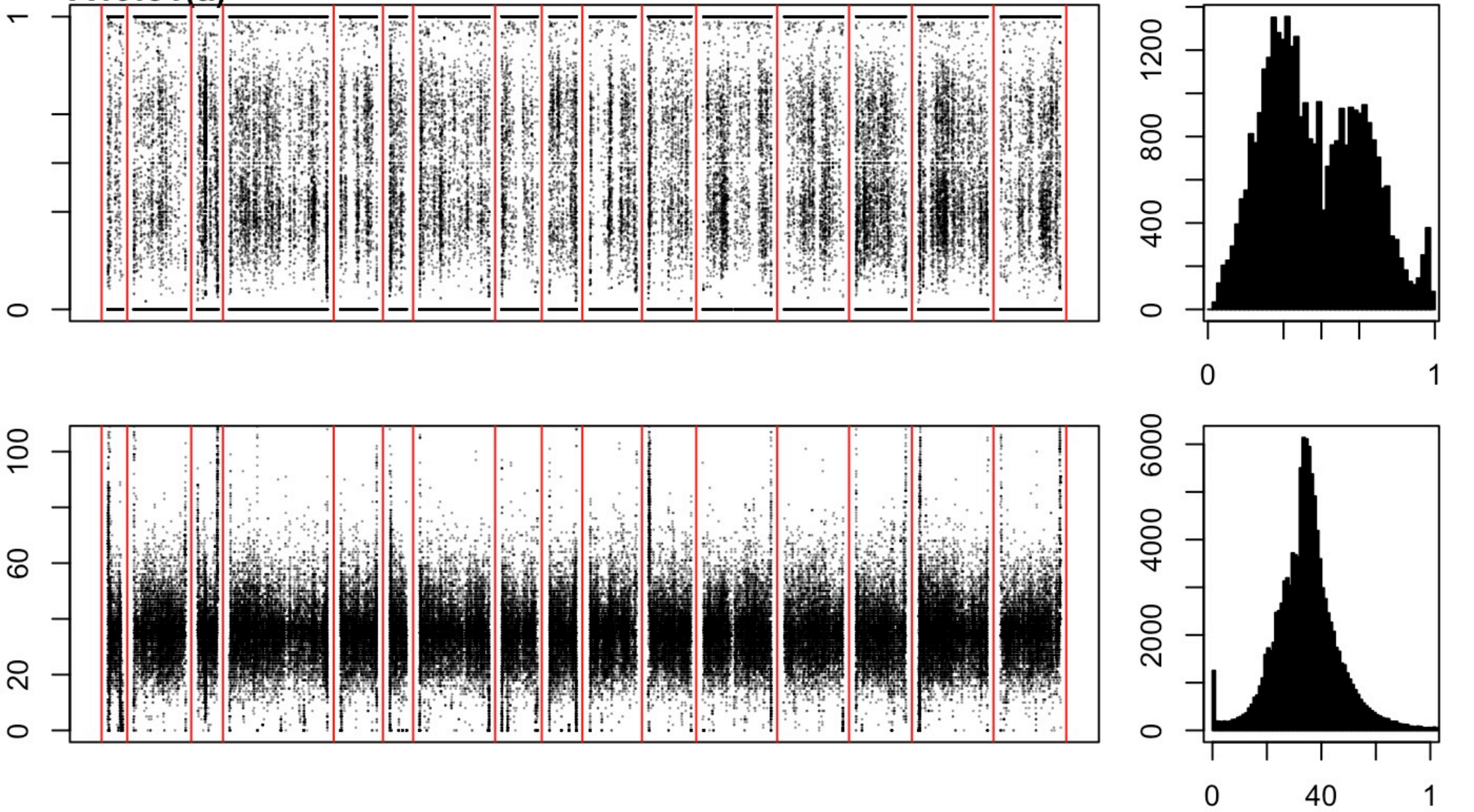

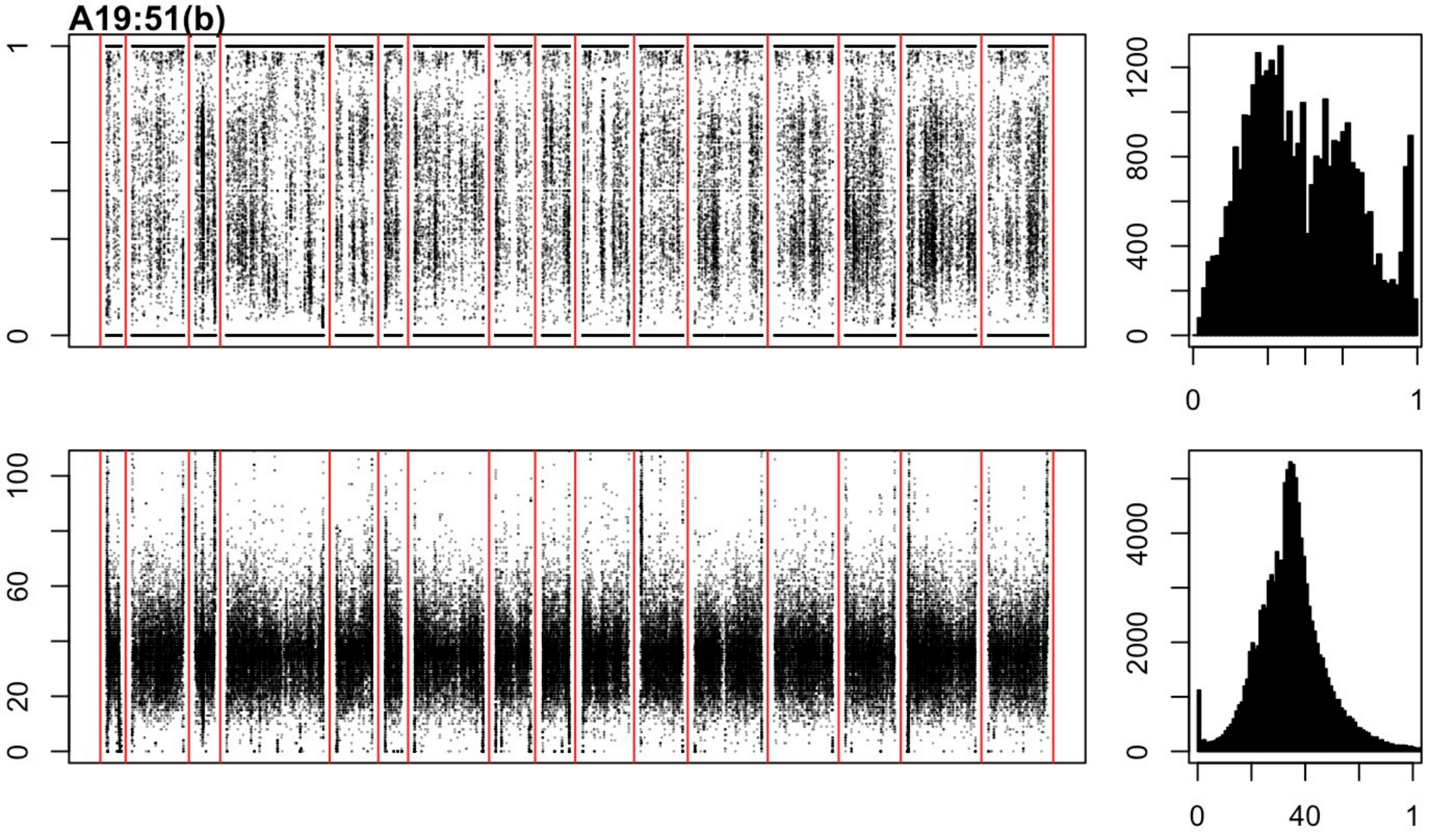
A19:51(b)
1
0
1200
800
400
0
0
1
100
60
20
0
4000
2000
0
0
40
1

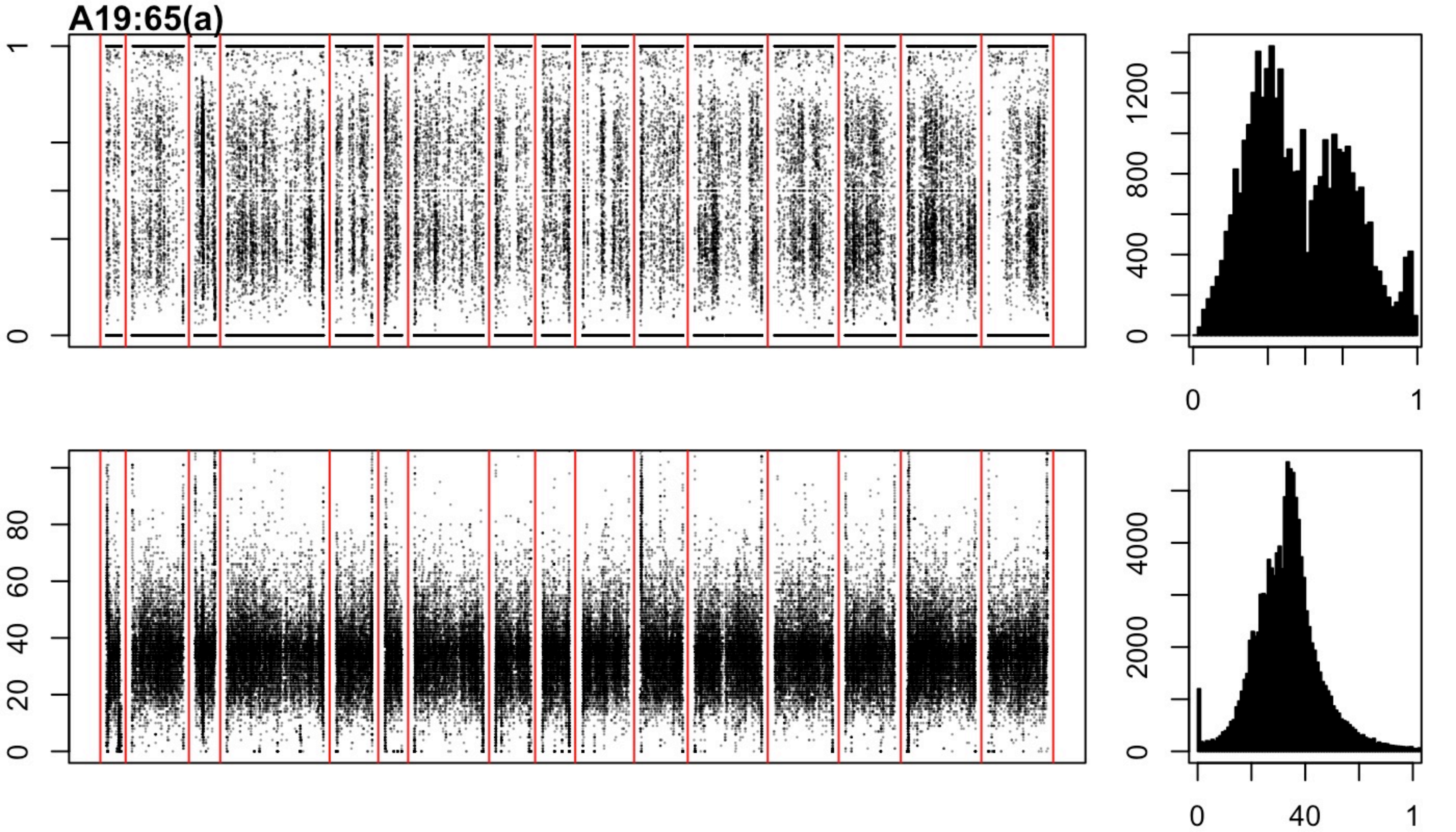
A19:65(a)
1
0
1200
800
400
0
0
1
80
60
40
20
0
4000
2000
0
0
40
1

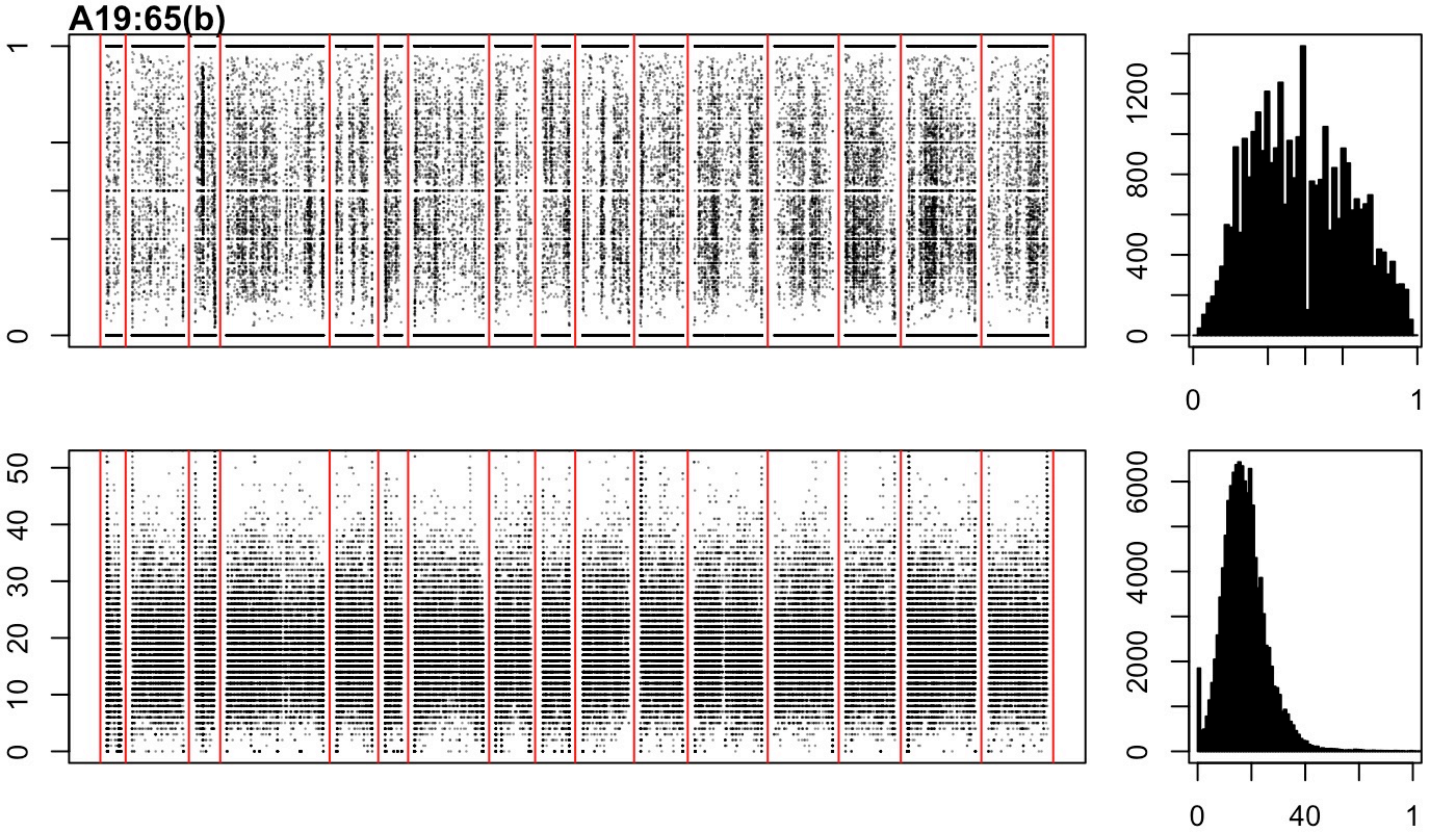
A19:65(b)
0
1
0
400
800
1200
0
1
0
10
20
30
40
50
0
2000
4000
6000
0
40
1

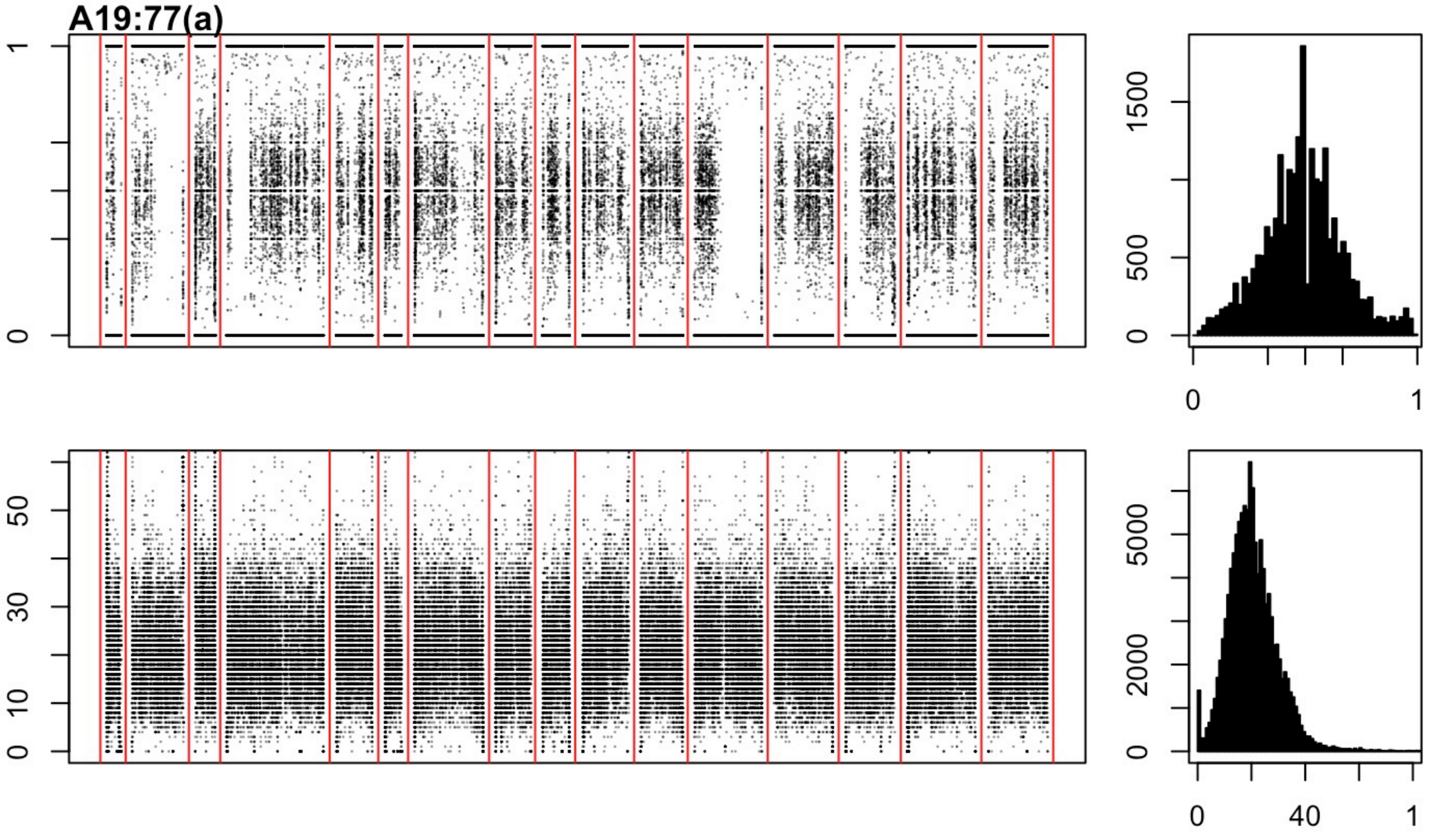
A19:77(a)
1
0
1500
500
0
0
1
50
30
10
0
5000
2000
0
0
40
1

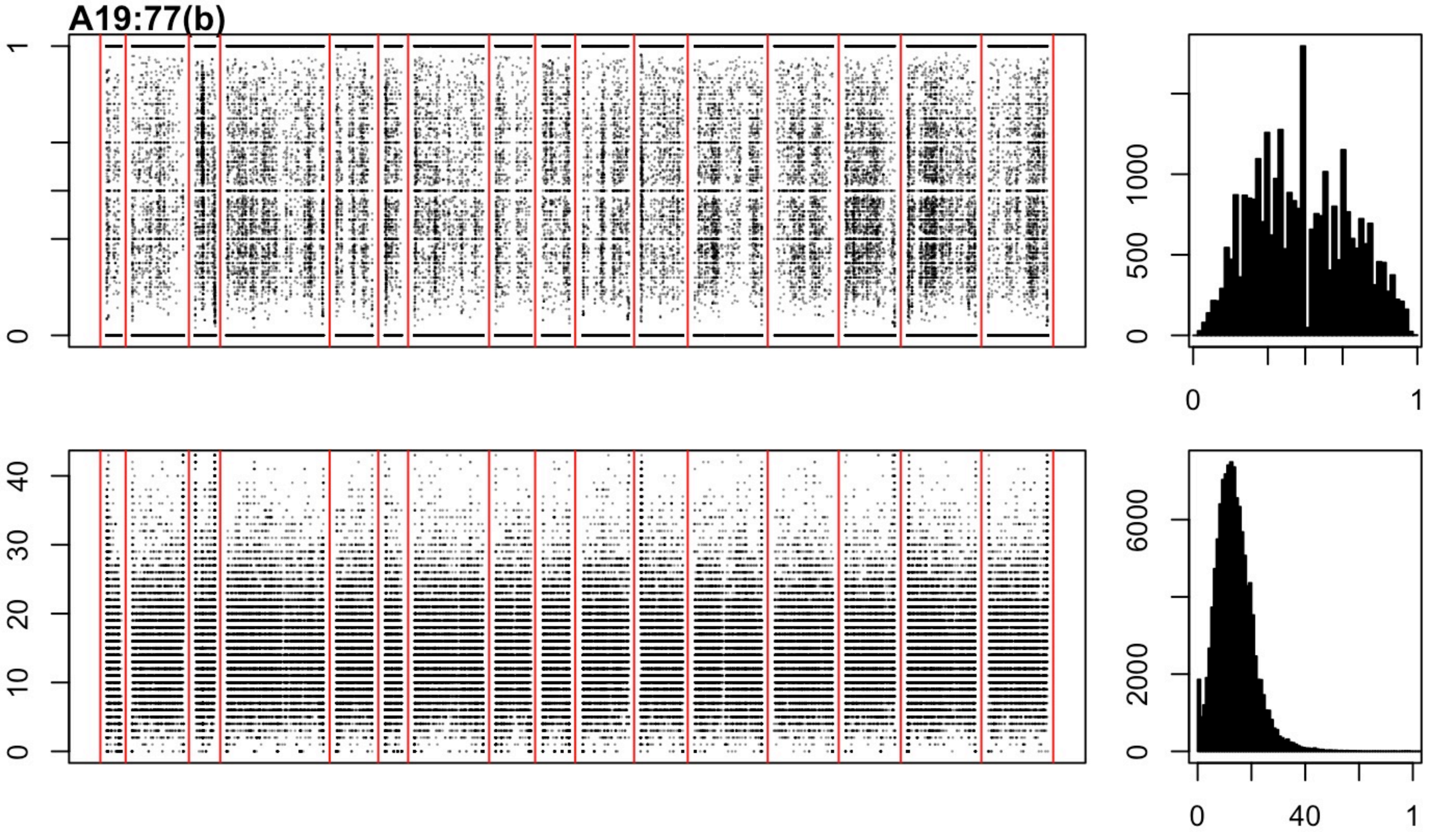
A19:77(b)
0
1
0
500
1000
0
1
0
10
20
30
40
0
2000
6000
0
40
1

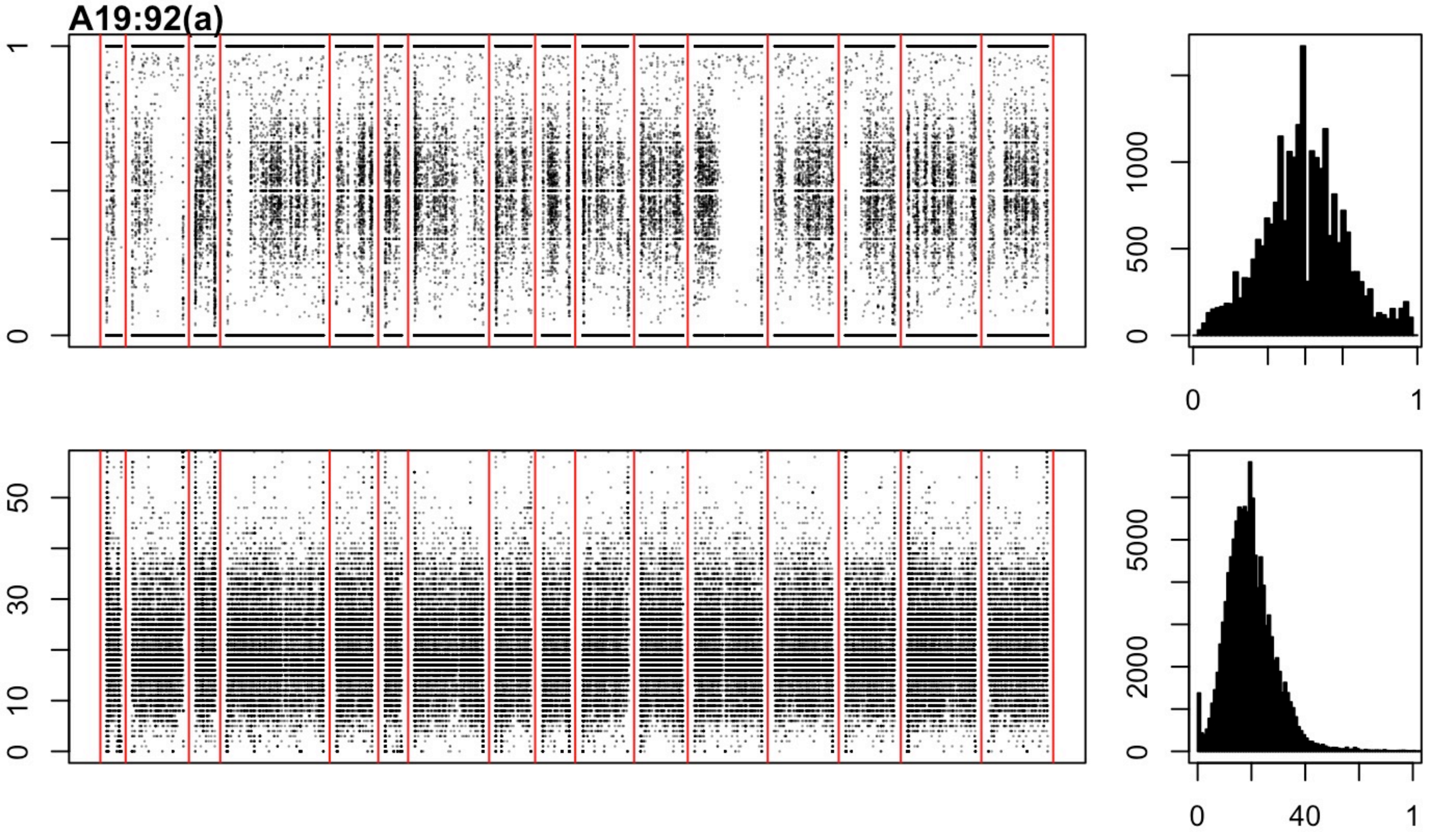
A19:92(a)
1
0
0
500
1000
0
1
50
30
10
0
0
2000
5000
0
40
1

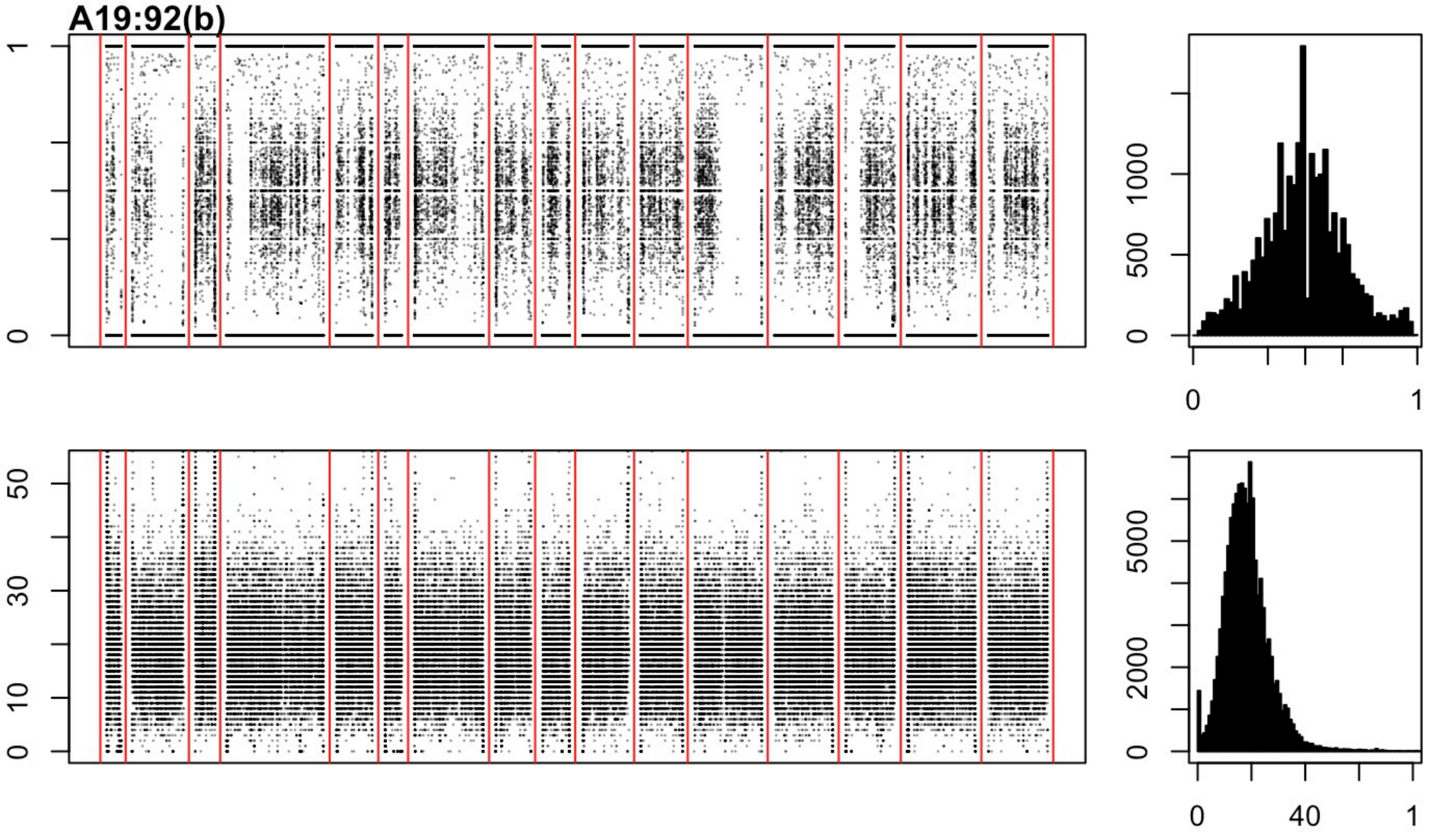
A19:92(b)
1
0
1000
500
0
0
1
50
30
10
0
5000
2000
0
0
40
1

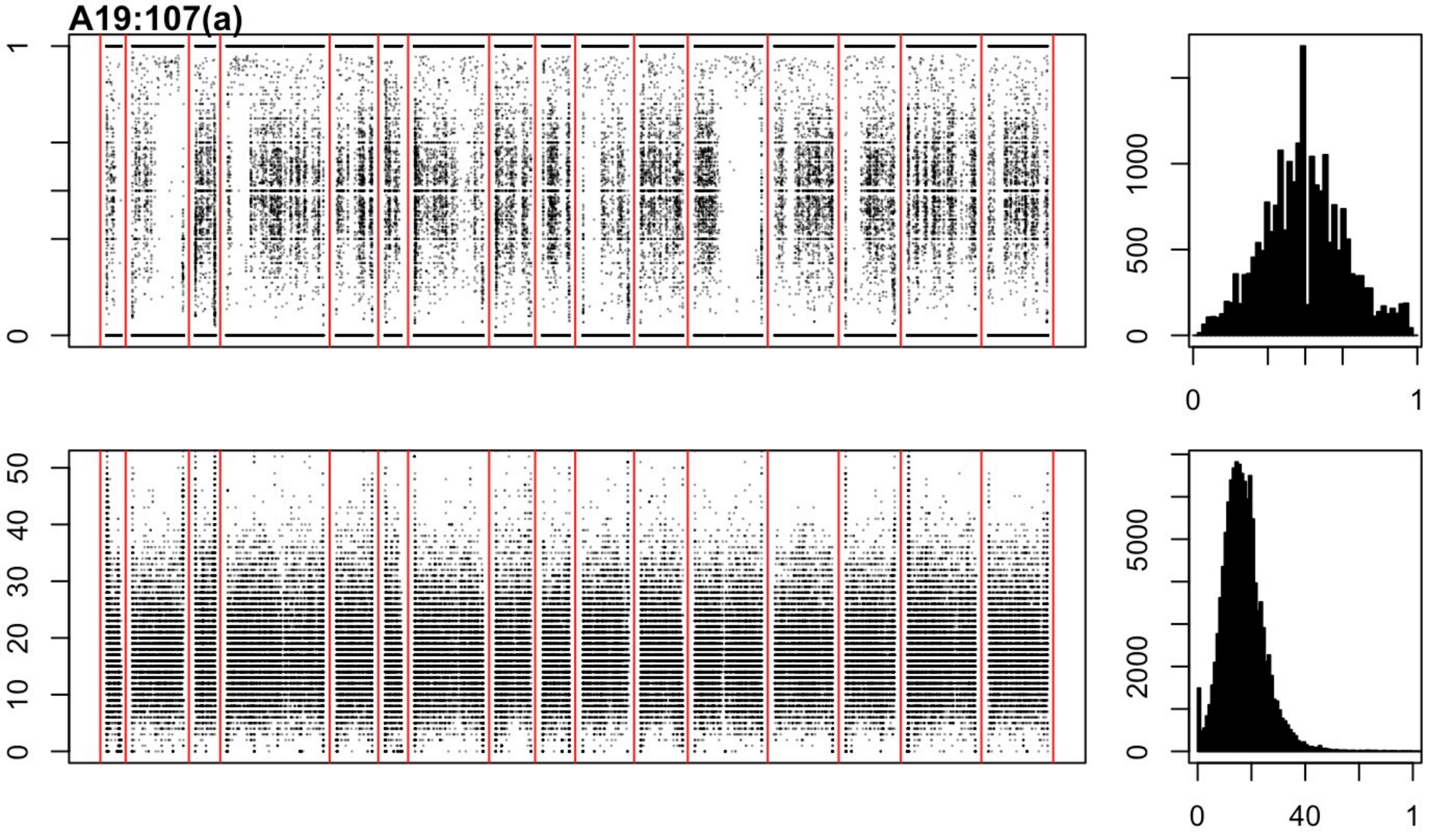

A19:107(a)
1
0
1000
500
0
0
1
50
40
30
20
10
0
5000
2000
0
0
40
1

A19:107(b)

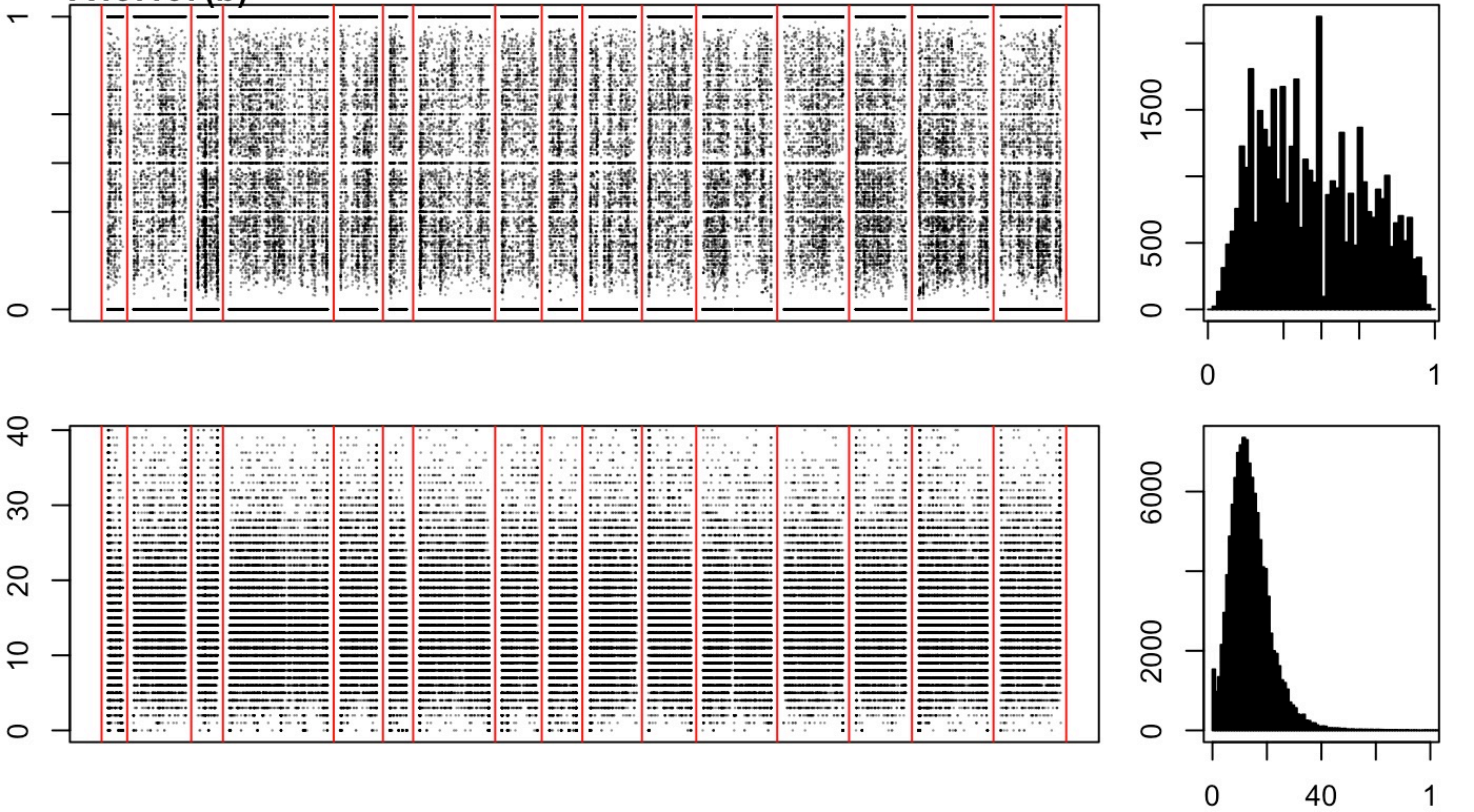

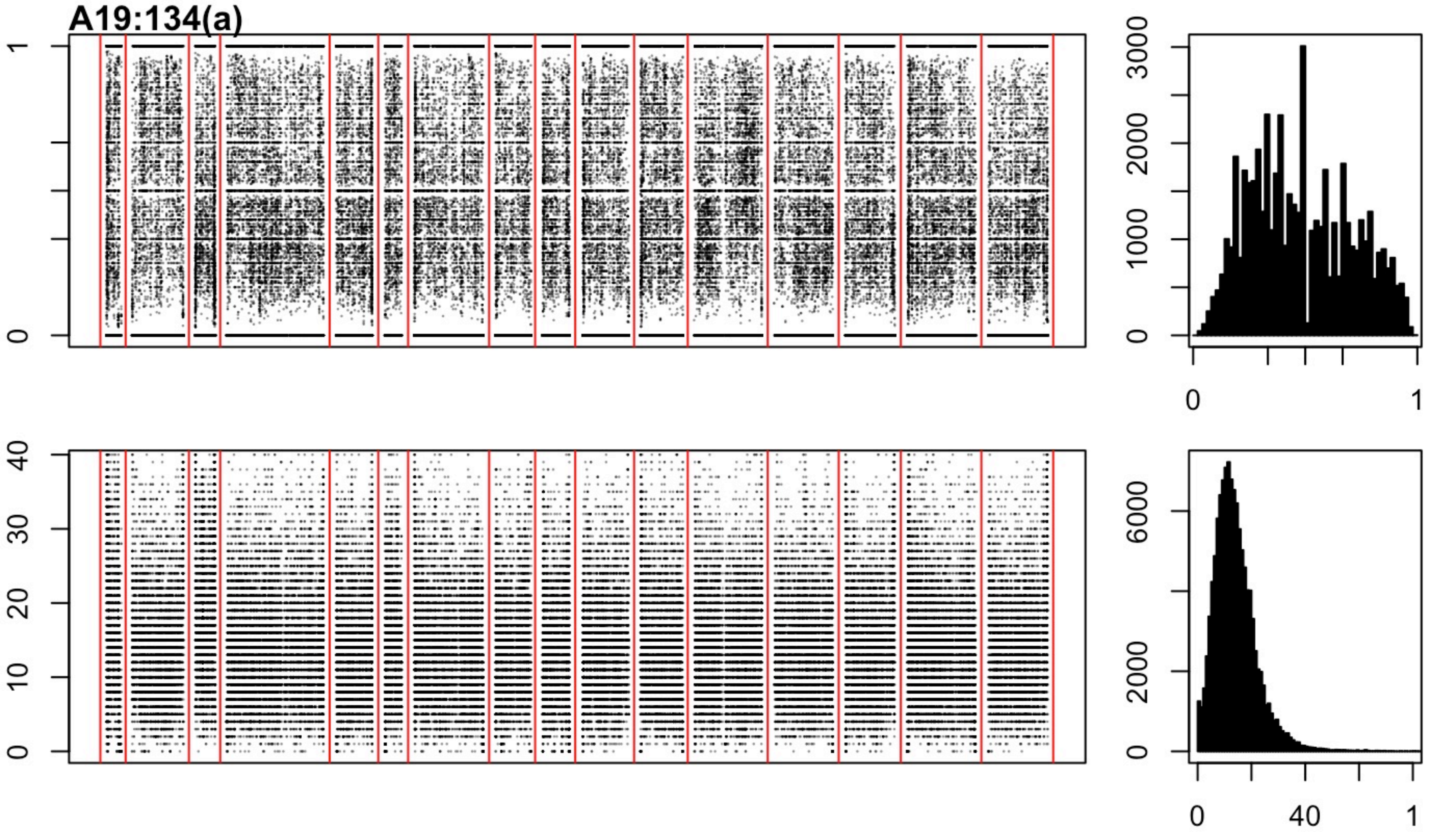
A19:134(a)
1
0
3000
2000
1000
0
0
1
40
30
20
10
0
6000
2000
0
0
40
1

A19:134(b)

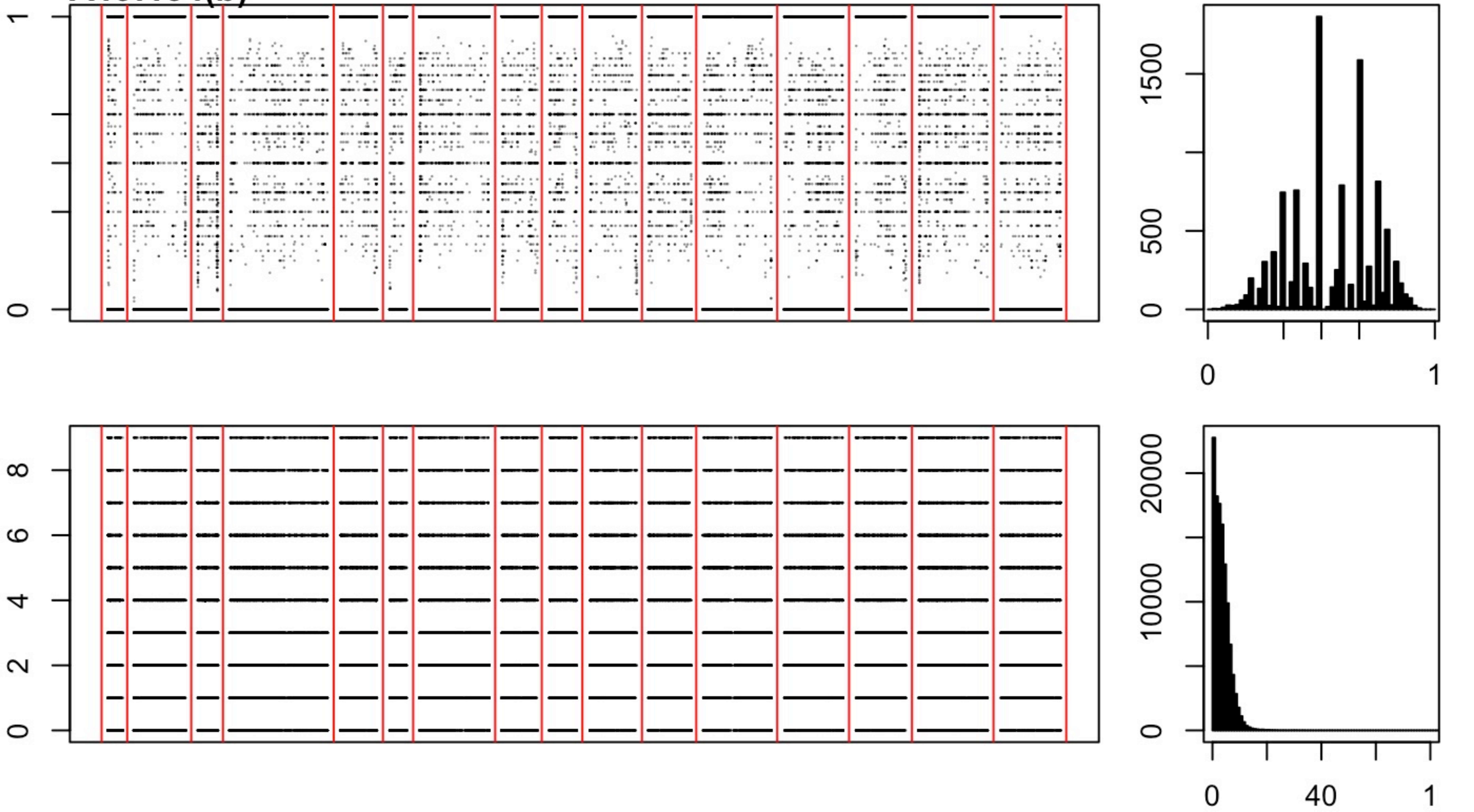

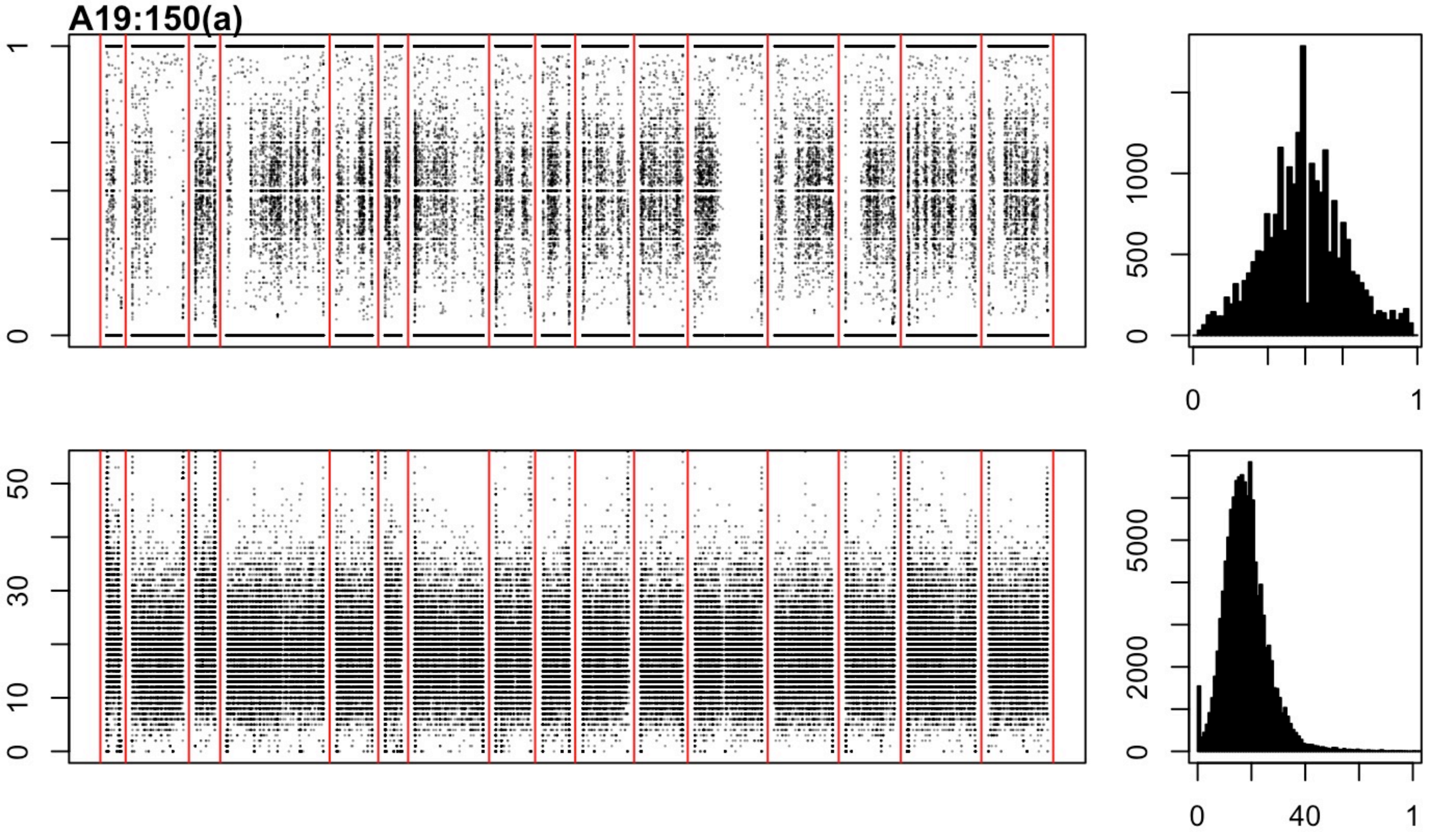

A19:150(a)
1
0
0
500
1000
0
1
50
30
10
0
0
2000
5000
0
40
1

A19:150(b)

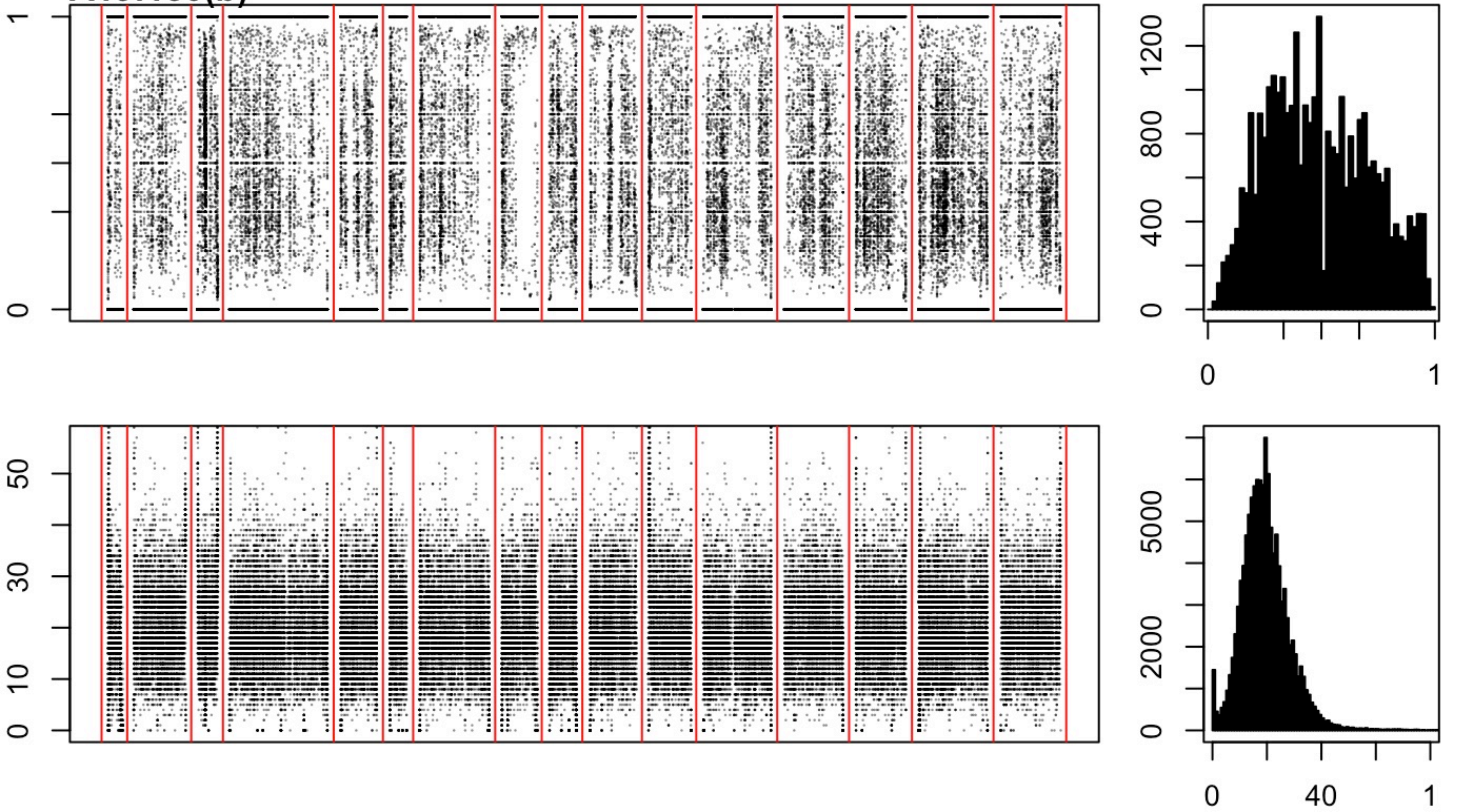

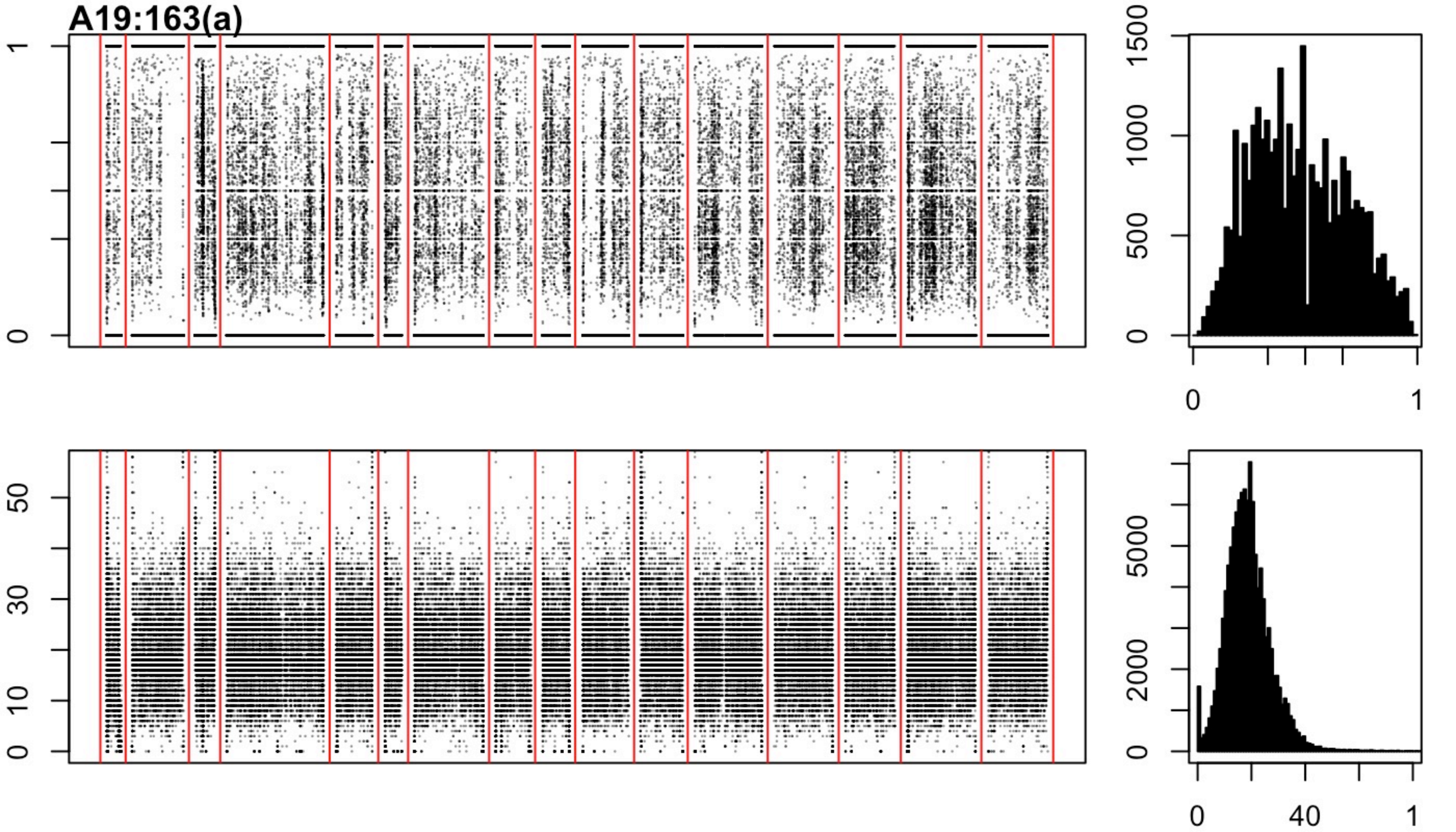
A19:163(a)
1
0
1500
1000
500
0
0
1
50
30
10
0
5000
2000
0
0
40
1

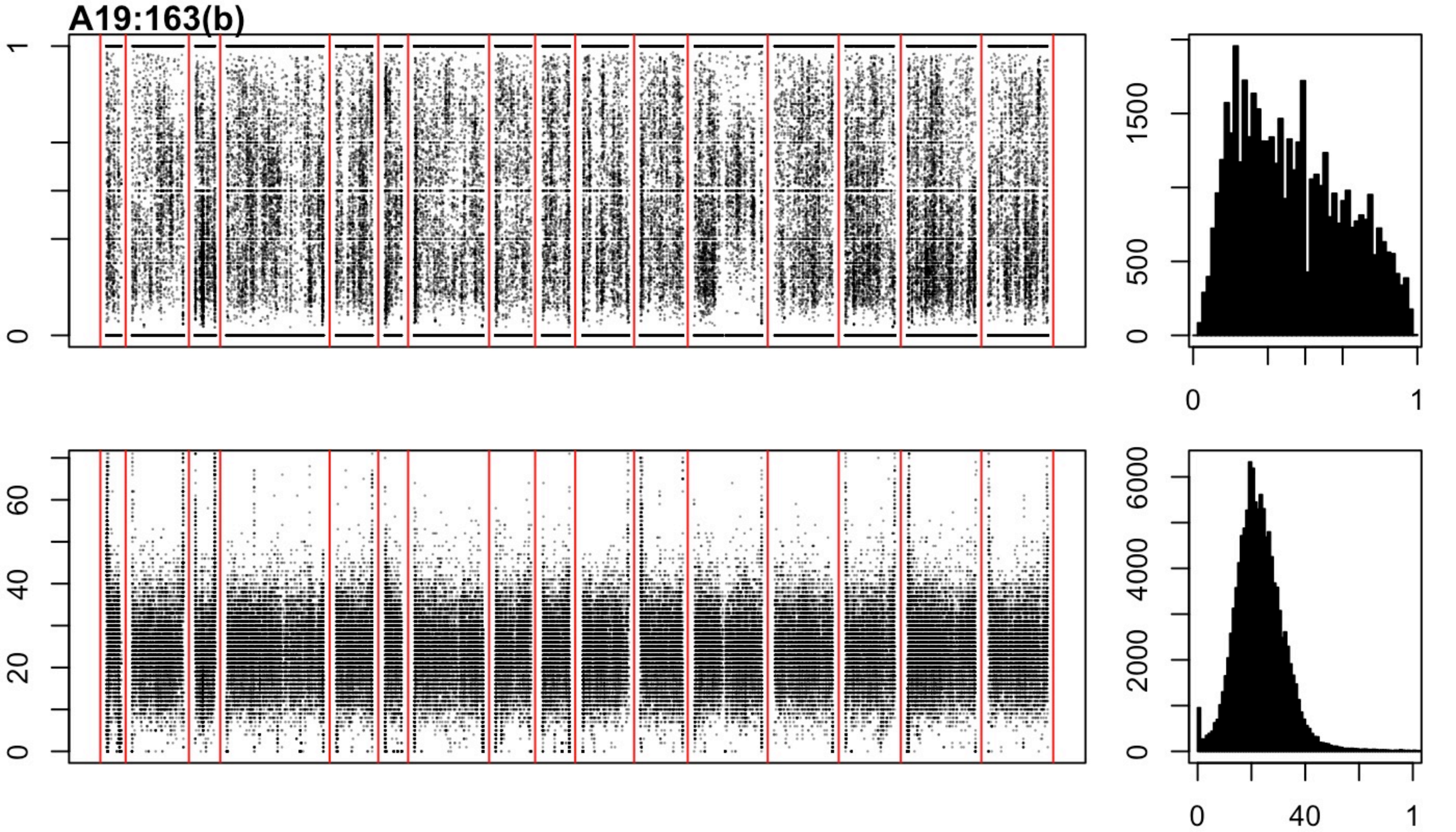
A19:163(b)
1
0
1500
500
0
0
1
60
40
20
0
6000
4000
2000
0
0
40
1

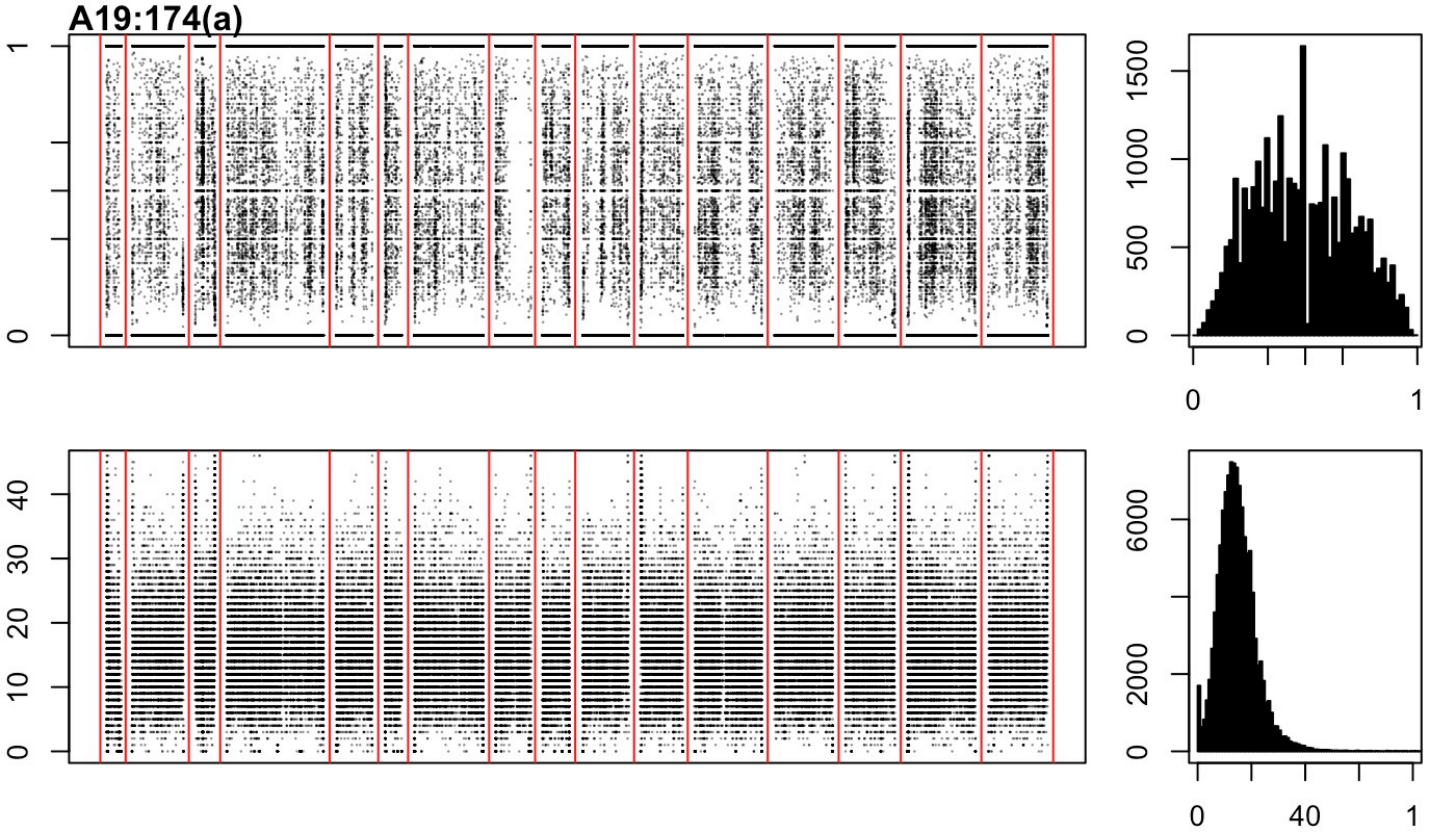
A19:174(a)
1
0
1500
1000
500
0
0
1
40
30
20
10
0
6000
2000
0
0
40
1

A19:174(b)

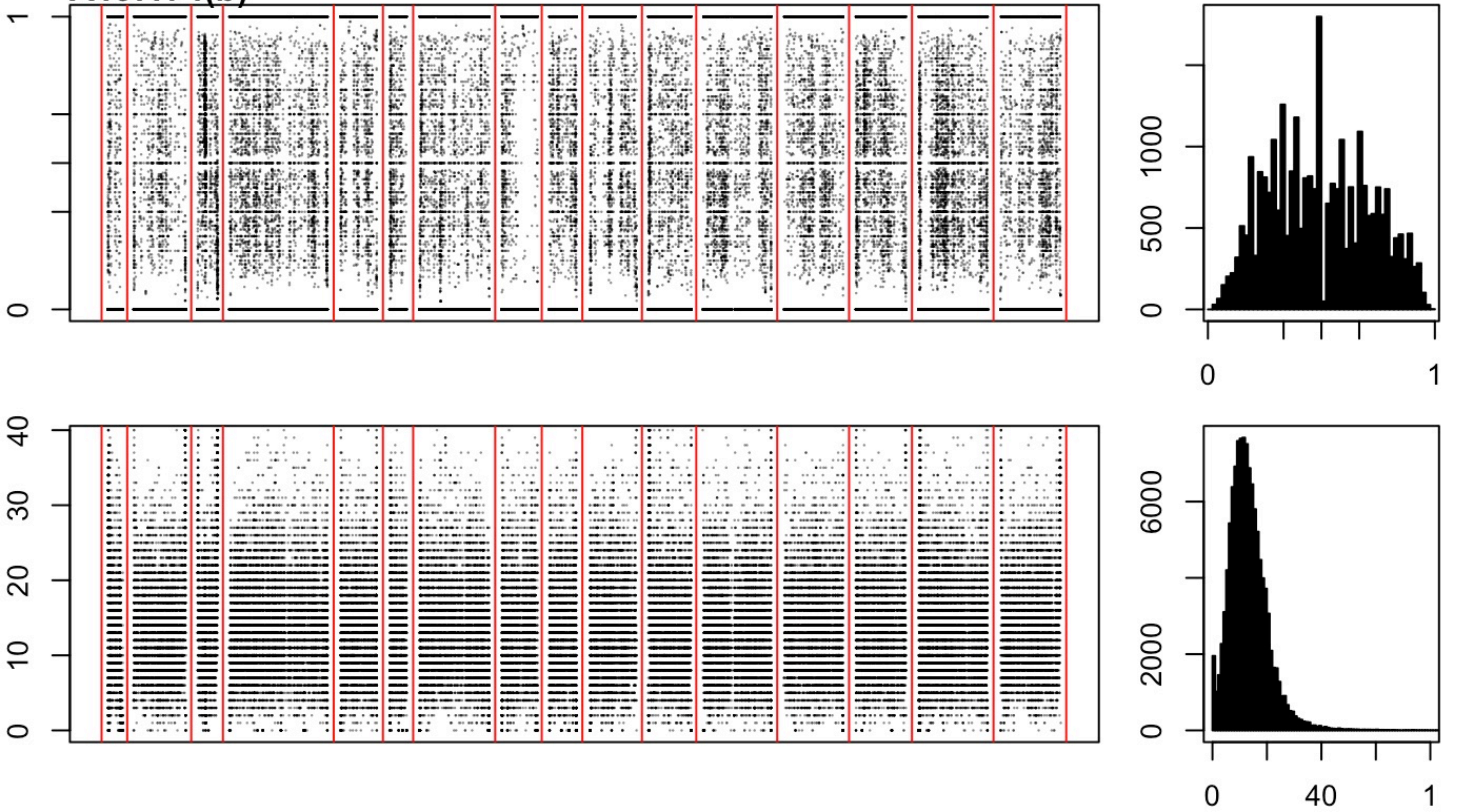

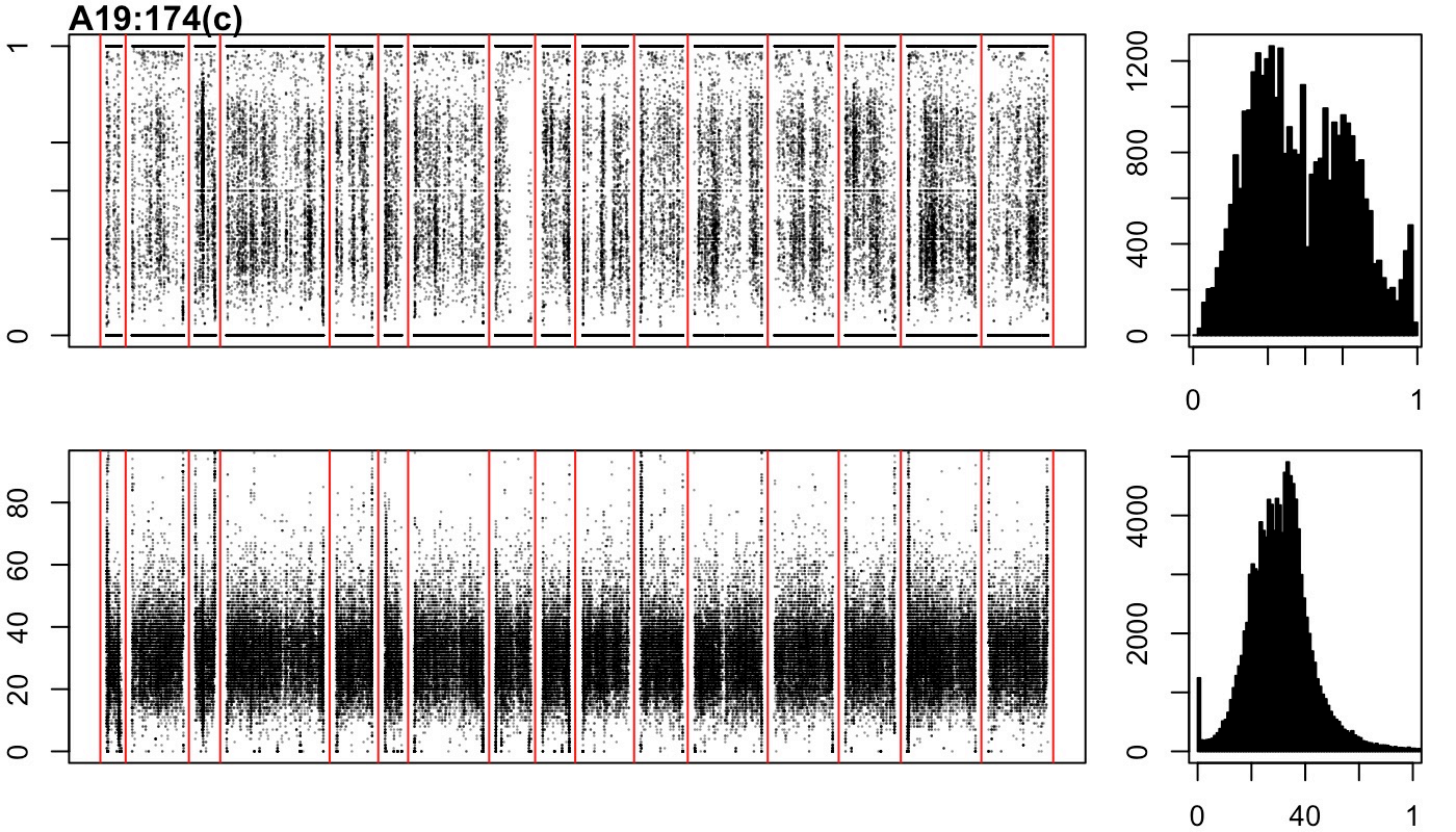

A19:174(c)
1
0
1200
800
400
0
0
1
80
60
40
20
0
4000
2000
0
0
40
1

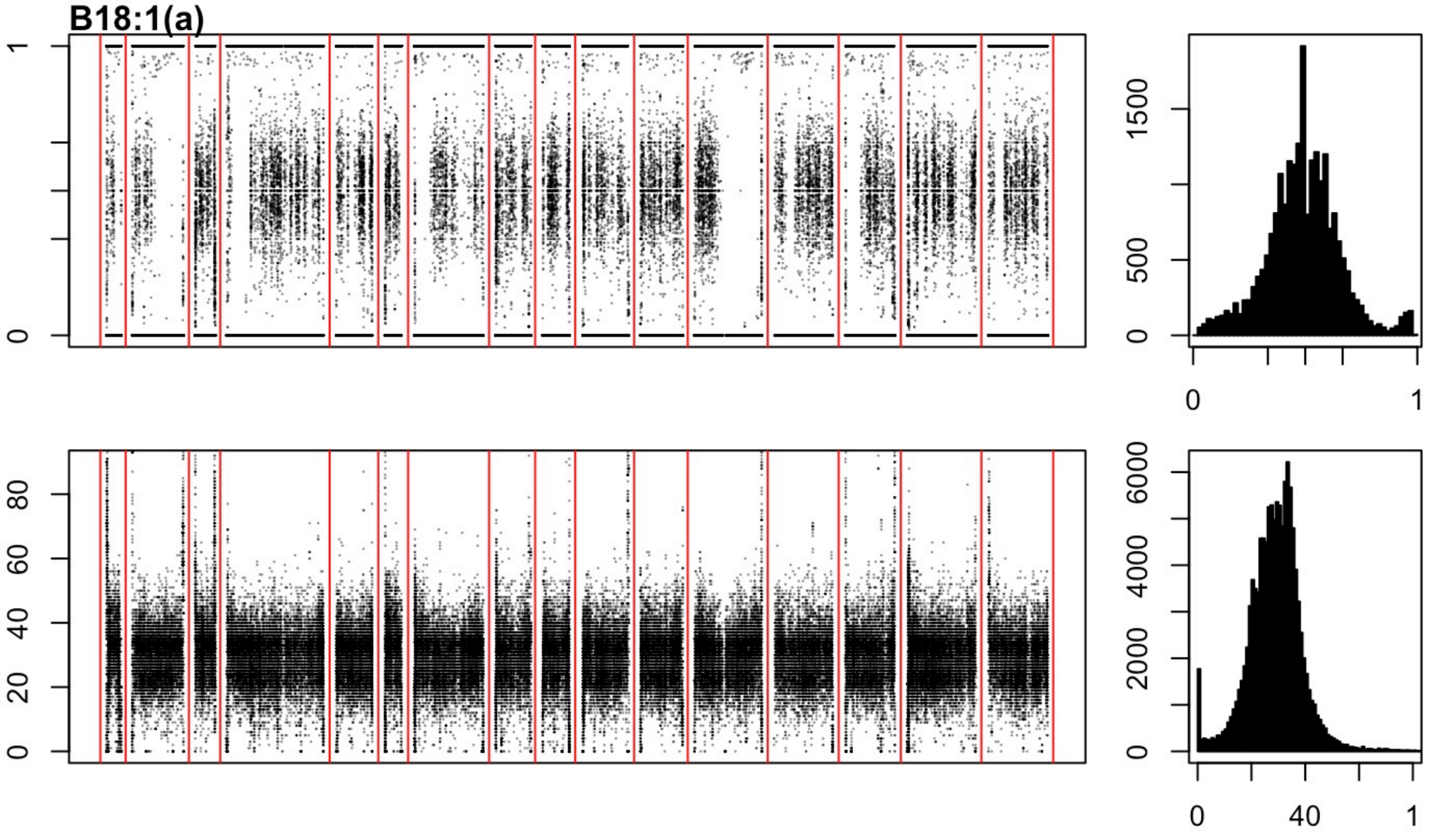

B18:1(a)
1
0
1500
500
0
0
1
80
60
40
20
0
6000
4000
2000
0
0
40
1

B18:1(b)

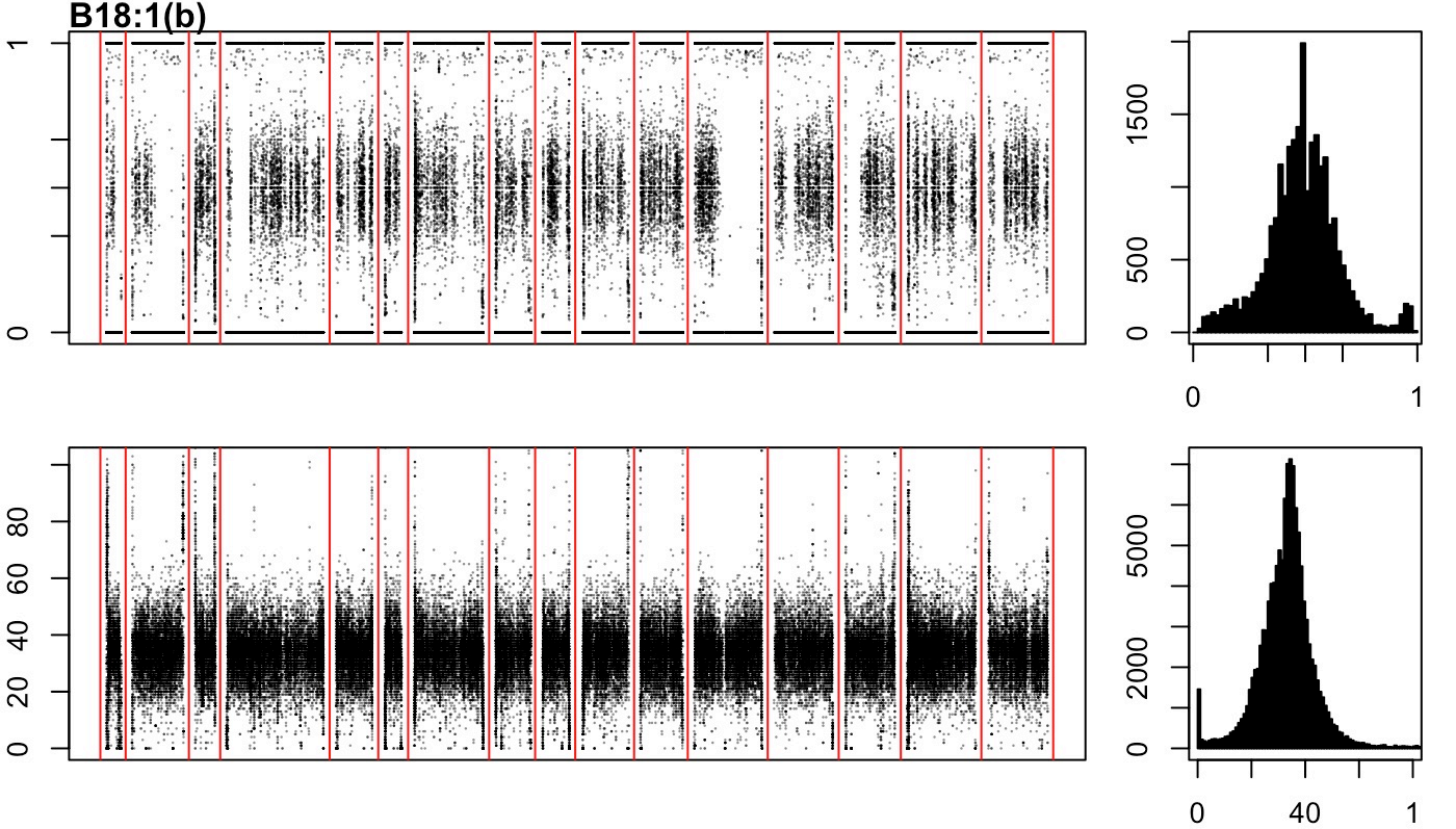

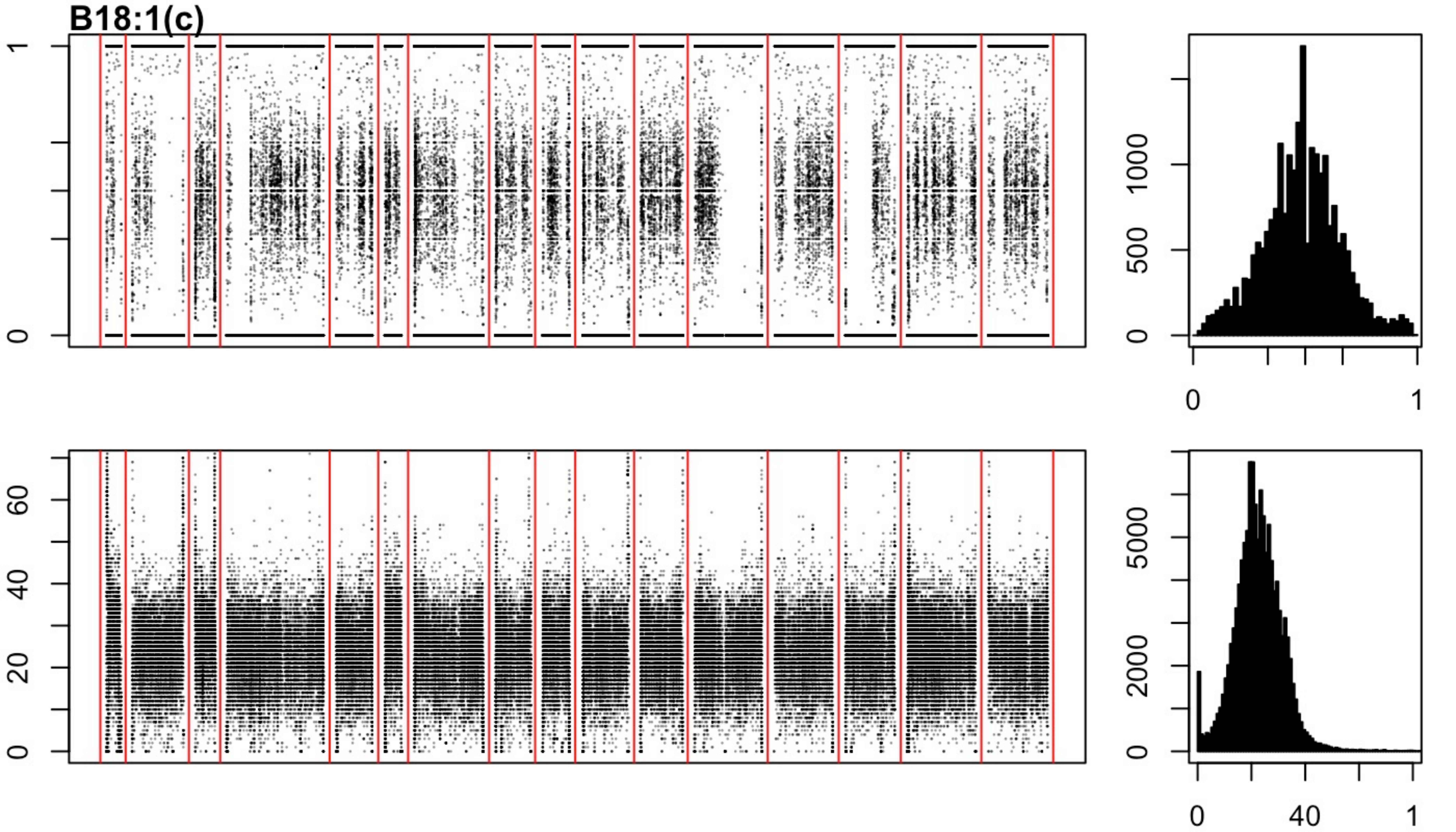

B18:1(c)
1
0
1000
500
0
0
1
60
40
20
0
5000
2000
0
0
40
1

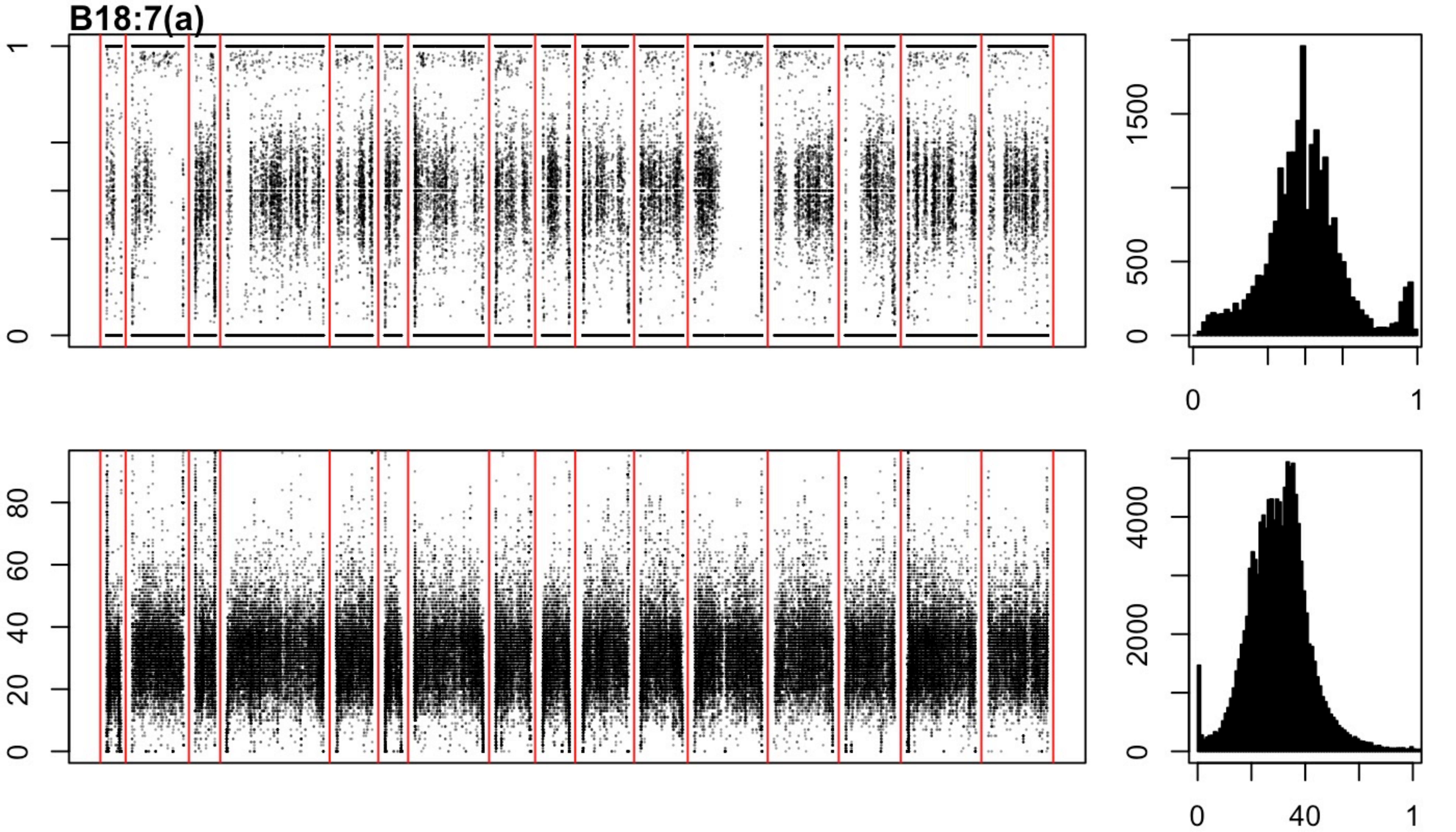

B18:7(a)
1
0
1500
500
0
0
1
80
60
40
20
0
4000
2000
0
0
40
1

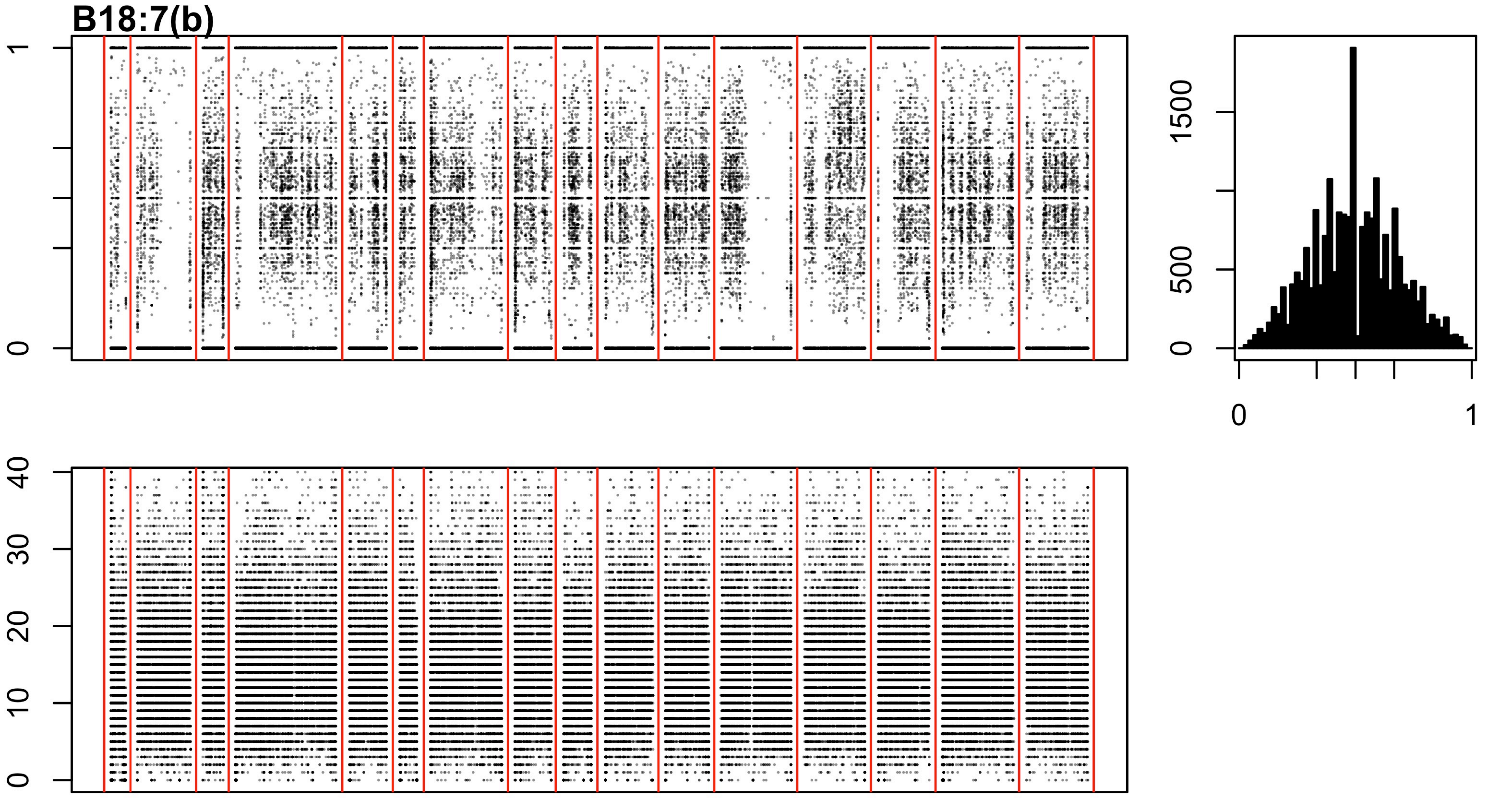
B18:7(b)
1
0
1500
500
0
0
1
40
30
20
10
0

B18:9(a)

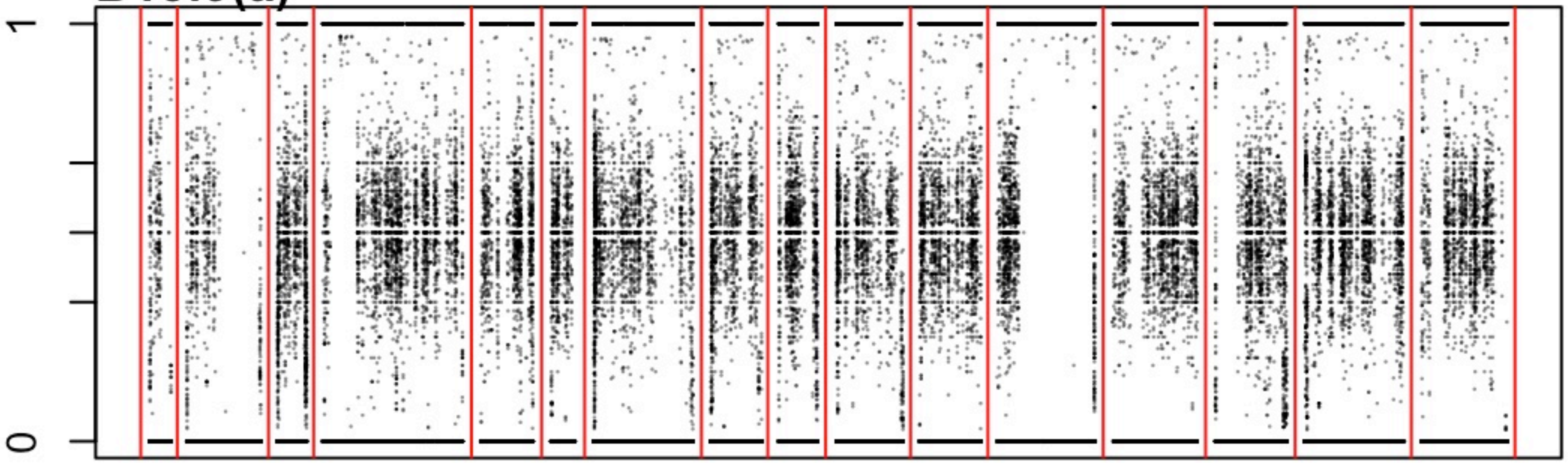

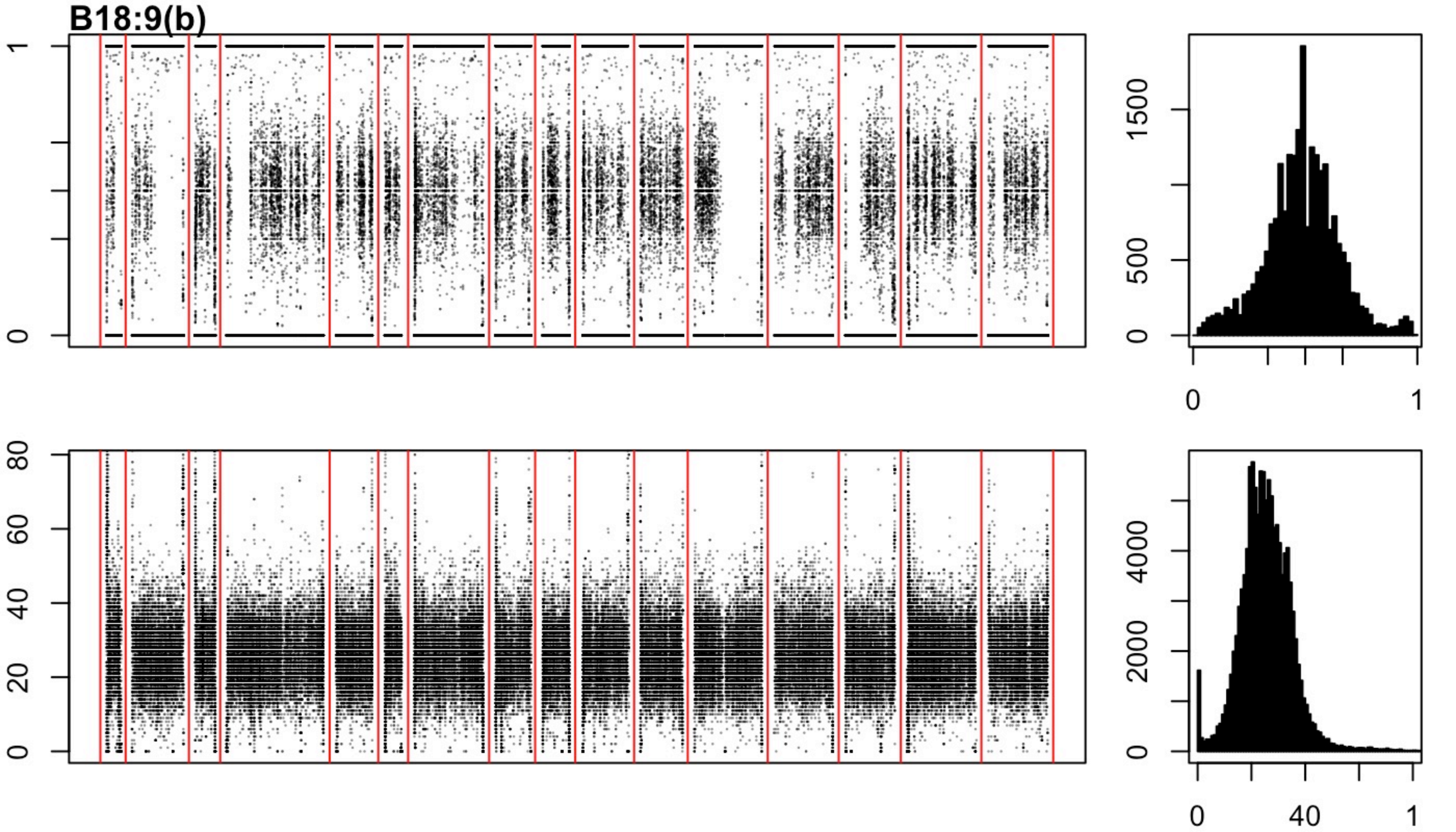

B18:9(b)
1
0
1500
500
0
0
1
80
60
40
20
0
4000
2000
0
0
40
1

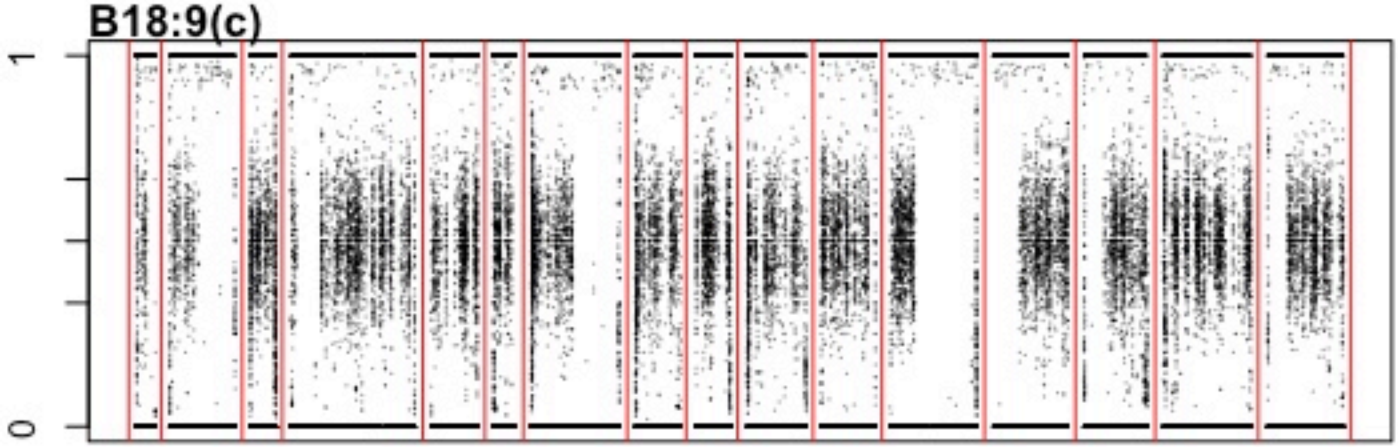
B18:9(c)
1
0

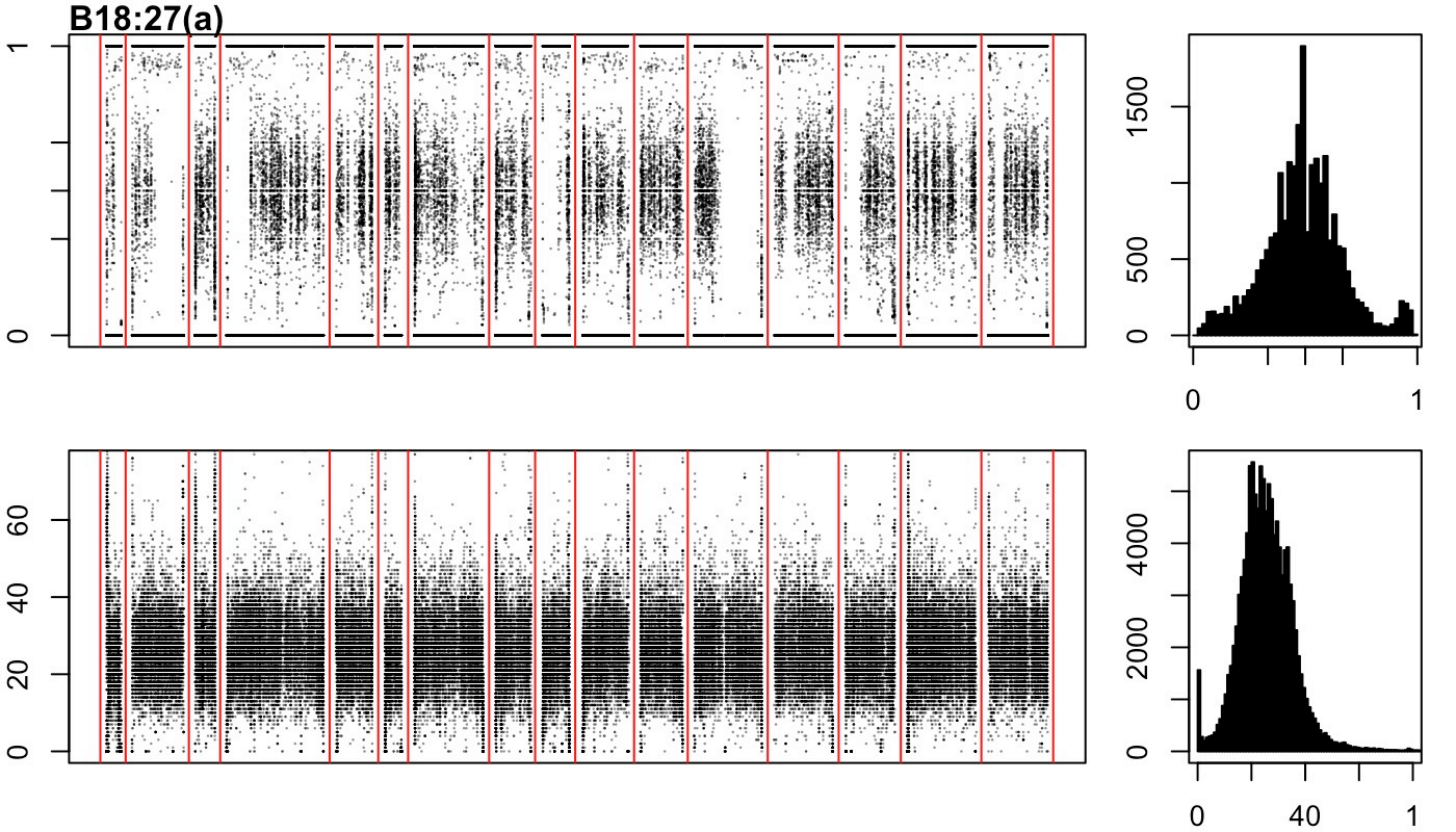

B18:27(a)
1
0
1500
500
0
0
1
60
40
20
0
4000
2000
0
0
40
1

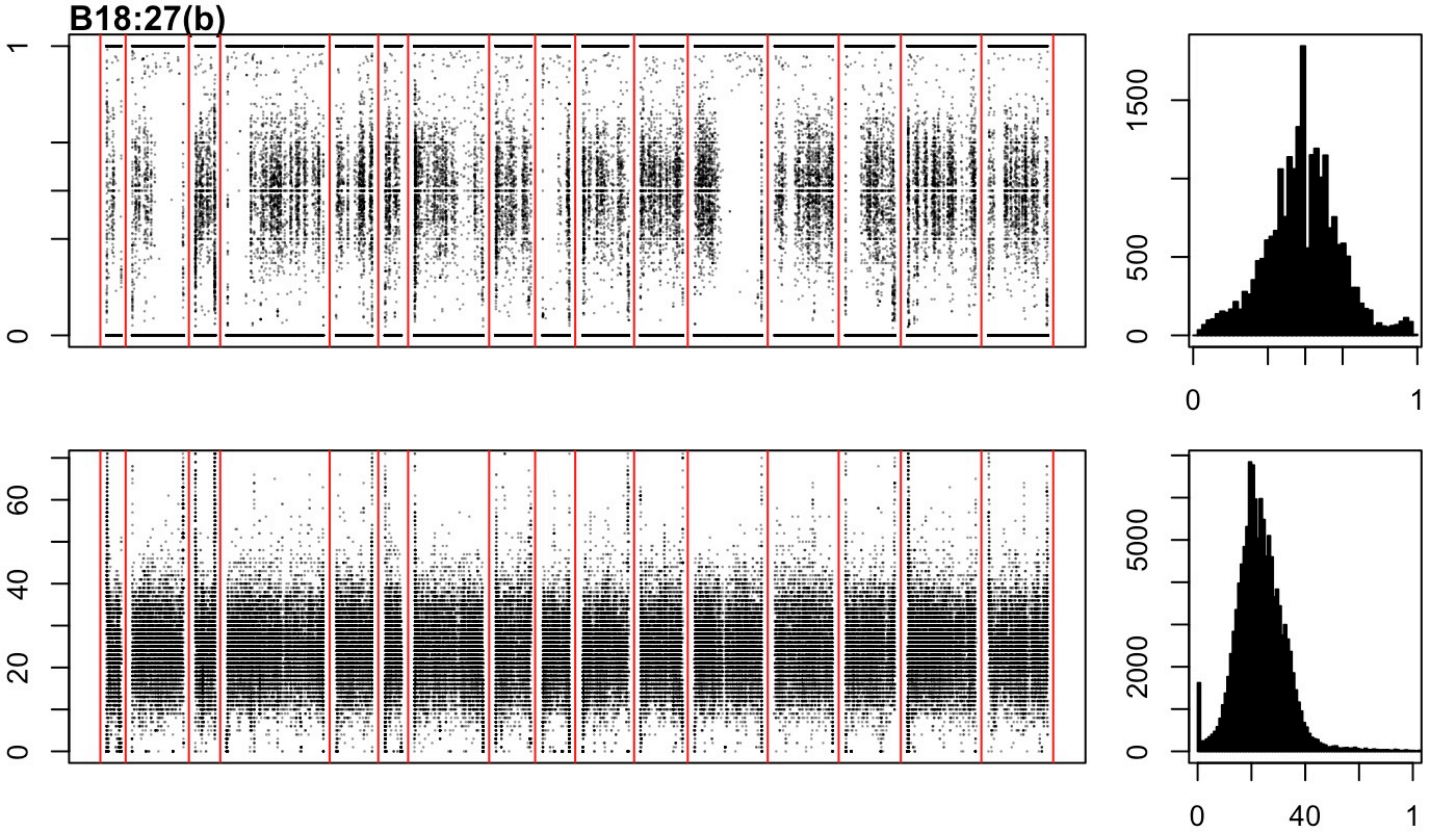

B18:27(b)
1
0
1500
500
0
0
1
60
40
20
0
5000
2000
0
0
40
1

B18:27(c)

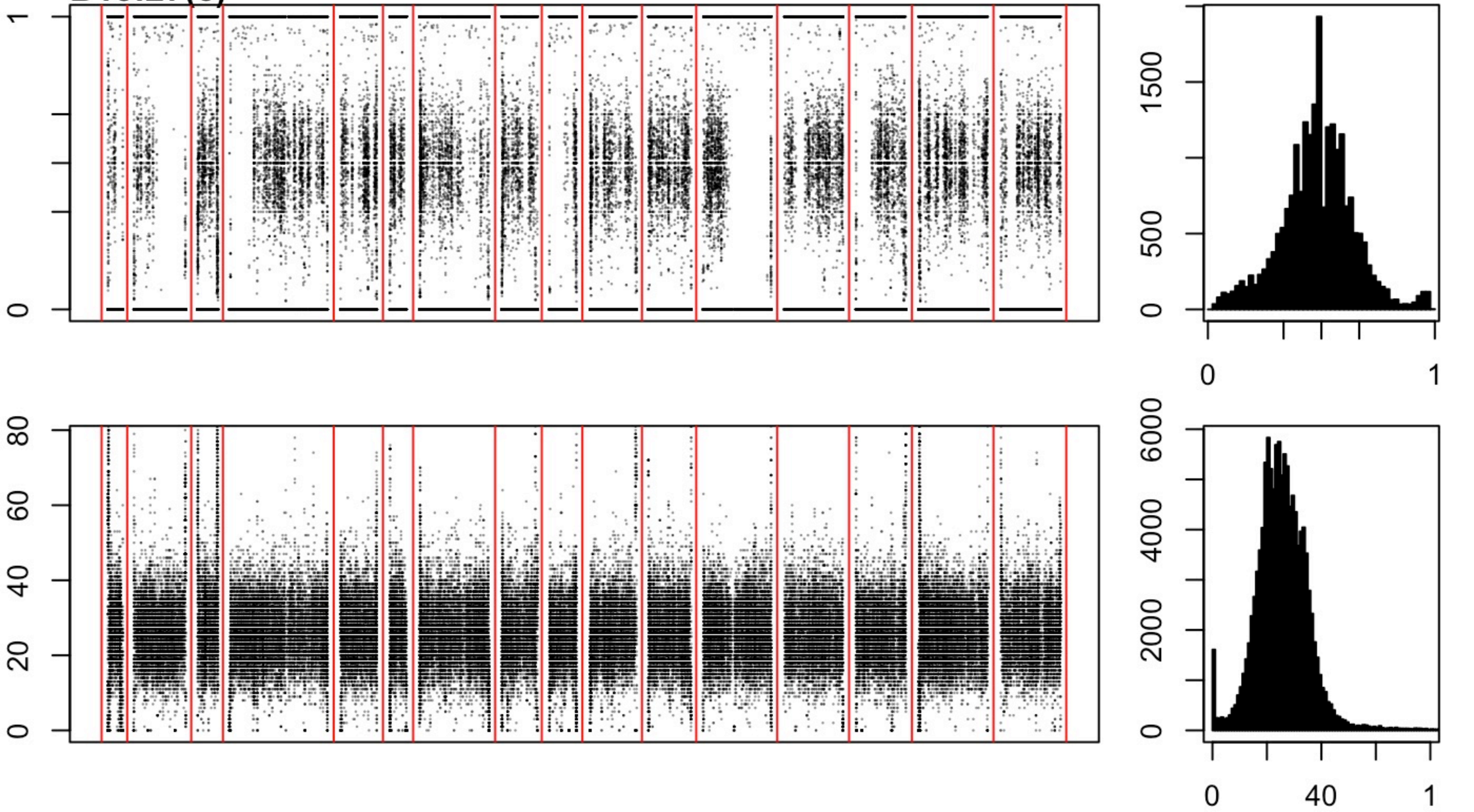

B18:47(a)

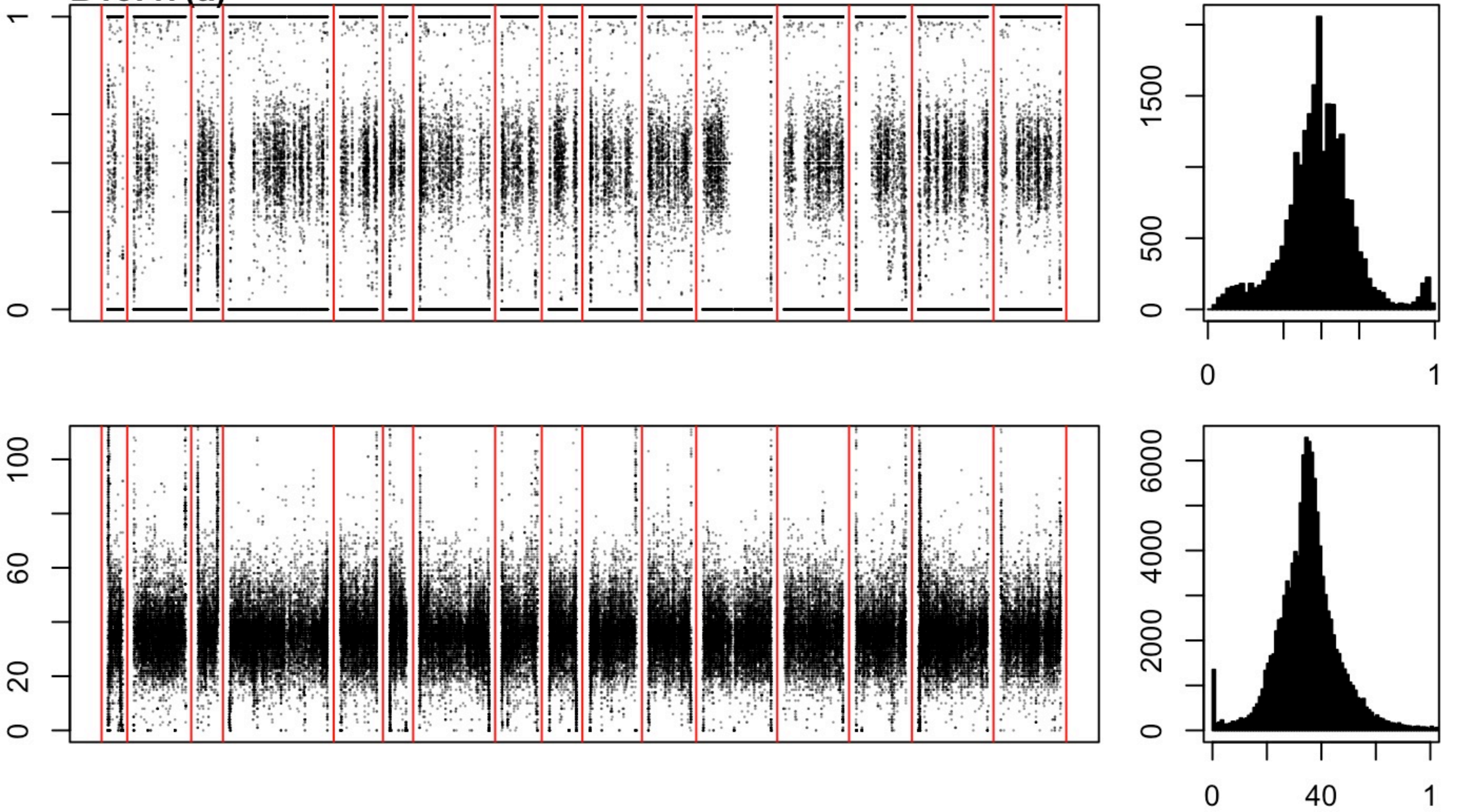

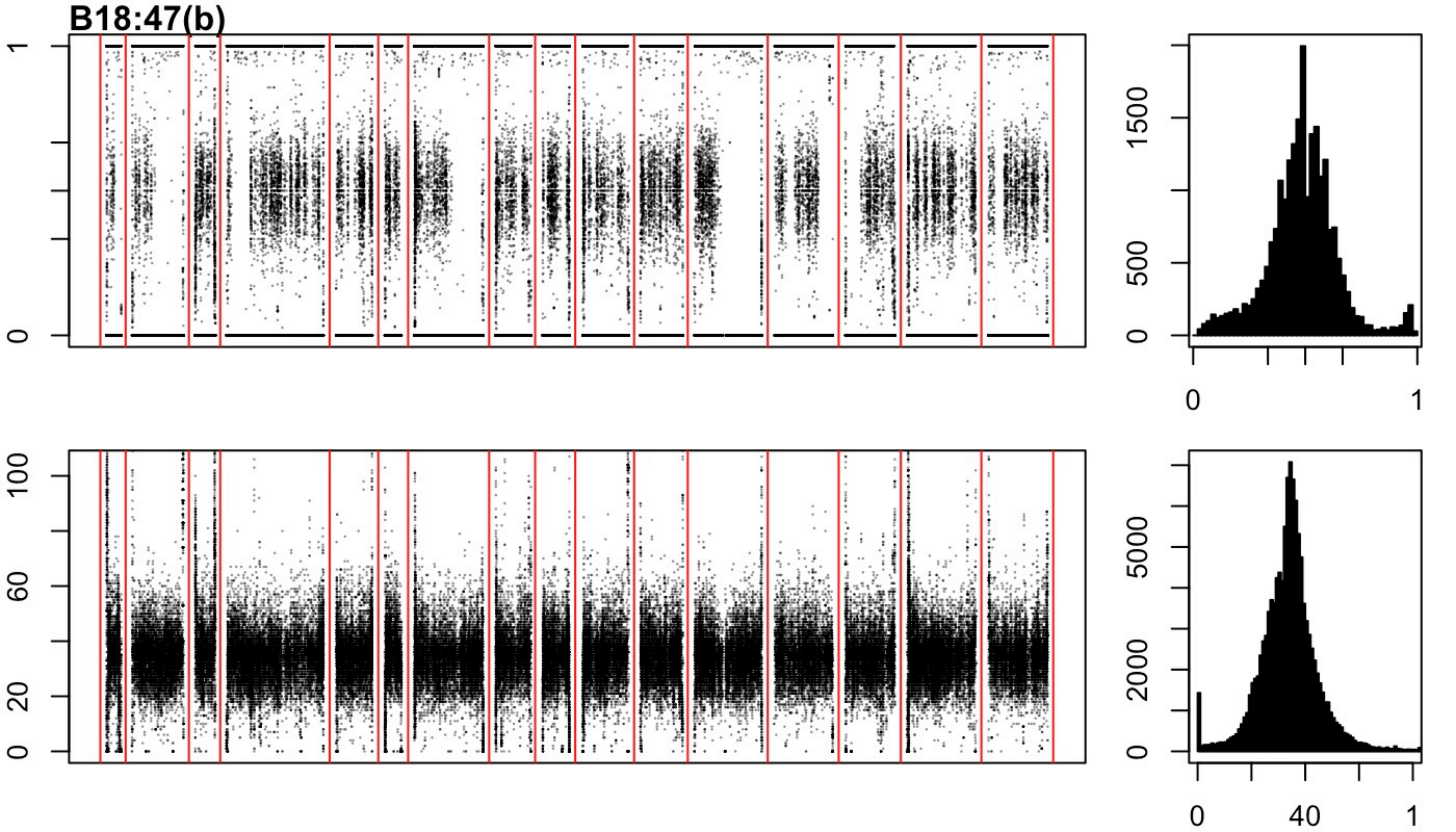
B18:47(b)
1
0
1500
500
0
0
1
100
60
20
0
5000
2000
0
0
40
1

B18:47(c)

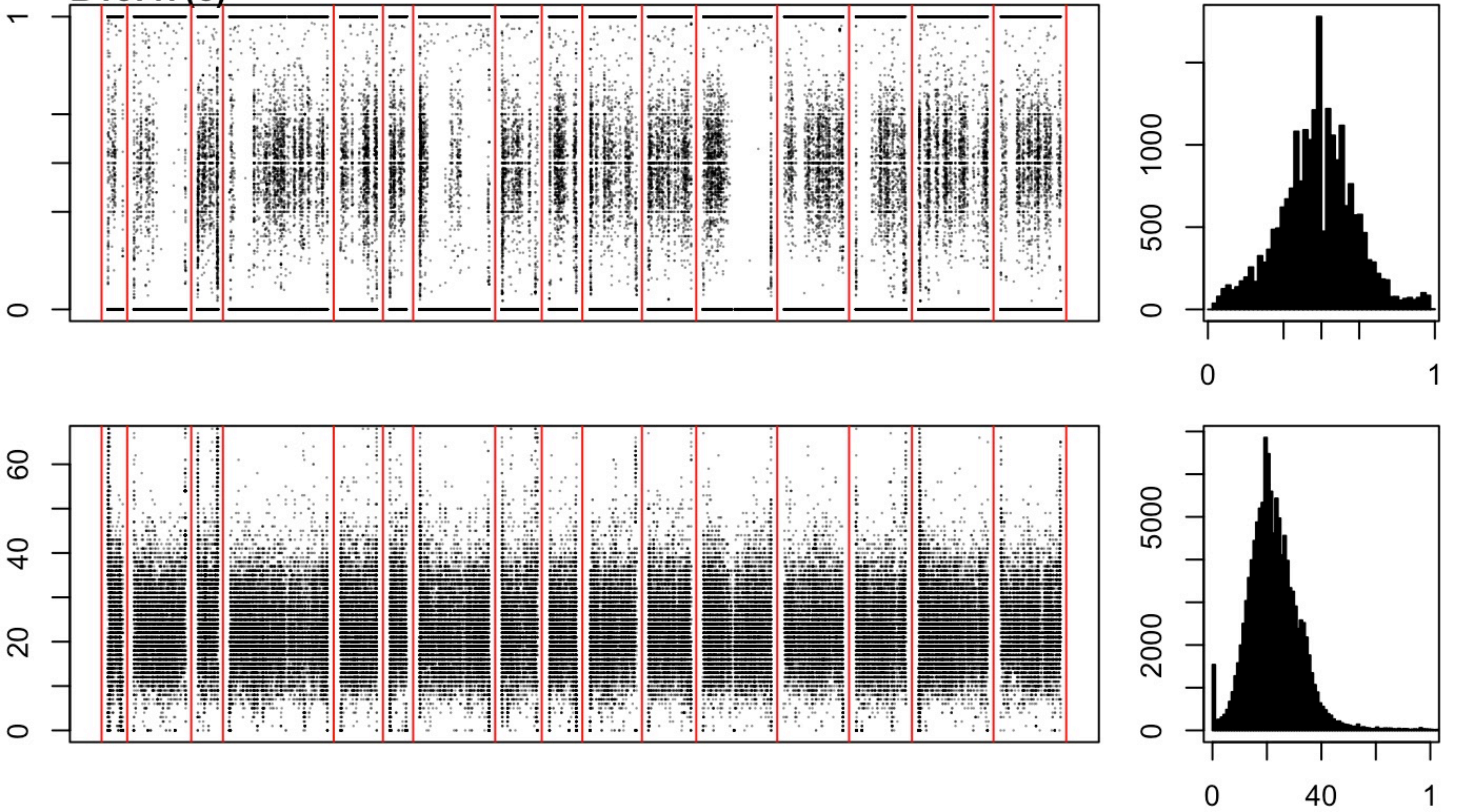

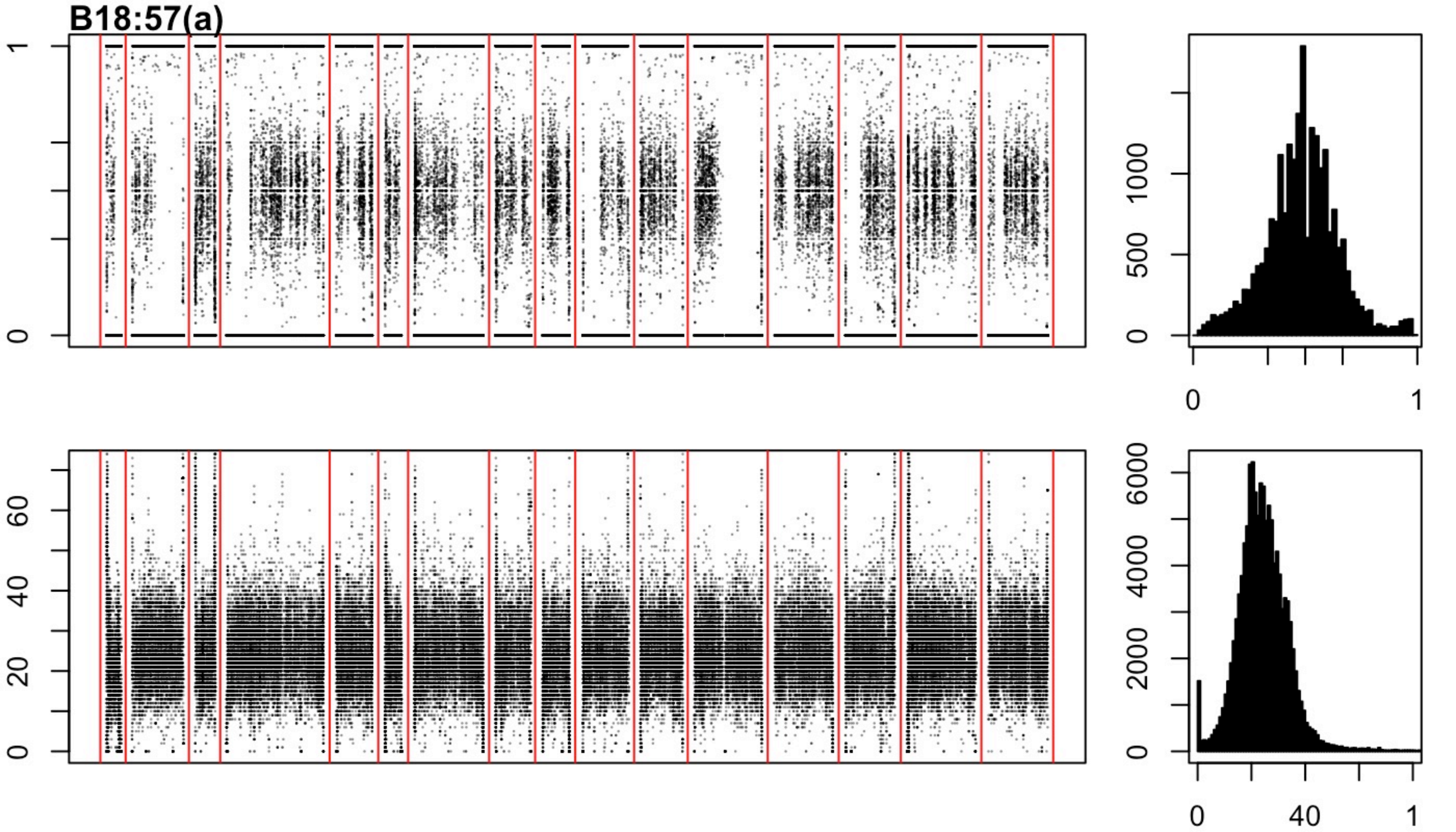
B18:57(a)

B18:57(b)

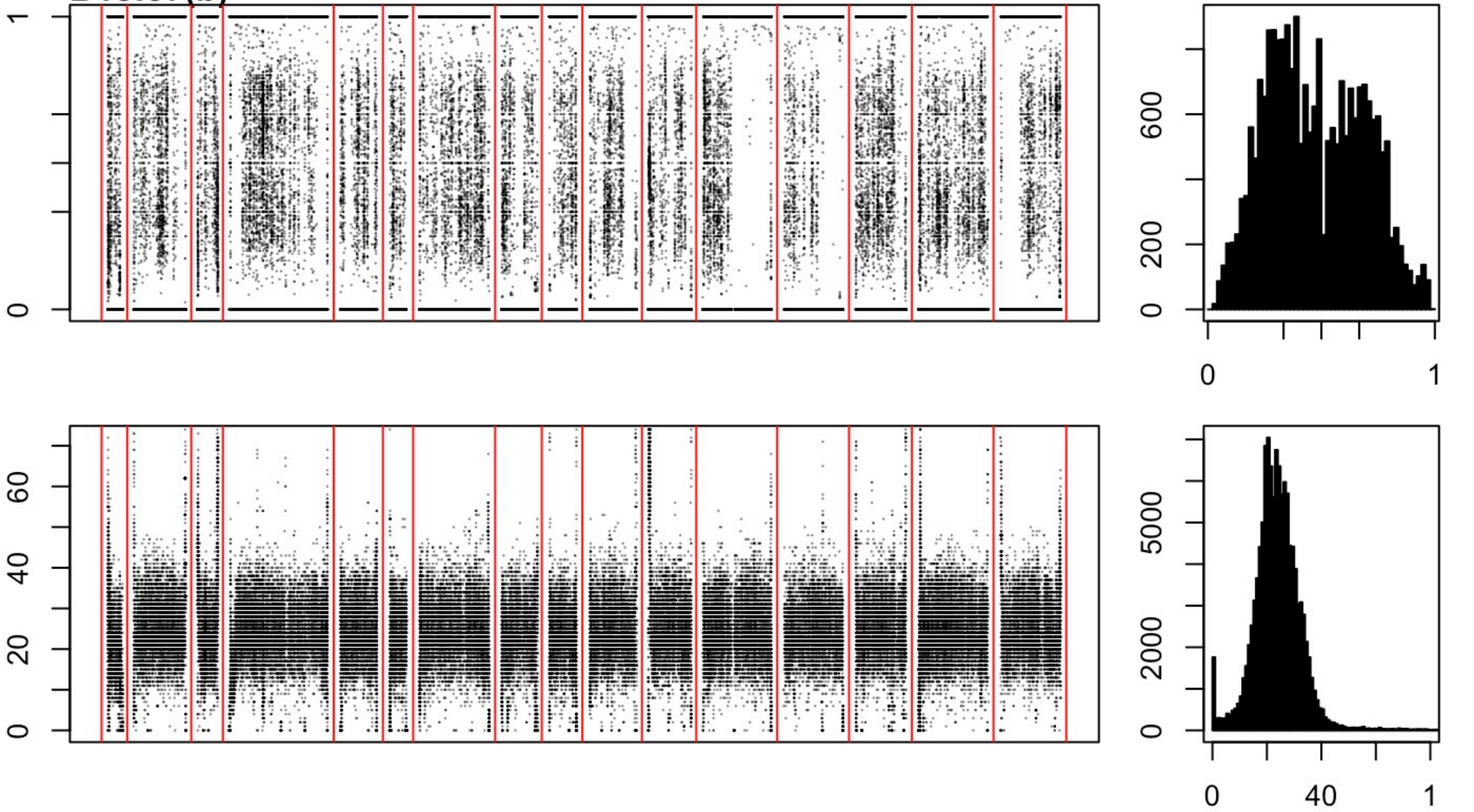

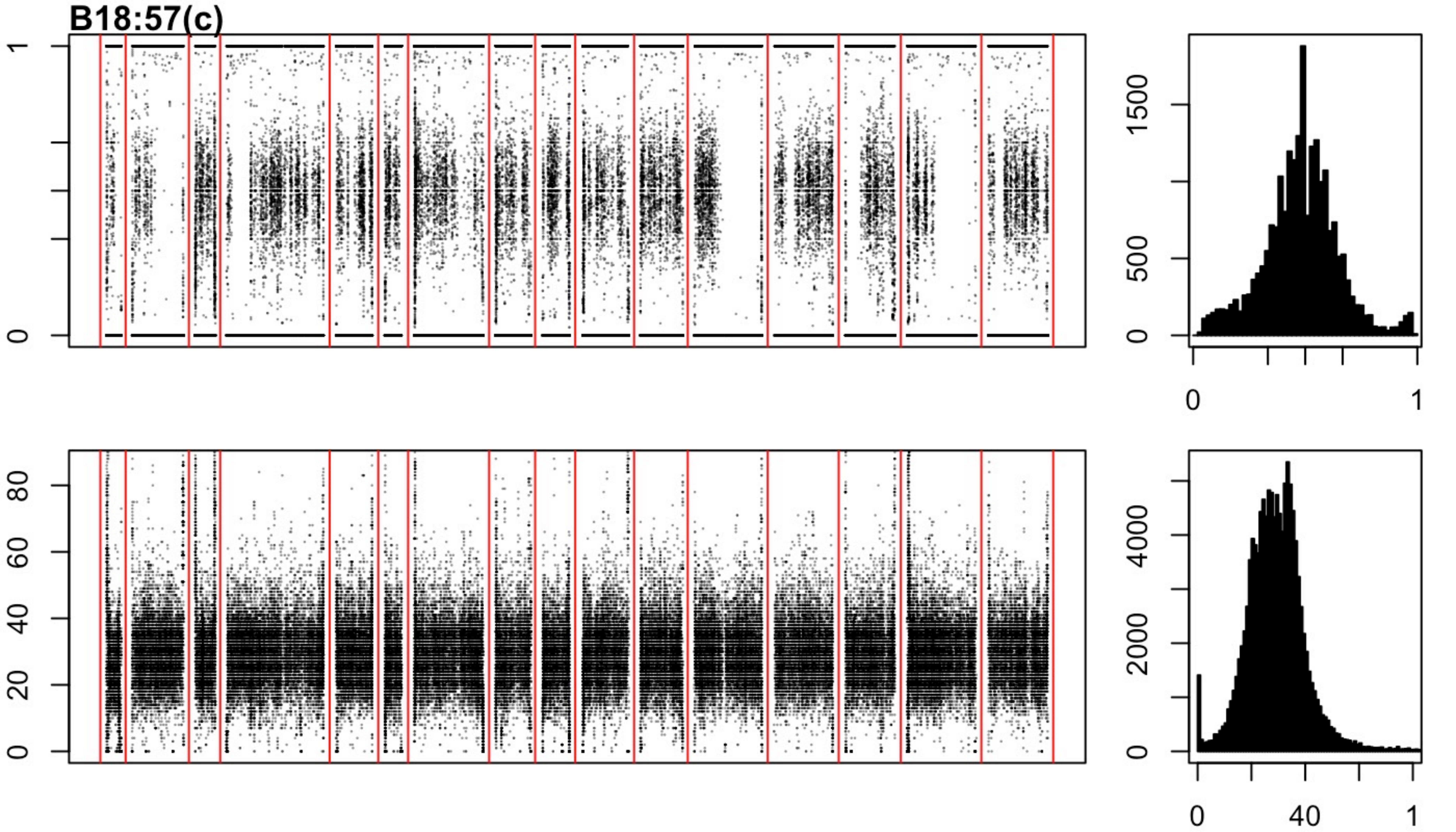
B18:57(c)
1
0
1500
500
0
0
1
80
60
40
20
0
4000
2000
0
0
40
1

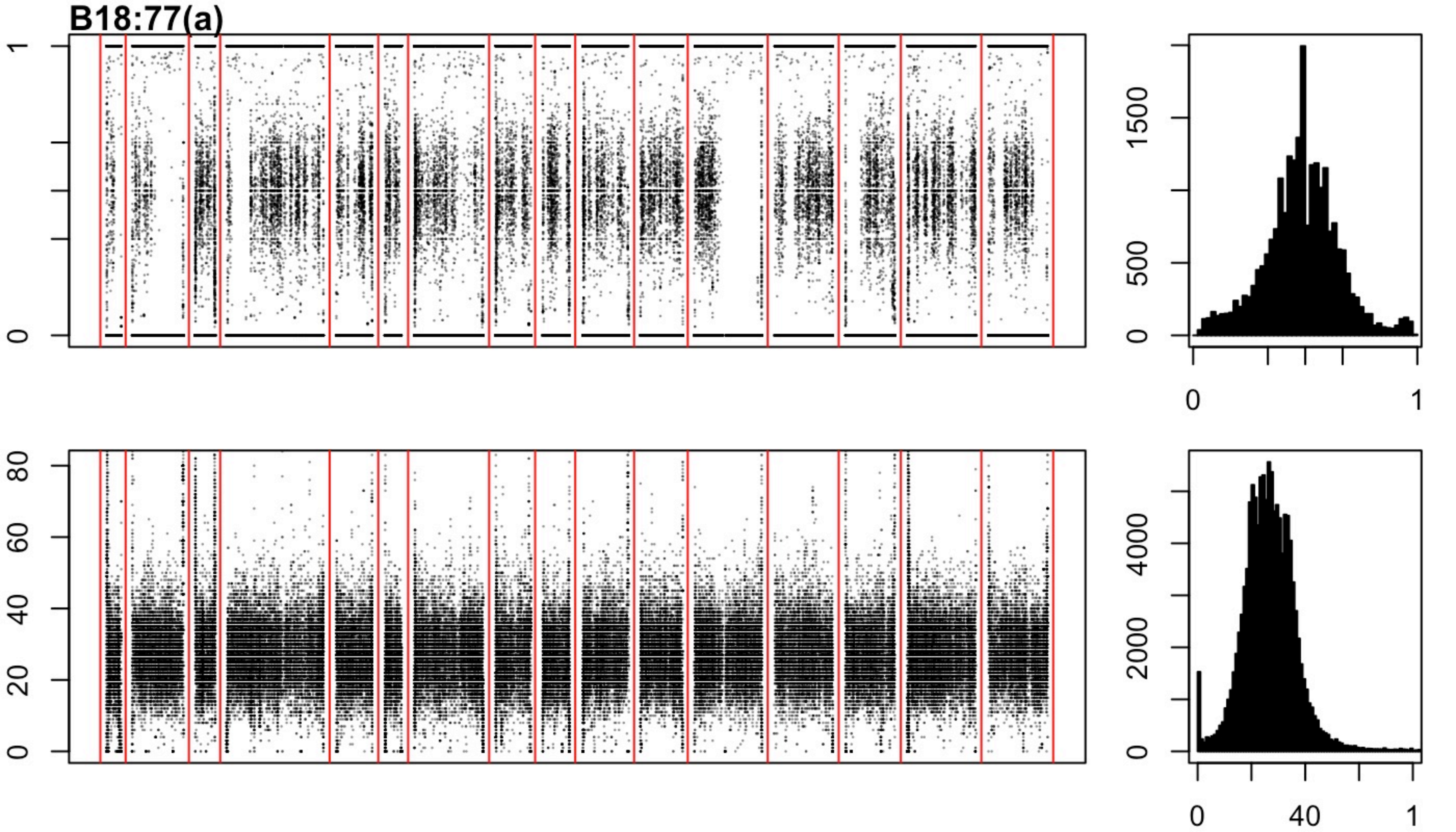

B18:77(a)
1
0
1500
500
0
0
1
80
60
40
20
0
4000
2000
0
0
40
1

B18:77(b)

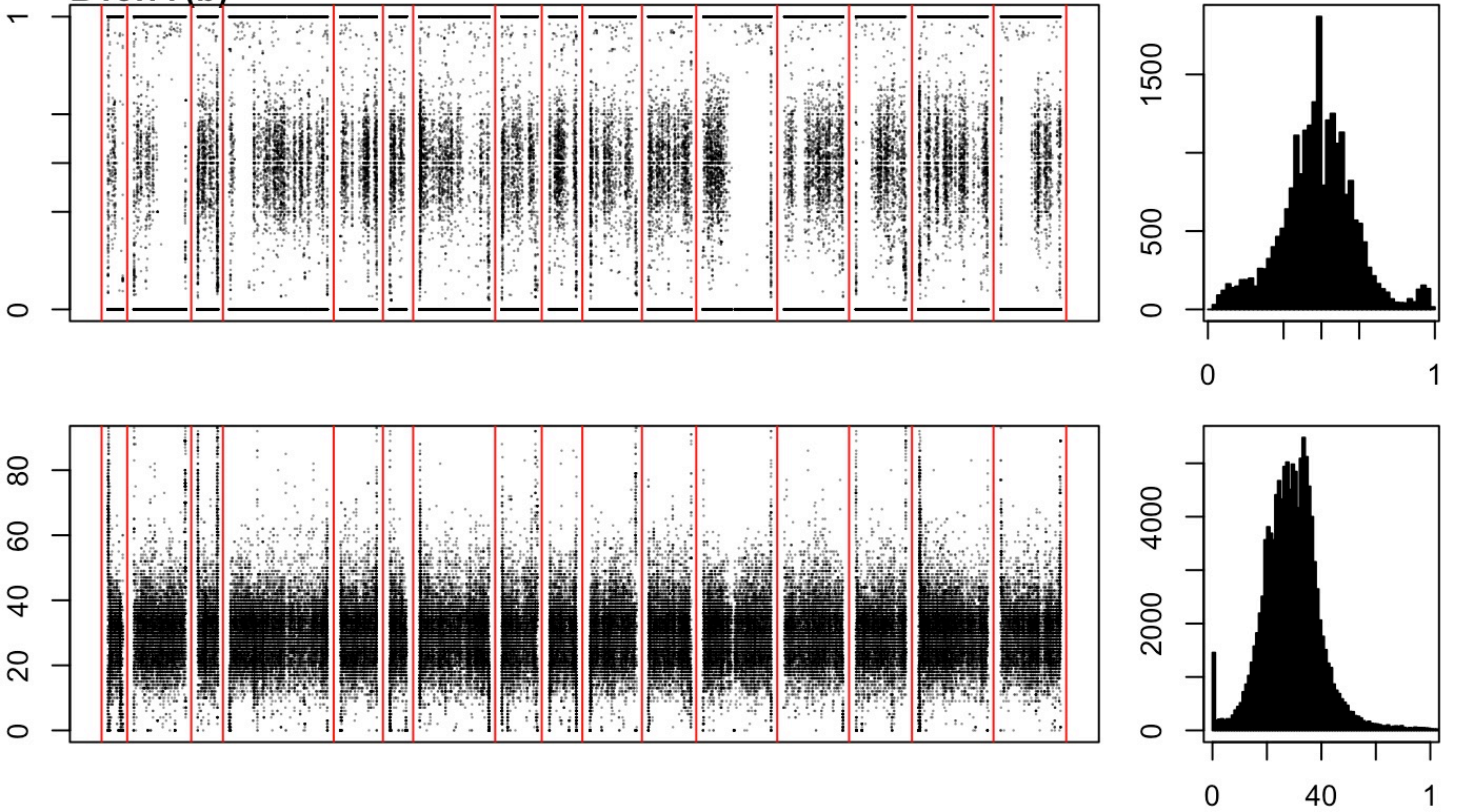

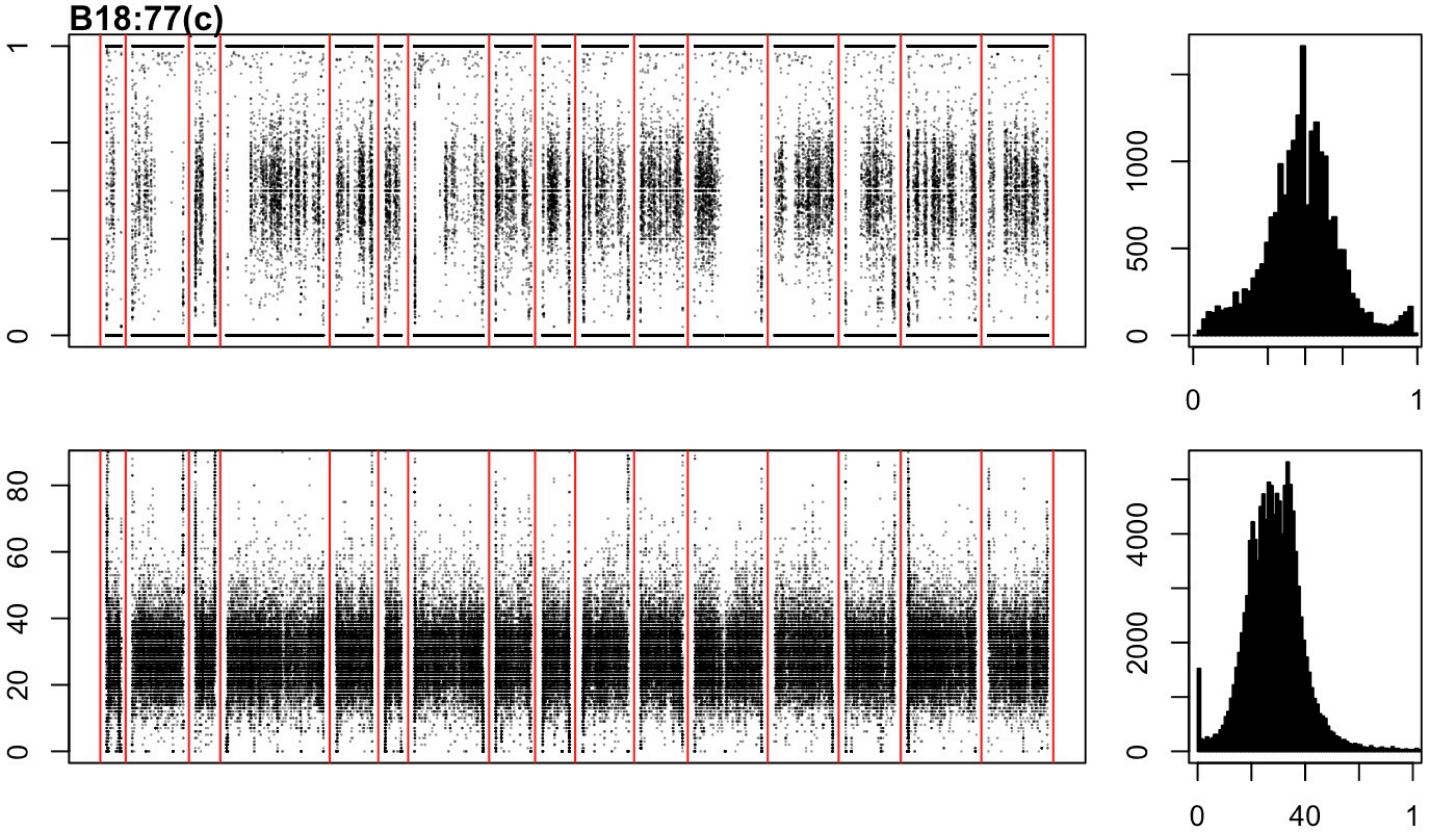
B18:77(c)
1
0
80
60
40
20
0
1000
500
0
0
1
4000
2000
0
0
40
1

B18:87(a)

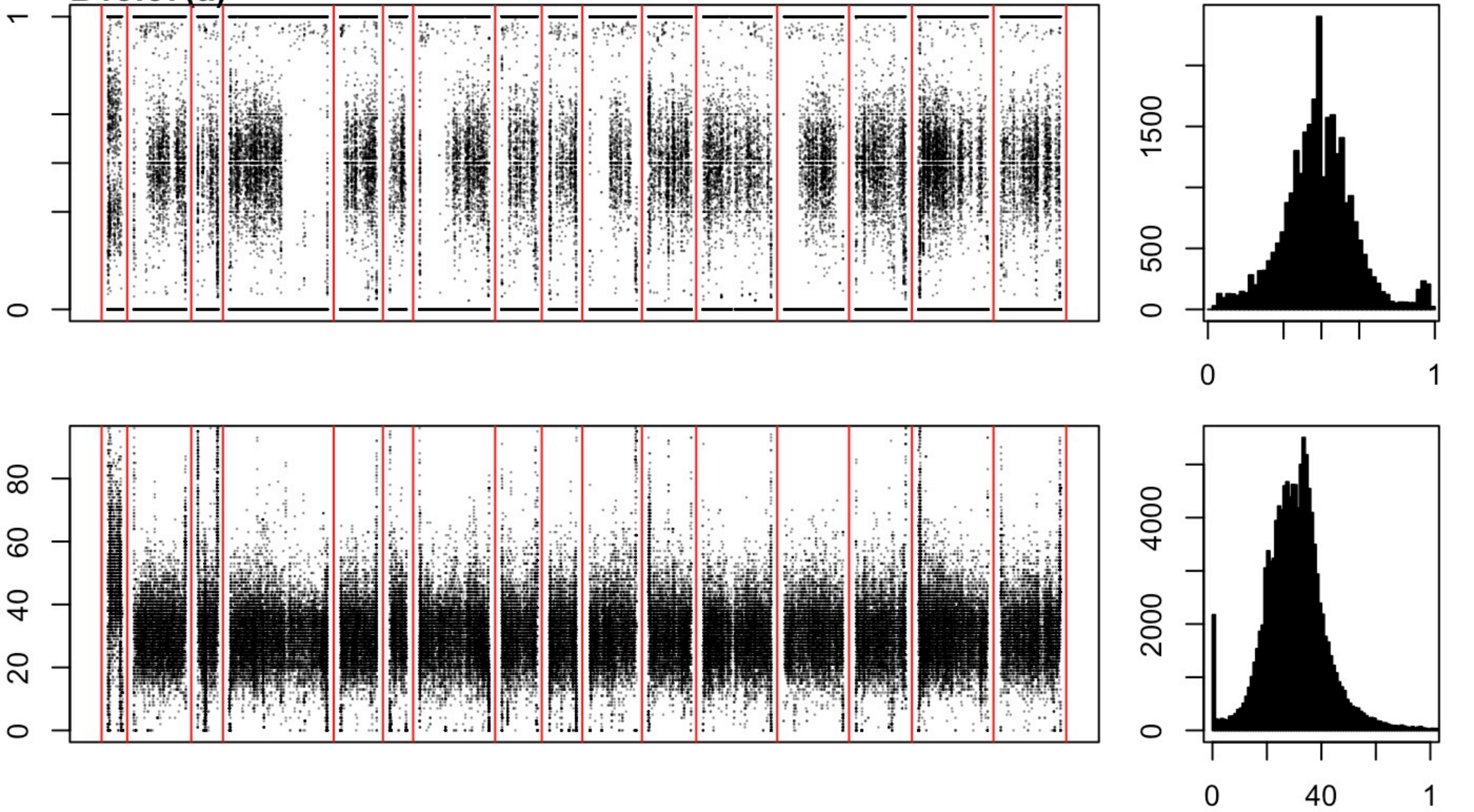

B18:87(b)

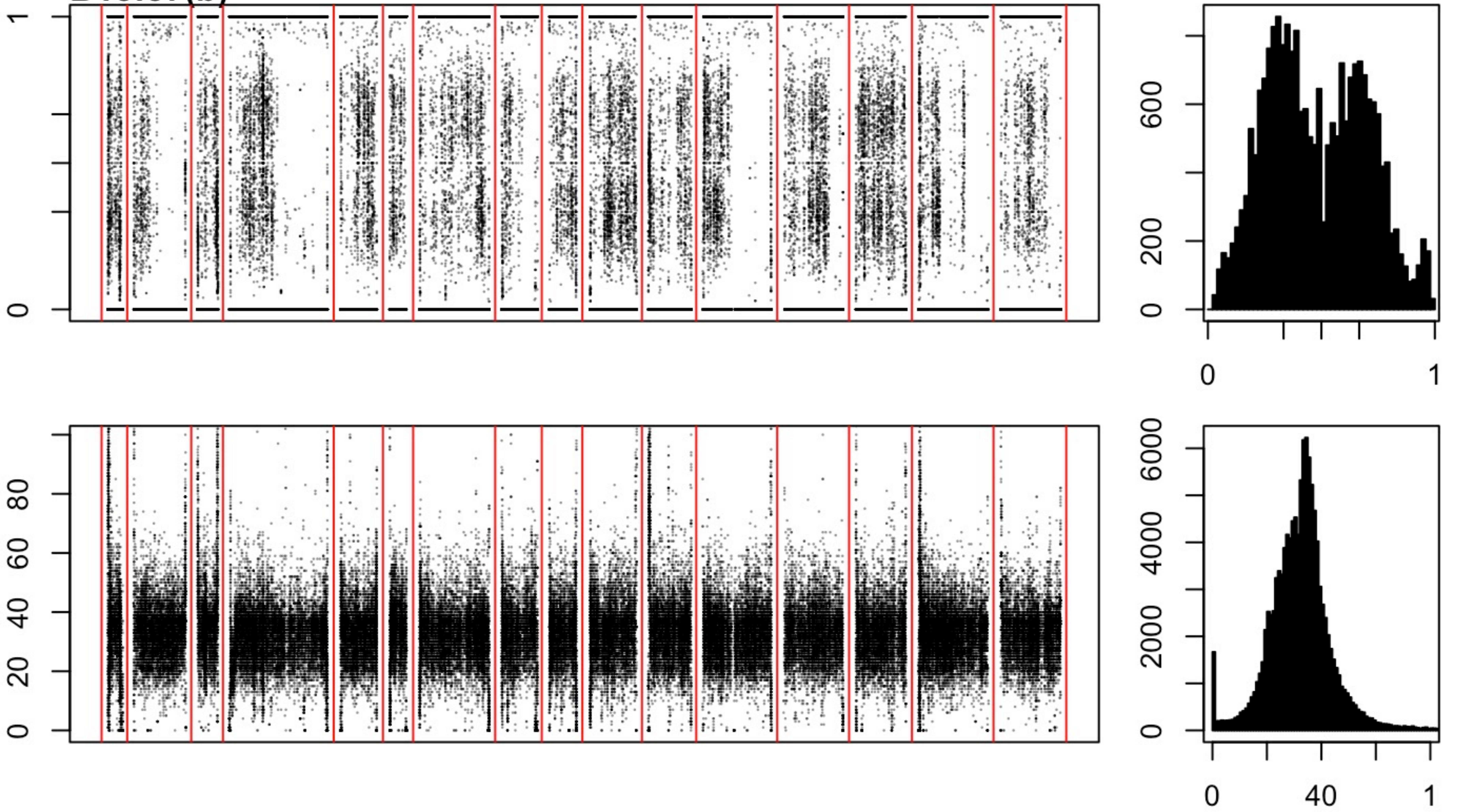

B18:87(c)

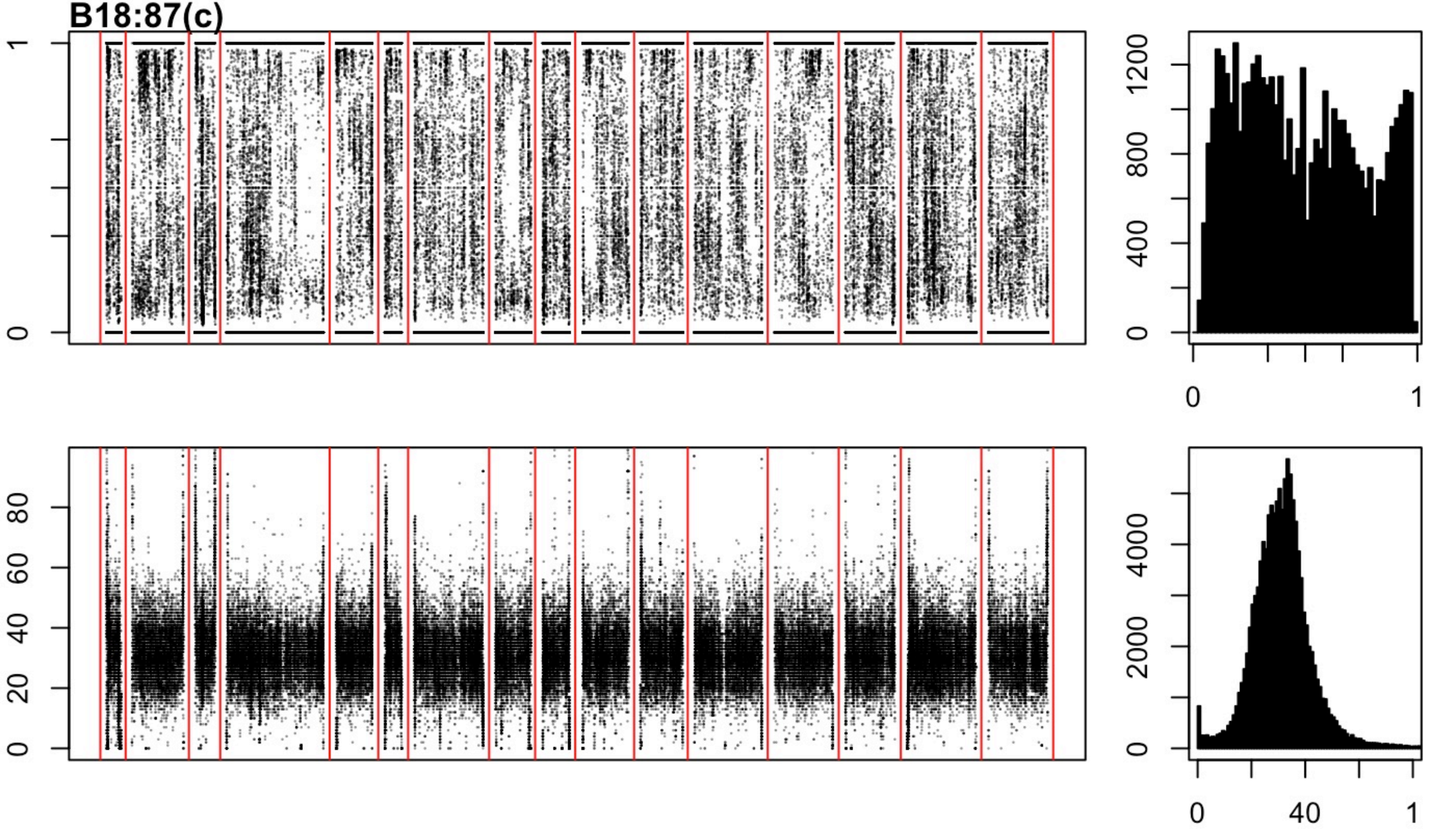

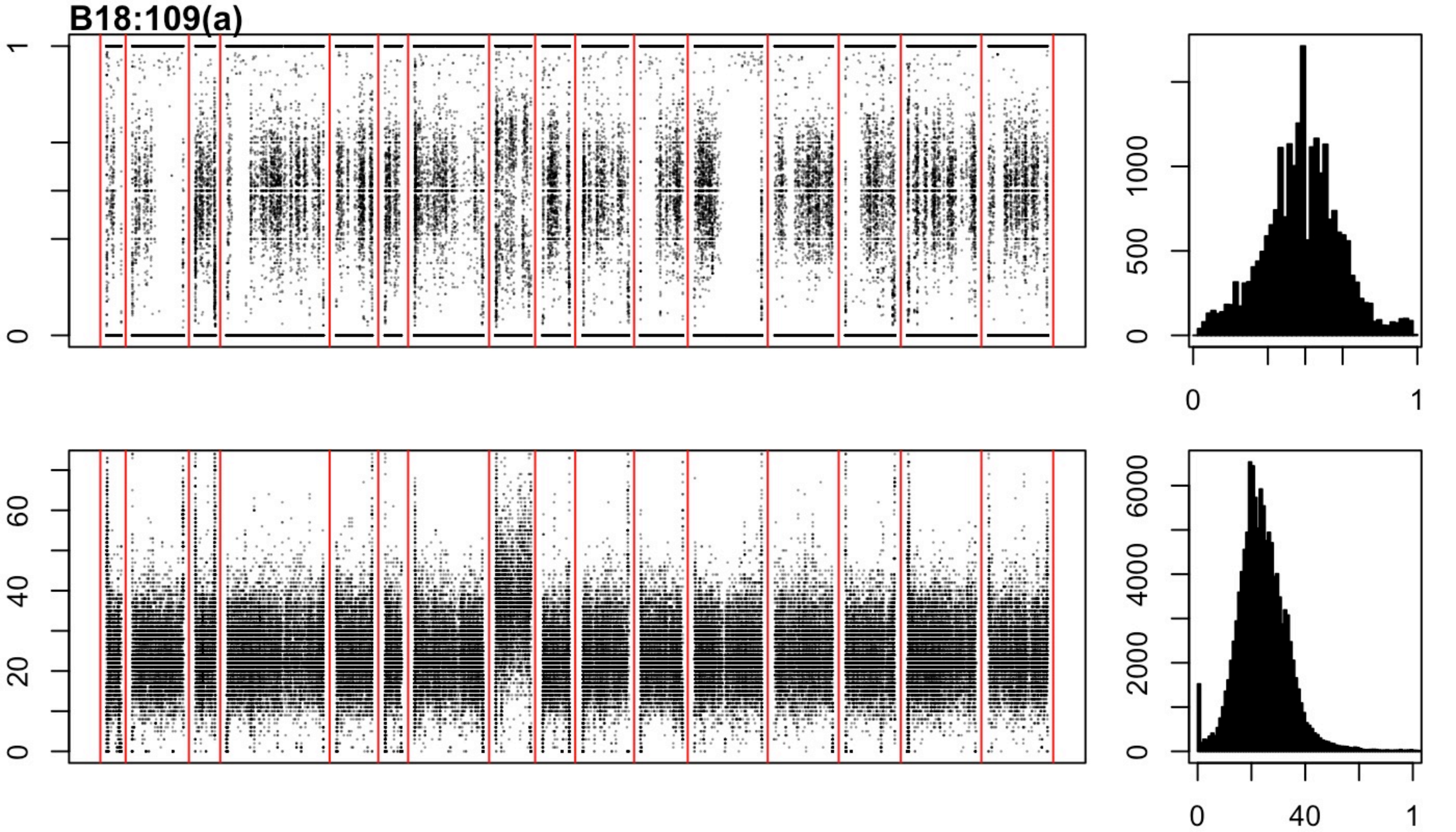

B18:109(a)
1
0
1000
500
0
0
1
60
40
20
0
6000
4000
2000
0
0
40
1

B18:109(b)

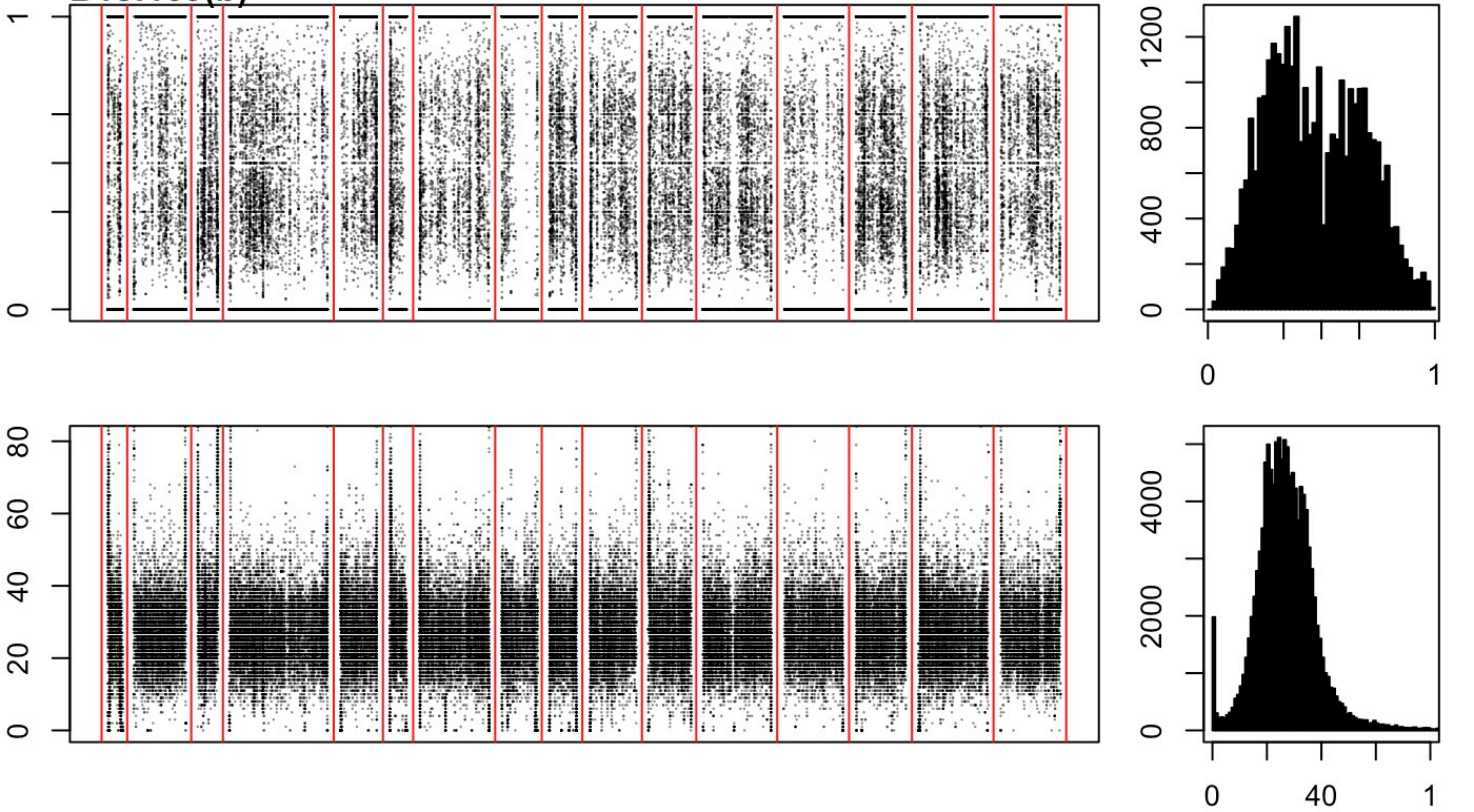

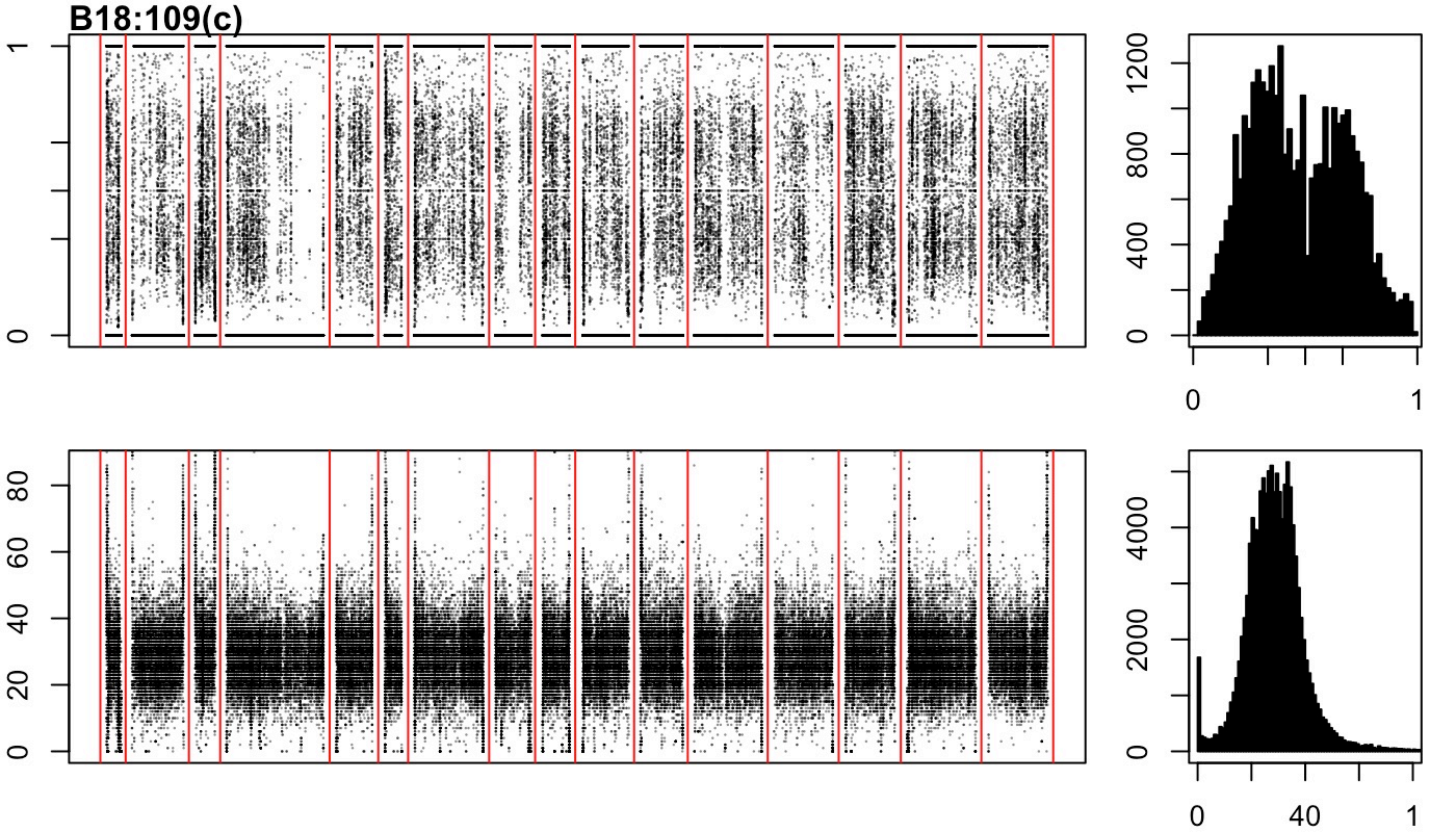

B18:109(c)

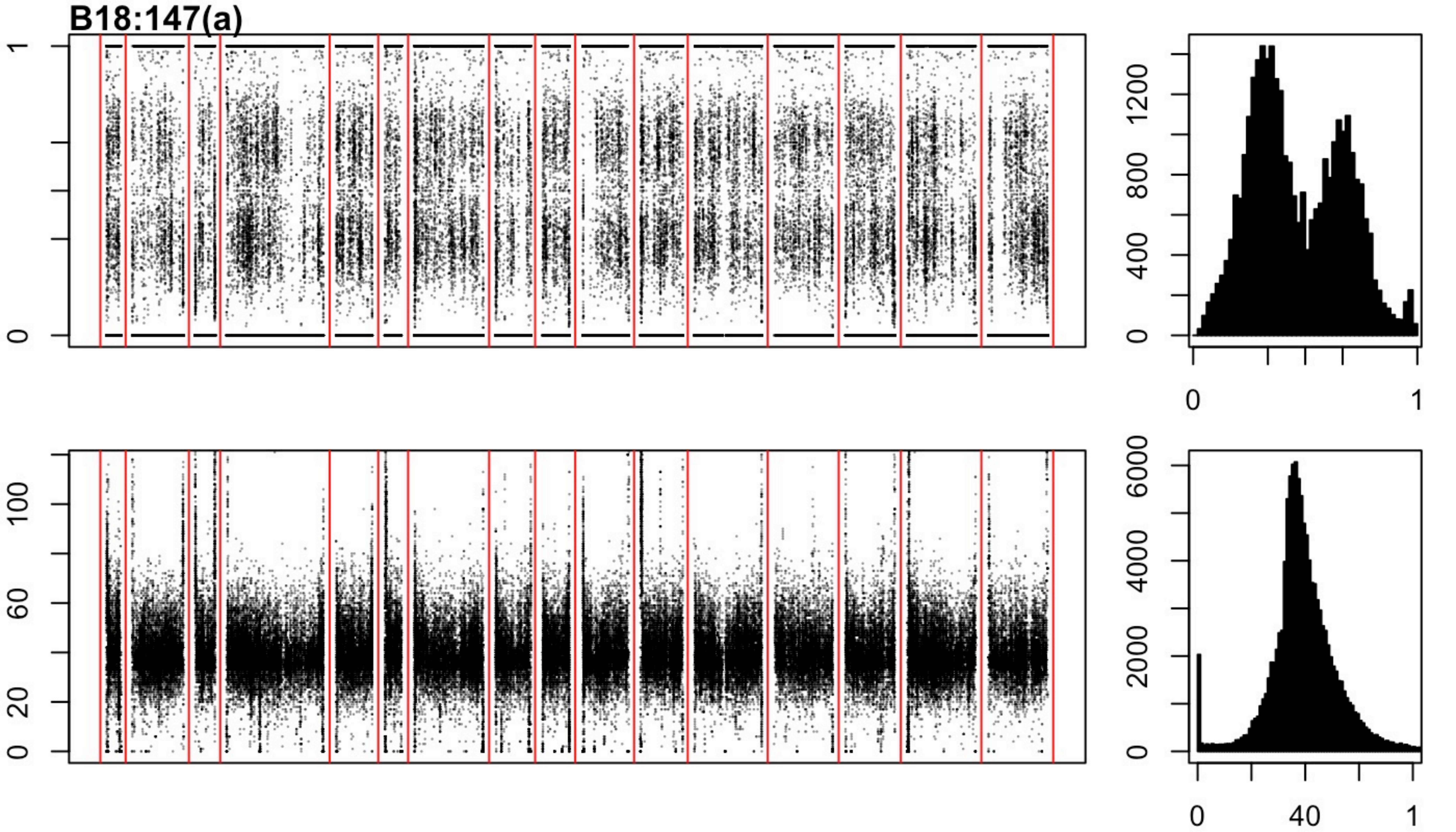
B18:147(a)
1
0
1200
800
400
0
0
1
100
60
20
0
6000
4000
2000
0
0
40
1

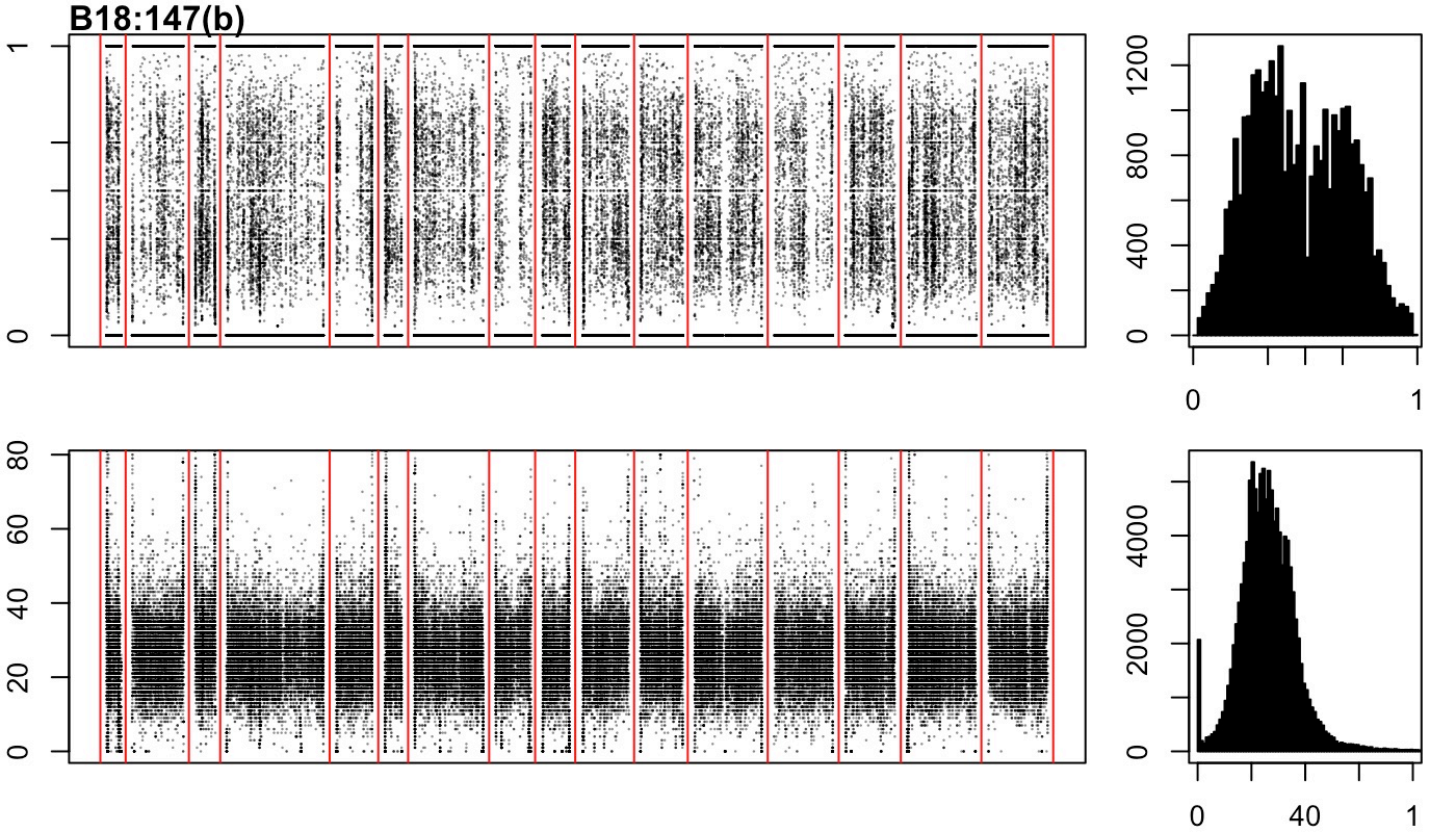
B18:147(b)
1
0
1200
800
400
0
0
1
80
60
40
20
0
4000
2000
0
0
40
1

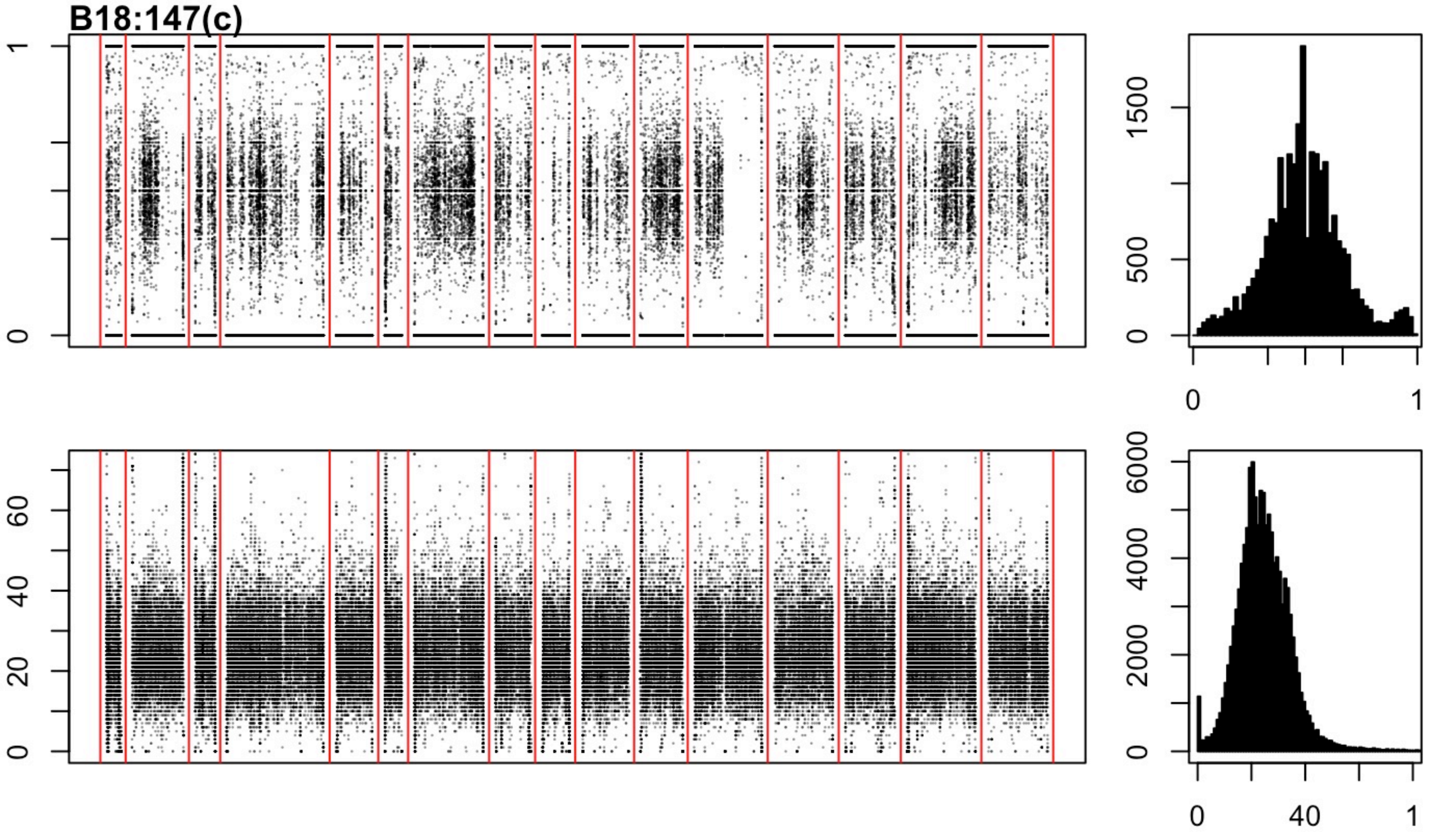
B18:147(c)
1
0
1500
500
0
0
1
60
40
20
0
6000
4000
2000
0
0
40
1

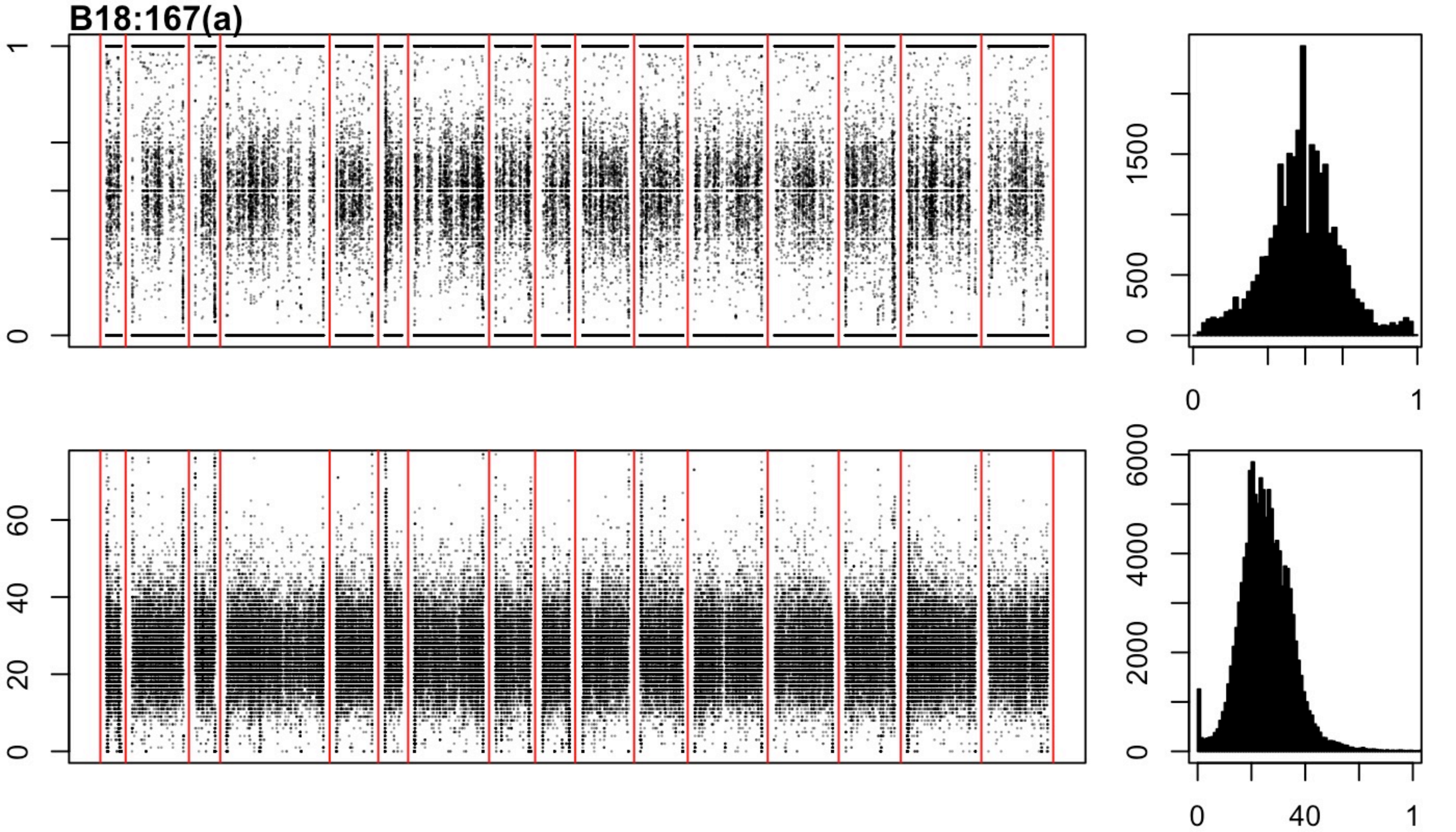
B18:167(a)
1
0
1500
500
0
0
1
60
40
20
0
6000
4000
2000
0
0
40
1

B18:167(b)

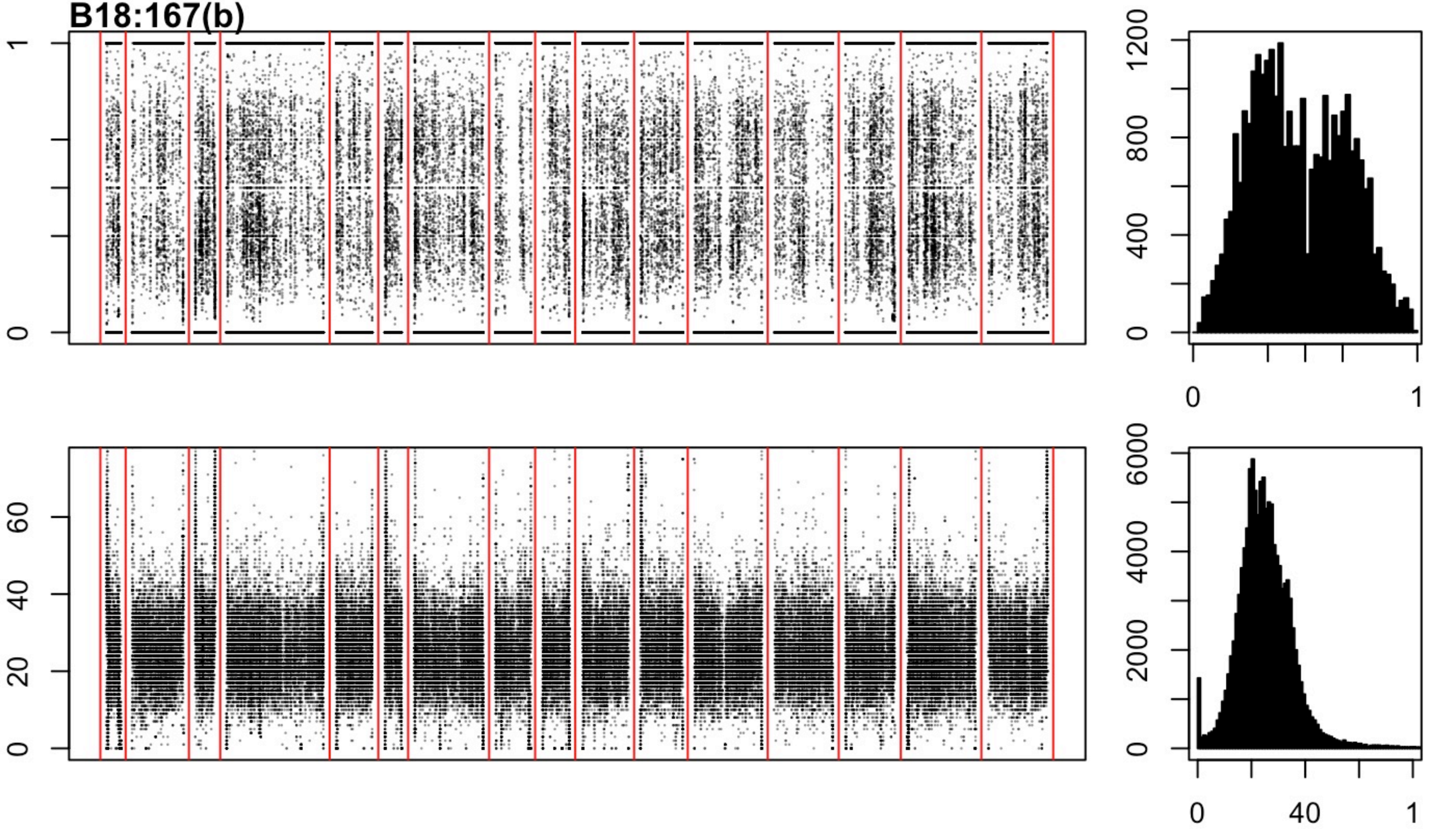

B18:167(c)

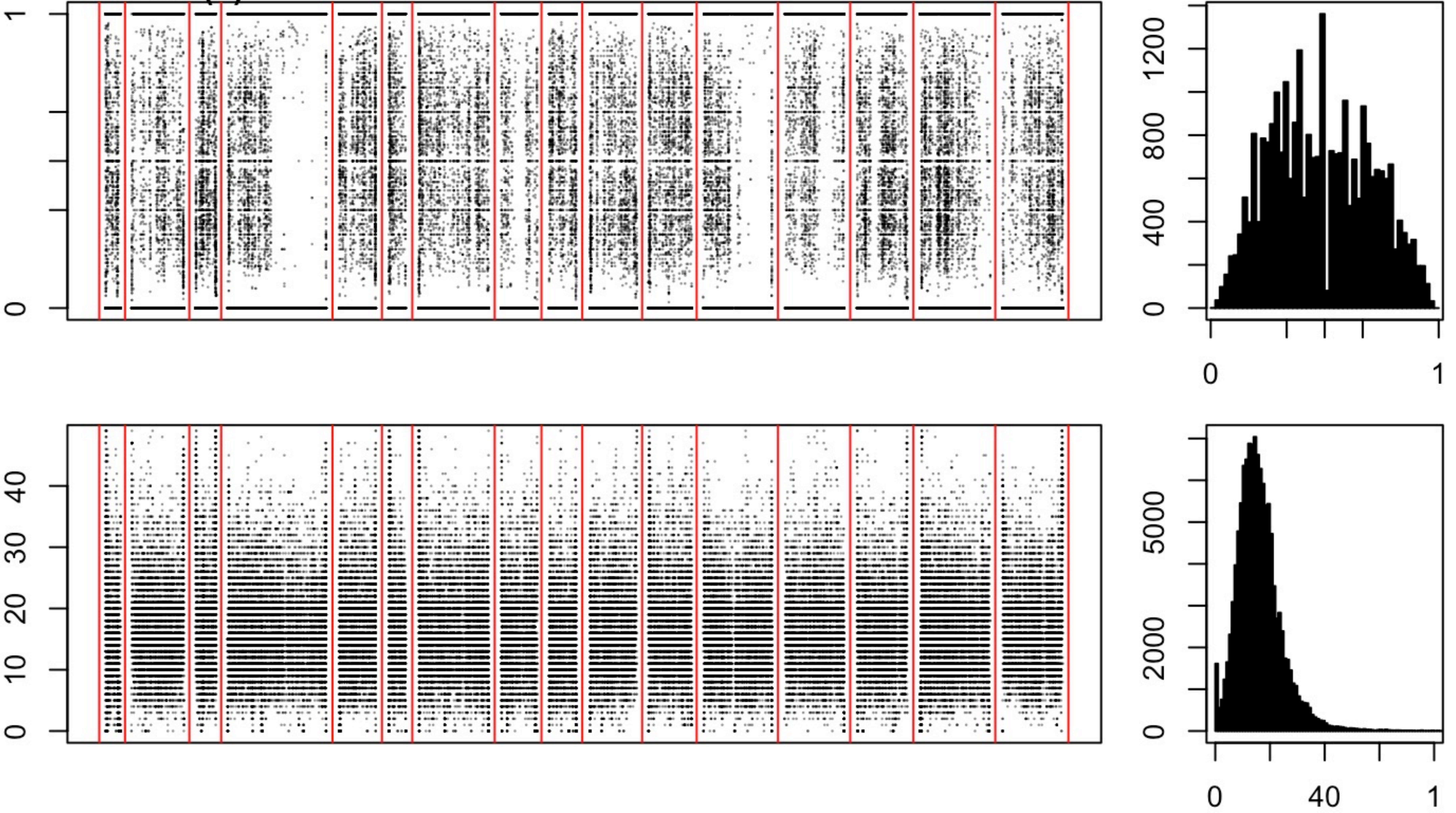

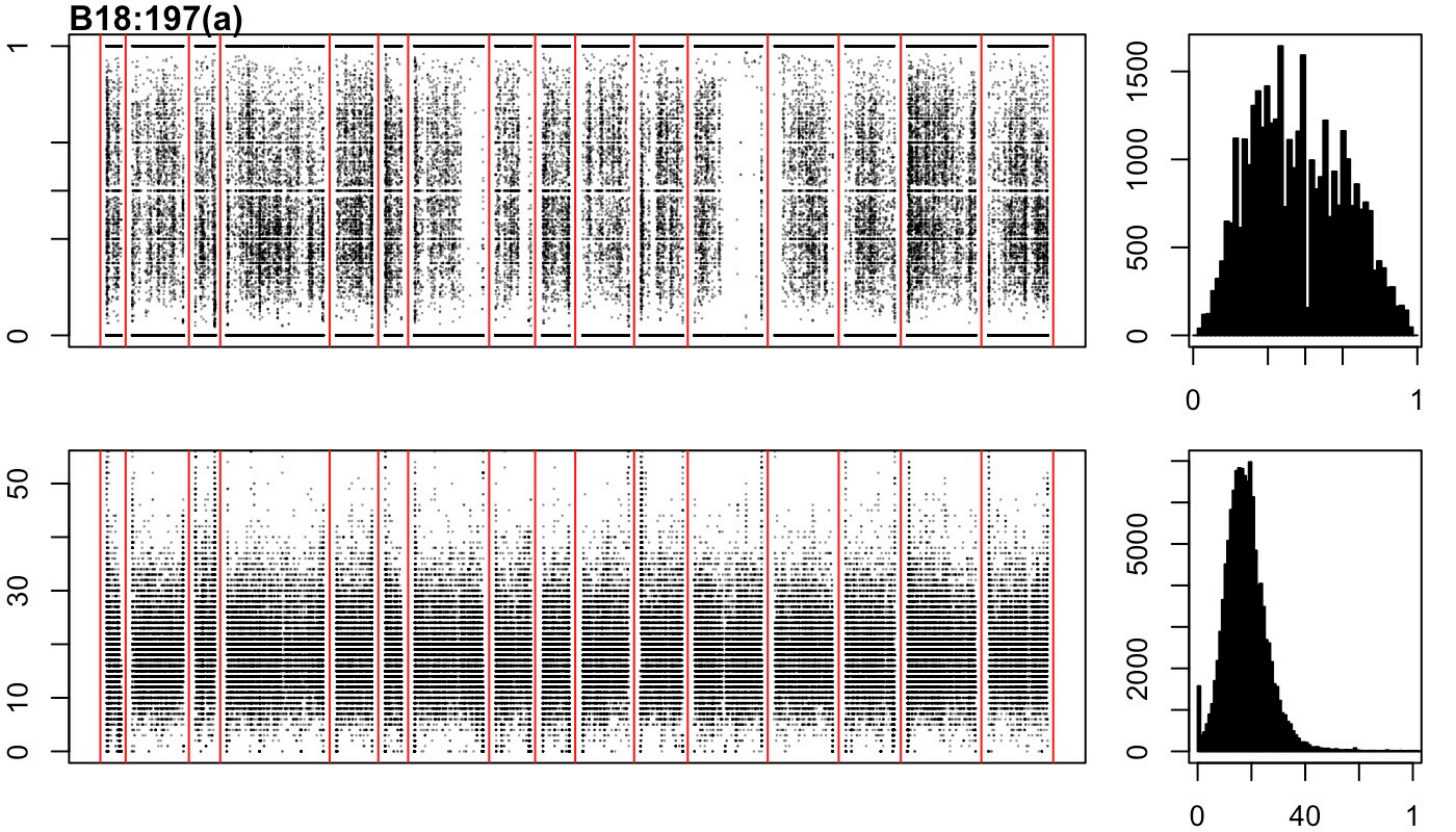

B18:197(a)
1
0
1500
1000
500
0
0
1
50
30
10
0
5000
2000
0
0
40
1

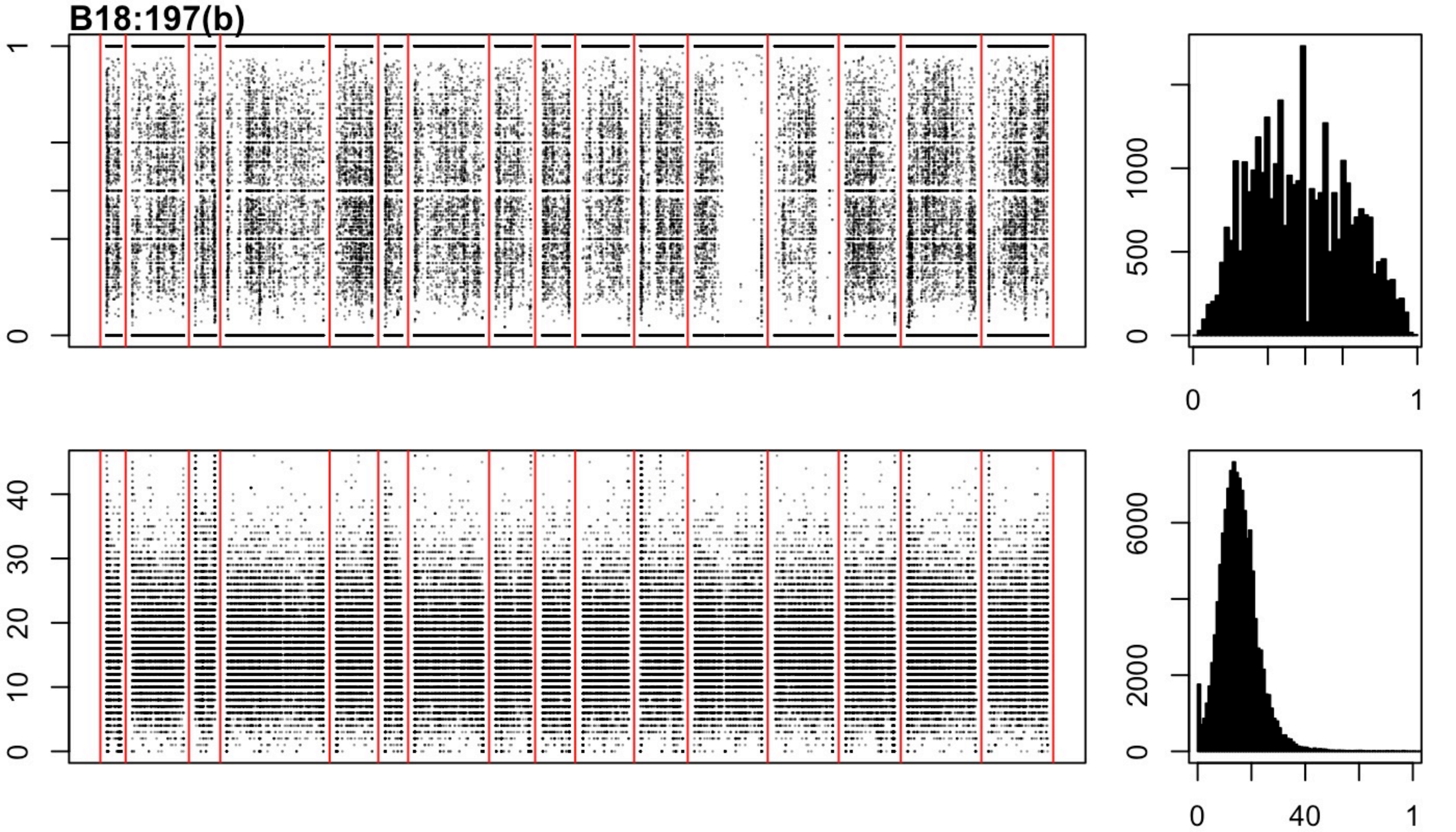
B18:197(b)
1
0
1000
500
0
0
1
40
30
20
10
0
6000
2000
0
0
40
1

B18:197(c)

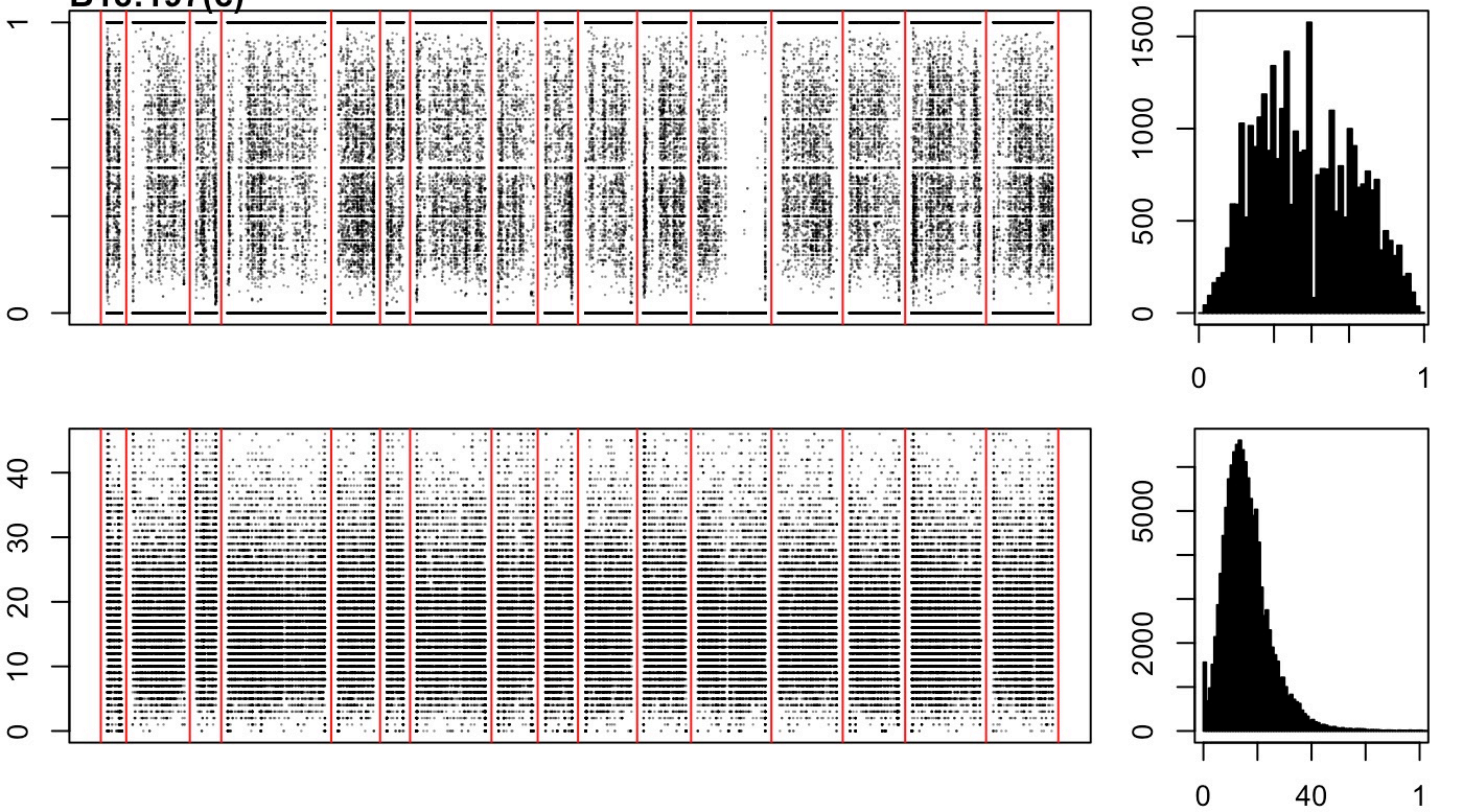

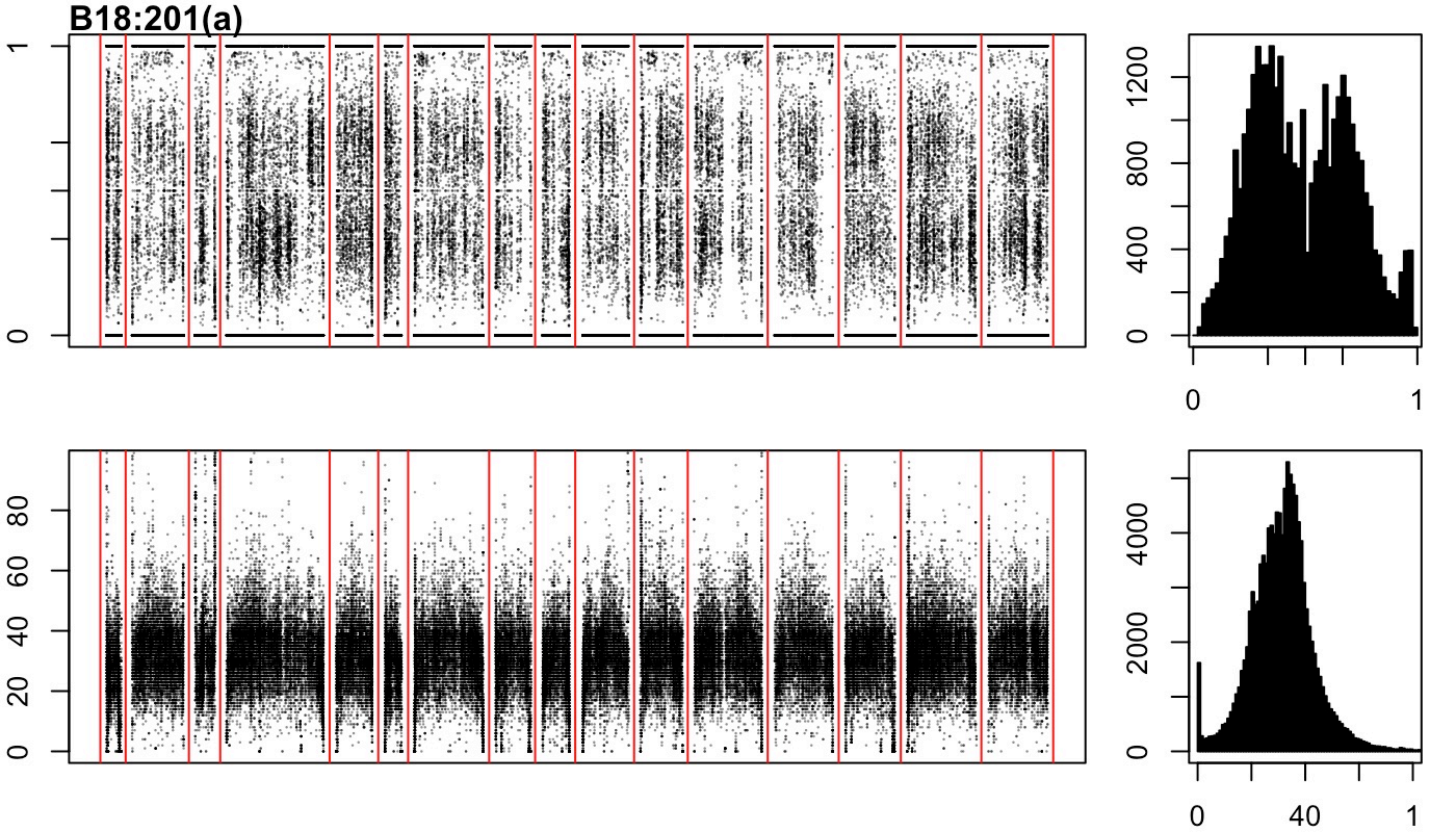

B18:201(a)
1
0
1200
800
400
0
0
1
80
60
40
20
0
4000
2000
0
0
40
1

B18:201(b)

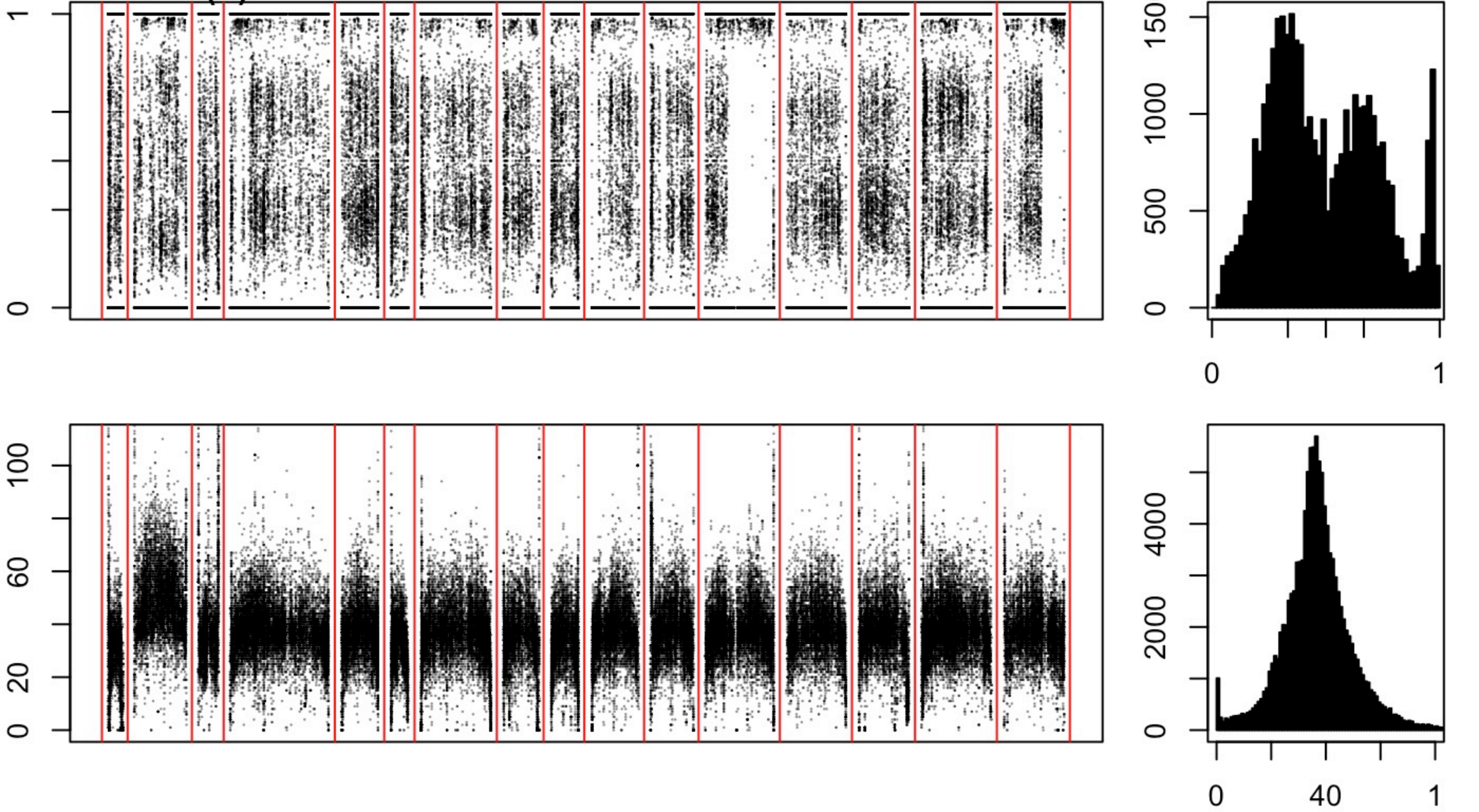

B18:203(a)

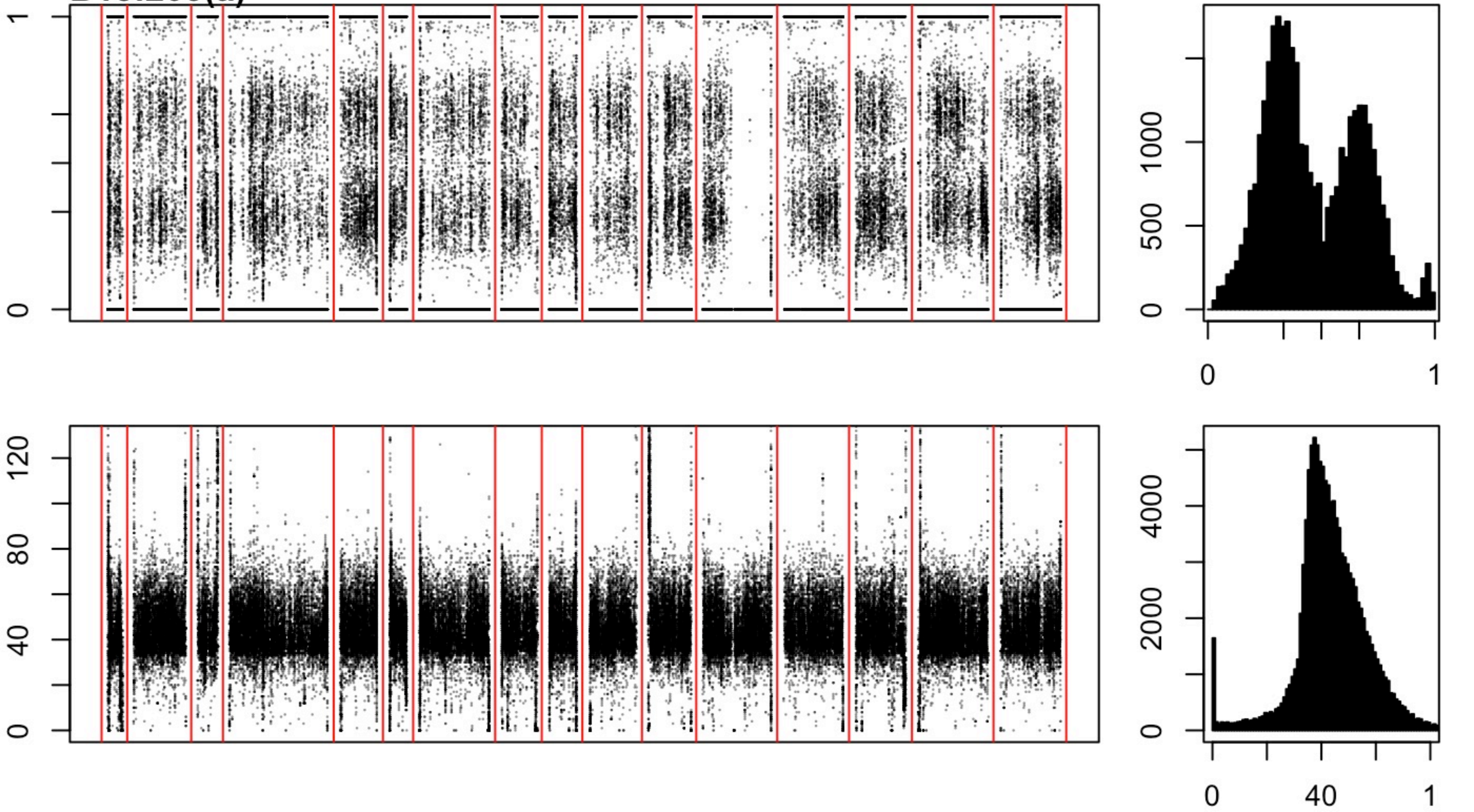

B18:203(b)

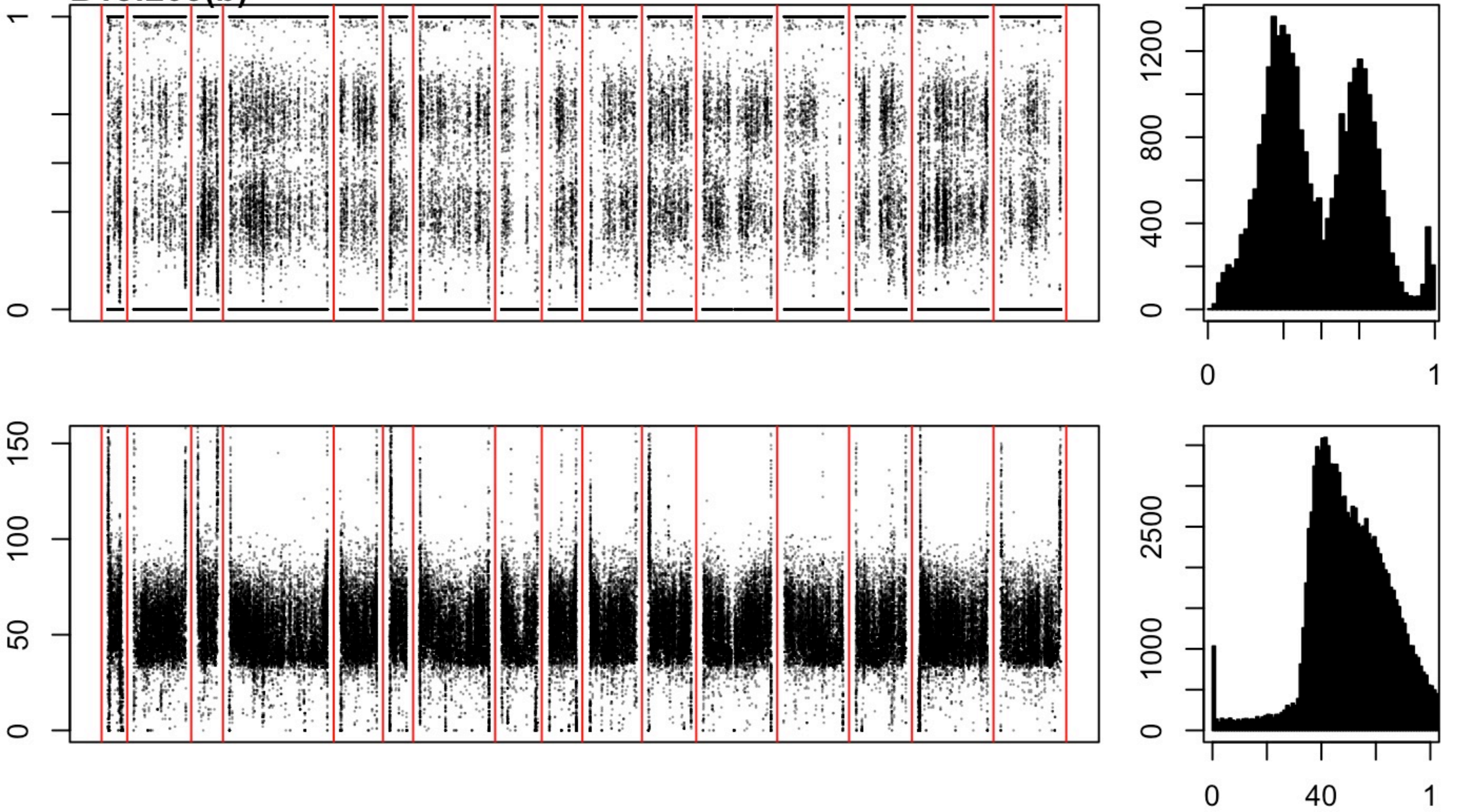

B18:203(c)

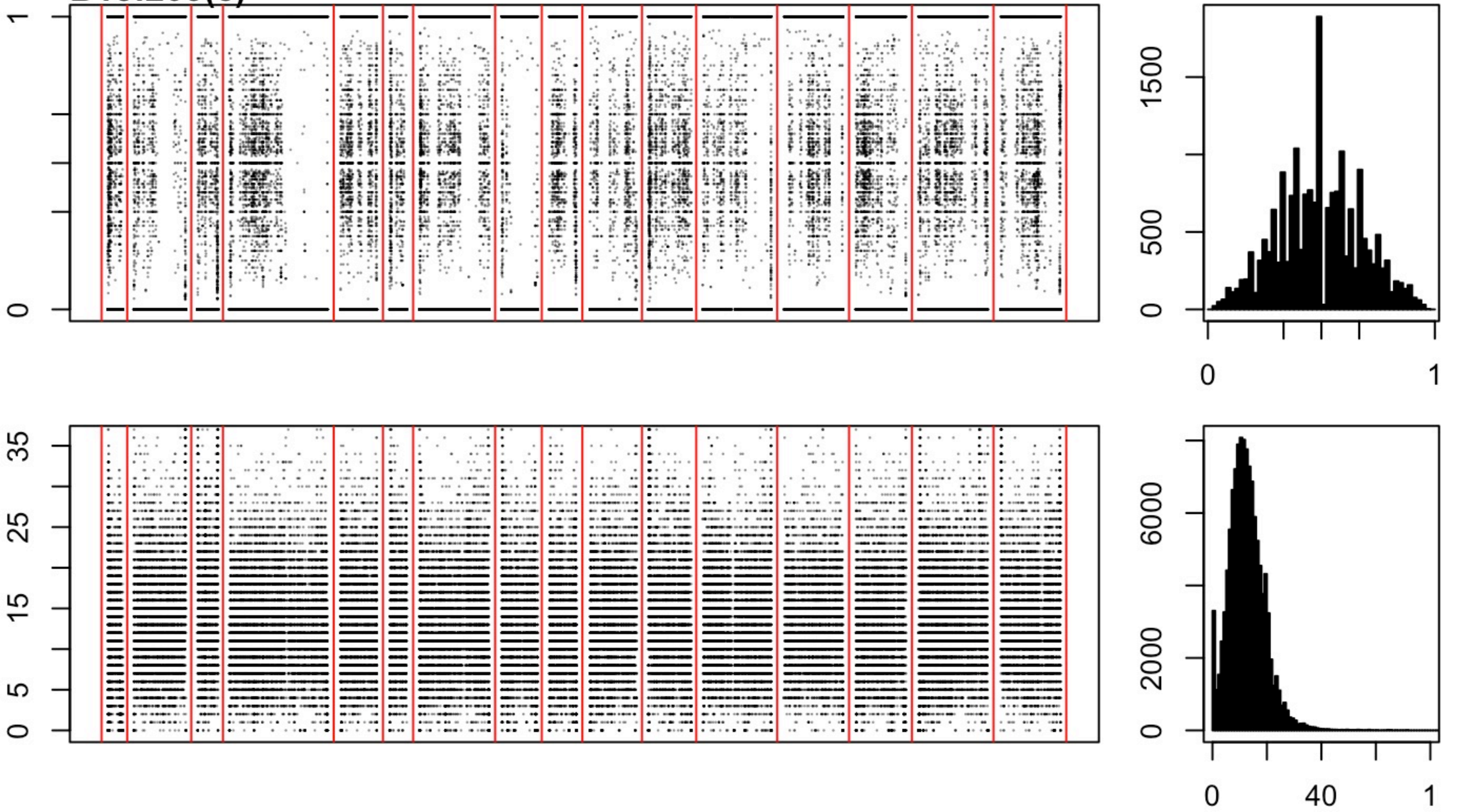

B18:27(a)

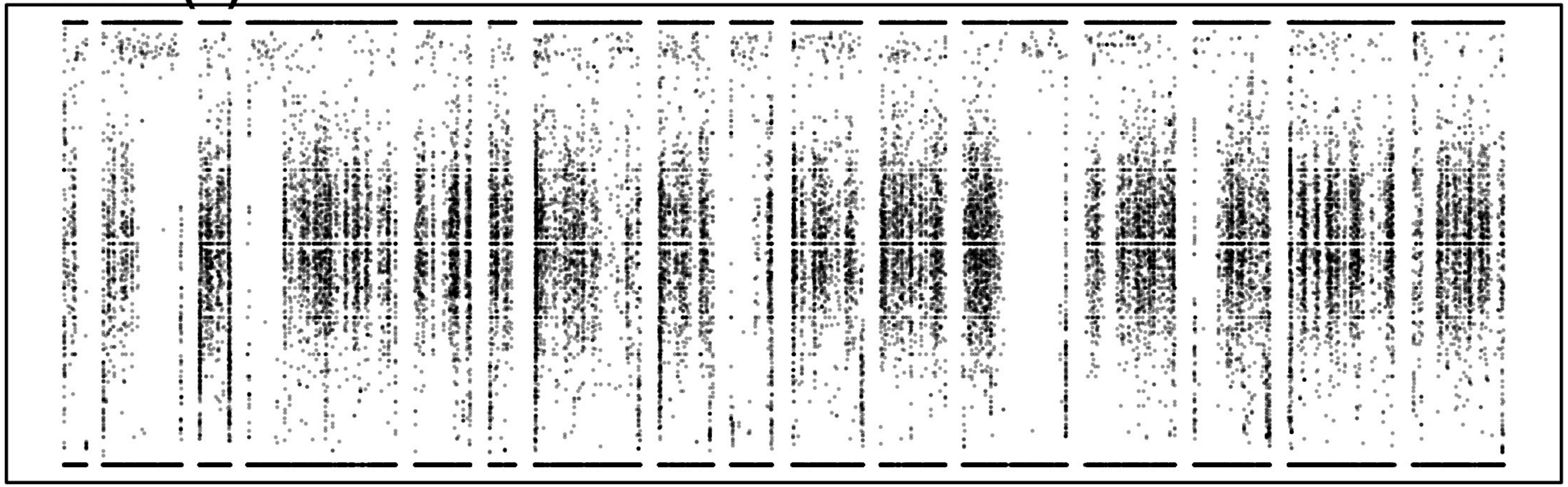

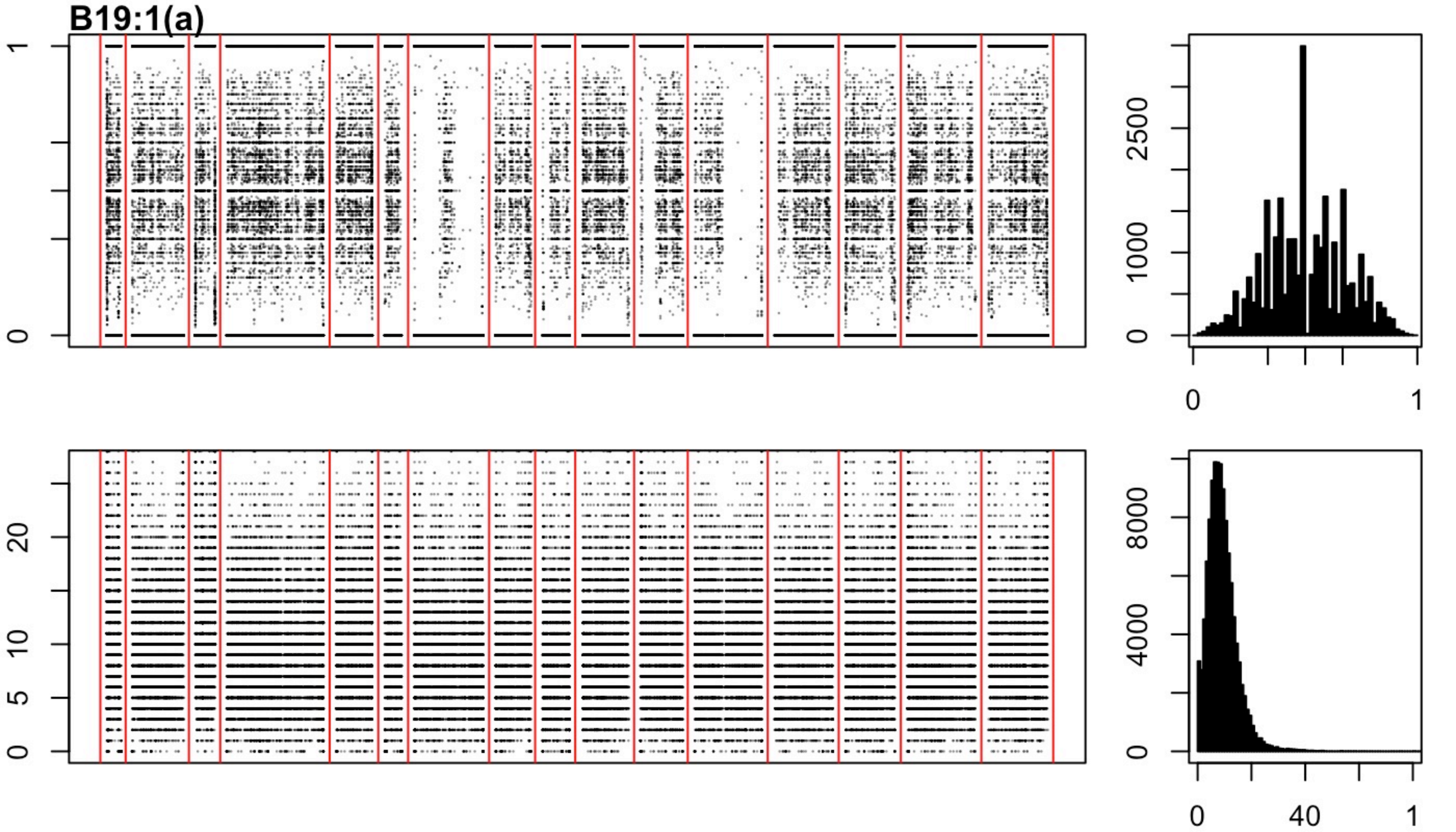
B19:1(a)
1
0
2500
1000
0
0
1
20
10
5
0
8000
4000
0
0
40
1

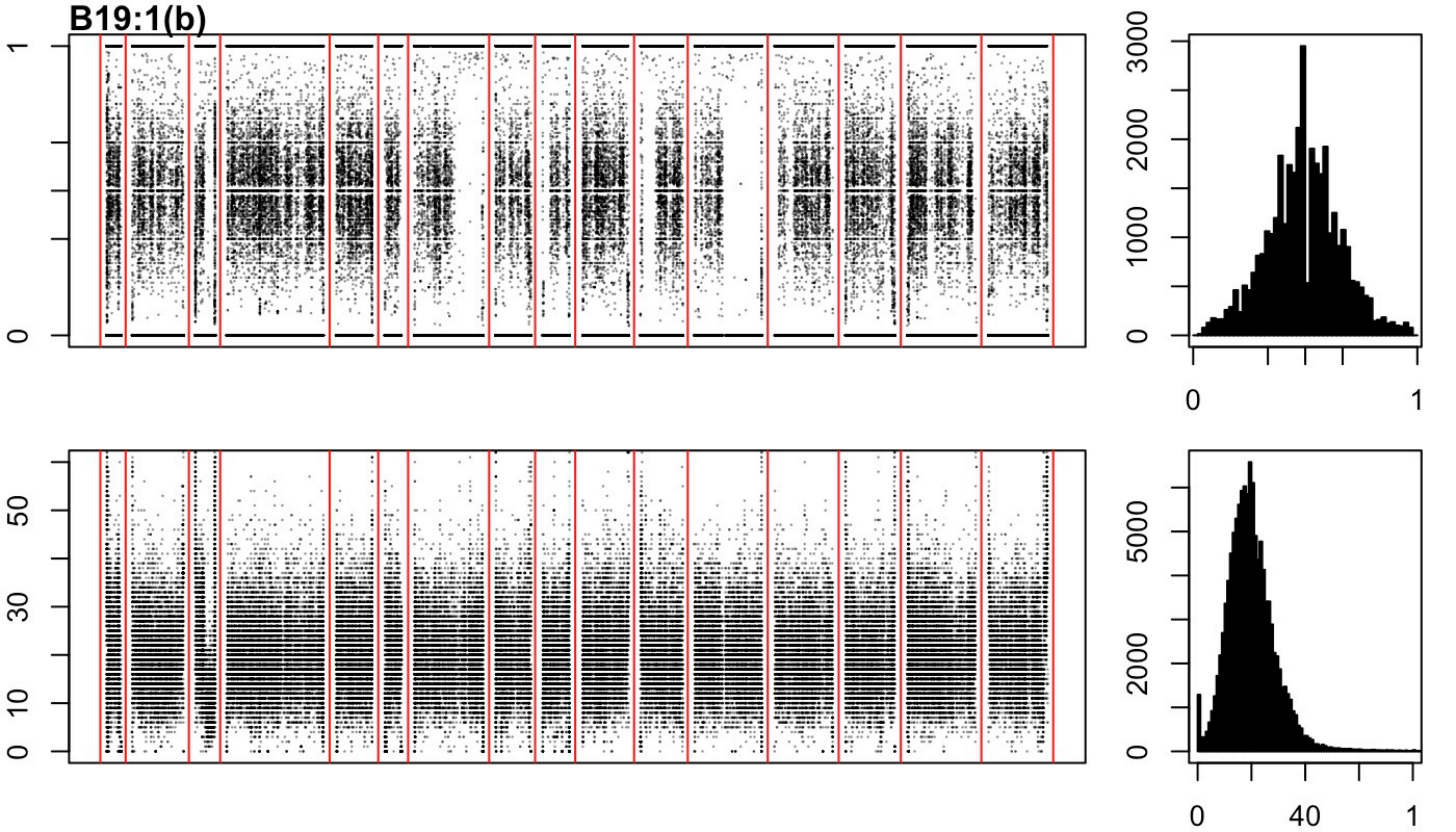

B19:1(b)
1
0
3000
2000
1000
0
0
1
50
30
10
0
5000
2000
0
0
40
1

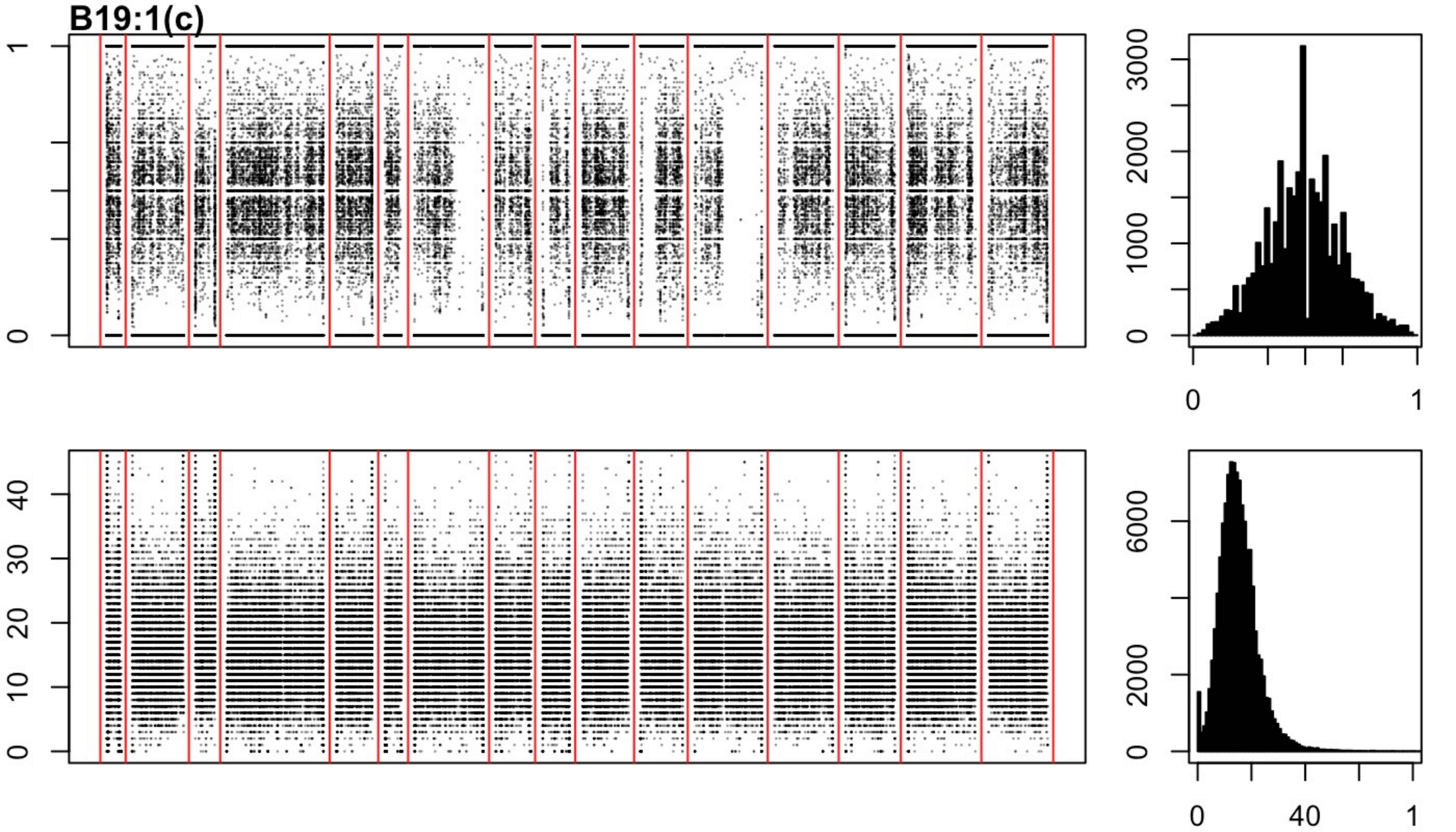
B19:1(c)
0
1
3000
2000
1000
0
0
1
0
10
20
30
40
6000
2000
0
0
40

B19:5(a)

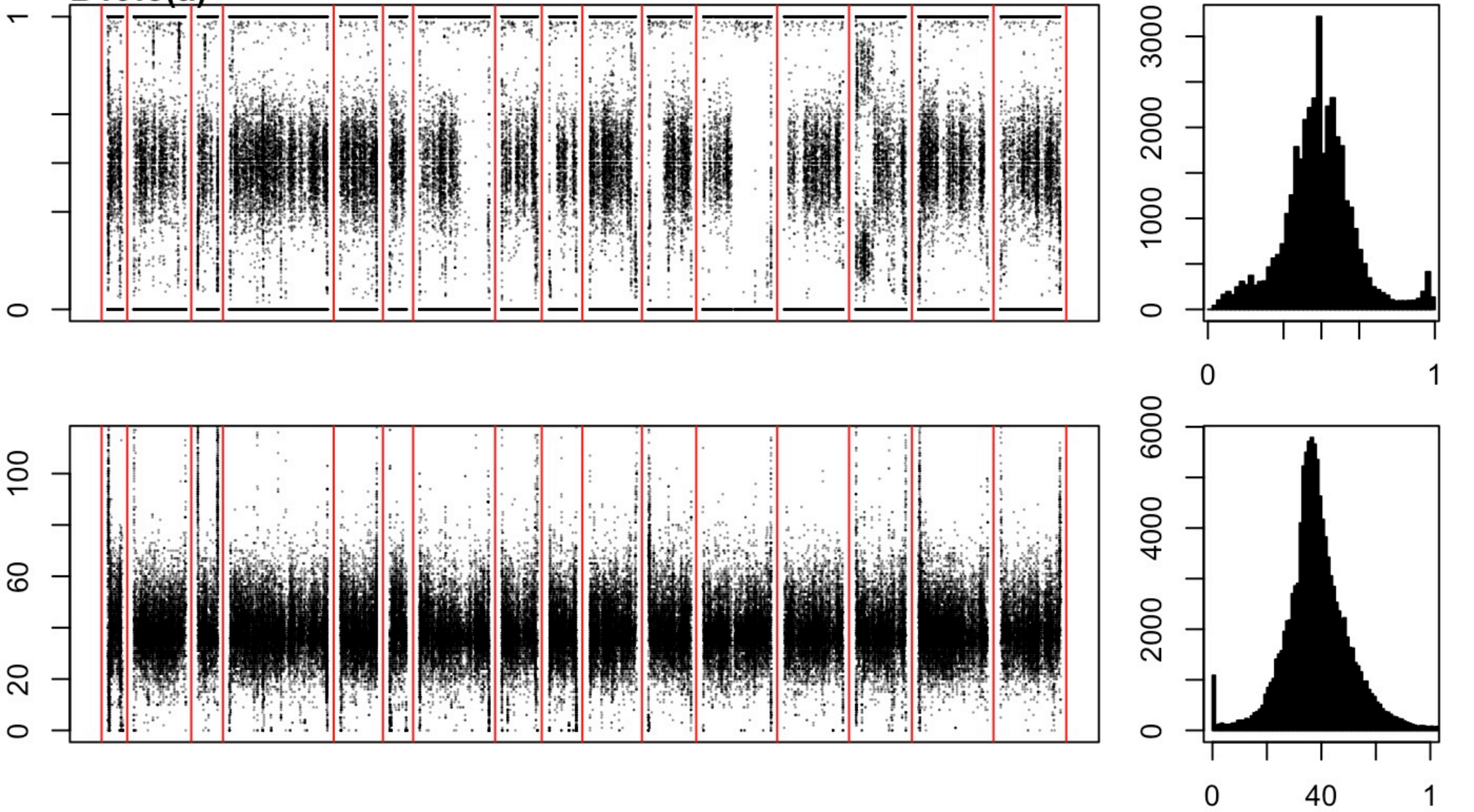

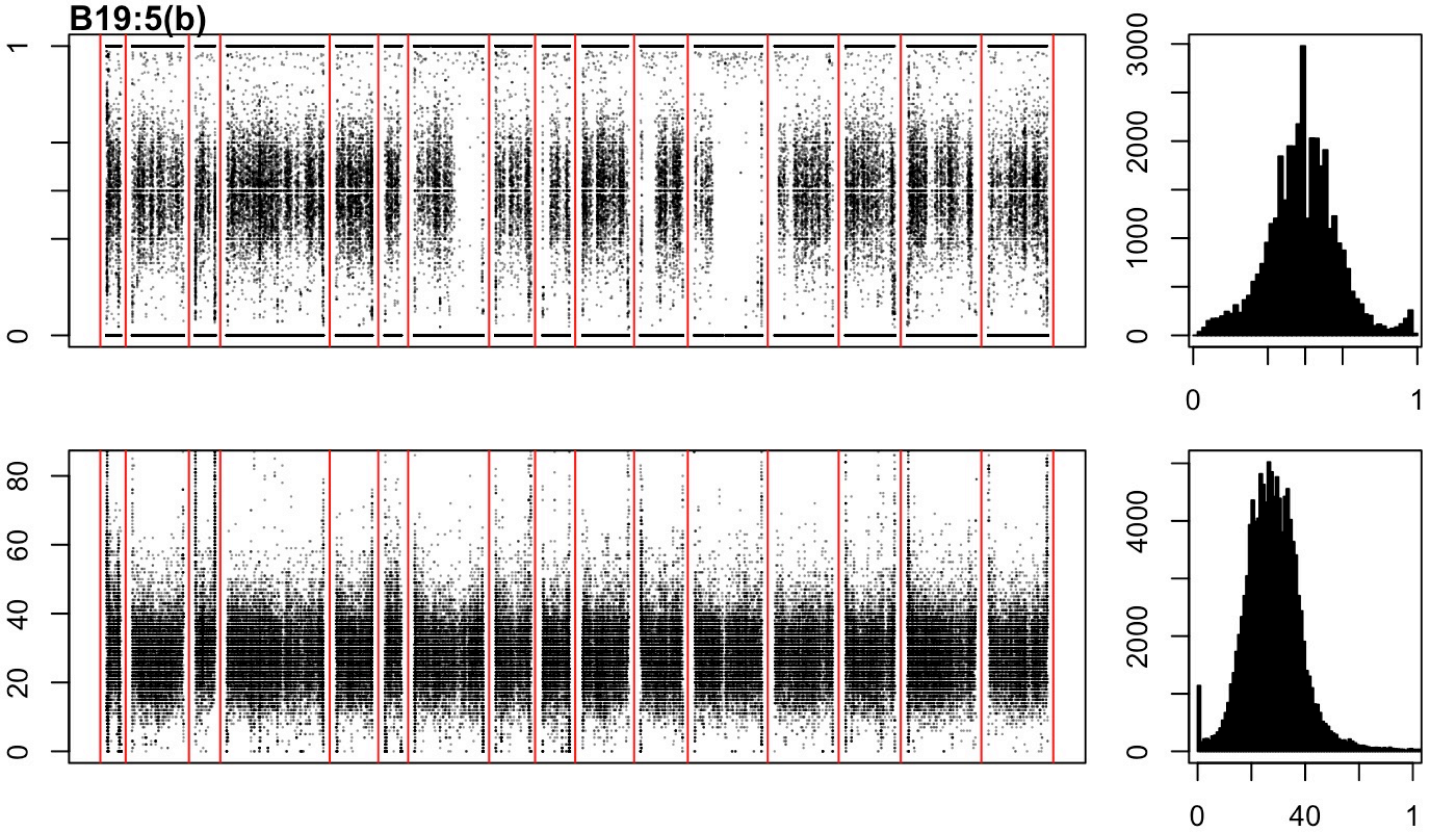
B19:5(b)
1
0
3000
2000
1000
0
0
1
80
60
40
20
0
4000
2000
0
0
40
1

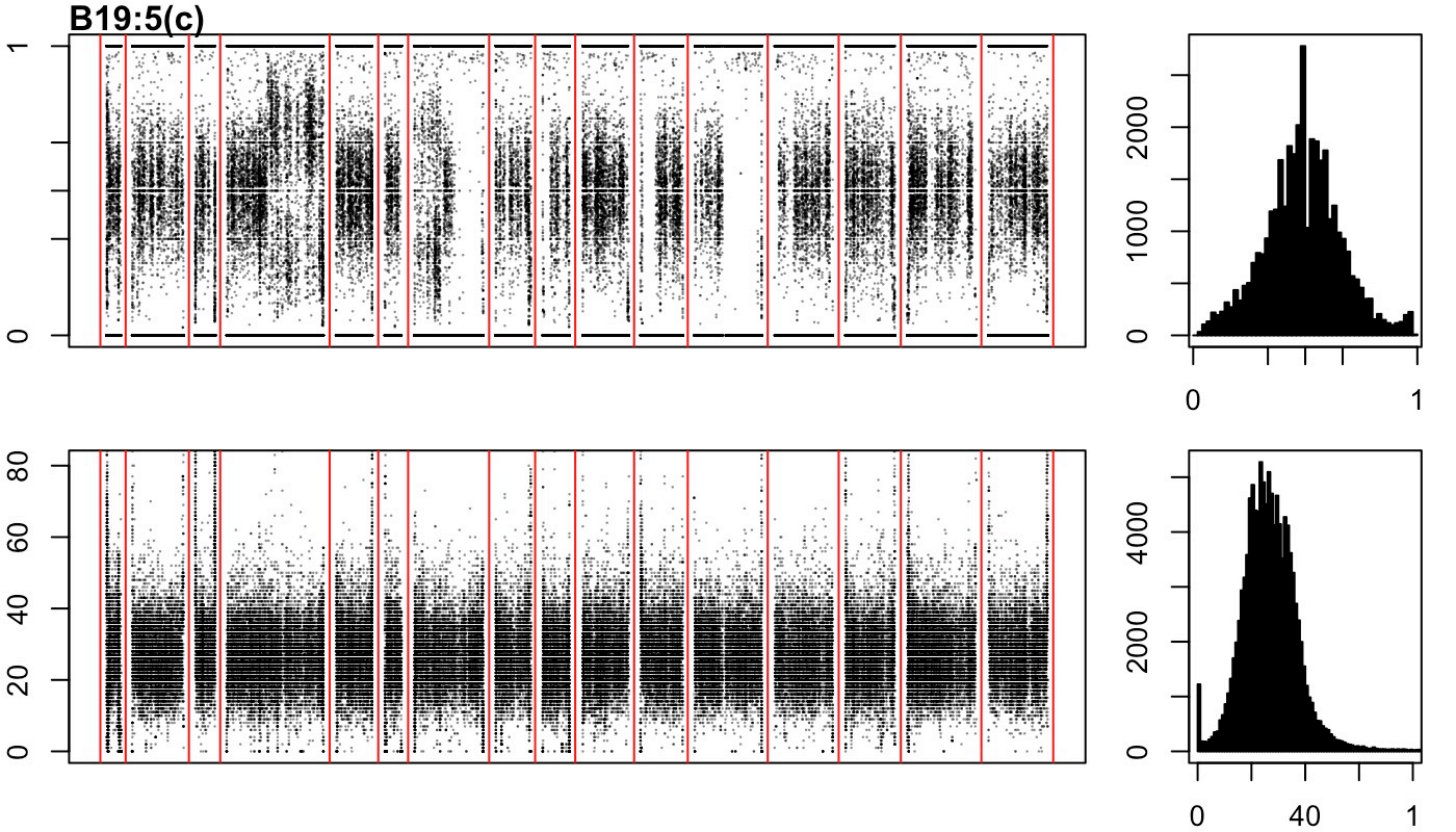
B19:5(c)
1
0
80
60
40
20
0
2000
1000
0
0
1
4000
2000
0
0
40
1

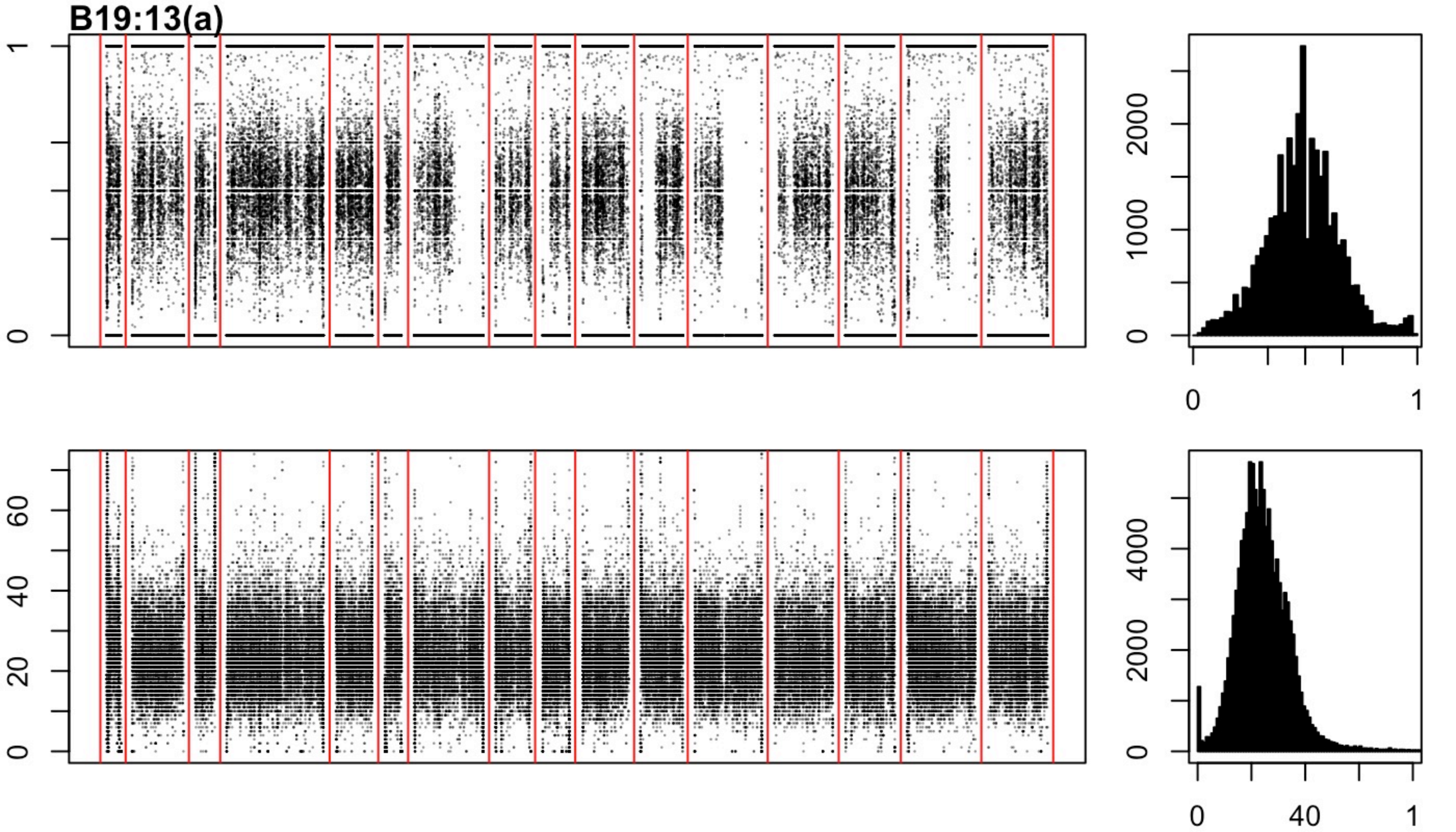

B19:13(a)
1
0
2000
1000
0
0
1
60
40
20
0
4000
2000
0
0
40
1

B19:13(b)

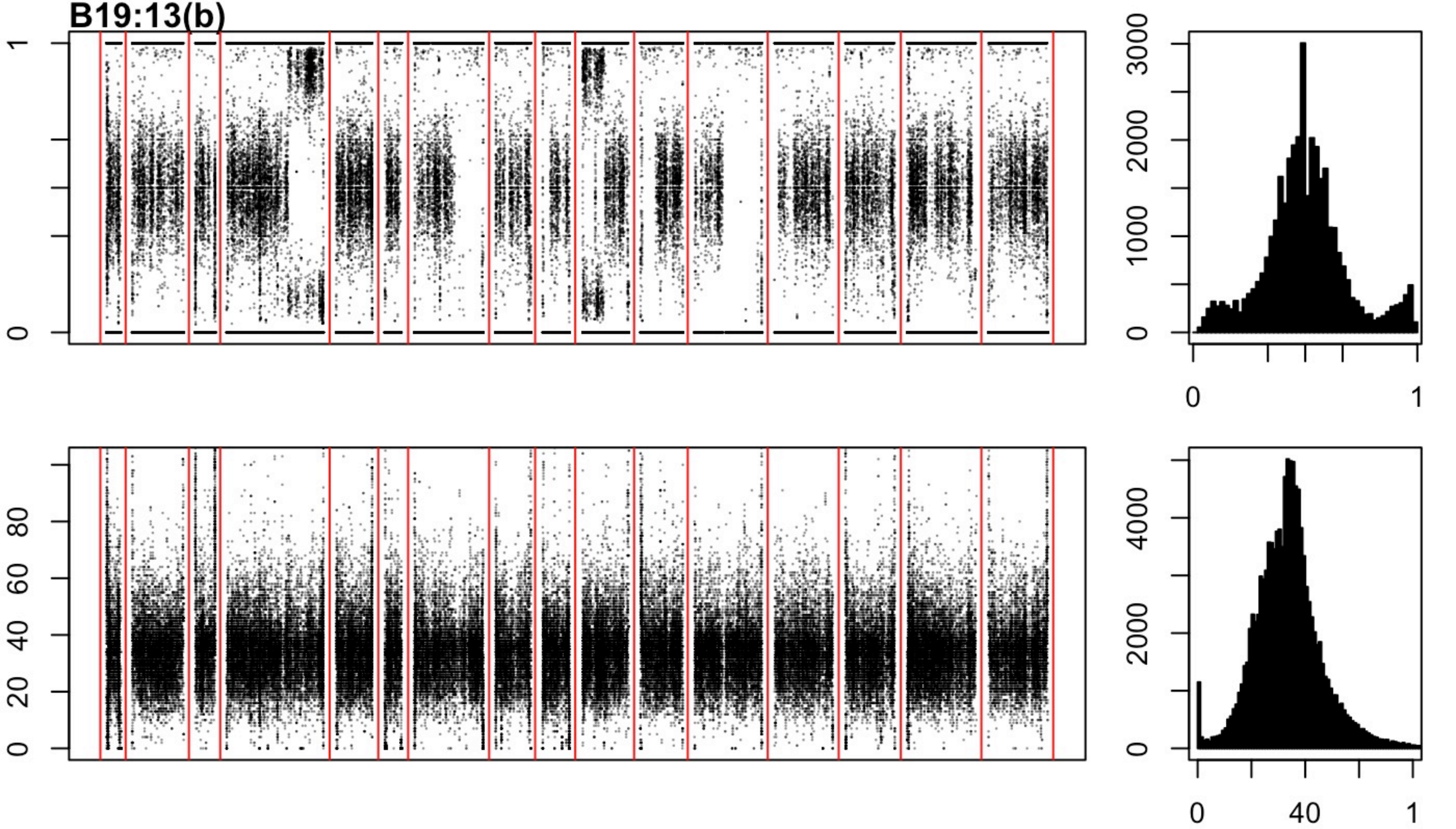

B19:13(c)

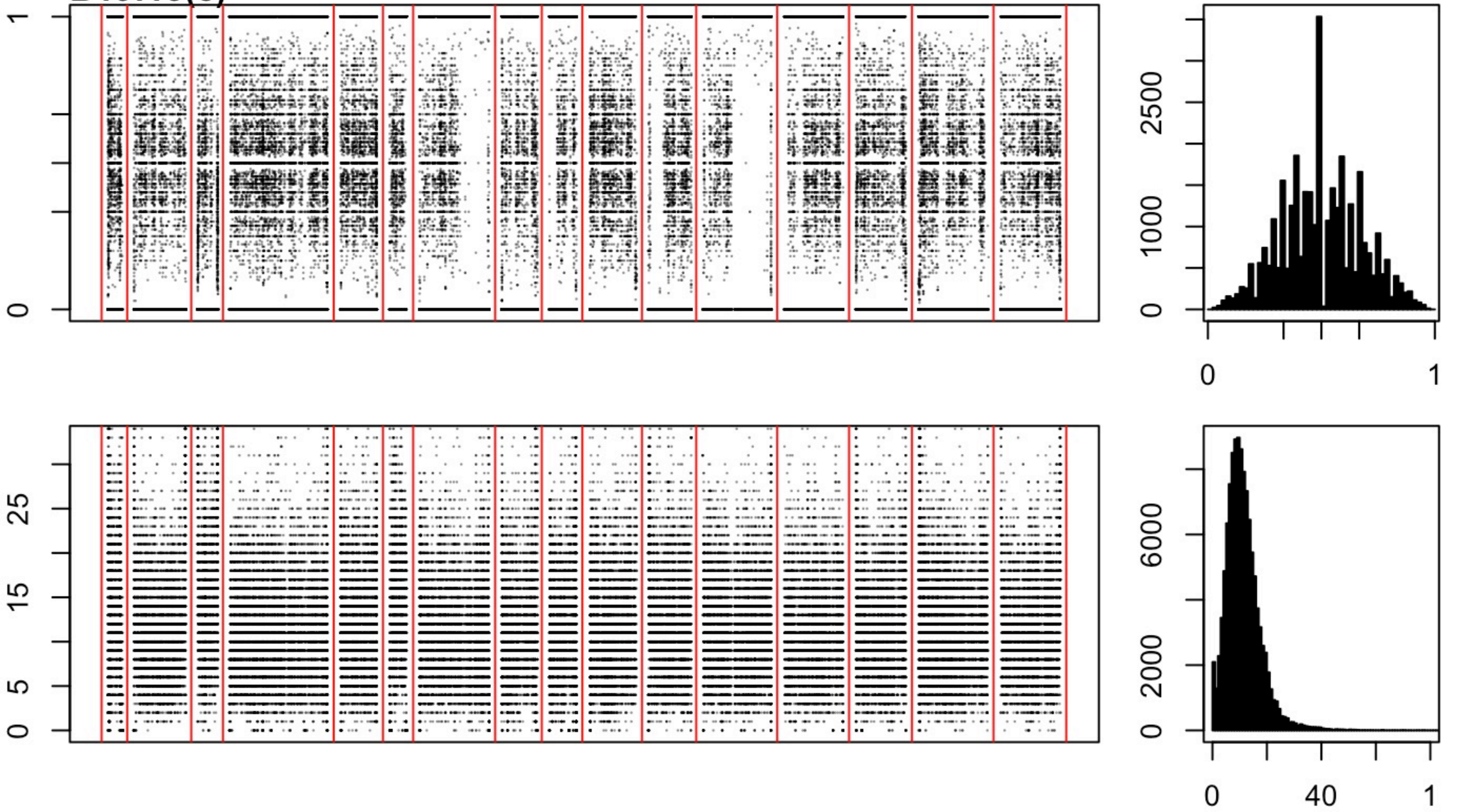

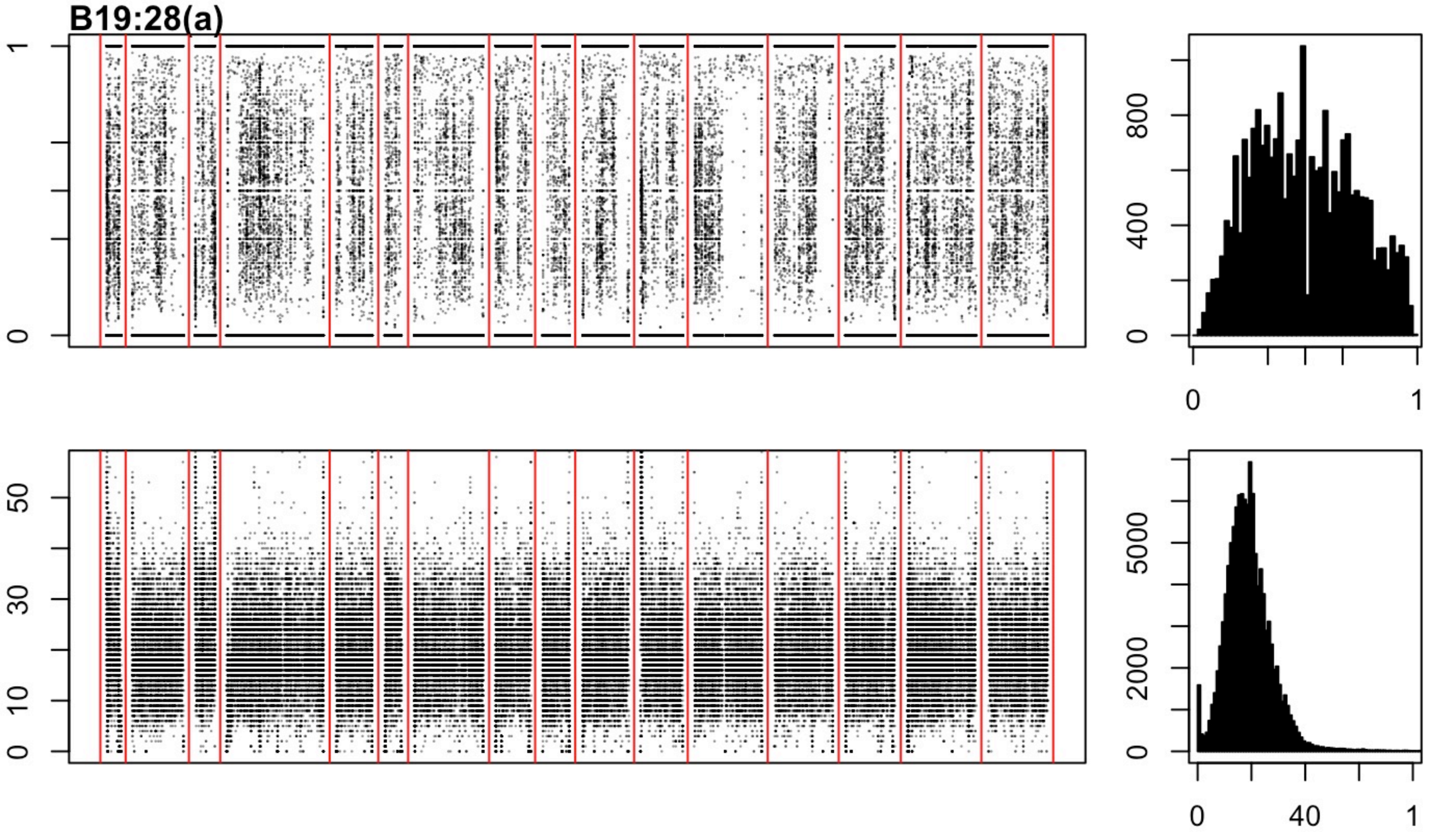

B19:28(a)
1
0
800
400
0
0
1
50
30
10
0
5000
2000
0
0
40
1

B19:28(b)

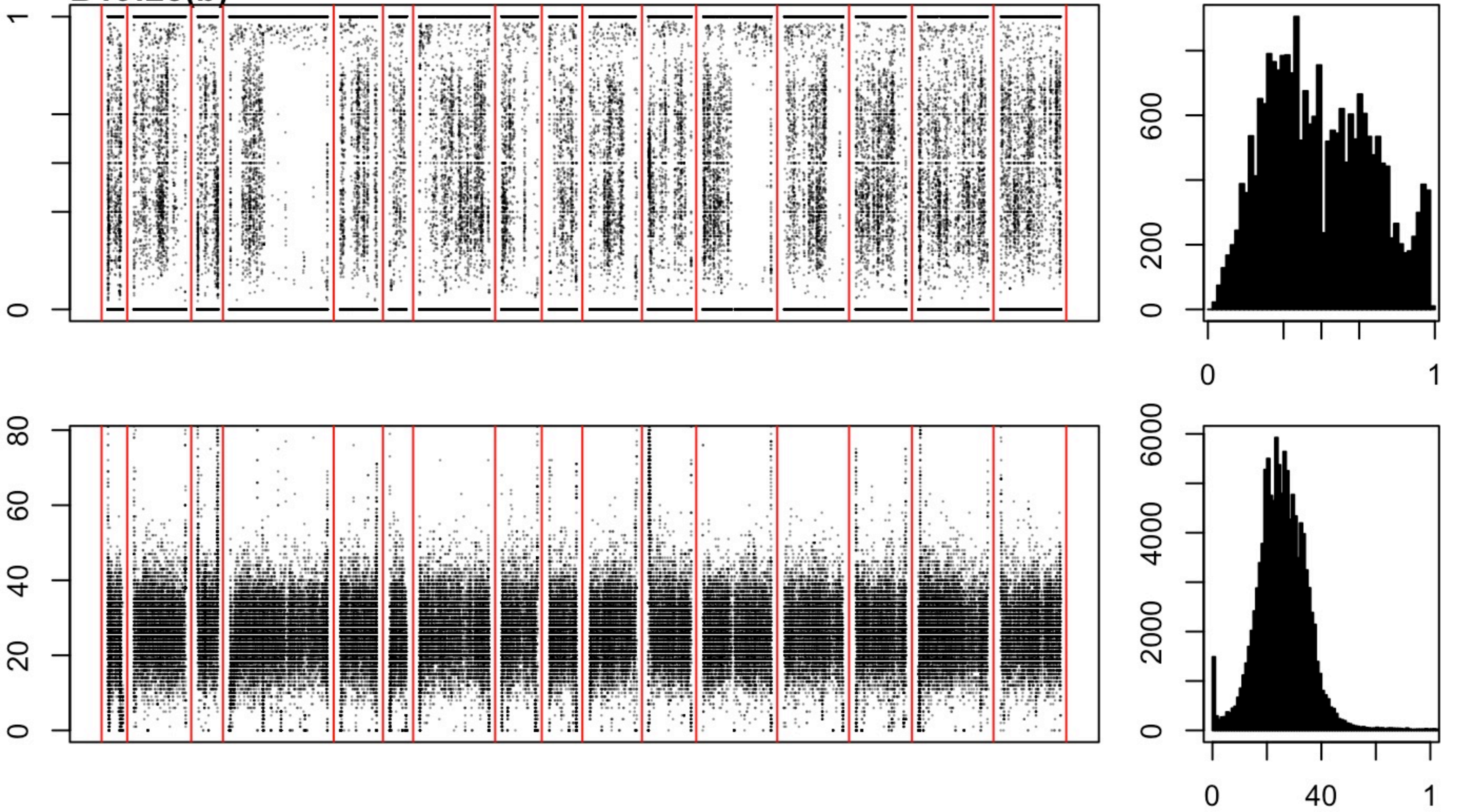

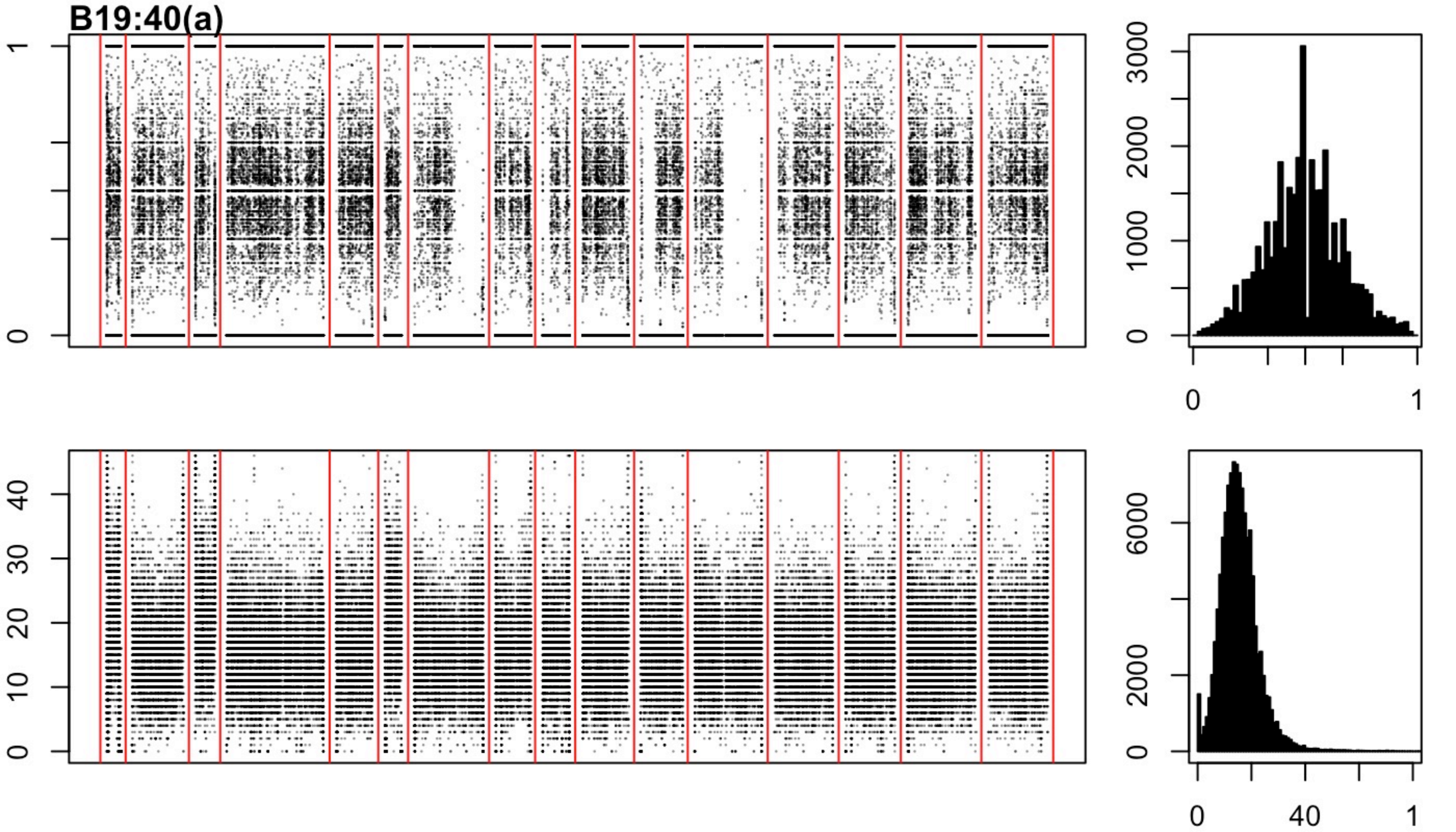
B19:40(a)
1
0
3000
2000
1000
0
0
1
40
30
20
10
0
6000
2000
0
0
40
1

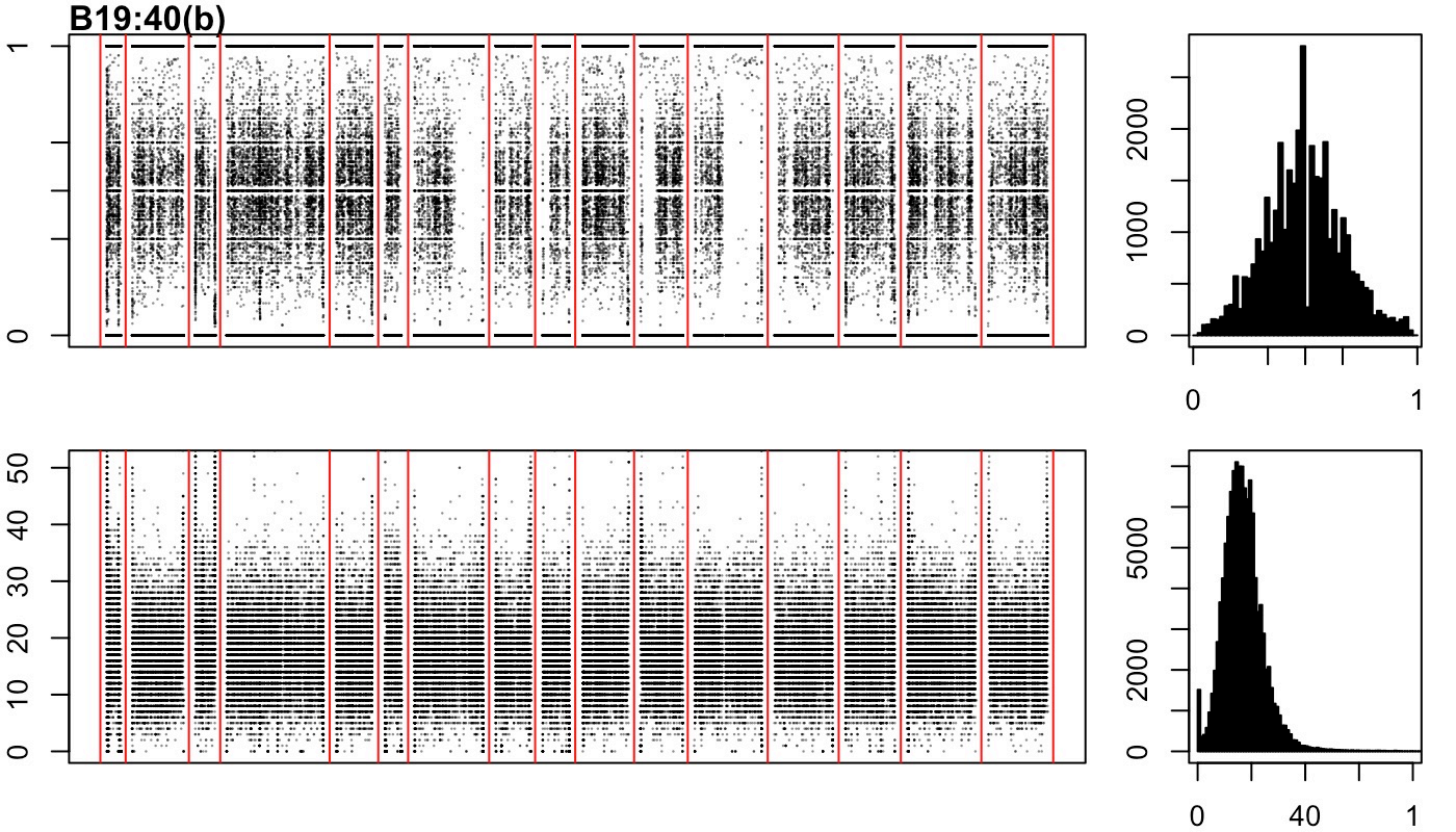

B19:40(b)
1
0
0
1
2000
1000
0
50
40
30
20
10
0
5000
2000
0
0
40
1

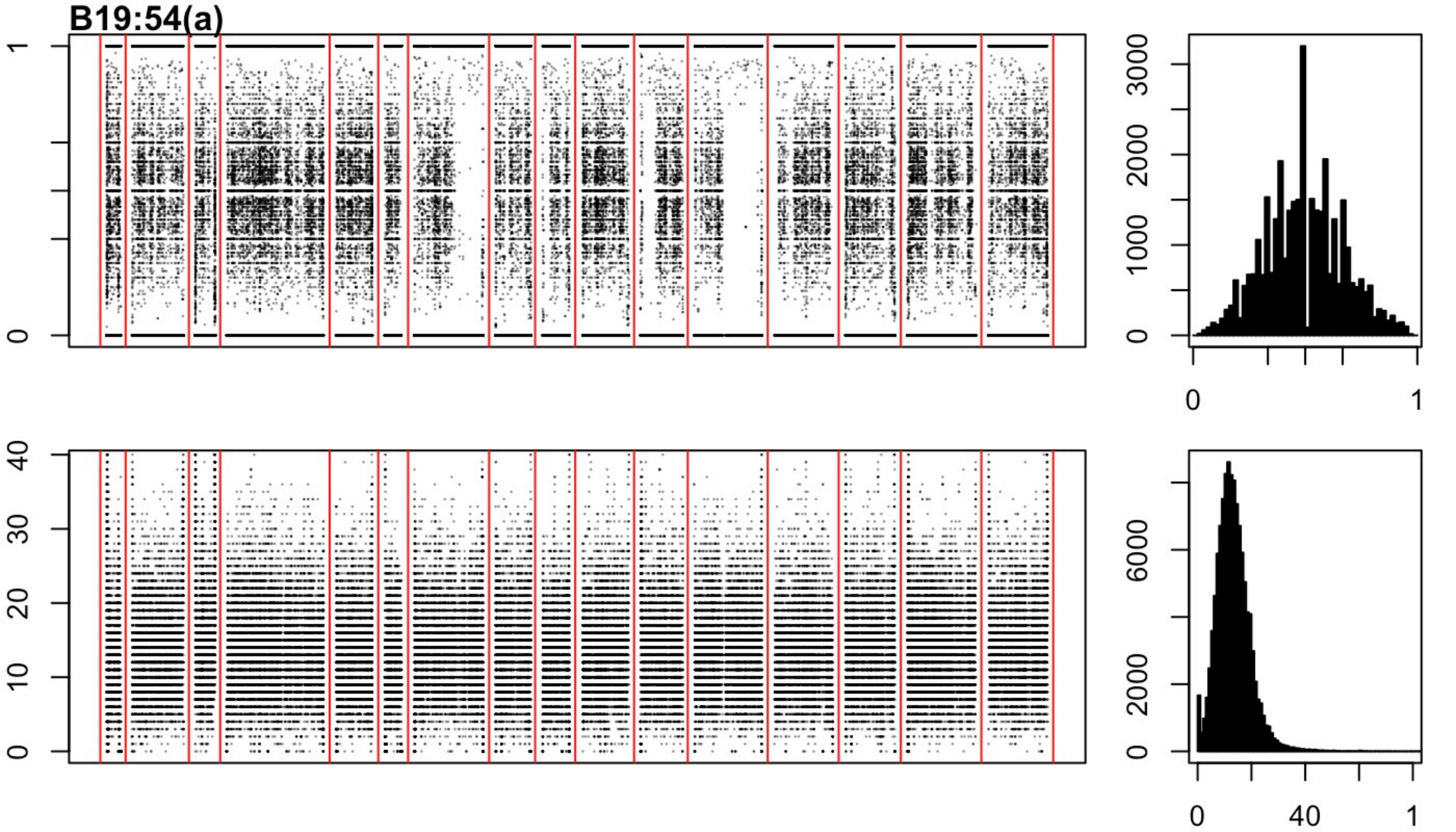
B19:54(a)
1
0
3000
2000
1000
0
0
1
40
30
20
10
0
6000
2000
0
0
40
1

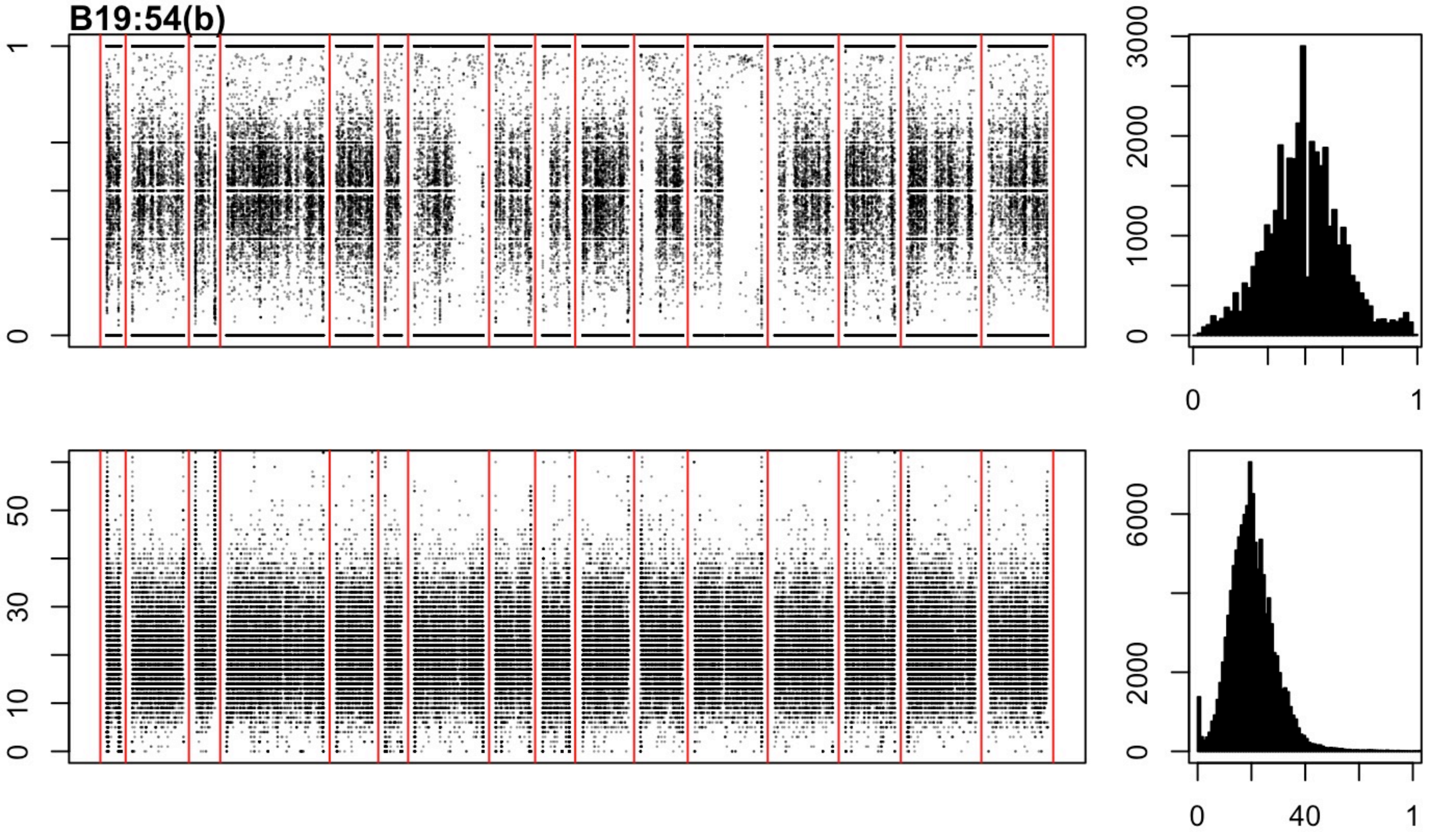

B19:54(b)
1
0
3000
2000
1000
0
0
1
50
30
10
0
6000
2000
0
0
40
1

B19:67(a)

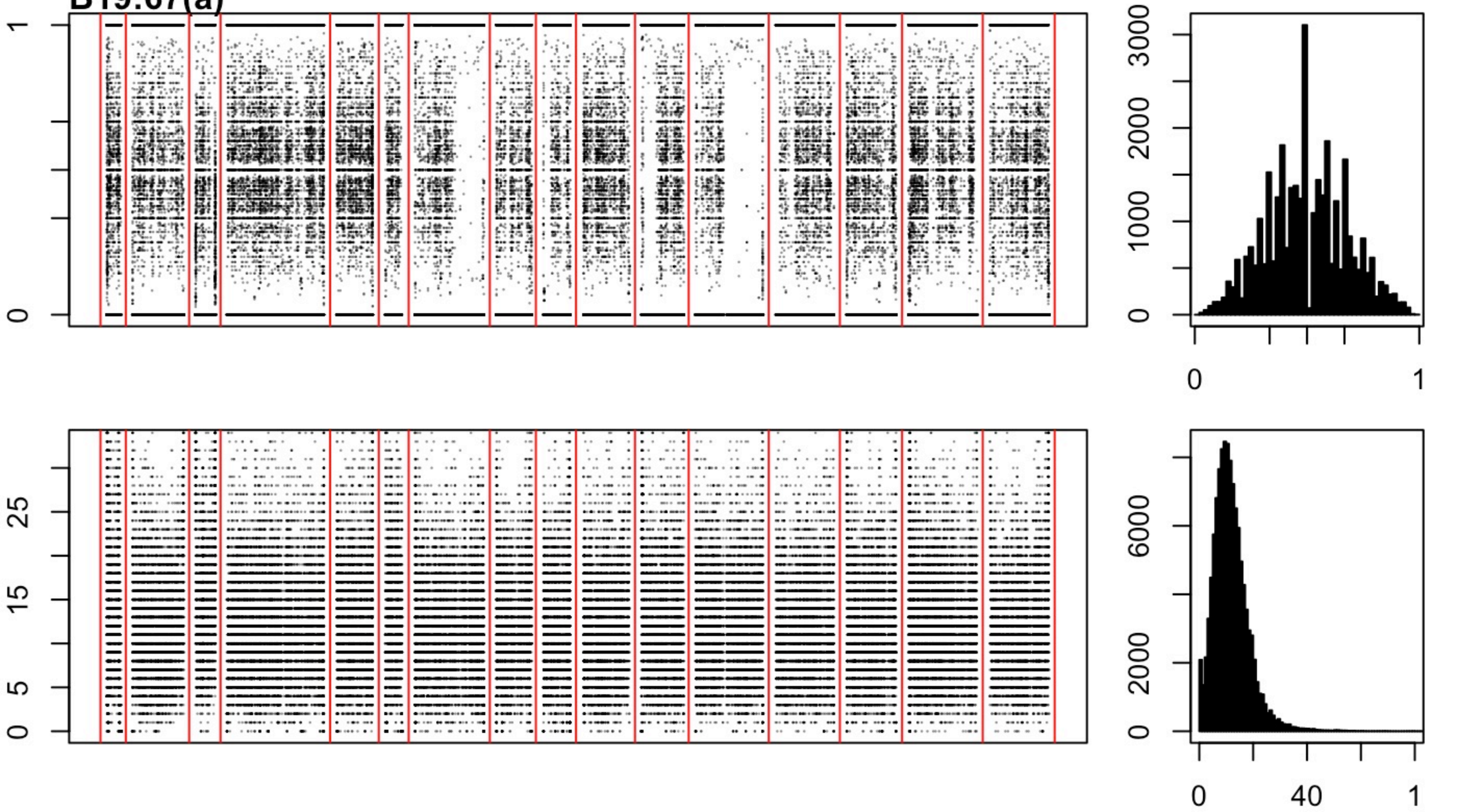

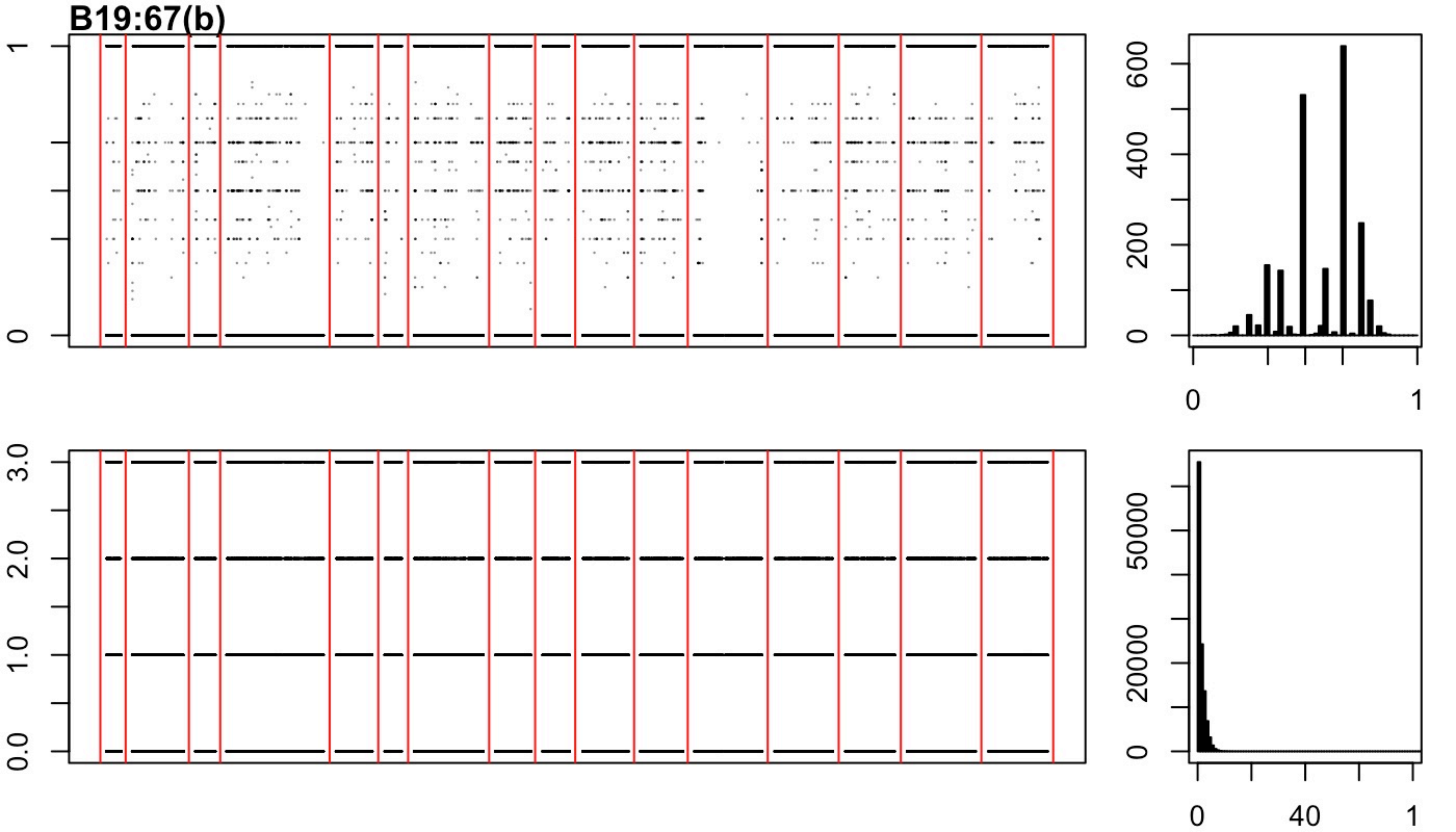

B19:67(b)
1
0
600
400
200
0
0
1
3.0
2.0
1.0
0.0
50000
20000
0
0
40
1

B19:81(a)

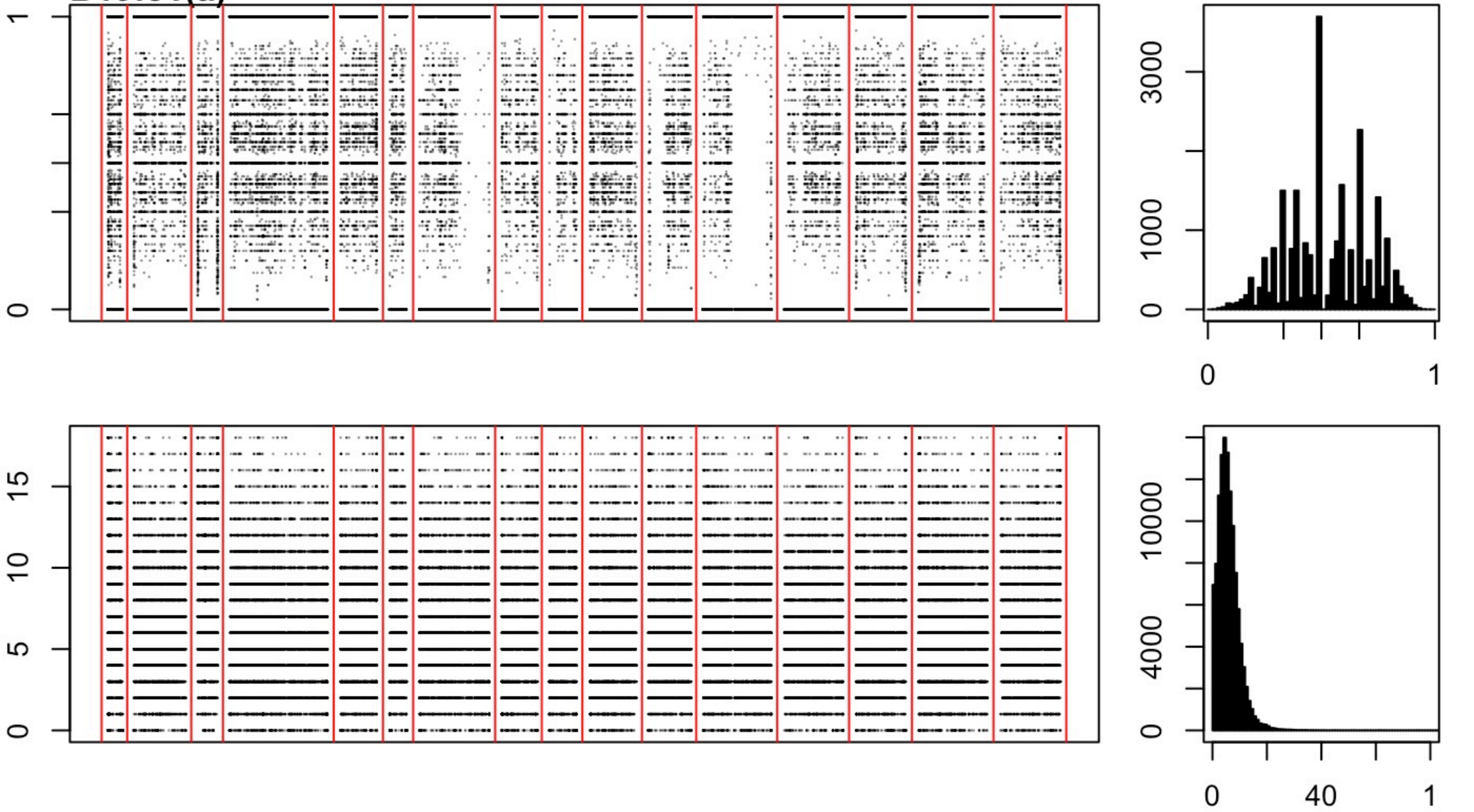

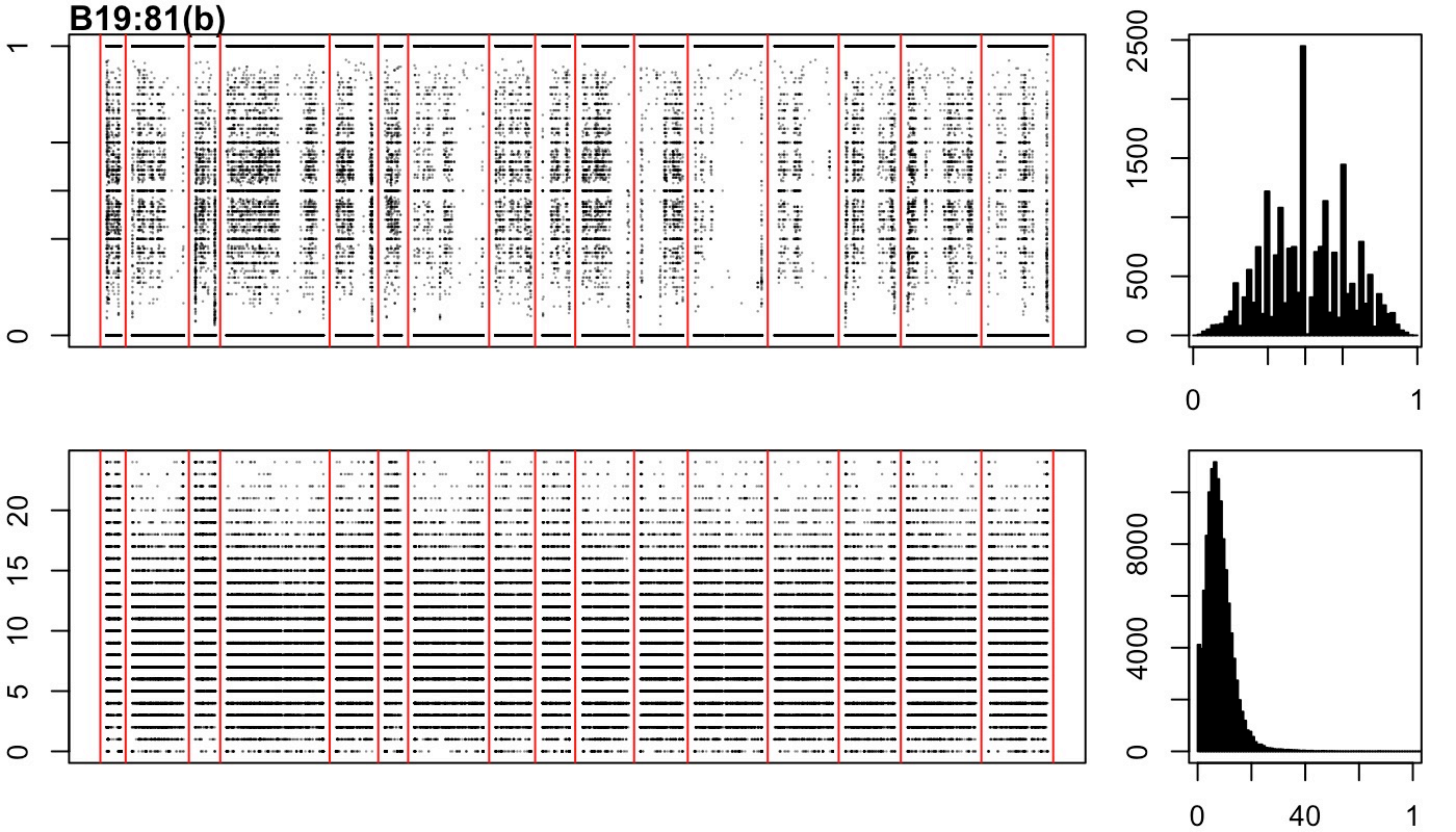
B19:81(b)
0
1
2500
1500
500
0
0
1
20
15
10
5
0
8000
4000
0
0
40
1

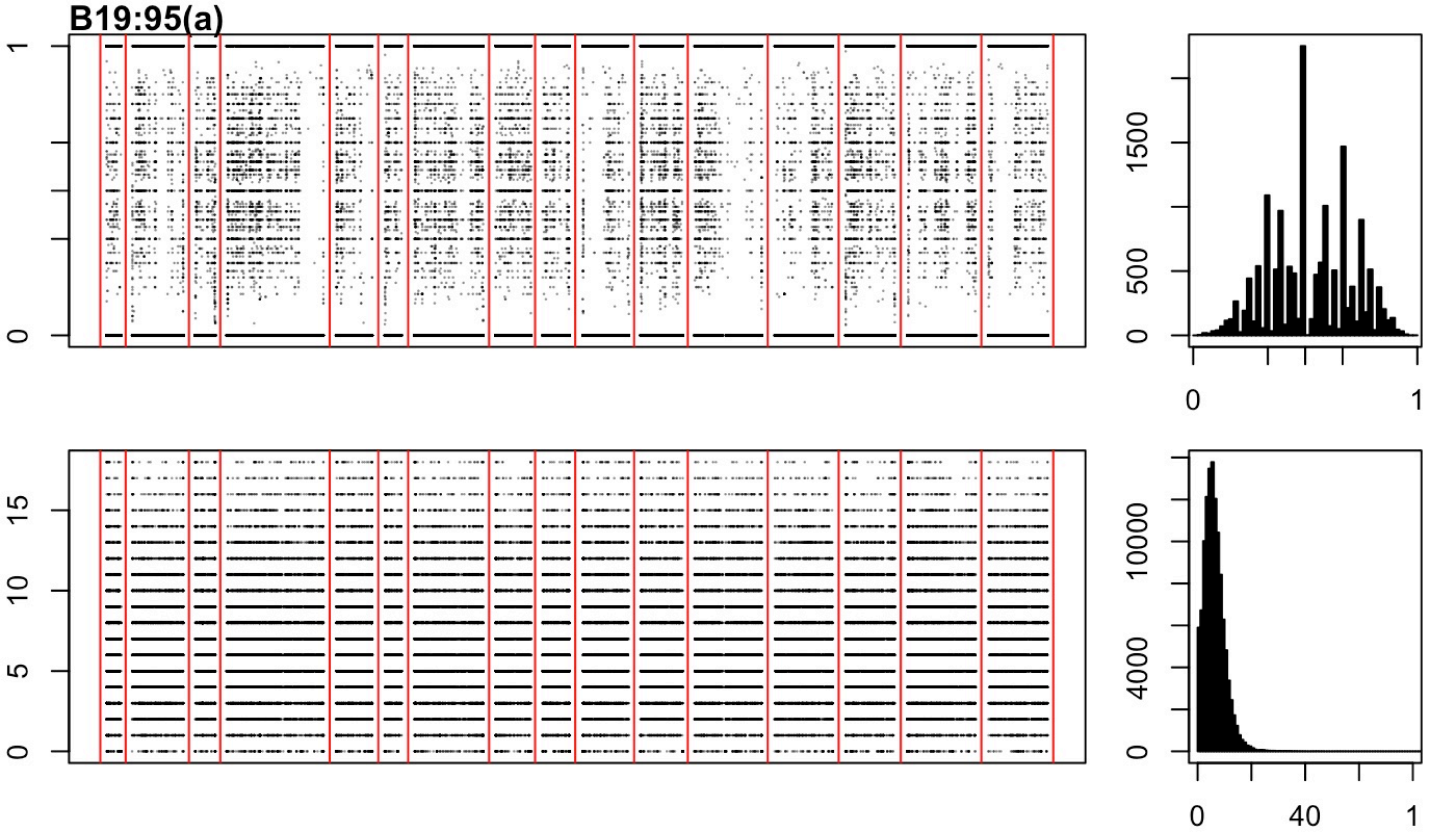
B19:95(a)
1
0
1500
500
0
0
1
15
10
5
0
10000
4000
0
0
40
1

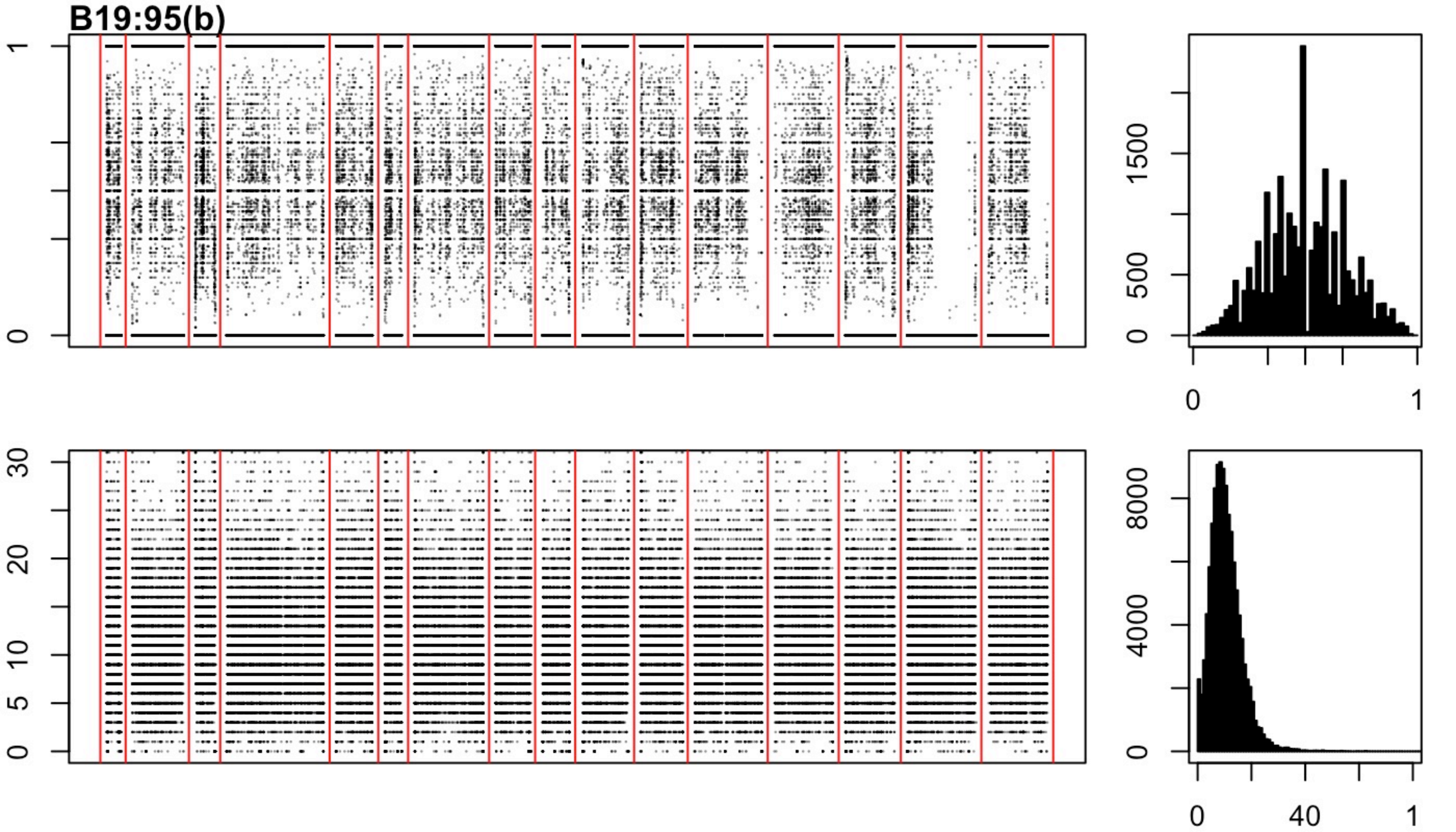

B19:95(b)
1
0
1500
500
0
0
1
30
20
10
5
0
8000
4000
0
0
40
1

B19:109(a)

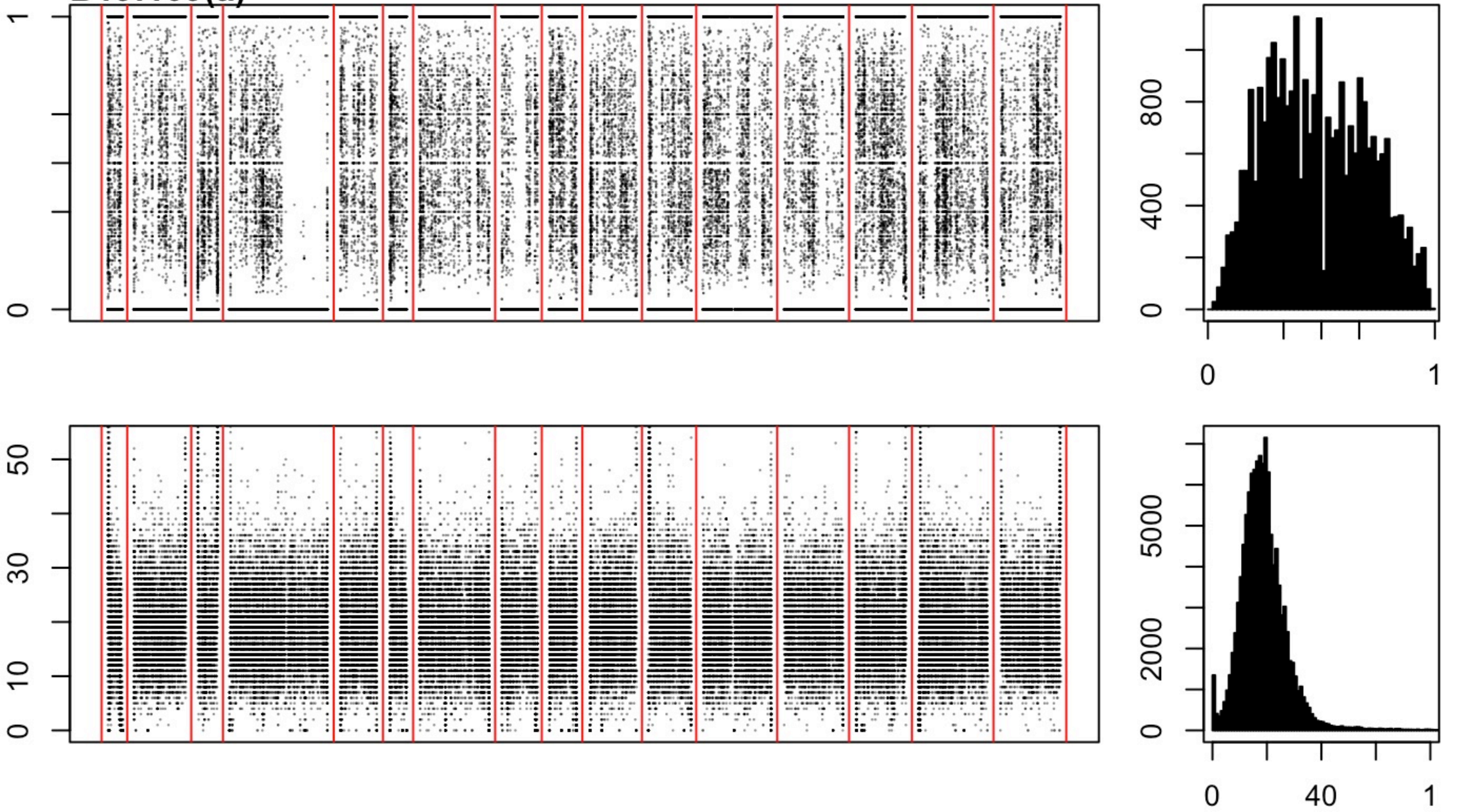

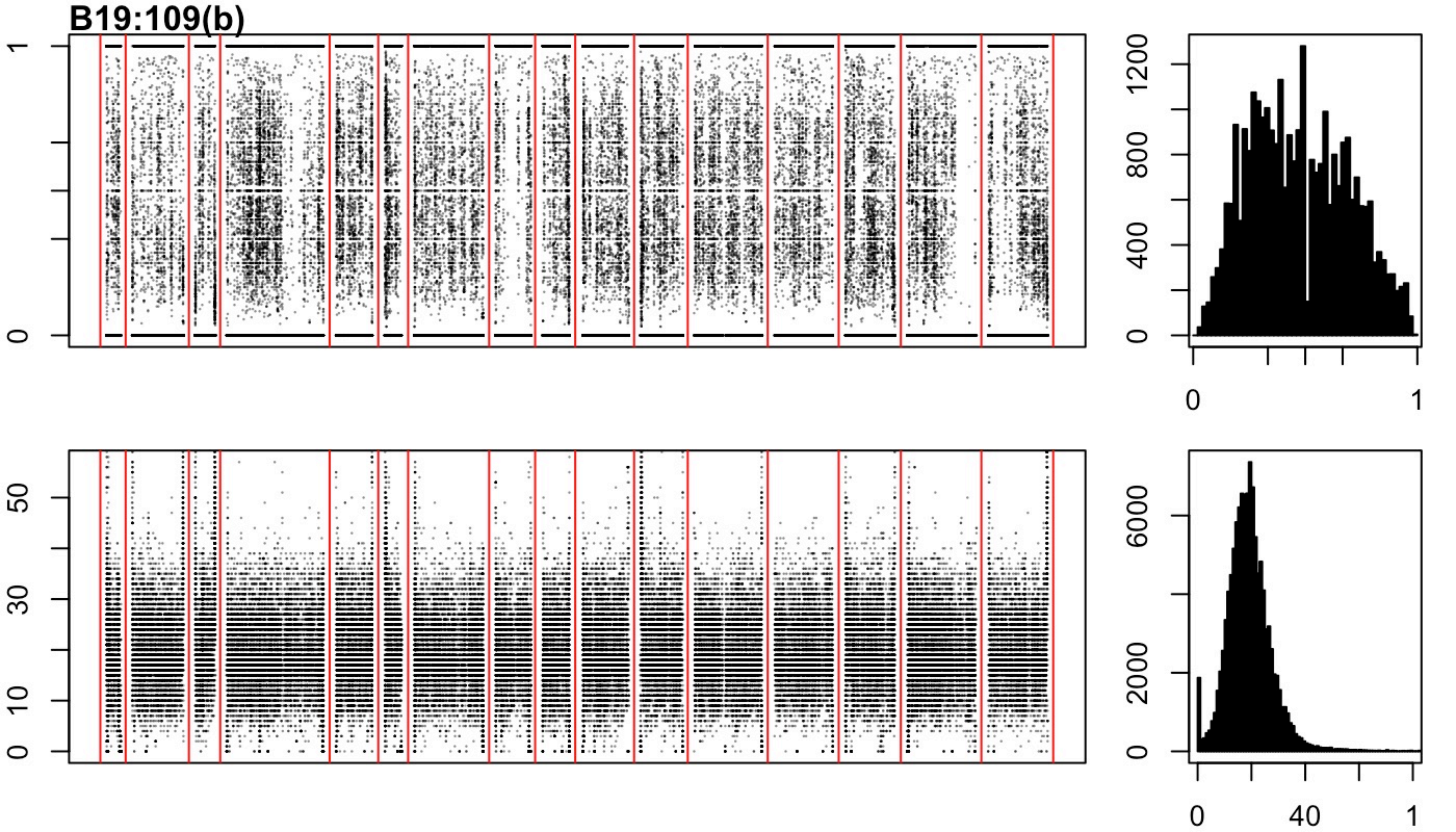

B19:109(b)
1
0
1200
800
400
0
0
1
50
30
10
0
6000
2000
0
0
40
1

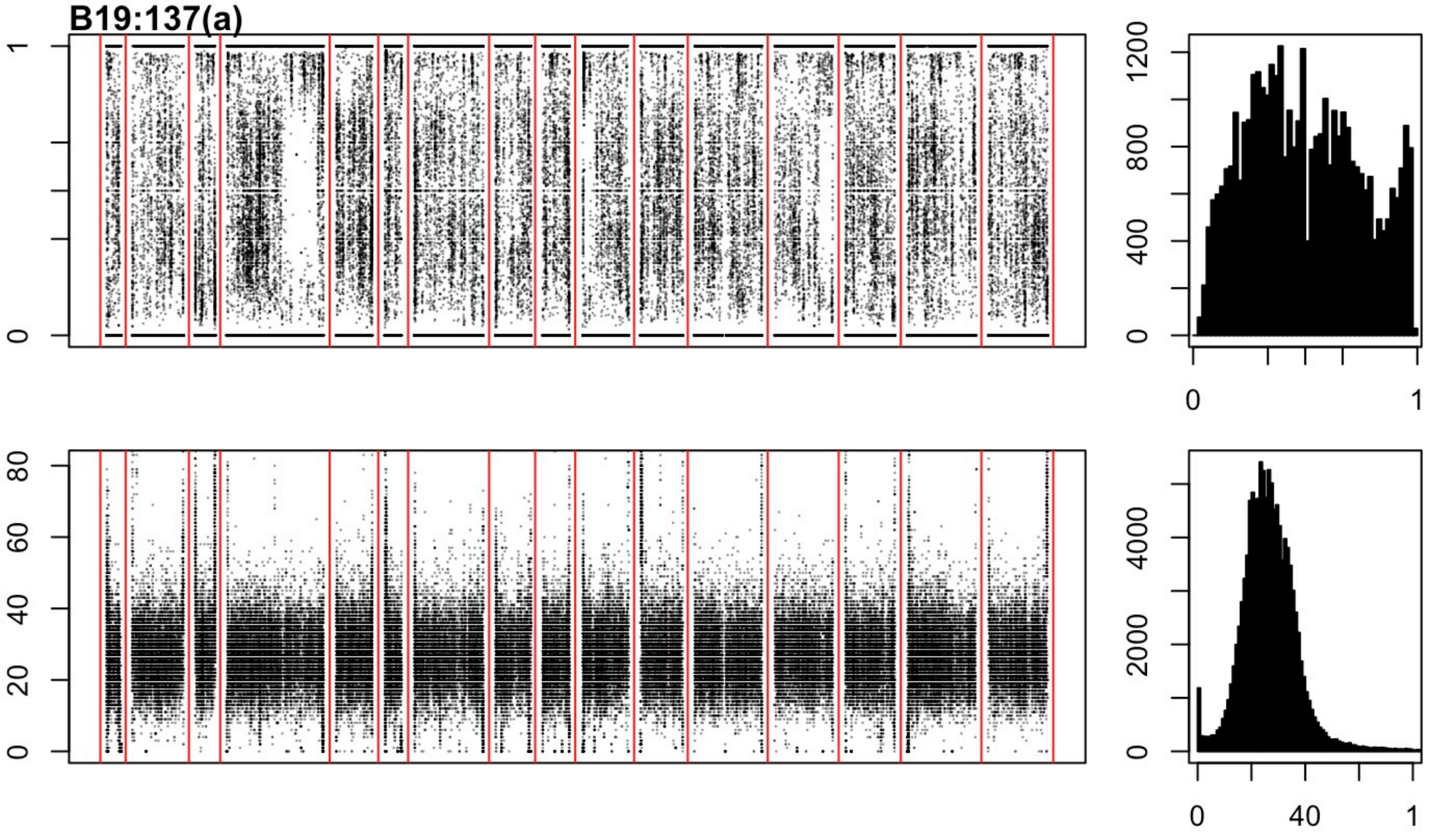
B19:137(a)
1
0
1200
800
400
0
0
1
80
60
40
20
0
4000
2000
0
0
40
1

B19:137(b)

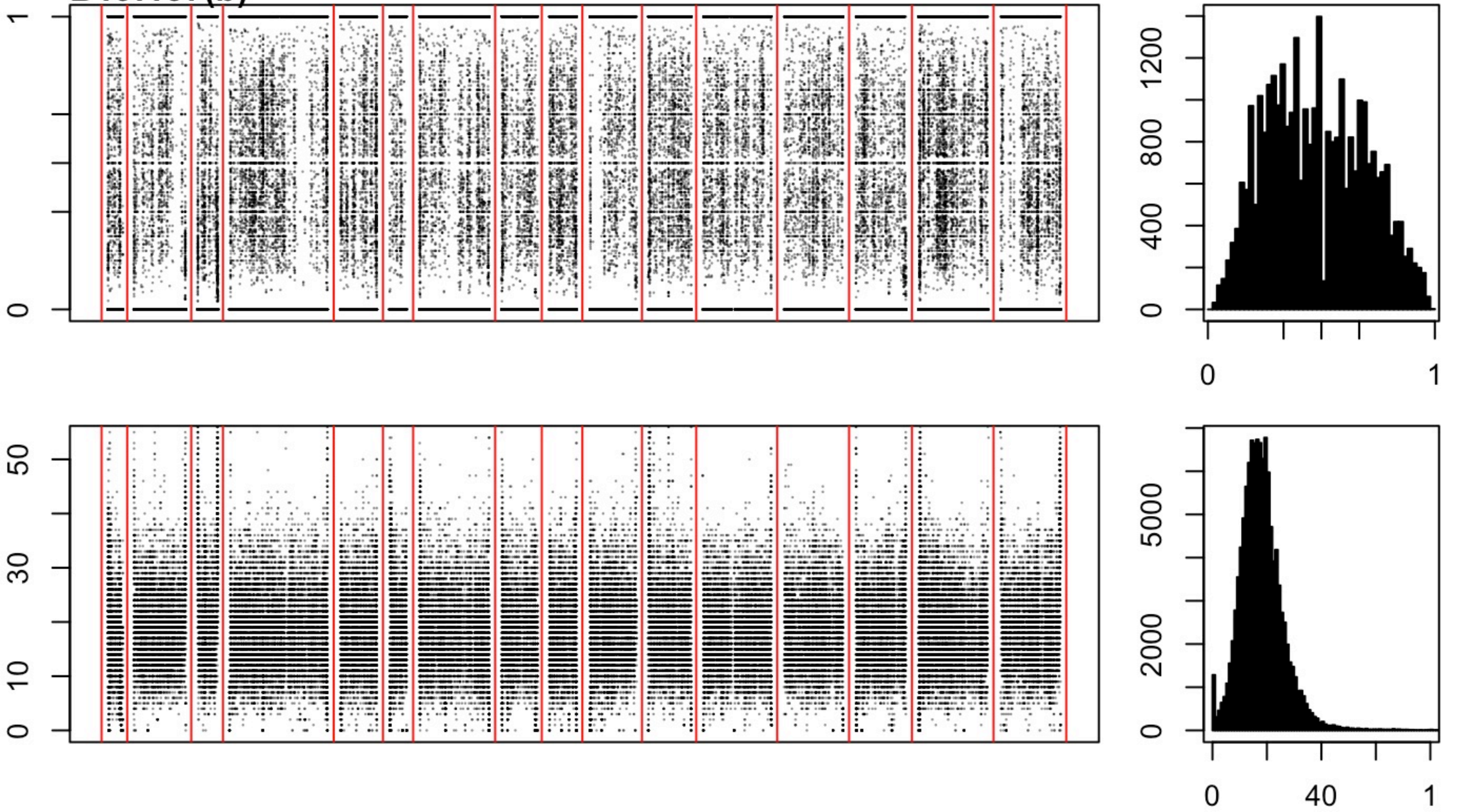

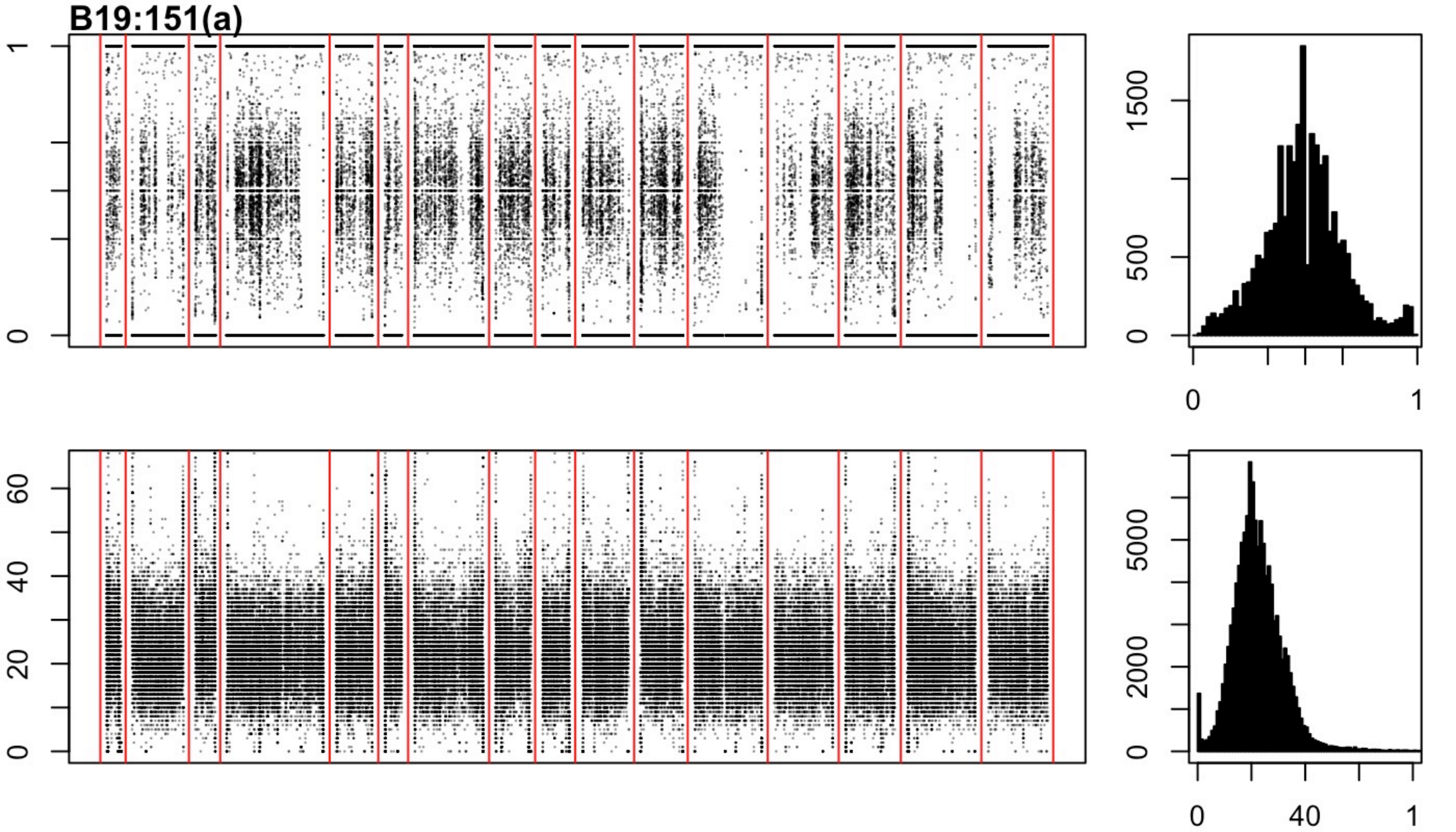

B19:151(a)
1
0
1500
500
0
0
1
60
40
20
0
5000
2000
0
0
40
1

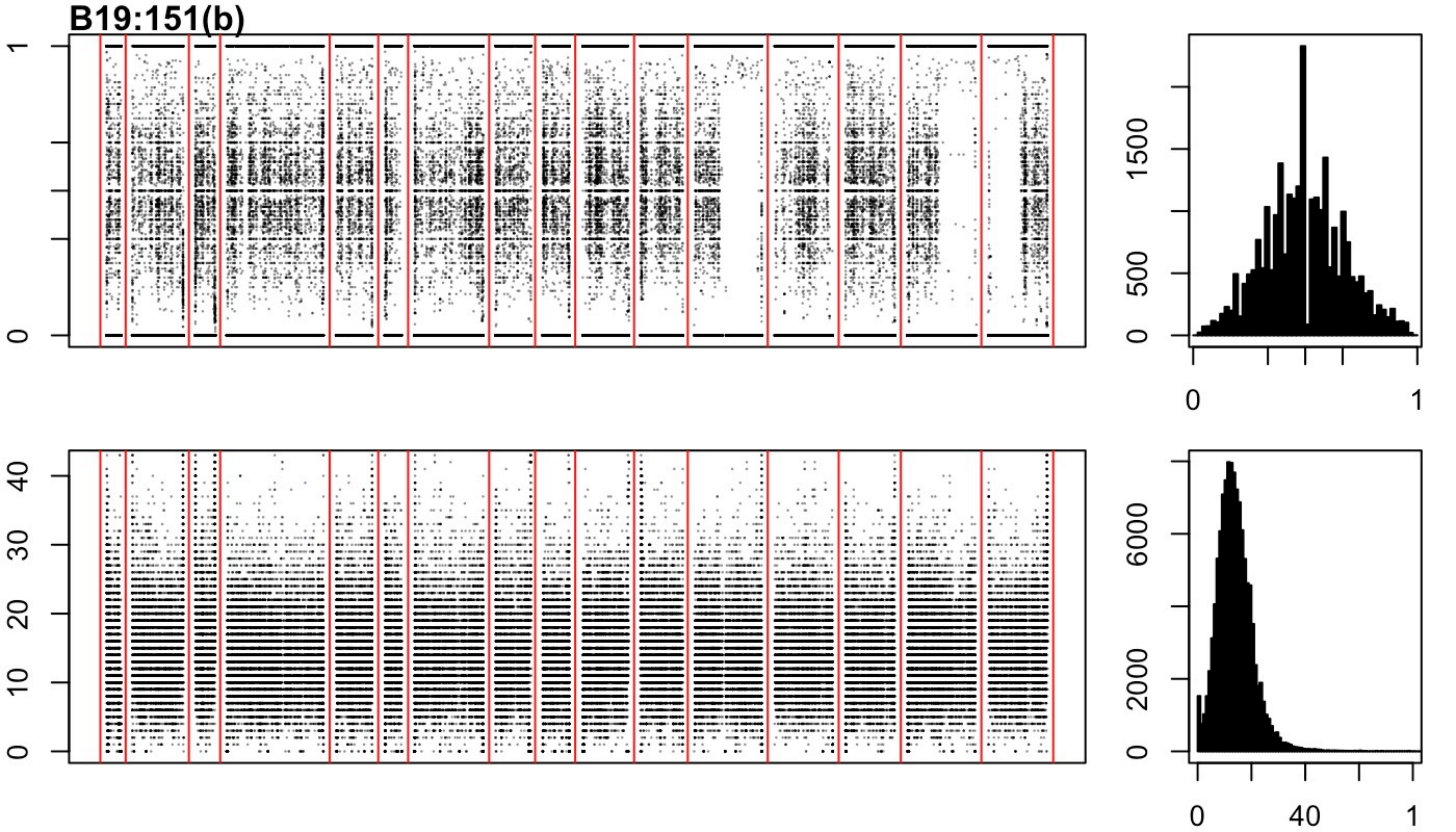
B19:151(b)
1
0
1500
500
0
0
1
40
30
20
10
0
6000
2000
0
0
40
1

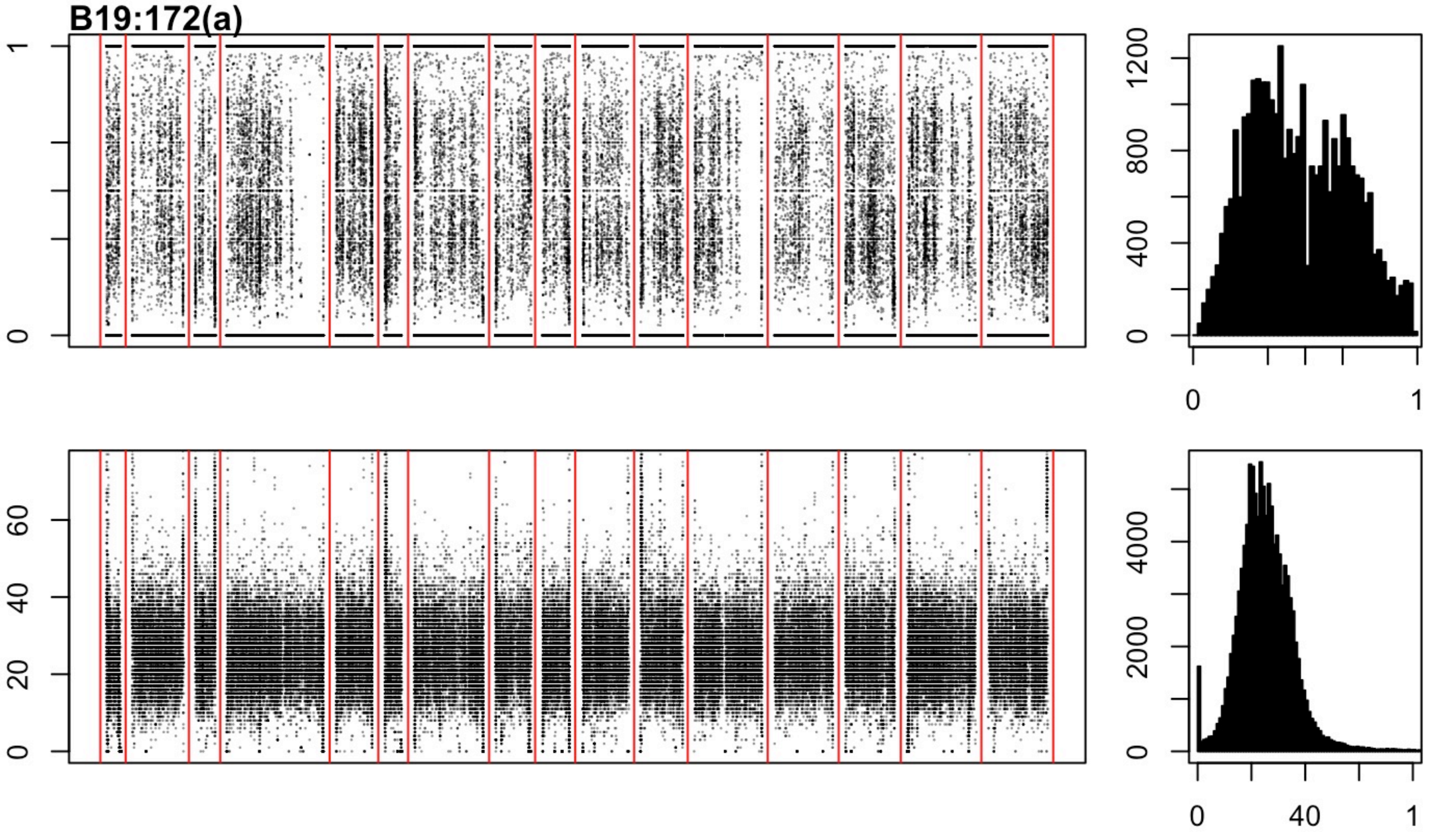

B19:172(a)
1
0
1200
800
400
0
0
1
60
40
20
0
4000
2000
0
0
40
1

B19:172(b)

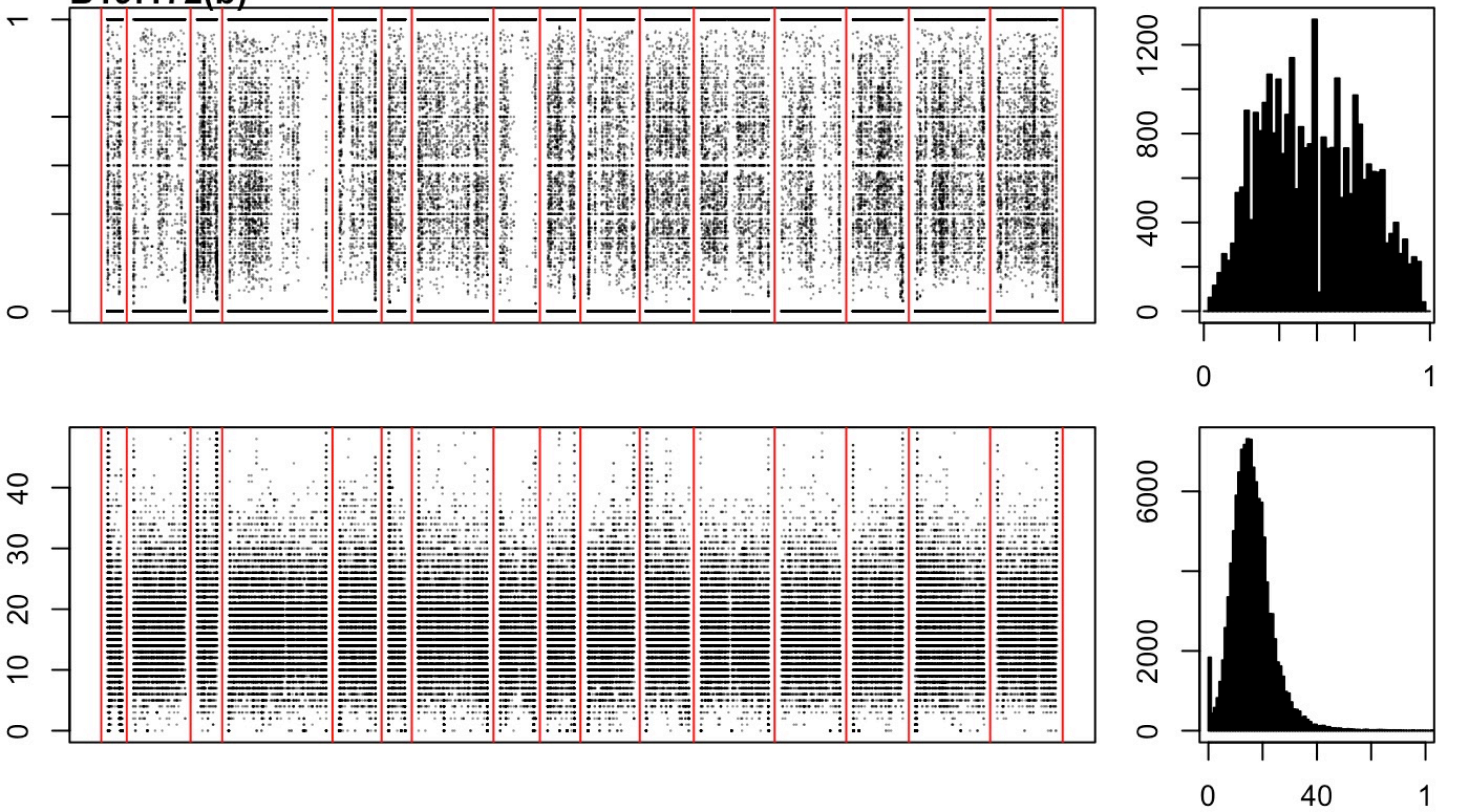

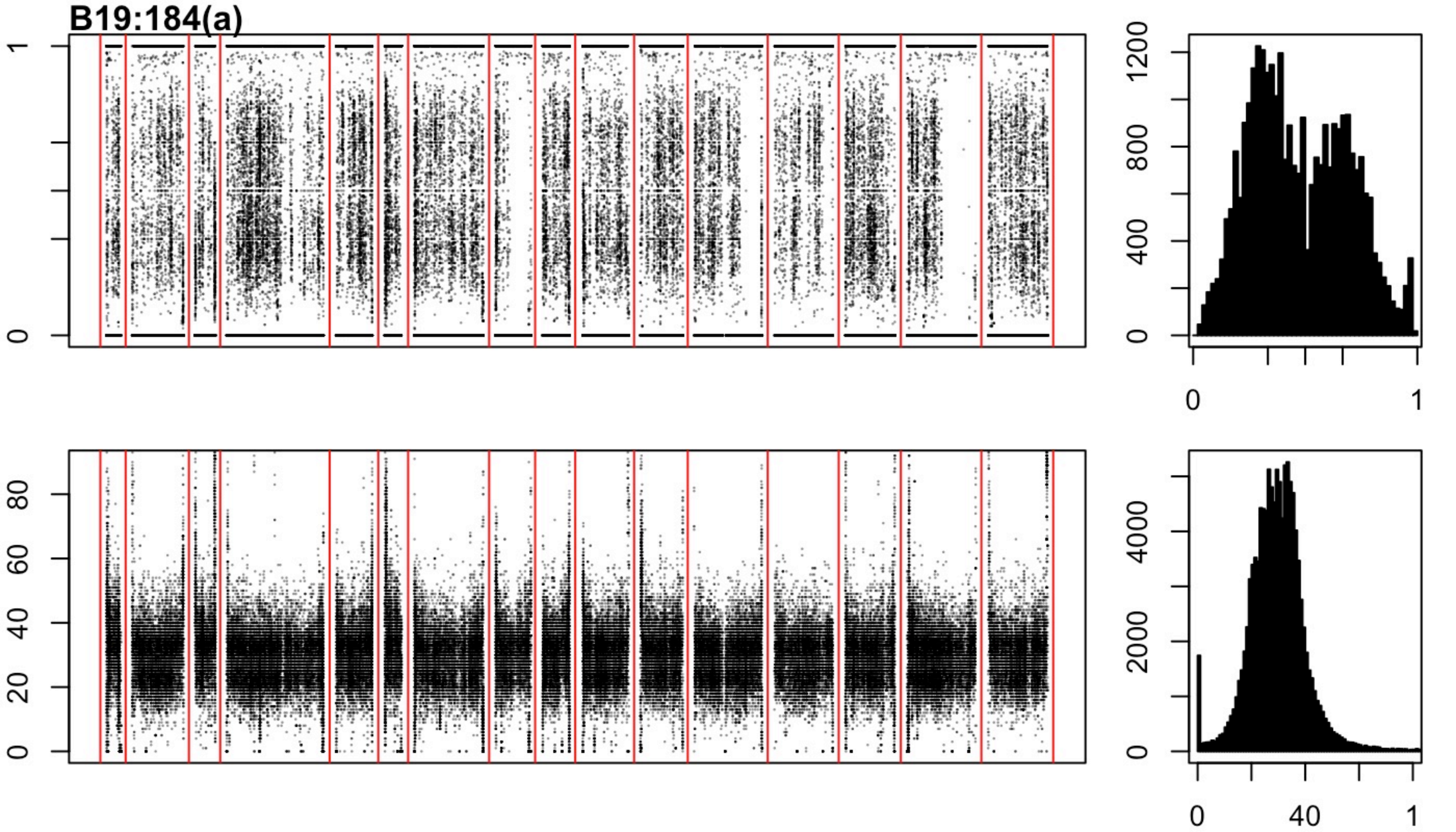
B19:184(a)
1
0
1200
800
400
0
0
1
80
60
40
20
0
4000
2000
0
0
40
1

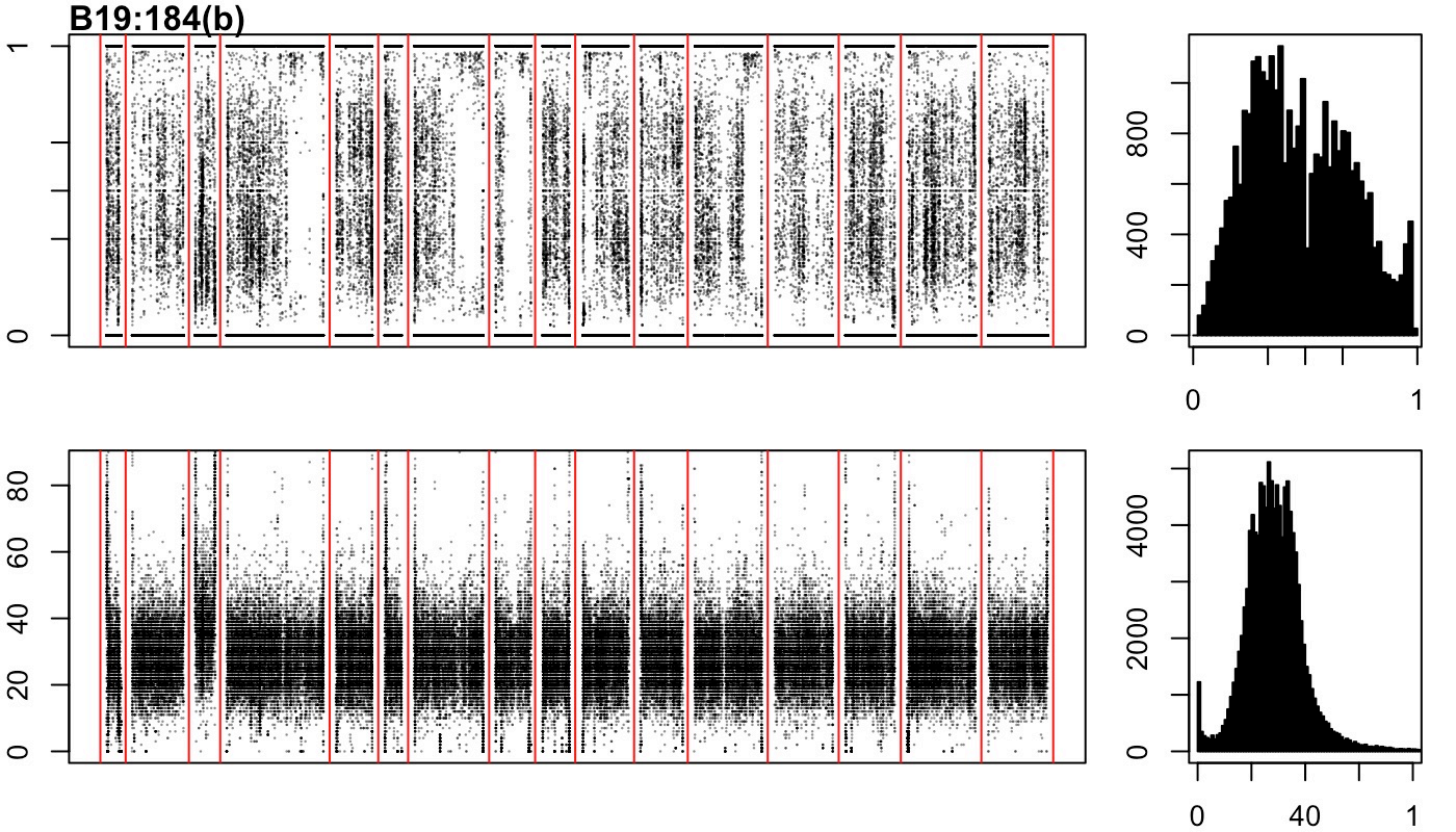
B19:184(b)
1
0
0
400
800
0
1
0
20
40
60
80
0
2000
4000
0
40
1

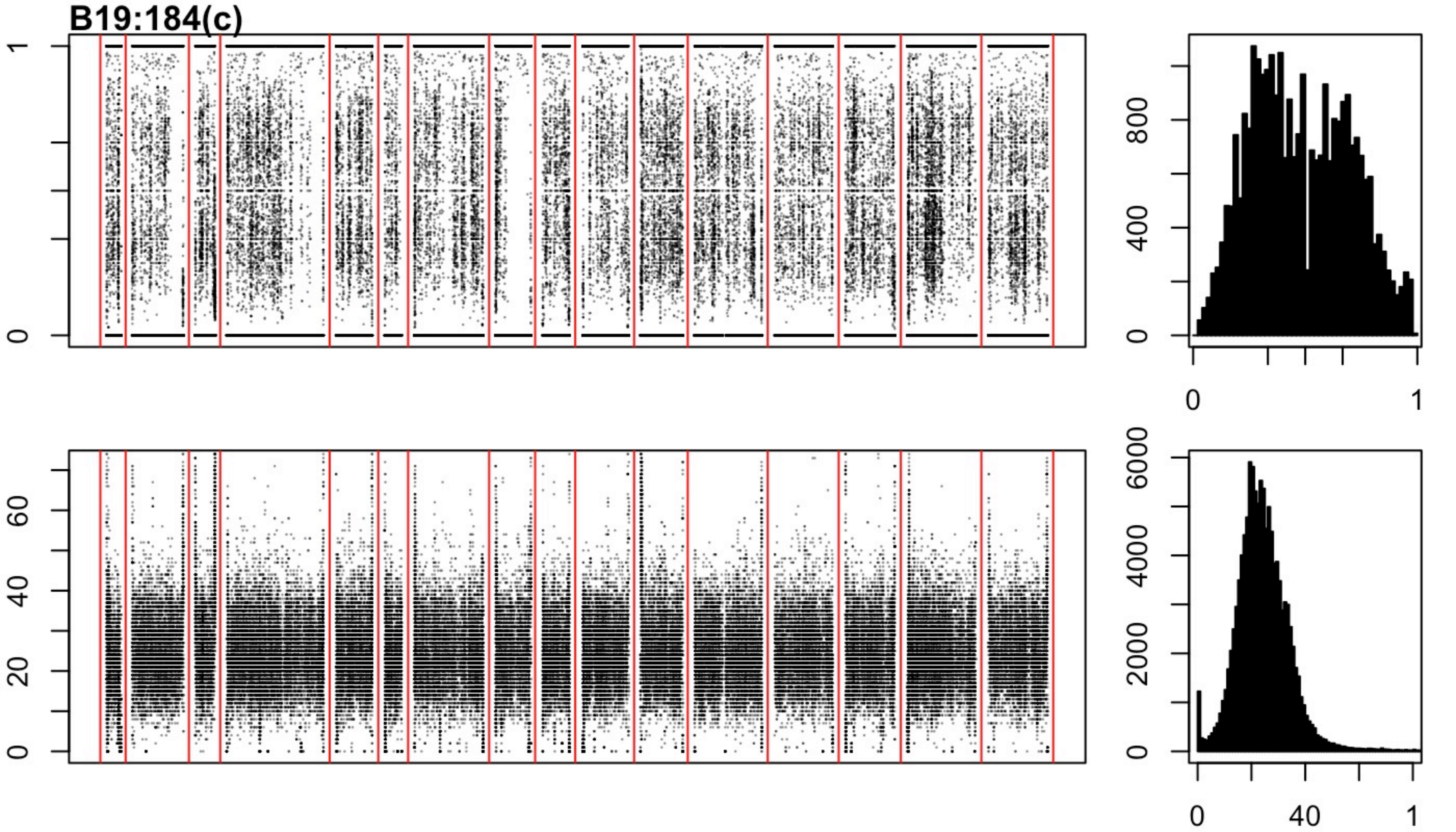

B19:184(c)
1
0
800
400
0
0
1
60
40
20
0
6000
4000
2000
0
0
40
1

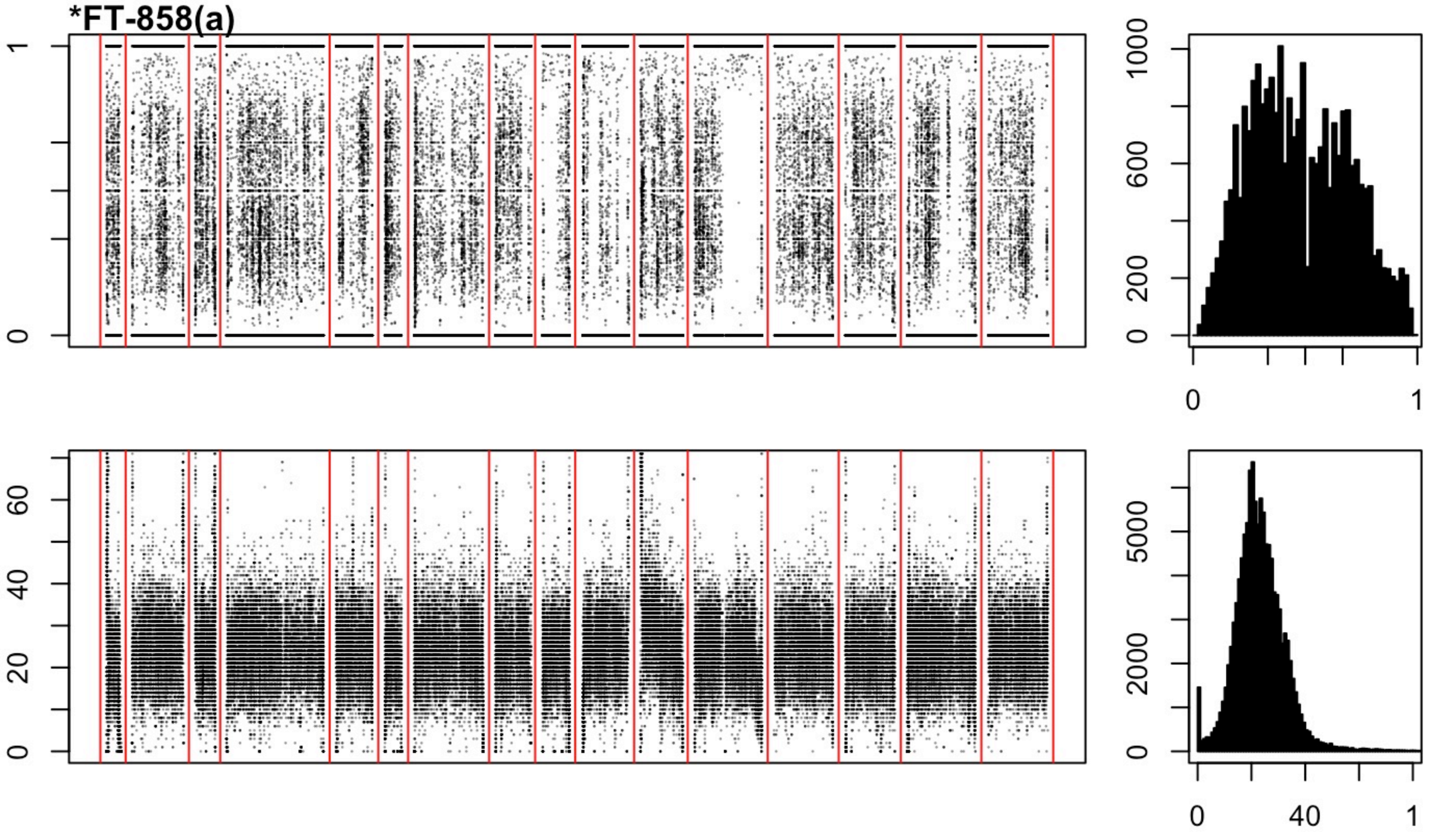
*FT-858(a)
1
0
1000
600
200
0
0
1
60
40
20
0
5000
5000
2000
0
0
40
1

*FT-858(b)

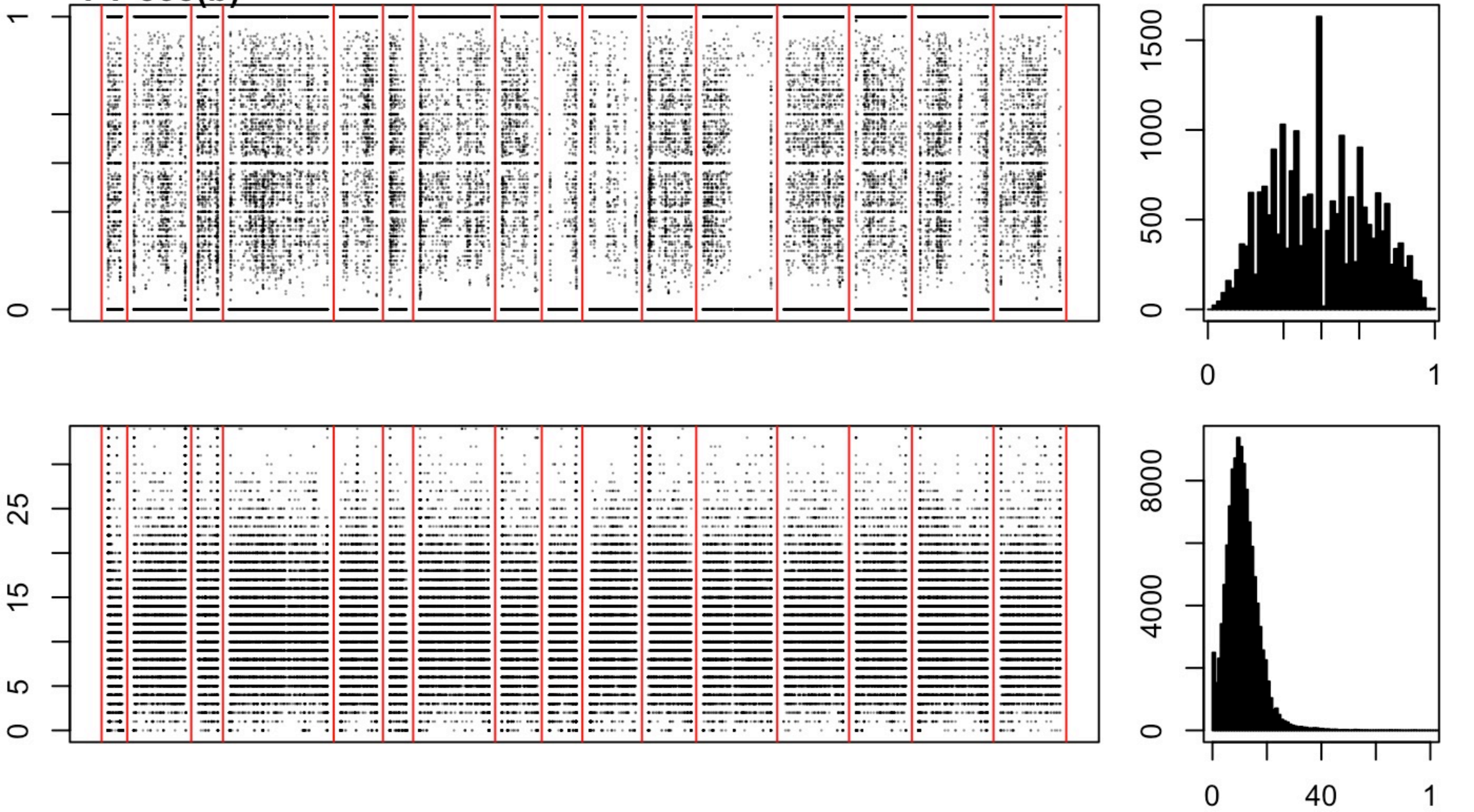

***FT-858(c)**

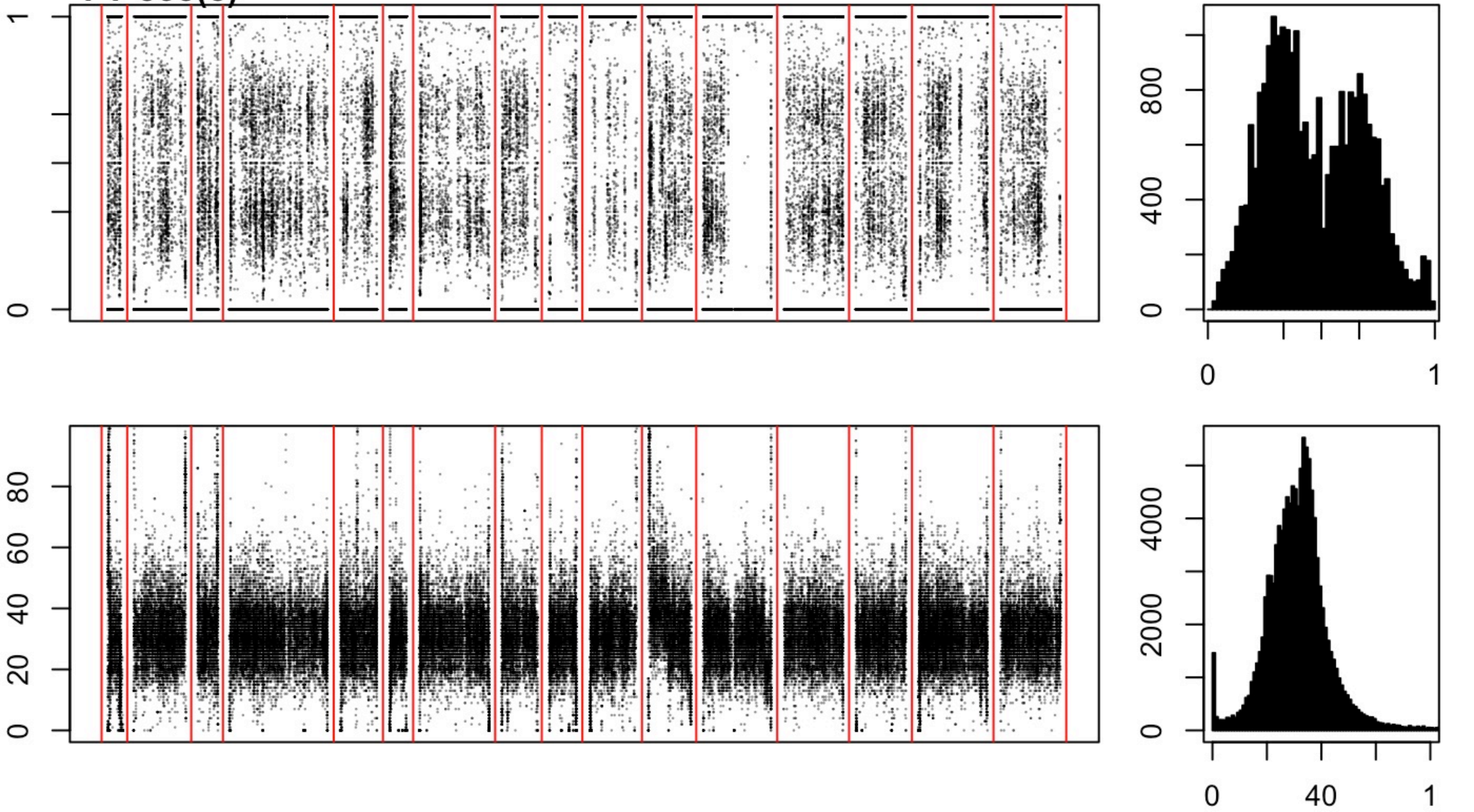

*IRA-D(a)

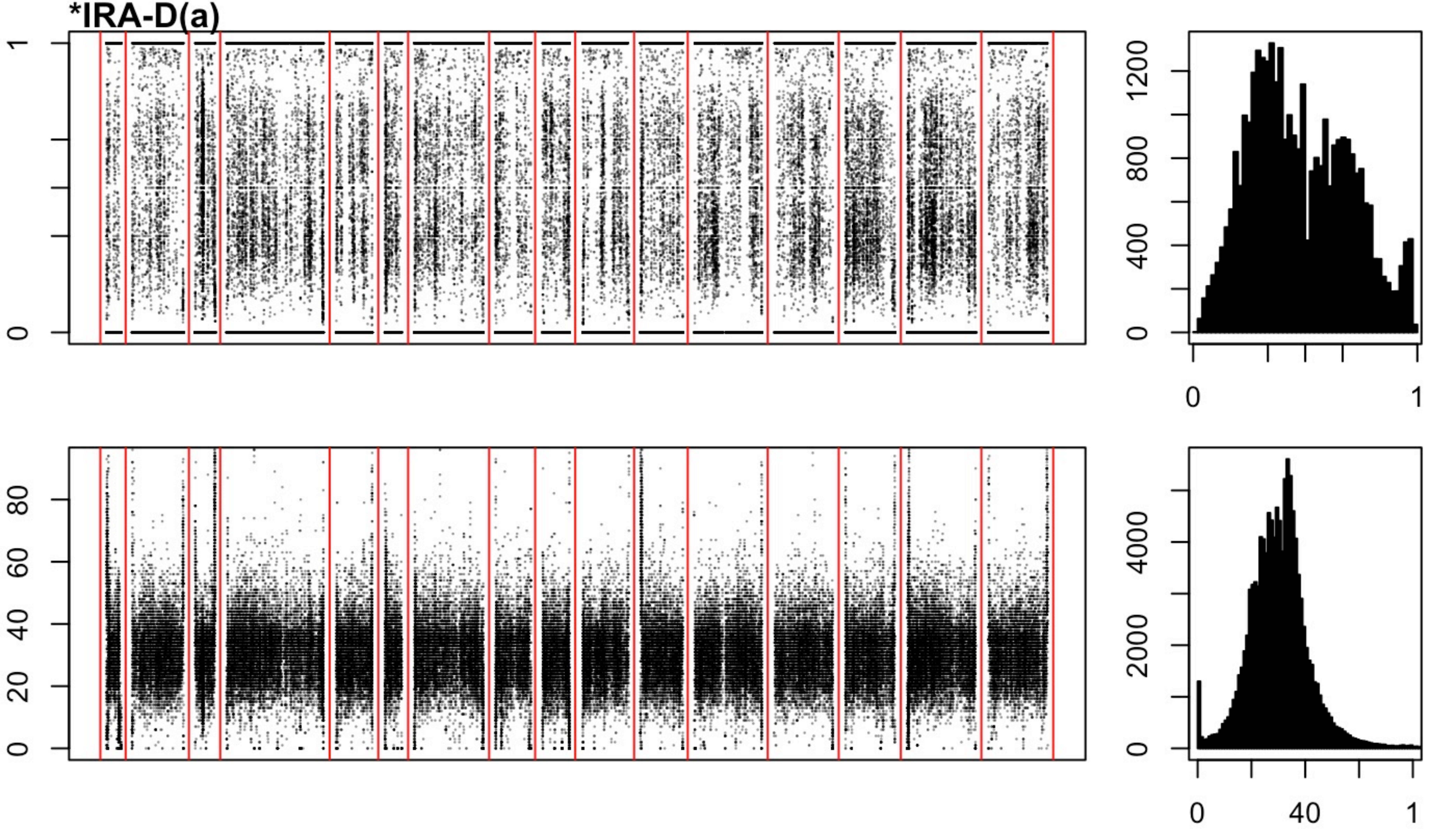

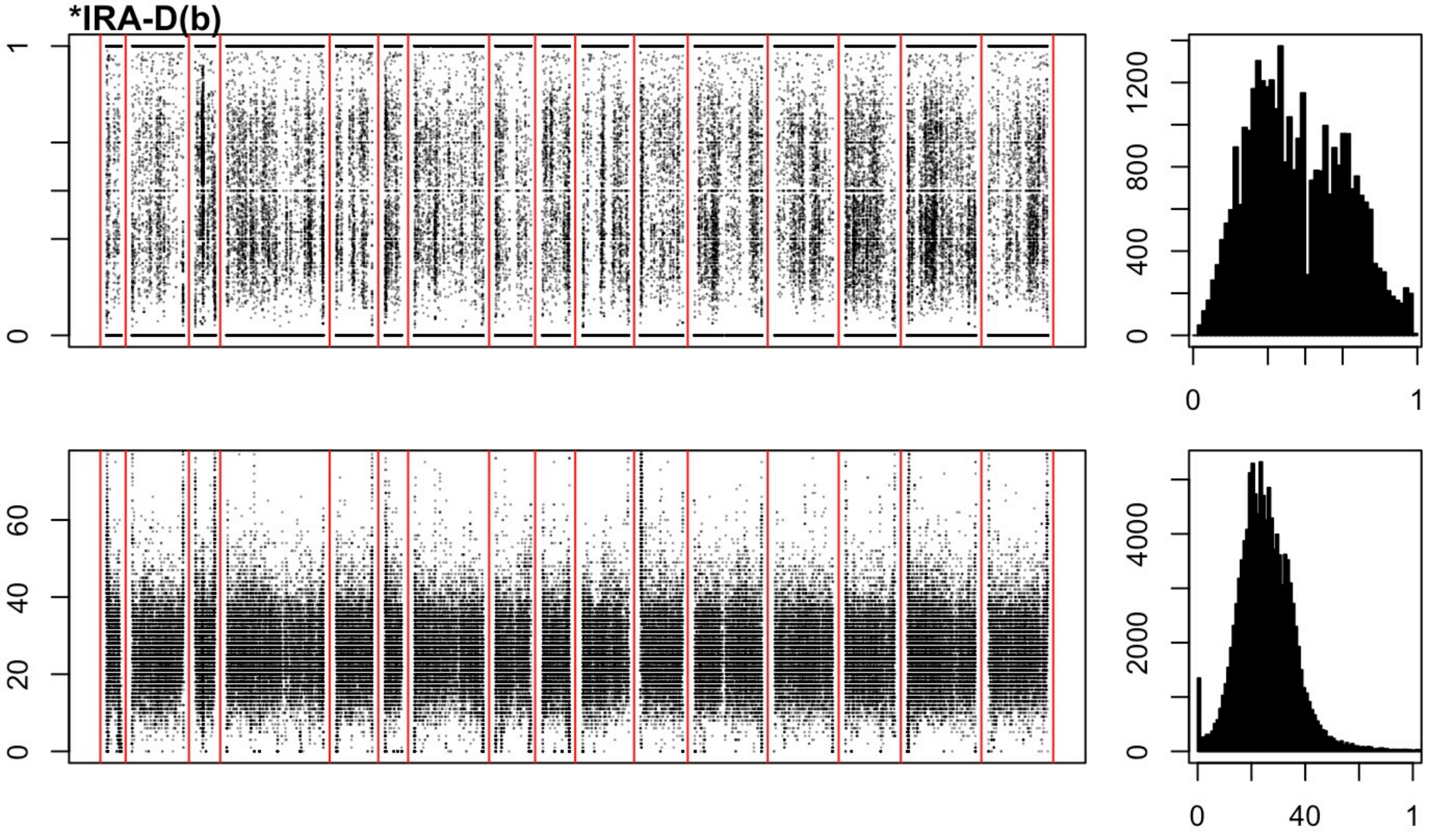
*IRA-D(b)
1
0
1200
800
400
0
0
1
60
40
20
0
4000
2000
0
0
40
1

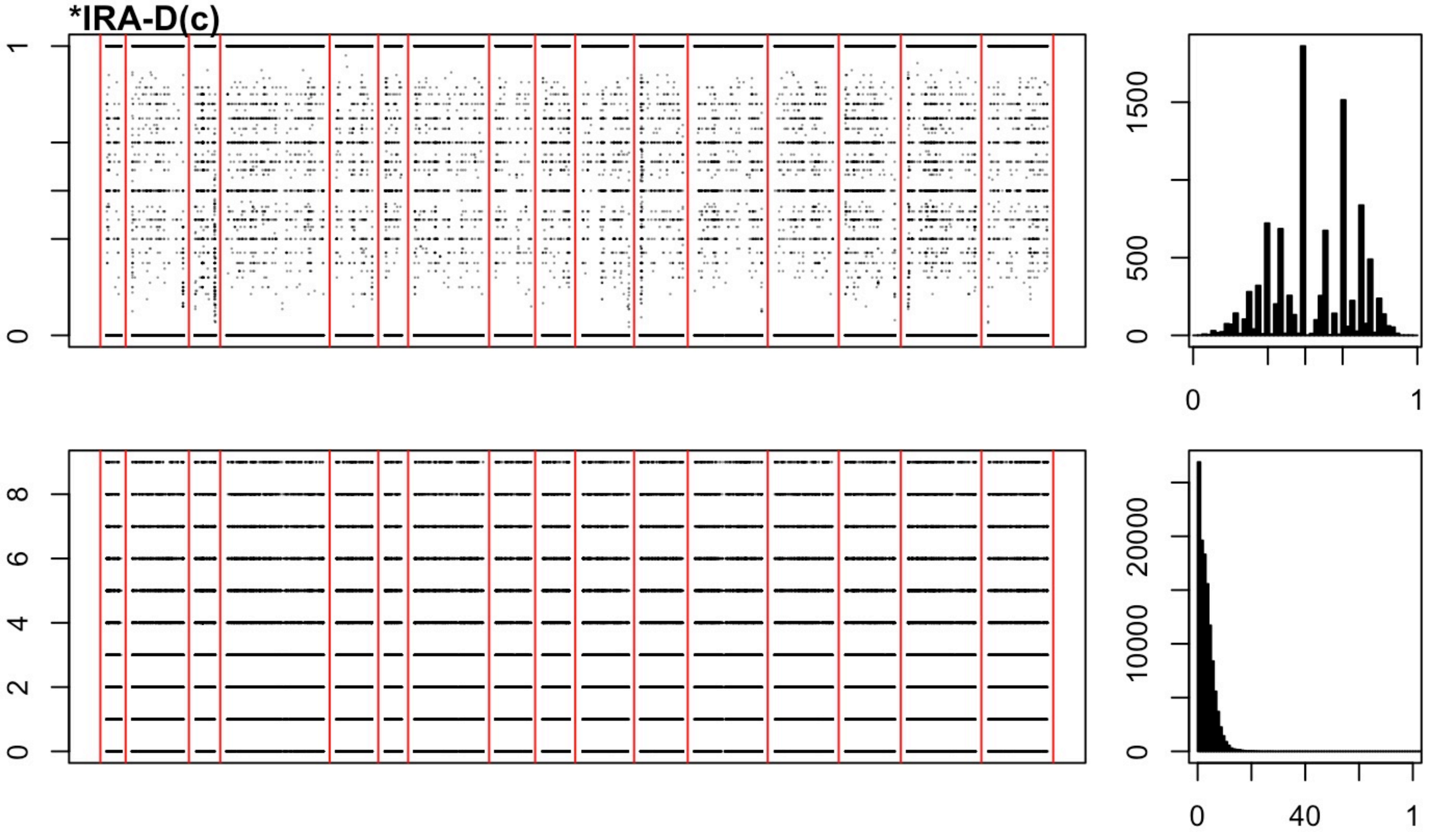

*IRA-D(c)
1
0
1500
500
0
0
1
8
6
4
2
0
20000
10000
0
0
40
1

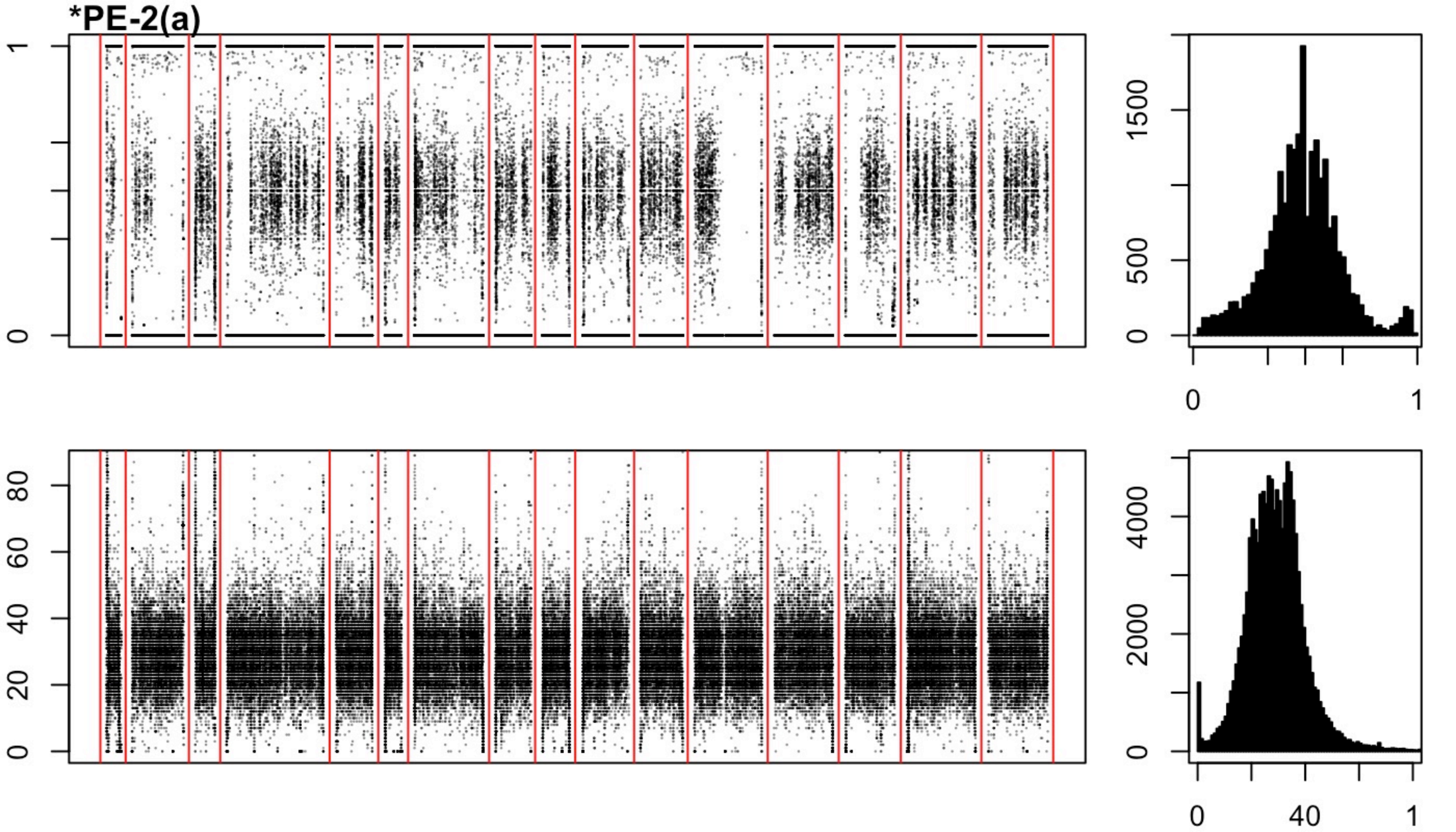
*PE-2(a)
1
0
1500
500
0
0
1
80
60
40
20
0
4000
2000
0
0
40
1

*PE-2(b)

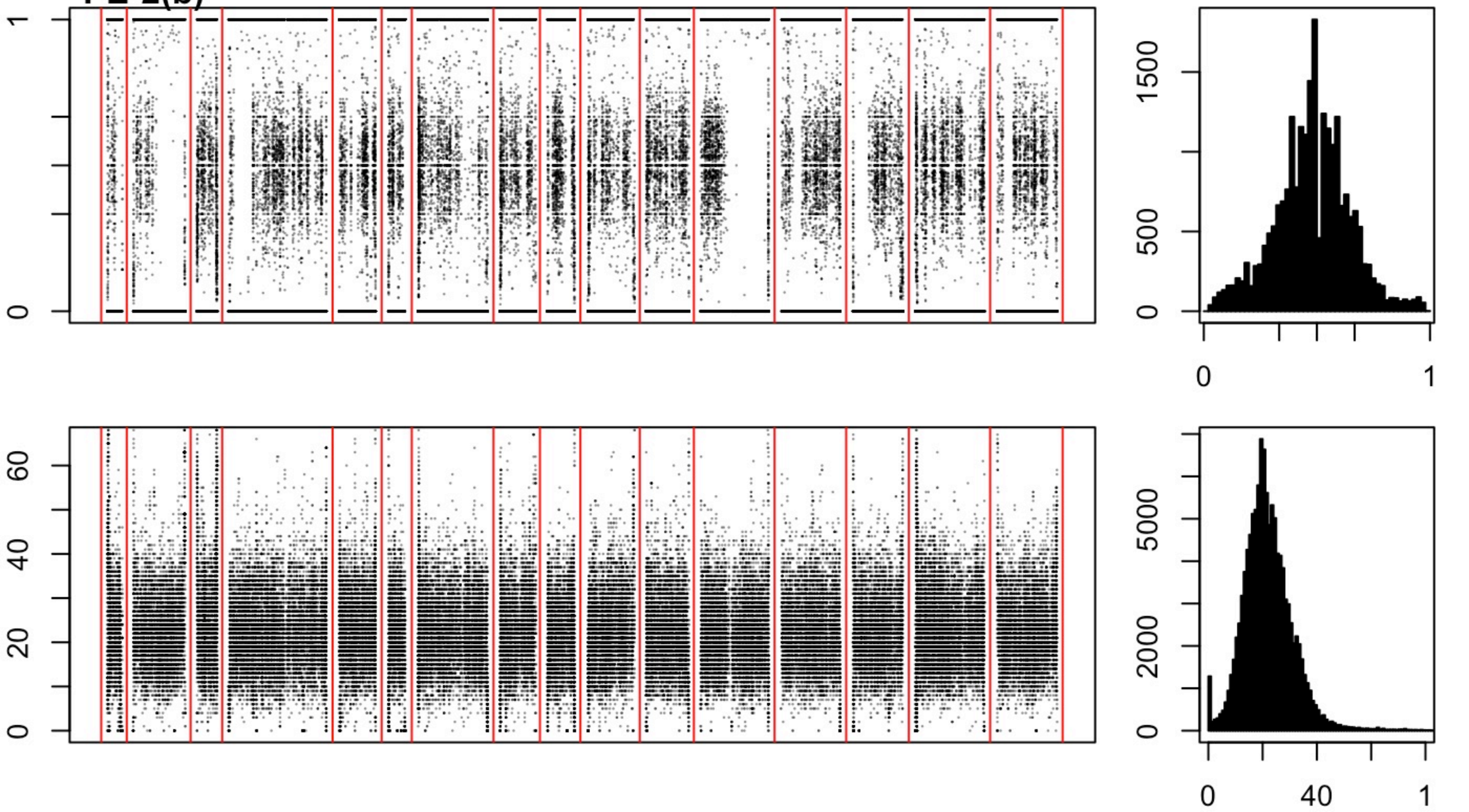

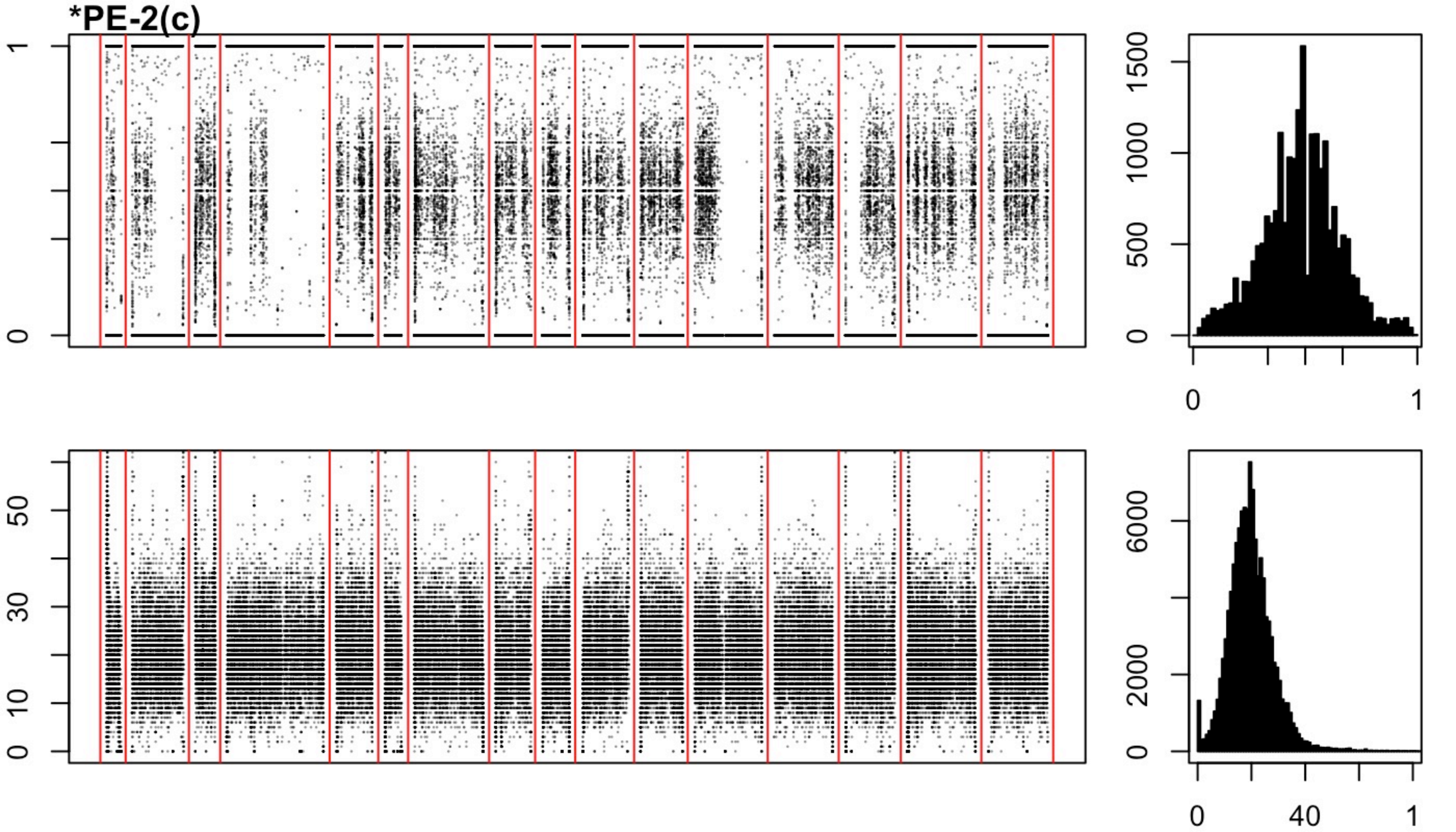
*PE-2(c)
1
0
1500
1000
500
0
0
1
50
30
10
0
6000
2000
0
0
40
1

*SA-1(a)

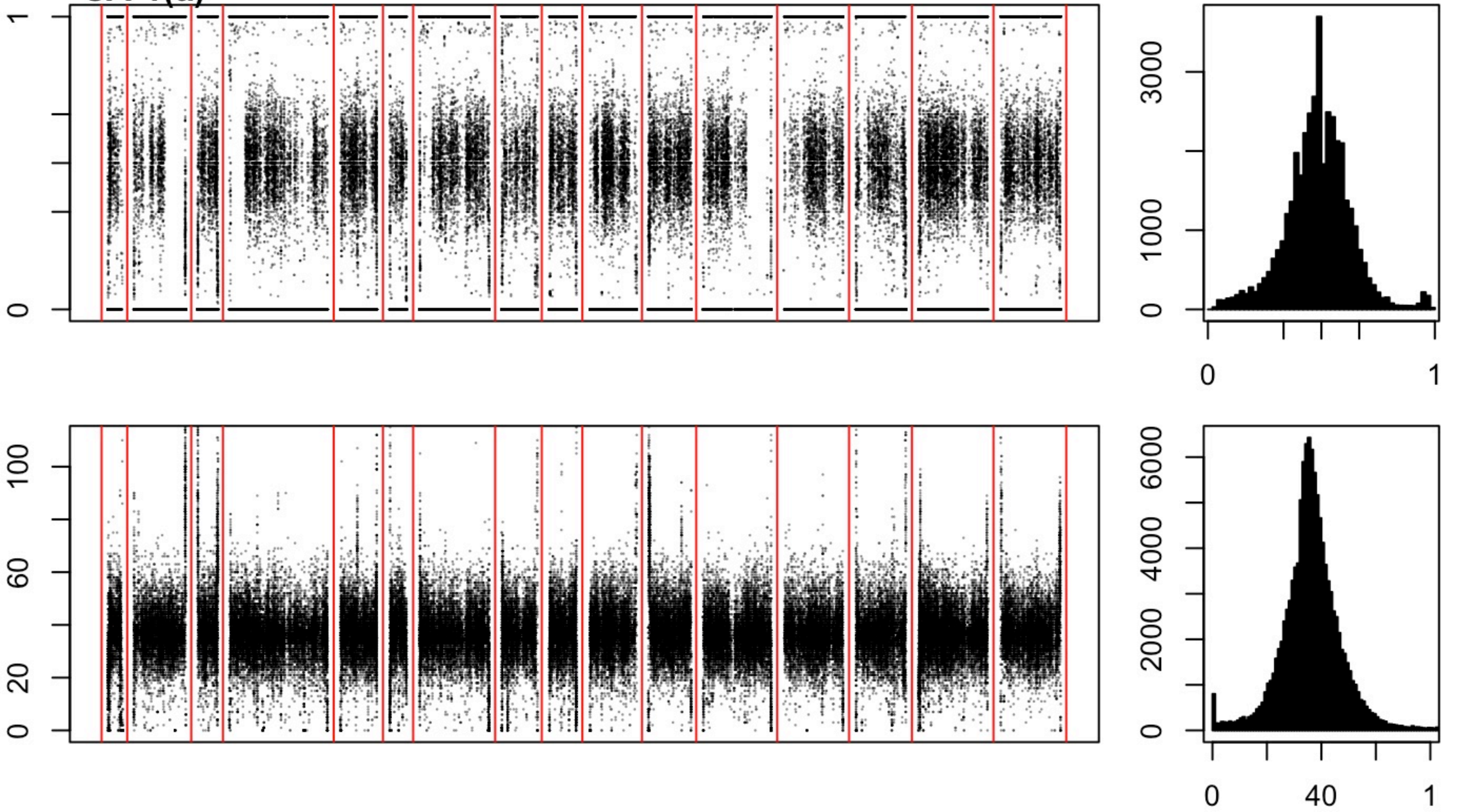

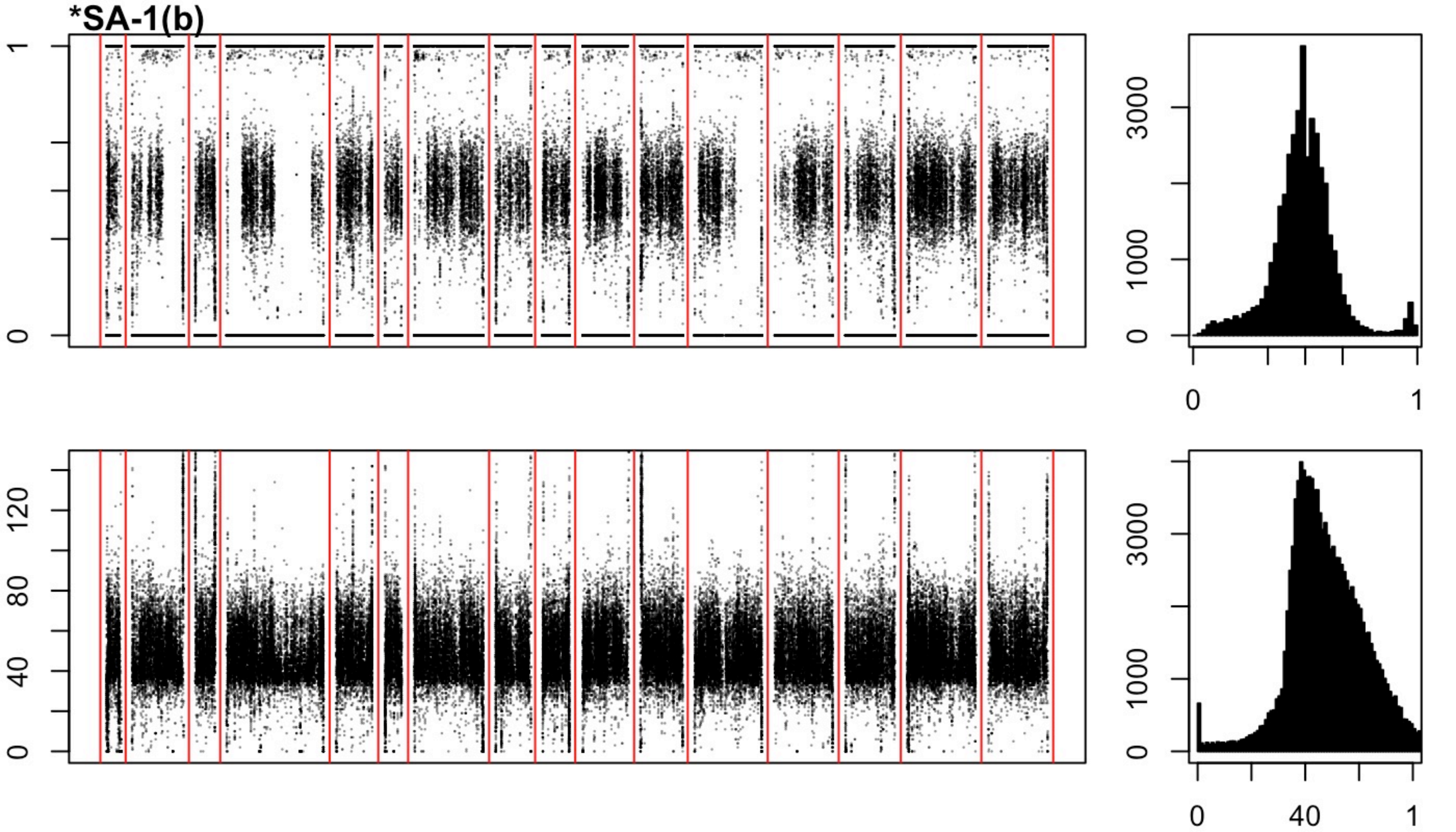
*SA-1(b)
1
0
3000
1000
0
0
1
120
80
40
0
3000
1000
0
0
40
1

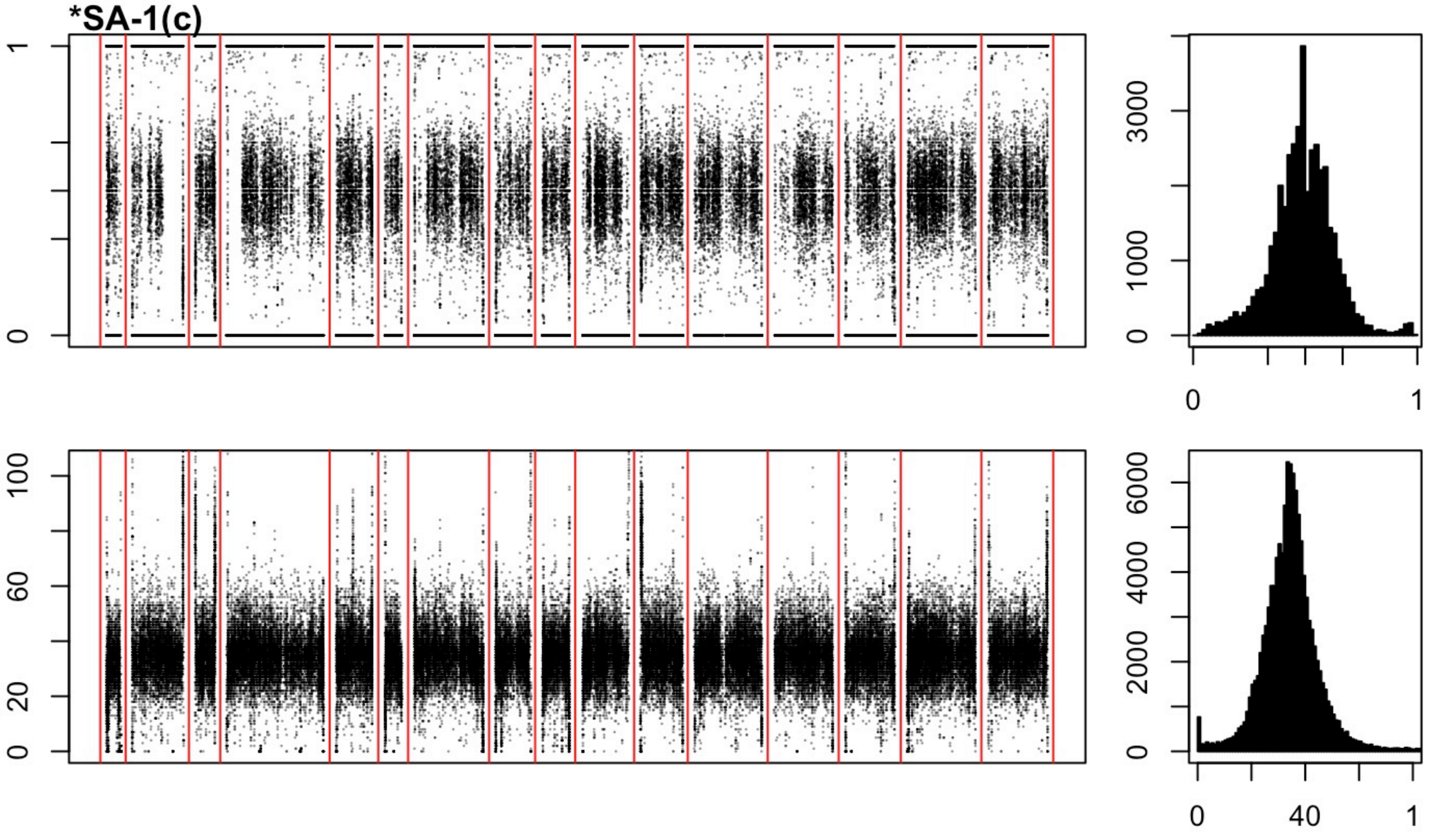
*SA-1(c)
1
0
3000
1000
0
0
1
100
60
20
0
6000
4000
2000
0
0
40
1

B18:7(a)

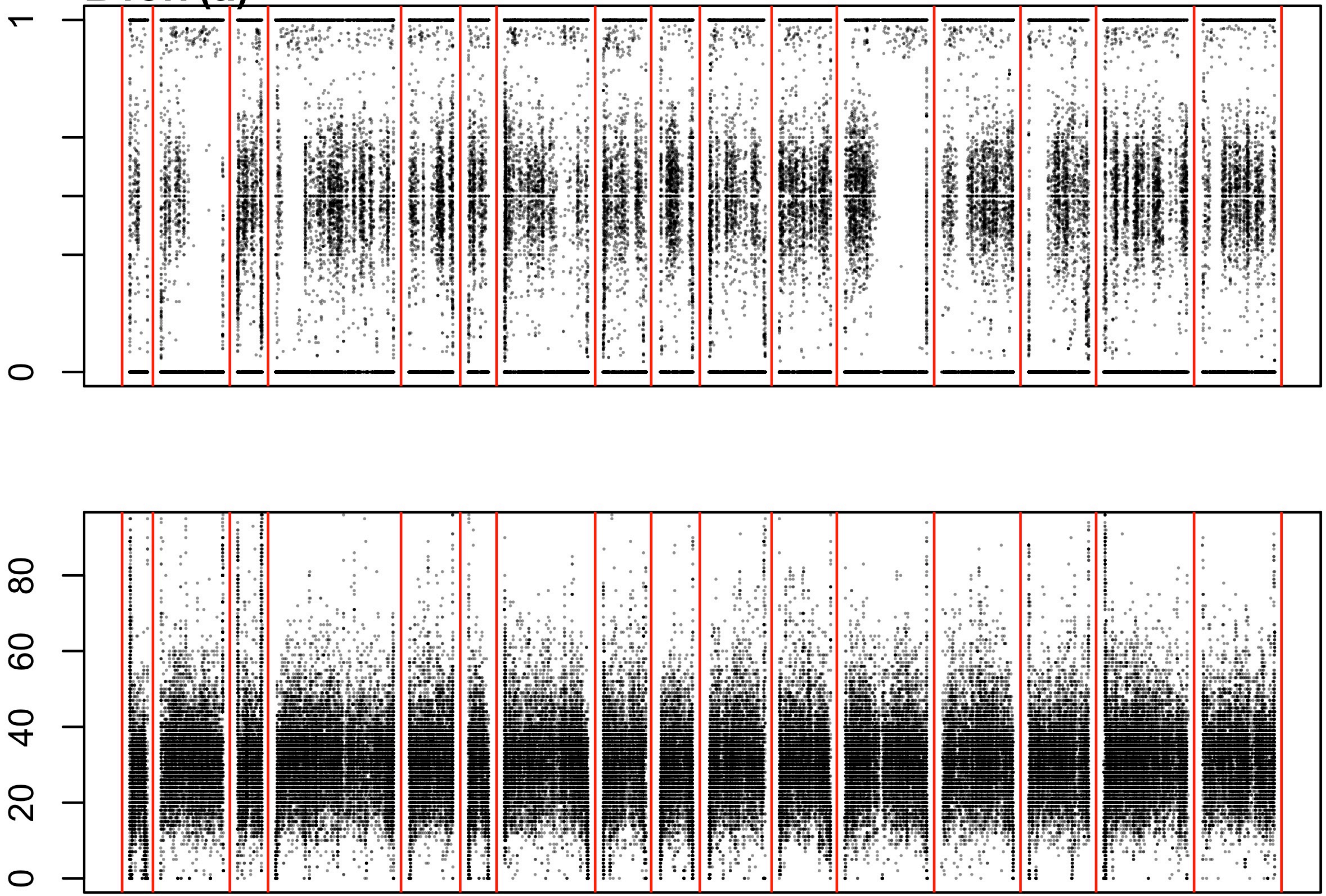

Supplement: jkad104_Supplementary_Data [file jkad104_supplementary_data.zip › File S1.pdf]
